# Potential Pandemic of H7N9 Avian Influenza A Virus in Human

**DOI:** 10.3389/fcimb.2018.00414

**Published:** 2018-11-23

**Authors:** Zhiqing Pu, Dan Xiang, Xiaobing Li, Tingting Luo, Xuejuan Shen, Robert W. Murphy, Ming Liao, Yongyi Shen

**Affiliations:** ^1^College of Veterinary Medicine, South China Agricultural University, Guangzhou, China; ^2^Joint Influenza Research Centre (SUMC/HKU), Shantou University Medical College, Shantou, China; ^3^Centre for Biodiversity and Conservation Biology, Royal Ontario Museum, Toronto, ON, Canada; ^4^Key Laboratory of Zoonosis Prevention and Control of Guangdong Province, Guangzhou, China

**Keywords:** H7N9, avian influenza A virus, human infection, genetic marker, host barrier

## Abstract

Since 2013, the H7N9 avian influenza A virus (AIV) has caused human infections and to the extent of now surpassing H5N1. This raises an alarm about the potential of H7N9 to become a pandemic problem. Our compilation of the amino acid changes required for AIVs to cross the species-barrier discovers 58 that have very high proportions in both the human- and avian-isolated H7N9 viruses. These changes correspond with sporadic human infections that continue to occur in regions of avian infections. Among the six internal viral genes, amino acid changes do not differ significantly between H9N2 and H7N9, except for V100A in PA, and K526R, D627K, and D701N in PB2. H9N2 AIVs provide internal genes to H7N9. Most of the amino acid changes in H7N9 appear to come directly from H9N2. Seventeen amino acid substitutions appear to have fixed quickly by the 5th wave. Among these, six amino acid sites in HA1 are receptor binding sites, and PB2-A588V was shown to promote the adaptation of AIVs to mammals. The accelerated fixation of mutations may promote the adaptation of H7N9 to human, but need further functional evidence. Although H7N9 AIVs still cannot efficiently transmit between humans, they have the genetic makeup associated with human infections. These viruses must be controlled in poultry to remove the threat of it becoming a human pandemic event.

## Introduction

The H7N9 avian influenza virus (AIV) is a triple reassortant virus having hemagglutinin (HA) and neuraminidase (NA) genes from migratory birds and six internal genes from chicken H9N2 AIVs (Lam et al., 2013; Liu et al., 2013). This dangerous virus has triggered five epidemics of human infections in China since 2013 (Iuliano et al., 2017; Wang et al., 2017) causing severe respiratory diseases and many fatalities (Chen et al., 2013). Although H7N9 does not have the capability for sustained human–human transmission, it has surpassed H5N1 in laboratory-confirmed human infections despite its limited dissemination outside of China (Webby and Yang, 2017). The 5th wave of human infection, which occurred from September 2016 to April 2017, started earlier than usual and saw rapid growth with 623 confirmed infections (Wang et al., 2017). This raises concerns about the potential ability of the virus to better develop an ability to infect human, and eventually attain efficient human–human infections. This would drive a global pandemic event.

Wild aquatic birds are the reservoir for AIVs and, fortunately, the viruses rarely infect humans. To infect humans, AIVs possess adaptive changes in some amino acid needed to fit the environment of their new host (Xiang et al., 2018). Molecular mechanisms that enable AIVs to cross the species-barrier and allow efficient transmission in mammals have been studied widely, and many amino acid changes have been suggested to play roles in these processes (Mertens et al., 2013; Schrauwen and Fouchier, 2014; Xiang et al., 2018). These genotypic markers facilitate assessments of the risk and likelihood of transmission from birds to mammals based on their genome sequences alone. Surveillance is crucial to track the emergence, evolution and spread of AIVs. Genotype data from the AIVs can be used further to identify and predict viruses that pose the greatest threat to human and animal health.

Herein, we assess the potential of H7N9 to drive a pandemic event. We collect all available H7N9 sequence data and identify changes at all amino acid sites known to play roles in crossing the species-barrier. This allows us to predict the potential transmission and pathogenicity in humans. Because H7N9 has frequently reassorted and attained internal genes from H9N2 viruses (Lam et al., 2015; Shi et al., 2017), we compare the distribution of six internal genes between H7N9 and H9N2. Finally, because the 5th wave had a rapid growth in cases of human infection, we assess the proportion and potential fixation of enabling amino acid changes in this wave.

## Materials and methods

### Source of sequences and preliminary treatment

All previously published genomes of H7N9 and H9N2 influenza A viruses (1994–2017) were obtained from the Influenza Virus Resource at the National Center for Biotechnology Information (NCBI) (www.ncbi.nlm.nih.gov/genomes/FLU), the Global Initiative on Sharing Avian Influenza Data (GISAID) database (www.gisaid.org) and the Influenza Research Database (FluDB) (www.FluDB.org). Redundant sequences were removed and laboratory strains were excluded. GenBank/GISAID accession numbers were listed in Supplementary Table 3. All eight gene segments were aligned separately using MAFFT v7.245(Katoh and Toh, 2010). Gblocks v0.91b (Castresana, 2000) was used to identify and remove poorly aligned positions and frame-shift errors in sequences.

### Analyses of the proportion of molecular markers

We compiled an inventory of amino acid mutations reported to be associated with changes in host tropism or increased pathogenicity in mammals. A total of 259 molecular markers were identified including 43 in PB2, 20 in PB1, 1 in PB1-F2, 44 in PA, 65 in HA, 24 in NP, 21 in NA, 10 in M1, 2 in M2, 24 in NS1, and 5 in NEP/NS2 (Supplementary Table 1). The frequencies of these amino acid changes in avian-isolated, human-isolated H7N9, and H9N2 AIVs from waves 1 to 5 were computed.

### Fixation of amino acid changes

We defined a “proportion switch” as the replacement of one major amino acid by another in successive waves. If the number of sequences with amino acid *a*_k_ at the *j*th position at wave *w* was n(*w, j*, a_k_), then the amino acid proportion *f* (*w, j*, a_k_) at the *j*th position at wave *w* was given by *f* (*w, j*, a_k_) = n(*w, j*, a_k_)/*N*(*w*) (Shih et al., 2007). For the proportions of two different amino acids at a given site *j* at wave *w, f* (*w, j*, a_m_) and *f* (*w, j*, a_n_), there was a proportion switch between *j*a_m_ and *j*a_n_ between waves *w* and *w*+1 when both of the following conditions were met: (i) *f* (*w, j*, a_m_) + *f* (*w, j*, a_n_) > 0.8 and (*w*+1, *j*, a_m_) + *f* (*w*+1*, j*, a_n_) > 0.75; and (ii) [*n*(*w, j*, a_m_) – *n*(*w, j*, a_n_)] and [*n*(*w*+1*, j*, a_m_) – *n*(*w*+1*, j*, a_n_)] had opposite signs or zero in absolute value (Shih et al., 2007). A switch in proportion at a site was recorded only when both of these conditions held.

## Results

### Changes in the proportion of 259 amino acid substitutions in H7N9 viruses

We identified 259 amino acid mutations in the literature reported to be associated with changes in host tropism or increased pathogenicity in mammals (Supplementary Table 1).

**Figure 1:**
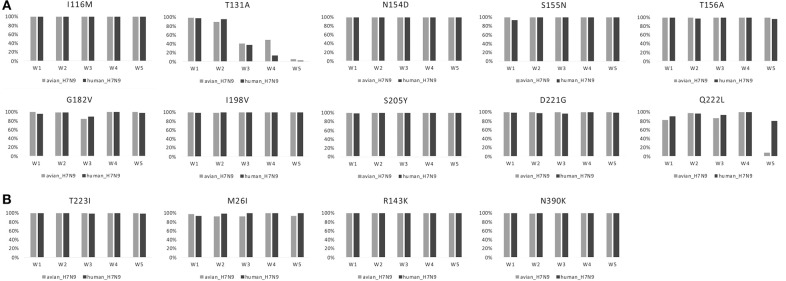
Changes in the proportion of 10 amino acid substitutions in HA and four in NA of H7N9 viruses. **(A)** HA. **(B)** NA.

**Figure 2:**
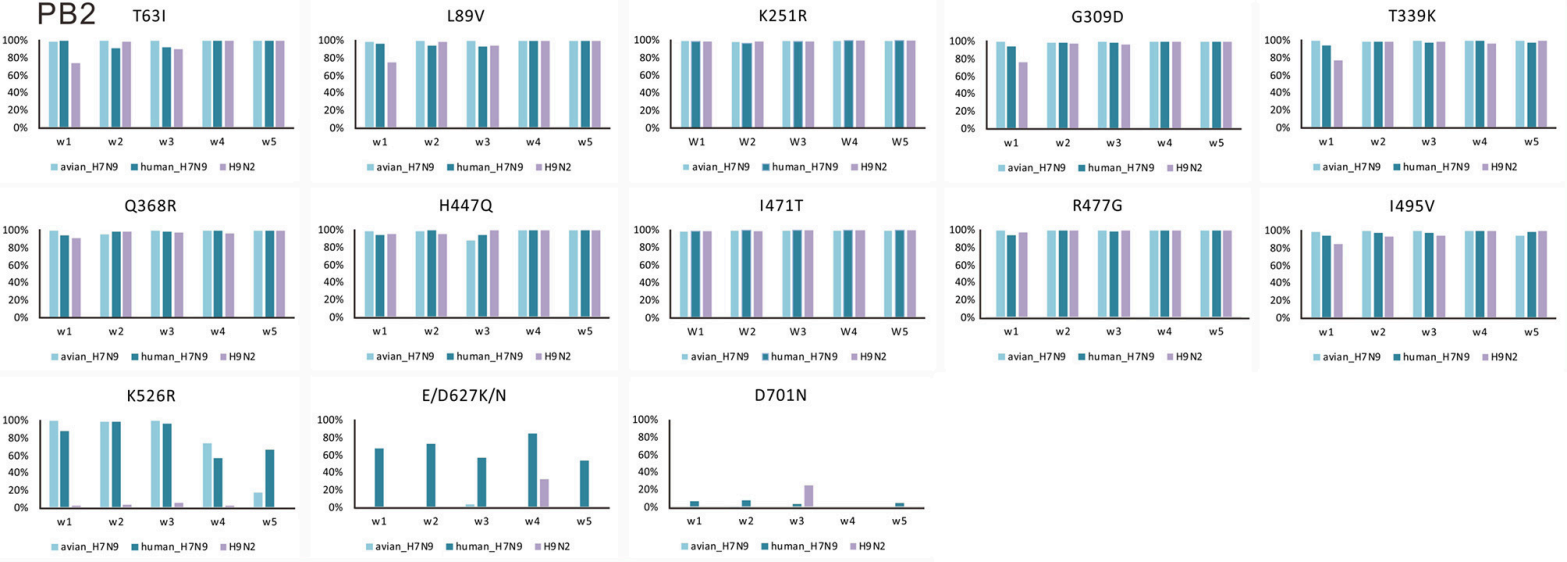
Changes in the proportion of 13 amino acid substitutions in PB2 of H7N9 and H9N2 viruses.

Amino acid changes S155N, T156A, G182V, S205Y, and Q222L (H5 numbering) in HA had very high proportions in both human- and avian-isolated H7N9 (Figure 1A). These changes increased virus-binding to α-2,6 sialo-saccharides. Several mutations have been suggested to enhance polymerase activity and increase virulence in mammals. These were also found to have very high proportions in both human- and avian-isolated H7N9 (Supplementary Table 2) as follows: three amino acids in HA (I116M, N154D, I198V, Figure 1A); four in NA (M26I, R143K, T223I, N390K, Figure 1B); 10 in PB2 (T63I, L89V, K251R, G309D, T339K, Q368R, H447Q, I471T, R477G, I495V, Figure 2); 11 in PB1 (A3V, L13P, R207K, K328N, I368V, S375N/T, H436Y, A469T, L473V, V652A, M677T, Supplementary Figure 1); nine in PA (V135A, H266R, F277S, C278Q, K356R, N383D, S409N, S/A515T, L653P, Supplementary Figure 2); five in MP (V15I/T, N30D, T215A, S31N, L55F, Supplementary Figure 3a); and six in NS (A/P42S, F103L, M106I, V149A, N200S, T47A, Supplementary Figure 3b); and four in NP (V41I, D210E, F253I, I353V, Supplementary Figure 3c).

E/D627K/N and D701N in PB2 had much higher proportions in human-isolated than avian-isolated H7N9 in all 5 waves (Figure 2). In waves 1–4, Q222L in HA and K526R in PB2 had high proportions in both human- and avian-isolated H7N9. In wave 5, these sites had high proportions in human-isolated H7N9 (80.30 and 66.87%, respectively), but low proportions in avian-isolated viruses (9.40 and 17.39%, respectively). Further examination of wave 5 showed that both mutations had very low proportions (0 and 9.52%, respectively) in high-pathogenic (HP) avian-isolated H7N9, but were fixed (100%) in low-pathogenic (LP) viruses.

In comparisons of H7N9 and H9N2, the former has high proportions of the amino acid changes V100A in PA and K526R, E/D627K/N, and D701N in PB2. In comparison, one in three sequences (33.33%) of H9N2 has amino acid change E/D627K/N in wave 4, and one in four sequences (25%) have change D701N in wave 3. However, the relatively high proportions of these two mutations may reflect a bias of having few available sequences.

### Fixation of 17 amino acid sites in the 5th wave

**Figure 3:**
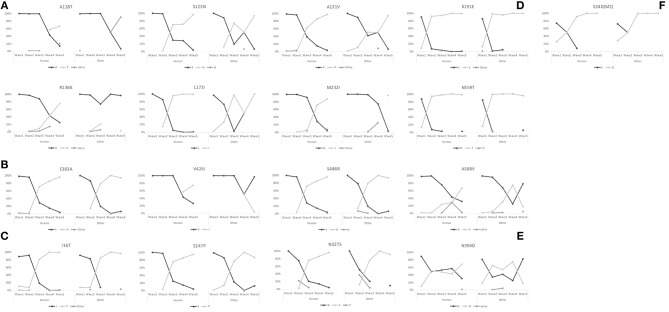
Proportion switch of 17 amino acid changes that tended to fix in the 5th wave. **(A)** Sites A118T, S123N, A131V, R136K, L173I, and M232I in HA1 (H5 numbering). **(B)** Sites E383A, V426I, and S486R in HA2 (H5 numbering). **(C)** Sites I16T, S247P, and N327S in NA (N9 numbering). **(D)** Sites K191E, N559T, and A588V in PB2 (N9 numbering). **(E)** Site N394D in PA (N9 numbering). **(F)** Site E24D in M2 (N9 numbering).

A trend for fixation occurred in the 5th wave in 17 sites: A118T, S123N, R136K, A131V, L173I, and M232I in HA1 (H5 numbering); E383A, V426I, and S486R in HA2 (H5 numbering); I16T, S247P, and N327S in NA (N9 numbering); N394D in PA (N9 numbering); E24D in M2 (N9 numbering); and K191E, N559T and A588V in PB2 (N9 numbering) (Figure 3). Amino acid changes R136K in HA1, V426I in HA2, N394D in PA, and A588V in PB2 showed much higher proportions in human-isolated than avian-isolated H7N9 in the 5th wave.

**Figure 4:**
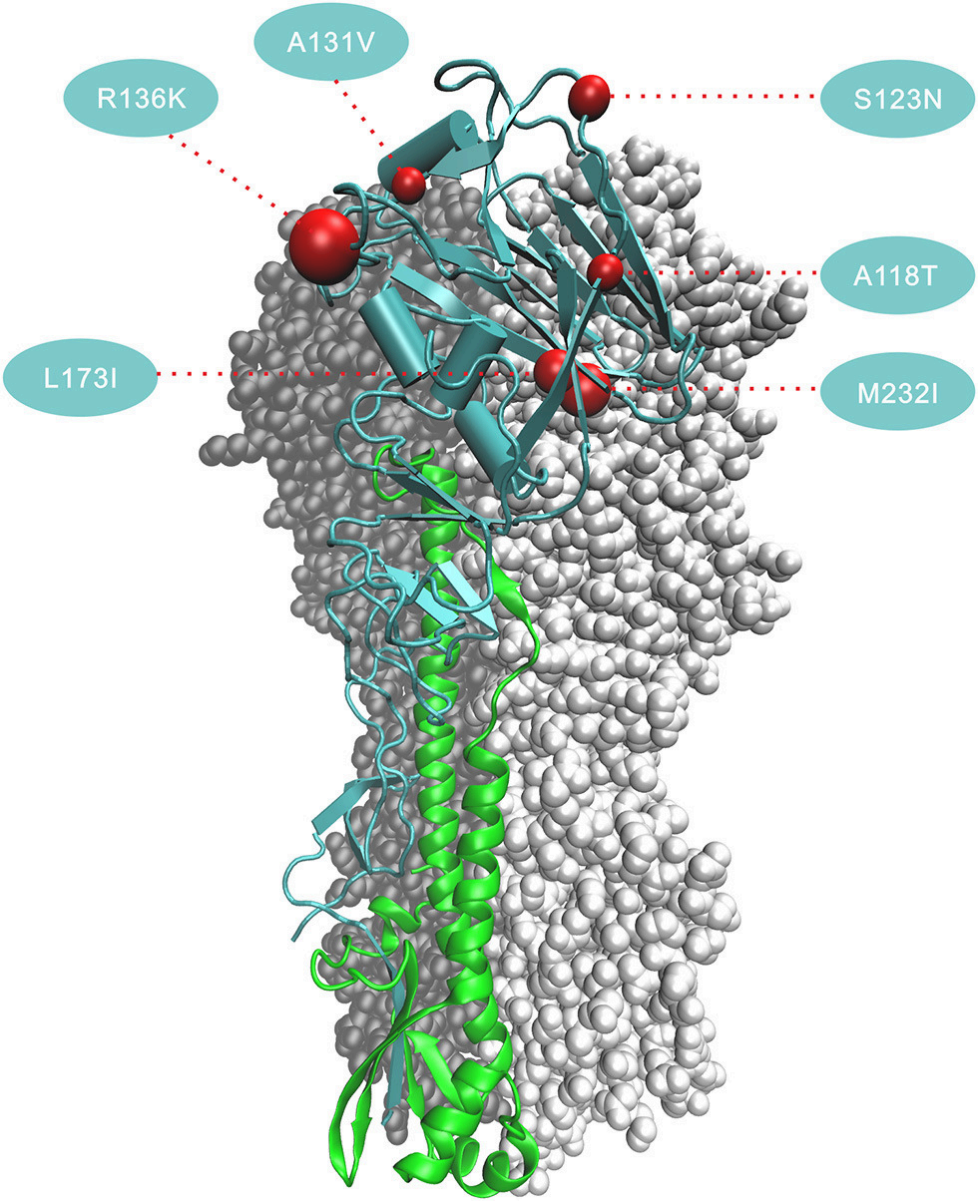
Positions of the six amino acid changes that fixed in the 5th wave in HA1 in the 3D structure of HA. The three-dimensional structure of HA is from avian-origin H7N9 influenza virus (A/Shanghai/1/2013) [PDB code: 4LCX]. Monomer shows subunit HA1 in blue and subunit HA2 in green. Numbers in the colored circles denote codon alignment number and red spheres show their locations in the three-dimensional structure. All amino acid changes occurring in HA1 (A118T, S123N, A131V, R136K, L173I, and M232I) locate in the receptor-binding region and in close proximity to the antigenic site.

The six amino acid substitutions in HA1 occurred at receptor binding sites; these mapped to the three-dimensional (3D) protein structures of the HA [Protein Data Bank (PDB) code:4LCX(Shi et al., 2013)] using VMD v1.9.3 (Humphrey et al., 1996) (Figure 4). Substitutions S123N and R136K began in the 2nd wave, and continued to increase in proportion in subsequent waves. The substitution ratios of A118T and M232I increased from zero in the first three waves to 50% (A138T) and 67% (M232I) in the 4th wave and then 76% (A138T) and 85% (M232I) in the 5th wave. In HA2, the proportions for the amino acid changes E383A and S486R rose from the 2nd wave to the 5th wave, and V426I started to increase starting with the 4th wave.

Substitutions I16T and S247P in NA had very low proportions in the 1st wave with (6.54 and 0.38%, respectively), but then their proportions increased quickly reaching 100 and 97.43%, respectively, by the 5th wave. Amino acid change N327S was first identified in the 2nd wave at a proportion of 9.90%. This change increased to 97.44%, and almost replaced 327N (2.56%) by the 5th wave.

## Discussion

Recent research on gain-of-function has identified genetic changes required for AIVs to cross the species-barrier and adapt to replication and transmission in mammals. Our compilation of these genetic changes (Supplementary Table 1) and surveillance data serve to predict that H7N9 poses a great threat to humans.

AIVs must adapt to their new host (humans) to infect successfully. To initiate infection, hemagglutinin (HA), which is the major surface glycoprotein of influenza viruses, binds to host cell surface complex glycans via a terminal sialic acid. The switch of the preference from avian- to human-type sialic acid receptors (α-2,6 sialo-saccharides) is a key element necessary for AIVs to cause human pandemics (Matrosovich et al., 2000). Amino acid changes S155N, T156A, G182V, S205Y, and Q222L (H5 numbering) in HA increase virus-binding to human α-2,6 sialo-saccharides (Suzuki et al., 1989; Yamada et al., 2006; Wang et al., 2010; Herfst et al., 2012; Imai et al., 2012). These changes occur in very high proportions in both human- and avian-isolated H7N9 viruses (Figure 1). This suggests that H7N9 viruses have an ability to bind to human-type receptors because receptor-binding specificity assays have shown that the human-isolated H7N9 viruses can bind to both avian-type (α2,3-linked sialic acid) and human-type (α2,6-linked sialic acid) receptors (Zhou et al., 2013).

A series of amino acid changes suggested to enhance polymerase activity and increase virulence in mammals have very high proportions in both the human- and avian-isolated H7N9 viruses (Supplementary Table 2). This further suggests that avian-isolated H7N9 viruses may have potential ability to infect human. H7N9 AIVs not only increase their receptor binding properties, but also enhance their polymerase activity and, thus, increase their virulence in mammals. This best explains the continuing, sporadic occurrence of human infections in China since its emergence in 2013. This also raises a red flag with respect to their continuing threat to public health.

Amino acid changes E/D627K/N and D701N in PB2 were shown to be critical adaptations for infecting mammals (Subbarao et al., 1993; Hatta et al., 2001; Gabriel et al., 2005; Li et al., 2005). They have much higher proportions in human-isolated H7N9 viruses than in avian-isolated forms in all five waves (Figure 1). Some H7N9 viruses transitioned from low to high pathogenicity in poultry by the 5th wave (Ke et al., 2017; Shi et al., 2017; Zhang et al., 2017). Mutations Q222L in HA and K526R in PB2 show very low proportions (0 and 9.52%, respectively) in HP avian-isolated H7N9 viruses, yet 100% in LP viruses. Thus, HP and LP H7N9 viruses may prefer different amino acids in these two sites. A founder effect of HP H7N9 viruses may provide an alternative explanation.

Six internal genes of H9N2 AIVs have very high proportions in a series of amino acid changes that associate with changes in host tropism or increased pathogenicity (Supplementary Table 2). This suggests that H9N2 also potentially possess the ability to infect human. H9N2 AIVs isolated in recent years preferentially bound to human-like receptors (Li et al., 2014; Kang and Jang, 2017), and sporadic human infections have been reported (Peiris et al., 1999; Butt et al., 2005; Gu et al., 2017). For these sites, except for V100A in PA, and K526R, D627K, and D701N in PB2, H9N2, and H7N9 do not differ significantly at other amino acid changes. H9N2 provided internal genes to H7N9 (Lam et al., 2015; Pu et al., 2015; Gu et al., 2017; Shi et al., 2017; Yang et al., 2017). Thus, most of the amino acid changes in H7N9 came directly from H9N2. E627K in PB2, which is the best characterized mammalian adaptive mutation (de Wit et al., 2010), has very high proportions only in human-isolated H7N9 viruses. The D701N substitution in PB2 was found to increase virulence, and to expand the host range of avian H5N1 to mammals in the absence of E627K (Steel et al., 2009). A few human-isolated H7N9 viruses have this amino acid change, which suggests that some adaptive amino acid changes occurred during their host-shift.

The 5th wave of human infection saw a rapid growth in the number of cases (Wang et al., 2017). Have H7N9 AIVs experienced an accelerated fixation of beneficial adaptations to the environmental of human? In addition to the known amino acid mutations associated with changes in host tropism or increased pathogenicity in mammals, our analyses detect a trend for the fixation of 17 amino acid changes in the 5th wave (Figure 3). Among these, six amino acids sites substituted in HA1 locate at receptor binding region (Figure 4). Amino acid change PB2-A588V which showed much higher proportion in human-isolated than avian-isolated H7N9 in the 5th wave, was proved to promote the adaptation of H7N9 to mammals (Xiao et al., 2016). This might explain the increased number of human infection cases in the 5th wave. The function of other sites needs further experimental study.

H7N9 AIVs have continuously evolved since 2013. Although they have triggered five epidemics of human infections, they still do not have efficient human-human transmission, and only fix to avian. Our analyses identify a series of amino acid changes that associate with cross-species transmission from avian to human in high proportions in both human- and avian-isolated H7N9 AIVs. Avian-isolated H7N9 viruses have the genetic makeup associated with human infections and this suggests that if we cannot control this subtype in poultry, an impending human pandemic is still on the doorstep.

## Author contributions

YS conceived, designed, and supervised the study. ZP, DX, XL, TL, and XS collected and analyzed the data. YS and RM wrote the drafts of the manuscript. ML commented on and revised drafts of the manuscript. All authors read and approved the final report.

### Conflict of interest statement

The authors declare that the research was conducted in the absence of any commercial or financial relationships that could be construed as a potential conflict of interest.

## Supplementary material

The Supplementary Material for this article can be found online at: https://www.frontiersin.org/articles/10.3389/fcimb. 2018.00414/full#supplementary-material

Supplementary Figure 1 Changes in the proportion of 11 amino acid substitutions in PB1 of H7N9 and H9N2 viruses.

Supplementary Figure 2 Changes in the proportion of 10 amino acid substitutions in PA of H7N9 and H9N2 viruses.

Supplementary Figure 3 Changes in the proportion of five amino acid substitutions in MP, six in NS and four in NP of H7N9 and H9N2 viruses. **(a)** MP. **(b)** NS. **(c)** NP.

Supplementary Table 1 Genetic changes reported to be associated with changes in host tropism or increased pathogenicity in mammals. The H5 numbering system was used to identify nucleotide positions in our analysis.

Supplementary Table 2 The proportion of 63 amino acid substitutions that associated with AIVs to infect mammals in H7N9 and H9N2 viruses. 58 of them have very high proportions not only in human-isolates, but also in avian-isolated H7N9 AIVs.

Supplementary Table 3 GenBank/GISAID accession numbers of viruses that used in this study.
