# Supplementary figures and images for: Potential Pandemic of H7N9 Avian Influenza A Virus in Human
Source: Front Cell Infect Microbiol. 2018 Nov 23;8:414. doi: 10.3389/fcimb.2018.00414 (PMC6265602; doi:10.3389/fcimb.2018.00414)

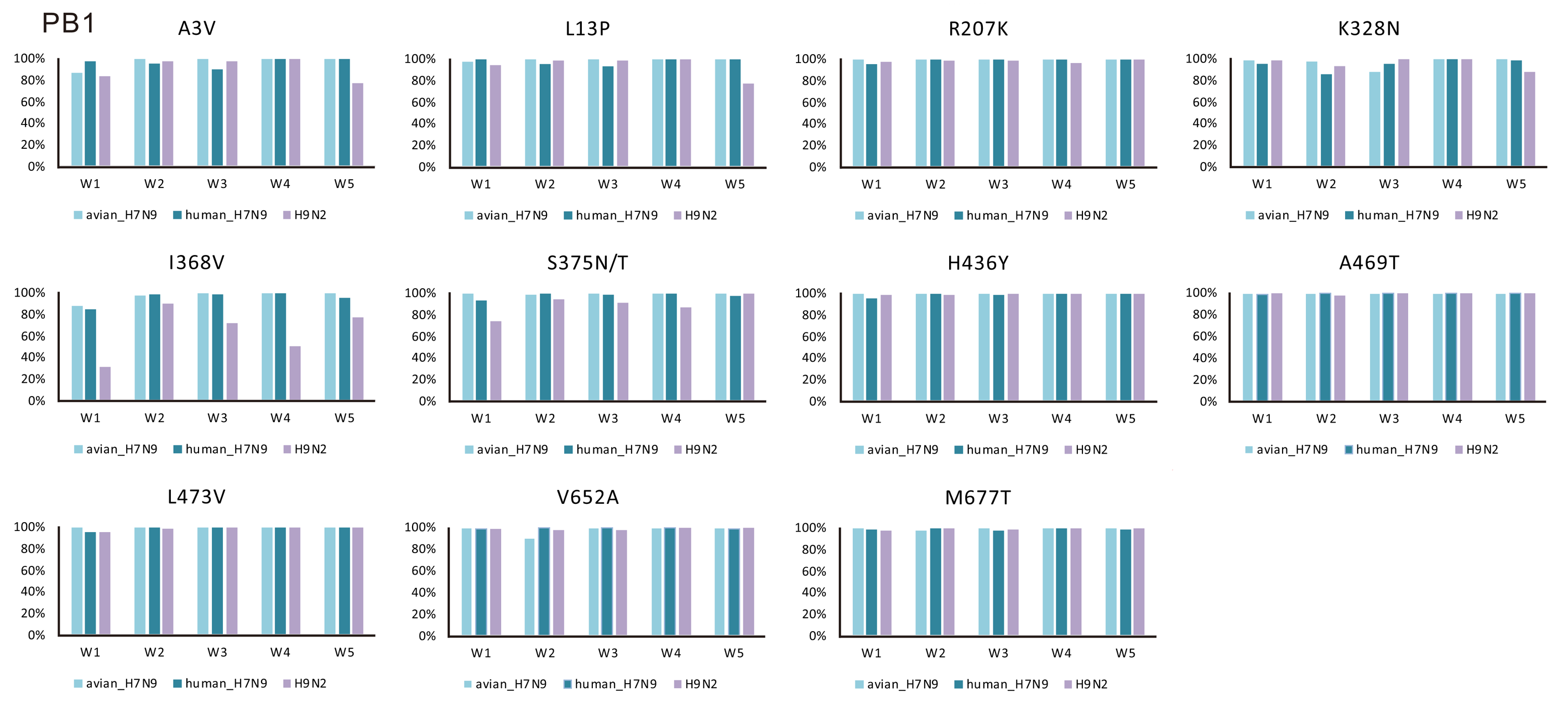

Supplement: Supplementary Figure 1 — Changes in the proportion of 11 amino acid substitutions in PB1 of H7N9 and H9N2 viruses. [file Image_1.TIF]

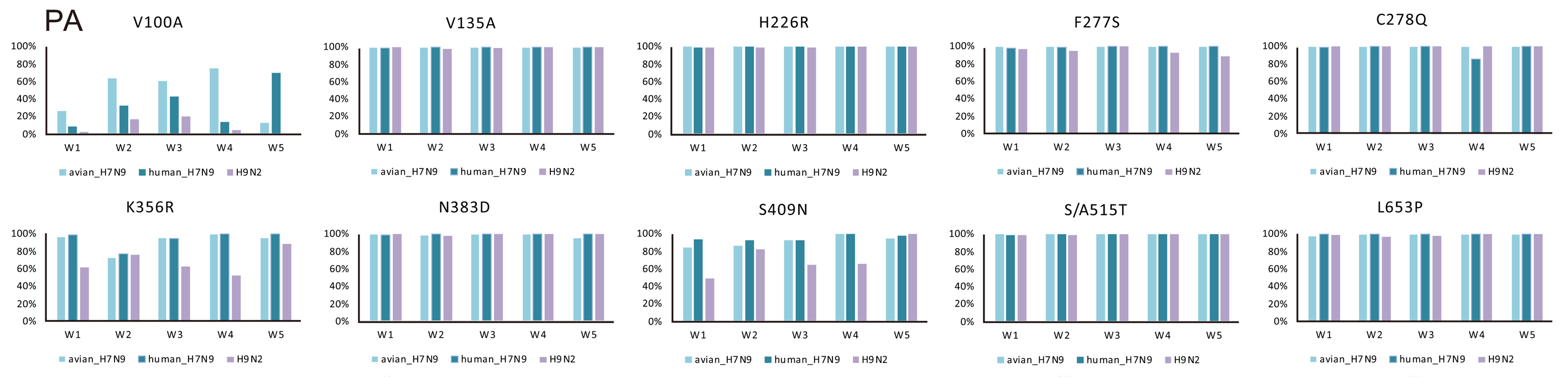

Supplement: Supplementary Figure 2 — Changes in the proportion of 10 amino acid substitutions in PA of H7N9 and H9N2 viruses. [file Image_2.TIF]

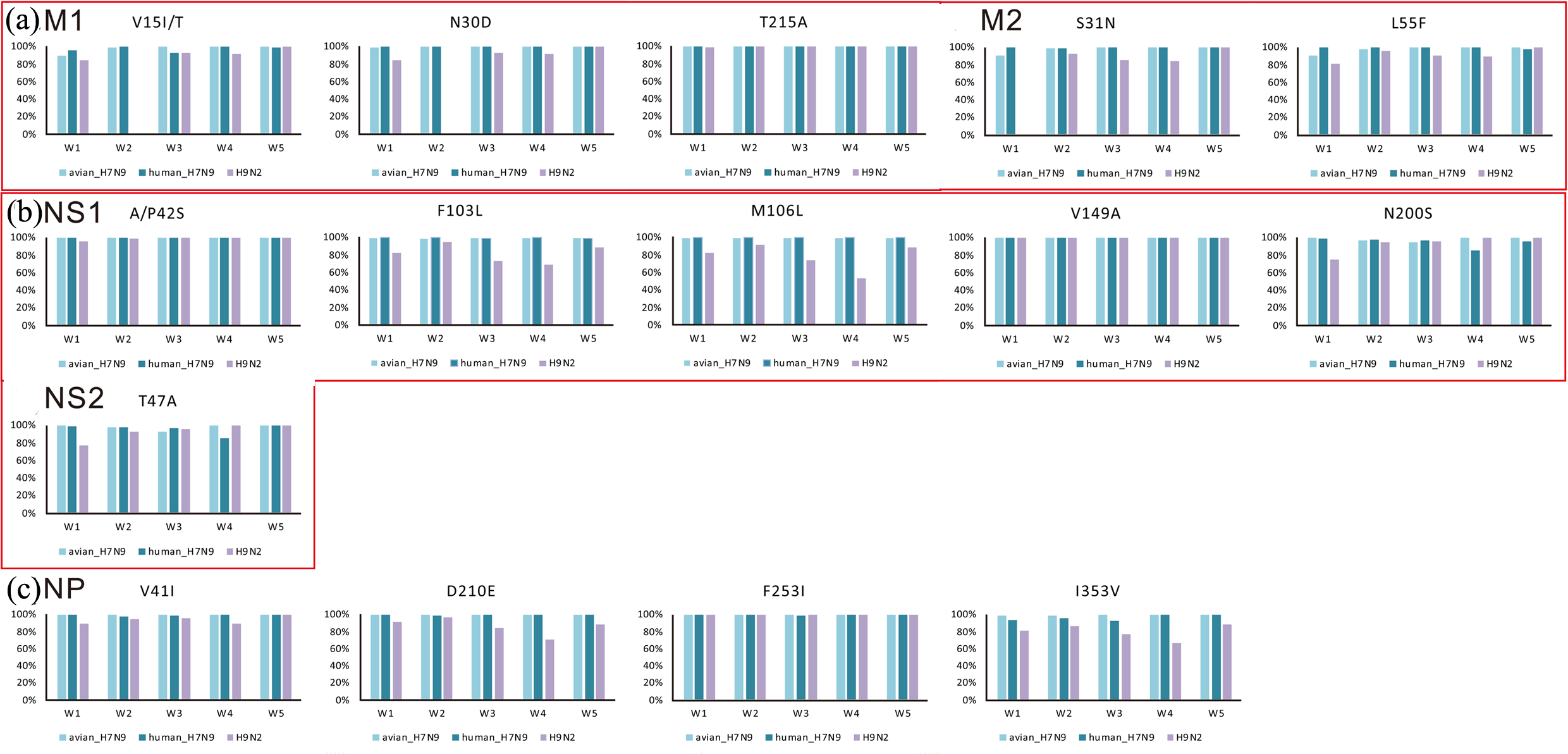

Supplement: Supplementary Figure 3 — Changes in the proportion of five amino acid substitutions in MP, six in NS and four in NP of H7N9 and H9N2 viruses. (a) MP. (b) NS. (c) NP. [file Image_3.TIF]
