# Supplementary material for: Potential Pandemic of H7N9 Avian Influenza A Virus in Human
Source: Front Cell Infect Microbiol. 2018 Nov 23;8:414. doi: 10.3389/fcimb.2018.00414 (PMC6265602; doi:10.3389/fcimb.2018.00414)
Supplement: Supplementary Table 1 — Genetic changes reported to be associated with changes in host tropism or increased pathogenicity in mammals. The H5 numbering system was used to identify nucleotide positions in our analysis. [file Table_1.DOCX]

Supplementary table 1. Genetic changes reported to be associated with changes in host tropism or increased pathogenicity in mammals. The H5 numbering system was used to identify nucleotide positions in our analysis.

| protein | AA substitution (H5 numbering) | phenotype | references |
| --- | --- | --- | --- |
| PB2 | V25A (with PA R443K) | Increased virulence in mice, enhanced the pathogenicity of the virus | [^1^](#_ENREF_1) |
|  | M28I | Increased polymerase activity, increased virulence in mammals and birds | [^2^](#_ENREF_2)^,^[^3^](#_ENREF_3) |
|  | A44S | Human host marker | [^4^](#_ENREF_4) |
|  | T63I (with PB1 M677T) | Pathogenic in mice | [^5^](#_ENREF_5) |
|  | M64T/I | Human host marker | [^4^](#_ENREF_4) |
|  | L89V | Enhanced polymerase activity, Increased virulence in mice | [^6^](#_ENREF_6) |
|  | A199S | Human host marker | [^3^](#_ENREF_3)^,^[^4^](#_ENREF_4)^,^[^7^](#_ENREF_7) |
|  | L207V | Adapt to mammalian hosts | [^8^](#_ENREF_8) |
|  | Q236H (with PB2 E627K; NP N309K) | Increased growth of viruses | [^9^](#_ENREF_9) |
|  | K251R | Increased virulence in mice | [^10^](#_ENREF_10) |
|  | D256G | Enhanced polymerase activity, Mammalian host marker | [^11^](#_ENREF_11) |
|  | T271A | Human host marker (host-specific polymerase activity) | [^3^](#_ENREF_3)^,^[^4^](#_ENREF_4)^,^[^12^](#_ENREF_12) |
|  | A274T | Increased polymerase activity, Increased virulence in mammals and birds | [^2^](#_ENREF_2) |
|  | G309D | Enhanced polymerase activity, Increased virulence in mice | [^6^](#_ENREF_6) |
|  | R318K | Increased virulence in mammals | [^13^](#_ENREF_13)^,^[^14^](#_ENREF_14) |
|  | T339K | Enhanced polymerase activity, Increased virulence in mice | [^6^](#_ENREF_6) |
|  | R/Q355K | Increased virulence in mammals | [^13^](#_ENREF_13)^,^[^15^](#_ENREF_15)^,^[^16^](#_ENREF_16) |
|  | Q368R | Increased polymerase activity, Increased virulence in mammals | [^17^](#_ENREF_17)^,^[^18^](#_ENREF_18) |
|  | E391Q | Increased polymerase activity, Increased virulence in mammals | [^17^](#_ENREF_17)^,^[^18^](#_ENREF_18) |
|  | F404L | Contribute to the increased virulence of the H9N2. | [^19^](#_ENREF_19) |
|  | H447Q | Increased polymerase activity, Increased virulence in mammals | [^17^](#_ENREF_17)^,^[^18^](#_ENREF_18) |
|  | P453H (with PB2 I471T) | Change the surface electrostatic potential drastically | [^20^](#_ENREF_20) |
|  | I471T (with PB2 P453H) | Change the surface electrostatic potential drastically | [^20^](#_ENREF_20) |
|  | L475M | Human host marker (host-specific polymerase activity) | [^4^](#_ENREF_4)^,^[^7^](#_ENREF_7)^,^[^12^](#_ENREF_12) |
|  | R477G | Enhanced polymerase activity, Increased virulence in mice | [^6^](#_ENREF_6) |
|  | I495V | Enhanced polymerase activity, Increased virulence in mice | [^6^](#_ENREF_6) |
|  | K526R | Increased polymerase activity, Increased virulence in mammals and birds (host-specific polymerase activity) | [^2^](#_ENREF_2)^,^[^12^](#_ENREF_12) |
|  | M535L | Increased polymerase activity, Increased virulence in mammals, Mammalian host marker | [^21^](#_ENREF_21) |
|  | I553V | Increased polymerase activity, Increased virulence in mammals and birds | [^2^](#_ENREF_2) |
|  | D567N | Human host marker | [^4^](#_ENREF_4)^,^[^7^](#_ENREF_7) |
|  | A588I/V | Human host marker (host-specific polymerase activity) | [^3^](#_ENREF_3)^,^[^4^](#_ENREF_4)^,^[^12^](#_ENREF_12)^,^[^22^](#_ENREF_22) |
|  | GQ590/591SR/K | Increased polymerase activity, Human host adaptation | [^23^](#_ENREF_23) |
|  | Q591K | Increased virulence in mammals | [^24^](#_ENREF_24) |
|  | L607V | Increased polymerase activity, Increased virulence in mammals and birds | [^2^](#_ENREF_2) |
|  | E/D627K/N | Human host marker, Enhanced polymerase activity, Increased virulence in mammals | [^4^](#_ENREF_4)^,^[^7^](#_ENREF_7)^,^[^11^](#_ENREF_11)^,^[^13^](#_ENREF_13)^,^[^15^](#_ENREF_15)^,^[^24-51^](#_ENREF_24) |
|  | A661T | Enhanced Transmission | [^3^](#_ENREF_3) |
|  | V667I | Enhanced Transmission | http://www.fludb.org |
|  | A/S674T | Human host marker | [^3^](#_ENREF_3)^,^[^4^](#_ENREF_4) |
|  | A676T | Enhanced polymerase active, Increased virulence in mice | [^6^](#_ENREF_6) |
|  | K699R | Enhanced virulence in mice | [^52^](#_ENREF_52) |
|  | D701N | Increased polymerase activity, Increased virulence in mammals, Mammalian host marker | [^12^](#_ENREF_12)^,^[^23-25^](#_ENREF_23)^,^[^37^](#_ENREF_37)^,^[^50^](#_ENREF_50)^,^[^53-58^](#_ENREF_53) |
|  | K702R | Human host marker | [^3^](#_ENREF_3)^,^[^4^](#_ENREF_4)^,^[^7^](#_ENREF_7) |
|  | S714R | Increased polymerase activity, Increased virulence in mammals, Mammalian host marker | [^55^](#_ENREF_55)^,^[^58^](#_ENREF_58) |
|  | | | |
| PB1 | D/A3V | Increased polymerase activity, Increased virulence in mammals | [^17^](#_ENREF_17)^,^[^18^](#_ENREF_18)^,^[^59^](#_ENREF_59) |
|  | L13P | Increased polymerase activity, Increased virulence in mammals, Mammalian host marker | [^55^](#_ENREF_55)^,^[^58^](#_ENREF_58) |
|  | M51T | Increased virulence in ducks | [^60^](#_ENREF_60) |
|  | A56V | Increased virulence in ducks | [^60^](#_ENREF_60) |
|  | G87E | Increased virulence in ducks | [^60^](#_ENREF_60) |
|  | H99Y | Airborne transmissible in mammals | [^26^](#_ENREF_26) |
|  | R118I (with PB2 E627K; PA L550M; HA G201R; NA S373N) | Increased virulence in mammals | [^61^](#_ENREF_61) |
|  | R207K | Increased polymerase activity in mammalian cells | [^62^](#_ENREF_62) |
|  | T296R | Increased polymerase activity , Increased virulence in mice | [^63^](#_ENREF_63) |
|  | M/V317I | Increased virulence in mammals | [^14-16^](#_ENREF_14)^,^[^64^](#_ENREF_64) |
|  | K328N | Increased polymerase activity, Increased virulence in mammals | [^17^](#_ENREF_17)^,^[^18^](#_ENREF_18) |
|  | I368V | Airborne transmissible in mammals | [^26^](#_ENREF_26) |
|  | S375N/T | Increased polymerase activity, Increased virulence in mammals, Human host marker | [^7^](#_ENREF_7)^,^[^17^](#_ENREF_17)^,^[^18^](#_ENREF_18) |
|  | H436Y | Increased polymerase activity and virulence in mallards, ferrets and mice | [^62^](#_ENREF_62) |
|  | A469T (with NS1 N205K; NEP T48N) | Conferred in contact transmissibility in guinea pigs. | [^65^](#_ENREF_65) |
|  | L473V | Increased polymerase activity and replication efficiency | [^66^](#_ENREF_66) |
|  | L598P | Increased polymerase activity and replication efficiency | [^66^](#_ENREF_66) |
|  | V652A | Increased virulence in mice | [^10^](#_ENREF_10) |
|  | M677T (with PB2 T63I) | Pathogenic in mice | [^5^](#_ENREF_5) |
|  | S678N | Increased polymerase activity, Increased virulence in mammals, Mammalian host marker | [^55^](#_ENREF_55)^,^[^58^](#_ENREF_58) |
|  | | | |
| PB1-F2 | N66S | Increased virulence in mammals | [^67-69^](#_ENREF_67) |
|  | | | |
| PA | D3V | Contribute to the increased virulence of the H9N2 | [^19^](#_ENREF_19) |
|  | P26L | Human host marker | [^4^](#_ENREF_4) |
|  | E31K | Increase viral yields and HA contents of different subtypes of avian influenza CVVs | [^70^](#_ENREF_70) |
|  | S37A | Significantly increased viral growth and polymerase activity in mammalian cells | [^71^](#_ENREF_71) |
|  | I38M (with HA H153N, S243R) | Increased pathogenicity in mice | [^72^](#_ENREF_72) |
|  | V44I | Enhance the replicative ability of an H5N1 virus in A549 cells and enhance its pathogenicity in mice | [^73^](#_ENREF_73) |
|  | D55N | Human host marker | [^3^](#_ENREF_3)^,^[^4^](#_ENREF_4)^,^[^7^](#_ENREF_7) |
|  | R57Q | Human host marker | [^4^](#_ENREF_4) |
|  | A70V (with PA S224P) | Reduced the virus's 50% mouse lethal dose | [^74^](#_ENREF_74) |
|  | T97I | Enhanced polymerase activity, Increased virulence in mice | [^75-79^](#_ENREF_75) |
|  | V100A | Human host marker | [^3^](#_ENREF_3)^,^[^4^](#_ENREF_4)^,^[^7^](#_ENREF_7) |
|  | P103H (with S659L) | Reduce polymerase activity and viral replication in mammalian cells and attenuate pathogenicity in mice | [^80^](#_ENREF_80) |
|  | V127I/A | Increased virulence in mammals | [^14^](#_ENREF_14)^,^[^73^](#_ENREF_73)^,^[^81^](#_ENREF_81) |
|  | S149P | Increased polymerase activity, Increased virulence in mammals and birds | [^2^](#_ENREF_2) |
|  | K158R | Increased polymerase activity | [^59^](#_ENREF_59) |
|  | S224P (with N383D) | Enhanced the pathogenicity and viral replication of H5N1 virus in mice | [^74^](#_ENREF_74)^,^[^82^](#_ENREF_82)^,^[^83^](#_ENREF_83) |
|  | S225C/R | Human host marker | [^4^](#_ENREF_4)^,^[^19^](#_ENREF_19) |
|  | C241Y | Enhance the replicative ability of an H5N1 virus in A549 cells and enhance its pathogenicity in mice | [^73^](#_ENREF_73) |
|  | H266R | Increased polymerase activity, Increased virulence in mammals and birds | [^2^](#_ENREF_2) |
|  | L268I | Human host marker | [^4^](#_ENREF_4) |
|  | F277S | Adapt to mammalian hosts | [^8^](#_ENREF_8) |
|  | C278Q | Adapt to mammalian hosts | [^8^](#_ENREF_8) |
|  | N321K | Increased polymerase activity | [^84^](#_ENREF_84) |
|  | K328R | Increased virulence in mice | [^78^](#_ENREF_78) |
|  | P332T | Increased virulence in mice | [^78^](#_ENREF_78) |
|  | L336M | Increased virulence in mammals | [^14^](#_ENREF_14)^,^[^81^](#_ENREF_81) |
|  | A337S | Human host marker | [^4^](#_ENREF_4) |
|  | A343T | Increased virulence in mammals and mice | [^73^](#_ENREF_73) |
|  | K356R | Increased virulence in mammals and mice | [^85^](#_ENREF_85) |
|  | I357K | Increased polymerase activity, Increased virulence in mammals and birds | [^2^](#_ENREF_2) |
|  | E382D | Human host marker | [^3^](#_ENREF_3)^,^[^7^](#_ENREF_7) |
|  | N383D (with S224P) | Enhanced the pathogenicity and viral replication of H5N1 virus in mice | [^82^](#_ENREF_82)^,^[^83^](#_ENREF_83) |
|  | A404S | Human host marker | [^4^](#_ENREF_4) |
|  | S409N | Enhanced Transmission, Human host marker | [^3^](#_ENREF_3)^,^[^4^](#_ENREF_4) |
|  | R443K (with PB2 V25A) | Increased virulence in mice, enhanced the pathogenicity of the virus | [^1^](#_ENREF_1) |
|  | S/A515T | Increased polymerase activity, Increased virulence in mammals and birds | [^2^](#_ENREF_2)^,^[^62^](#_ENREF_62) |
|  | L550M (with PB2 E627K; PB1 R118I; HA G201R; NA S373N) | Increased virulence in mammals | [^61^](#_ENREF_61) |
|  | T552S | Human host marker | [^3^](#_ENREF_3)^,^[^4^](#_ENREF_4)^,^[^7^](#_ENREF_7) |
|  | Q556R | Increased virulence in mice | [^78^](#_ENREF_78) |
|  | I581V | Enhance the replicative ability of an H5N1 virus in A549 cells and enhance its pathogenicity in mice | [^73^](#_ENREF_73) |
|  | F611S | Adapt to mammalian hosts | [^8^](#_ENREF_8) |
|  | K615N | Increased polymerase activity, Increased virulence in mammals, Mammalian host marker | [^55^](#_ENREF_55)^,^[^58^](#_ENREF_58) |
|  | L653P | Adapt to mammalian hosts | [^8^](#_ENREF_8) |
|  | S659L (with P103H) | Reduce polymerase activity and viral replication in mammalian cells and attenuate pathogenicity in mice | [^80^](#_ENREF_80) |
|  | | | |
| HA | E57K (with S123P, N193K, R497K) | Increased virus binding to 2,6 | [^86^](#_ENREF_86) |
|  | G74R | May serve as molecular markers for H9N2 virus evolution, and may aid improving AIV vaccine effectiveness | [^87^](#_ENREF_87) |
|  | A/I/P/S/T86V | Increased virulence in mammals | [^14^](#_ENREF_14)^,^[^88^](#_ENREF_88) |
|  | D94N | Increased virus binding to 2,6, Enhanced virus fusion | [^89^](#_ENREF_89) |
|  | A100T | Increased virulence in mice | [^78^](#_ENREF_78) |
|  | H103Y (with T156A, Q222L, G224S) | Airborne transmissible in mammals | [^26^](#_ENREF_26) |
|  | L104F | Increased pathogenicity in mice | [^72^](#_ENREF_72) |
|  | E107K | Increased virulence in mice | [^90^](#_ENREF_90) |
|  | T108I | Increasing viral replicative ability | [^91^](#_ENREF_91) |
|  | I116M | Potential to alter the virulence of H1N1pdm09 in swine | [^92^](#_ENREF_92) |
|  | K118N | Achieve high egg-growth to for rapid generation of vaccine production candidates | [^93^](#_ENREF_93) |
|  | S121N | Increased virus binding to 2,6, Increased replication in mammals | [^94^](#_ENREF_94) |
|  | S123P (with E75K, N193K, R497K) | Increased virus binding to 2,6 | [^86^](#_ENREF_86)^,^[^95^](#_ENREF_95) |
|  | N/D124S | Increased virulence in mammals | [^14^](#_ENREF_14)^,^[^88^](#_ENREF_88) |
|  | L129V/del (with A134V, I151T) | Increased virus binding to 2,6 | [^96^](#_ENREF_96)^,^[^97^](#_ENREF_97) |
|  | T131A | Improve immunogenicity of H7N7HA vaccine against H7N7/NL/219/03 virus | [^98^](#_ENREF_98) |
|  | S133A | Increased pseudovirus binding to 2,6 | [^99^](#_ENREF_99) |
|  | A134V | Increased virus binding to 2,6 | [^96^](#_ENREF_96)^,^[^100-103^](#_ENREF_100) |
|  | Q/H/I138L/N | Increased virulence in mammals | [^14^](#_ENREF_14)^,^[^88^](#_ENREF_88) |
|  | G139R | Increased virus binding to 2,6 | [^86^](#_ENREF_86)^,^[^104^](#_ENREF_104) |
|  | S142G | Potential to alter the virulence of H1N1pdm09 in swine | [^92^](#_ENREF_92) |
|  | I151T (with L129V/del) | Increased virus binding to 2,6 | [^96^](#_ENREF_96)^,^[^97^](#_ENREF_97) |
|  | H153N (with HA S243R; PA I38M) | Increased pathogenicity in mice | [^72^](#_ENREF_72) |
|  | N154D (with N220K, Q222L, N224D, T315I) | Airborne transmissibility in mammals | [^105^](#_ENREF_105)^,^[^106^](#_ENREF_106) |
|  | S155N | Increased virus binding to 2,6, Increased replication in mammals | [^94^](#_ENREF_94)^,^[^107^](#_ENREF_107) |
|  | T156A | Increased virus binding to 2,6, Airborne transmissible in mammals | [^26^](#_ENREF_26)^,^[^53^](#_ENREF_53)^,^[^94^](#_ENREF_94)^,^[^107^](#_ENREF_107) |
|  | G/L/S/N182K/D/V/P | Increased virus binding to 2,6 | [^86^](#_ENREF_86)^,^[^101^](#_ENREF_101)^,^[^104^](#_ENREF_104) |
|  | E183G | Increased virus binding to 2,6 | [^108^](#_ENREF_108)^,^[^109^](#_ENREF_109) |
|  | S184N (with D221N) | Increased virulence in mammals | [^110^](#_ENREF_110) |
|  | T/E186G/D/V | Increased virus binding to 2,6 | [^108^](#_ENREF_108)^,^[^109^](#_ENREF_109)^,^[^111^](#_ENREF_111)^,^[^112^](#_ENREF_112) |
|  | T188I | Increased pseudovirus binding to 2,6 | [^99^](#_ENREF_99) |
|  | N/K189S/R/D/E | Increased virus binding to 2,6, Increased replication in mammals | [^94^](#_ENREF_94)^,^[^109^](#_ENREF_109) [^19^](#_ENREF_19)^,^[^113^](#_ENREF_113) |
|  | Q192R/H | Increased virus binding to 2,6 | [^86^](#_ENREF_86)^,^[^97^](#_ENREF_97)^,^[^104^](#_ENREF_104)^,^[^108^](#_ENREF_108) |
|  | N193K | Increased virus binding to 2,6 | [^86^](#_ENREF_86) |
|  | I198V | Improve immunogenicity of H7N7HA vaccine against H7N7/NL/219/03 virus | [^98^](#_ENREF_98) |
|  | G201E/R (with PB2 E627K; PB1 R118I; PA L550M; NA S373N) | Increased virulence in mammals | [^61^](#_ENREF_61)^,^[^90^](#_ENREF_90) |
|  | S205Y | Increased virus binding to 2,6 | [^114^](#_ENREF_114) |
|  | V210I | Increased virus binding to 2,6 | [^97^](#_ENREF_97) |
|  | K212E/R/G | Increased virulence in mammals | [^14^](#_ENREF_14)^,^[^88^](#_ENREF_88) |
|  | G214E | Increased virulence in mice | [^90^](#_ENREF_90) |
|  | R216I | Increased virulence in mice | [^78^](#_ENREF_78) |
|  | K218E | Altered pathogenicity and tissue tropism in mice | [^99^](#_ENREF_99)^,^[^115^](#_ENREF_115) |
|  | N220K (with N154D, Q222L, T315I) | Airborne transmissible in mammals | [^106^](#_ENREF_106)^,^[^116^](#_ENREF_116) |
|  | D221G/N | Change in receptor binding affinity from human to avian receptors | [^93^](#_ENREF_93)^,^[^110^](#_ENREF_110)^,^[^113^](#_ENREF_113)^,^[^116-121^](#_ENREF_116) |
|  | Q222L/R | Increased virus binding to 2,6, Airborne transmissible in mammals | [^26^](#_ENREF_26)^,^[^78^](#_ENREF_78)^,^[^93^](#_ENREF_93)^,^[^105^](#_ENREF_105)^,^[^106^](#_ENREF_106)^,^[^120-124^](#_ENREF_120) |
|  | Q/S223N/R/P | Increased virus binding to 2,6 | [^94^](#_ENREF_94)^,^[^101^](#_ENREF_101)^,^[^104^](#_ENREF_104)^,^[^107-109^](#_ENREF_107)^,^[^125-128^](#_ENREF_125) |
|  | G224S | Increased virus binding to 2,6, Airborne transmissible in mammals | [^26^](#_ENREF_26)^,^[^94^](#_ENREF_94)^,^[^104^](#_ENREF_104)^,^[^107-109^](#_ENREF_107)^,^[^118^](#_ENREF_118)^,^[^123^](#_ENREF_123) |
|  | P235S | Increased virus binding to 2,6 | [^97^](#_ENREF_97) |
|  | S243R (with HA H153N; PA I38M) | Increased pathogenicity in mice | [^72^](#_ENREF_72) |
|  | V248M | Potential to alter the virulence of H1N1pdm09 in swine | [^92^](#_ENREF_92) |
|  | E251K | Increased virus binding to 2,6 | [^108^](#_ENREF_108) |
|  | A263T | Increased virulence in mammals | [^14^](#_ENREF_14)^,^[^88^](#_ENREF_88) |
|  | N301D | Decreased the virulence of mouse-adapted virus | [^129^](#_ENREF_129) |
|  | T315I (with N154D, N220K, Q222L) | Airborne transmissible in mammals | [^106^](#_ENREF_106) |
|  | R351K | Increased virulence in mice | [^78^](#_ENREF_78) |
|  | Q368K | Reduced viral fitness | [^130^](#_ENREF_130) |
|  | D372Y/G/E/N | Enhanced receptor binding to α2,3-linked sialic acid receptors | [^128^](#_ENREF_128)^,^[^130^](#_ENREF_130)^,^[^131^](#_ENREF_131) |
|  | N376T | Increased virulence in mammals | [^79^](#_ENREF_79) |
|  | K3881L | Decreased pH of fusion, Increased HA stability, Increased replication efficiency in mice | [^132^](#_ENREF_132)^,^[^133^](#_ENREF_133) |
|  | G395E | Enhanced polymerase activity, Increased virulence in mice | [^75^](#_ENREF_75) |
|  | F427L | Important for adaptation of H5N5 AIVs to mammals | [^134^](#_ENREF_134) |
|  | E435K | Decreased pH of fusion | [^133^](#_ENREF_133) |
|  | N444K | Increased pH of fusion | [^133^](#_ENREF_133) |
|  | N484S | Decreased the virulence of mouse-adapted virus | [^129^](#_ENREF_129) |
|  | R497K (with E75K, S123P, N193K) | Increased virus binding to 2,6 | [^86^](#_ENREF_86) |
|  | | | |
| NP | G16D | Human host marker | [^4^](#_ENREF_4) |
|  | V41I | Might contribute to viral transmissibility | [^135^](#_ENREF_135) |
|  | S50G (with PB2 Q591K) | Increased growth of viruses | [^9^](#_ENREF_9) |
|  | I61L | Human host marker | [^3^](#_ENREF_3)^,^[^4^](#_ENREF_4) |
|  | K91R | Significantly decreased the polymerase activity | [^136^](#_ENREF_136) |
|  | R99K | Airborne transmissible in mammals | [^26^](#_ENREF_26) |
|  | V105M | Contribute to the increased virulence of the H9N2 | [^19^](#_ENREF_19) |
|  | L136M | Enhanced Transmission | [^3^](#_ENREF_3) |
|  | K198R | Significantly decreased the polymerase activity | [^136^](#_ENREF_136) |
|  | D210E | Might contribute to viral transmissibility | [^135^](#_ENREF_135) |
|  | F253I | Results in attenuated pathogenicity of the virus in mice | [^137^](#_ENREF_137) |
|  | L283P | Human host marker | [^3^](#_ENREF_3)^,^[^4^](#_ENREF_4) |
|  | A284T | Increased virulence in mice | [^78^](#_ENREF_78) |
|  | R305K | Human host marker | [^4^](#_ENREF_4) |
|  | N309K (with PB2 Q236H, E627K) | Increased growth of viruses | [^9^](#_ENREF_9) |
|  | F313Y | Human host marker | [^3^](#_ENREF_3)^,^[^4^](#_ENREF_4) |
|  | N319K | Increased polymerase activity, Mammalian host marker | [^55^](#_ENREF_55)^,^[^56^](#_ENREF_56) |
|  | V343L | Being highly BNP-sensitive to moderately BNP-resistant | [^138^](#_ENREF_138) |
|  | S345N | Airborne transmissible in mammals | [^26^](#_ENREF_26) |
|  | M352I | Increased virulence in mice | [^78^](#_ENREF_78) |
|  | I353V | Increased virulence in mice | [^10^](#_ENREF_10) |
|  | Q357L (with PB2 I27K) | Increased virulence in mammals, Human host marker | [^4^](#_ENREF_4)^,^[^56^](#_ENREF_56) |
|  | E434K | Enhanced viral RNA polymerase activity in vitro | [^128^](#_ENREF_128) |
|  | K470R | Significantly increased its virulence in vitro and in vivo | [^136^](#_ENREF_136) |
|  | | | |
| NA | M26I | Increased virulence in mice | [^78^](#_ENREF_78) |
|  | A27T | Contribute to the increased virulence of the H9N2 | [^19^](#_ENREF_19) |
|  | I106V | Increased virulence in mice | [^10^](#_ENREF_10)^,^[^74^](#_ENREF_74) |
|  | E119K/D | Increased virulence in mammals | [^139^](#_ENREF_139)^,^[^140^](#_ENREF_140) |
|  | Q136K | Causing resistance to or reduced inhibition by NAIs (oseltamivir, zanamivir, and peramivir) | [^139^](#_ENREF_139)^,^[^141^](#_ENREF_141)^,^[^142^](#_ENREF_142) |
|  | R143K | Increased virulence in mammals and mice | [^103^](#_ENREF_103) |
|  | N146S | Increased virulence in mice | [^78^](#_ENREF_78) |
|  | G147E | Increased virulence in mammals and mice | [^103^](#_ENREF_103) |
|  | D151G | Interferes with drug susceptibility assessment | [^143^](#_ENREF_143) |
|  | T223I | Increased virulence in mammals | [^13^](#_ENREF_13)^,^[^15^](#_ENREF_15)^,^[^16^](#_ENREF_16) |
|  | V241I | Contributed to the significantly higher baseline IC50 value obtained to oseltamivir for 2010/2011 viruses | [^144^](#_ENREF_144) |
|  | N248D | Increased virulence in mice | [^10^](#_ENREF_10) |
|  | H275Y | Increased virulence in mammals | [^145^](#_ENREF_145) |
|  | R293K | Highly resistant to the oseltamivir | [^146^](#_ENREF_146)^,^[^147^](#_ENREF_147) |
|  | S354N | Increased virulence in mice | [^90^](#_ENREF_90) |
|  | K/S373A/N (with PB2 E627K; PB1 R118I; PA L550M; HA G201R) | Increased virulence in mammals | [^61^](#_ENREF_61)^,^[^144^](#_ENREF_144)^,^[^148^](#_ENREF_148) |
|  | N390K | Viral growth was reduced | [^149^](#_ENREF_149)^,^[^150^](#_ENREF_150) |
|  | W403R | Human host marker | [^148^](#_ENREF_148) |
|  | G394D | Increased virulence in mice | [^78^](#_ENREF_78) |
|  | I429T | Alters NAI susceptibility | [^151^](#_ENREF_151) |
|  | P433S (with H275Y) | Viral growth was reduced | [^149^](#_ENREF_149) |
|  | | | |
| M1 | V15I/T | Increased virulence in mammals | [^13^](#_ENREF_13)^,^[^15^](#_ENREF_15)^,^[^16^](#_ENREF_16) |
|  | N30D | Increased virulence in mammals | [^152^](#_ENREF_152) |
|  | V115I | Human host marker | [^4^](#_ENREF_4) |
|  | T121A | Human host marker | [^4^](#_ENREF_4) |
|  | M128R | Increased virulence in mice | [^78^](#_ENREF_78) |
|  | T137A | Human host marker | [^3^](#_ENREF_3)^,^[^4^](#_ENREF_4) |
|  | A166V | Contribute to the increased virulence of the H9N2. | [^19^](#_ENREF_19) |
|  | S183A | Resulted in the failure of virus production | [^153^](#_ENREF_153) |
|  | T185A | Resulted in the failure of virus production | [^153^](#_ENREF_153) |
|  | T215A | Increased virulence in mammals | [^152^](#_ENREF_152) |
|  | | | |
| M2 | E16G | Enhanced Transmission | [^3^](#_ENREF_3) |
|  | L55F | Enhanced Transmission | [^3^](#_ENREF_3) |
|  | | | |
| NS1 | M31I | Increased virulence in mammals | [^81^](#_ENREF_81) |
|  | A/P42S | Increased virulence in mammals, Antagonism of IFNinduction | [^14^](#_ENREF_14)^,^[^154^](#_ENREF_154) |
|  | G45R | Increased virulence in mammals | [^155^](#_ENREF_155) |
|  | I64T | Increased innate immune responses after viral infection, augmented IFN sensitivity, and virus attenuation in mice | [^156^](#_ENREF_156) |
|  | D74N | Increased virulence in mammals | [^157^](#_ENREF_157) |
|  | T80E | Reduced influenza virus replication through controlling RIG-I-mediated IFN production and vRNP activity | [^158^](#_ENREF_158) |
|  | I81M | Human host marker | [^4^](#_ENREF_4) |
|  | D87E | Increased virulence in mammals | [^159^](#_ENREF_159)^,^[^160^](#_ENREF_160) |
|  | Y89F | Limiting an interferon (IFN) response to infection | [^161^](#_ENREF_161) |
|  | T/D92E | Increased virulence in mammals, Escape of antiviral host response | [^14^](#_ENREF_14)^,^[^15^](#_ENREF_15)^,^[^160^](#_ENREF_160)^,^[^162^](#_ENREF_162)^,^[^163^](#_ENREF_163) |
|  | L98F | Increased virulence in mammals | [^164^](#_ENREF_164)^,^[^165^](#_ENREF_165) |
|  | I101M | Increased virulence in mammals | [^164^](#_ENREF_164)^,^[^165^](#_ENREF_165) |
|  | F103L (with M106L) | Diminished ability to regulate type I IFN responses | [^166^](#_ENREF_166)^,^[^167^](#_ENREF_167) |
|  | M106L (with F103L) | Diminished ability to regulate type I IFN responses | [^166^](#_ENREF_166)^,^[^167^](#_ENREF_167) |
|  | T/D/V/R/A127N | Increased virulence in mammals | [^14^](#_ENREF_14)^,^[^168^](#_ENREF_168) |
|  | V149A | Pathogenicity in mice, Antagonism of IFN induction | [^169^](#_ENREF_169) |
|  | D/G189N | Increased virulence in mammals | [^14^](#_ENREF_14)^,^[^81^](#_ENREF_81) |
|  | V194I | Attenuated in a mouse model of influenza infection | [^170^](#_ENREF_170) |
|  | S195T/Y | Increased virulence in mammals | [^14^](#_ENREF_14)^,^[^81^](#_ENREF_81)^,^[^171^](#_ENREF_171) |
|  | N200S (with NEP/NS2 T47A) | Decreased IFN antagonism | [^100^](#_ENREF_100) |
|  | N/G205R/K (with NEP/NS2 M51I, T48N; PB2 A469T) | Decreased IFN antagonism, Conferred enhanced in-contact transmissibility in guinea pigs | [^65^](#_ENREF_65)^,^[^100^](#_ENREF_100) |
|  | P215T | Human host marker | [^4^](#_ENREF_4) |
|  | E227R | Human host marker | [^4^](#_ENREF_4) |
|  | S228P | Increased virulence in mammals | [^14^](#_ENREF_14) |
|  | | | |
| NEP/NS2 | M31I | Increased virulence in mammals | [^14^](#_ENREF_14)^,^[^81^](#_ENREF_81) |
|  | T47A (with NS1 N200S) | Decreased IFN antagonism | [^100^](#_ENREF_100) |
|  | T48N (with PB1 A469T, NS1 N205K) | Conferred enhanced in-contact transmissibility in guinea pigs | [^65^](#_ENREF_65) |
|  | M51I (with NS1 G205R) | Decreased IFN antagonism | [^100^](#_ENREF_100) |
|  | H/L56Y | Increased virulence in mammals | [^14^](#_ENREF_14)^,^[^81^](#_ENREF_81) |

**References:**

1 Zhao, D. *et al.* Molecular Determinants of Virulence and Stability of a Reporter-Expressing H5N1 Influenza A Virus. *J Virol* **89**, 11337-11346 (2015).

2 Leung, B. W., Chen, H. & Brownlee, G. G. Correlation between polymerase activity and pathogenicity in two duck H5N1 influenza viruses suggests that the polymerase contributes to pathogenicity. *Virology* **401**, 96-106 (2010).

3 Shaw, M. *et al.* Molecular changes associated with the transmission of avian influenza a H5N1 and H9N2 viruses to humans. *J Med Virol* **66**, 107-114 (2002).

4 Finkelstein, D. B. *et al.* Persistent host markers in pandemic and H5N1 influenza viruses. *J Virol* **81**, 10292-10299 (2007).

5 Li, J. *et al.* PB1-mediated virulence attenuation of H5N1 influenza virus in mice is associated with PB2. *J Gen Virol* **92**, 1435-1444 (2011).

6 Li, J. *et al.* Single mutation at the amino acid position 627 of PB2 that leads to increased virulence of an H5N1 avian influenza virus during adaptation in mice can be compensated by multiple mutations at other sites of PB2. *Virus Res* **144**, 123-129 (2009).

7 Taubenberger, J. K. *et al.* Characterization of the 1918 influenza virus polymerase genes. *Nature* **437**, 889-893 (2005).

8 Mei, K. *et al.* Deep sequencing reveals the viral adaptation process of environment-derived H10N8 in mice. *Infect Genet Evol* **37**, 8-13 (2016).

9 Danzy, S. *et al.* Mutations to PB2 and NP proteins of an avian influenza virus combine to confer efficient growth in primary human respiratory cells. *J Virol* **88**, 13436-13446 (2014).

10 Prokopyeva, E. A., Sobolev, I. A., Prokopyev, M. V. & Shestopalov, A. M. Adaptation of influenza A(H1N1)pdm09 virus in experimental mouse models. *Infect Genet Evol* **39**, 265-271 (2016).

11 Manzoor, R. *et al.* PB2 protein of a highly pathogenic avian influenza virus strain A/chicken/Yamaguchi/7/2004 (H5N1) determines its replication potential in pigs. *J Virol* **83**, 1572-1578 (2009).

12 Foeglein, A. *et al.* Influence of PB2 host-range determinants on the intranuclear mobility of the influenza A virus polymerase. *J Gen Virol* **92**, 1650-1661 (2011).

13 Chen, H. *et al.* Polygenic virulence factors involved in pathogenesis of 1997 Hong Kong H5N1 influenza viruses in mice. *Virus Res* **128**, 159-163 (2007).

14 Lycett, S. J. *et al.* Detection of mammalian virulence determinants in highly pathogenic avian influenza H5N1 viruses: multivariate analysis of published data. *J Virol* **83**, 9901-9910 (2009).

15 Lee, M. S. *et al.* Characterization of an H5N1 avian influenza virus from Taiwan. *Vet Microbiol* **124**, 193-201 (2007).

16 Katz, J. M. *et al.* Molecular correlates of influenza A H5N1 virus pathogenesis in mice. *J Virol* **74**, 10807-10810 (2000).

17 Salomon, R. *et al.* The polymerase complex genes contribute to the high virulence of the human H5N1 influenza virus isolate A/Vietnam/1203/04. *J Exp Med* **203**, 689-697 (2006).

18 Govorkova, E. A. *et al.* Lethality to ferrets of H5N1 influenza viruses isolated from humans and poultry in 2004. *J Virol* **79**, 2191-2198 (2005).

19 Liu, Q. *et al.* A nonpathogenic duck-origin H9N2 influenza A virus adapts to high pathogenicity in mice. *Arch Virol* **159**, 2243-2252 (2014).

20 Tsurumura, T., Qiu, H., Yoshida, T., Tsumori, Y. & Tsuge, H. Crystallization and preliminary X-ray diffraction studies of a surface mutant of the middle domain of PB2 from human influenza A (H1N1) virus. *Acta Crystallogr F Struct Biol Commun* **70**, 72-75 (2014).

21 Chen, G. W. *et al.* Genomic Signatures for Avian H7N9 Viruses Adapting to Humans. *PLoS One* **11**, e0148432 (2016).

22 Xiao, C. *et al.* PB2-588 V promotes the mammalian adaptation of H10N8, H7N9 and H9N2 avian influenza viruses. *Sci Rep* **6**, 19474 (2016).

23 Mehle, A. & Doudna, J. A. Adaptive strategies of the influenza virus polymerase for replication in humans. *Proc Natl Acad Sci U S A* **106**, 21312-21316 (2009).

24 Yamada, S. *et al.* Biological and structural characterization of a host-adapting amino acid in influenza virus. *PLoS Pathog* **6**, e1001034 (2010).

25 Steel, J., Lowen, A. C., Mubareka, S. & Palese, P. Transmission of influenza virus in a mammalian host is increased by PB2 amino acids 627K or 627E/701N. *PLoS Pathog* **5**, e1000252 (2009).

26 Herfst, S. *et al.* Airborne transmission of influenza A/H5N1 virus between ferrets. *Science* **336**, 1534-1541 (2012).

27 Hatta, M. *et al.* Growth of H5N1 influenza A viruses in the upper respiratory tracts of mice. *PLoS Pathog* **3**, 1374-1379 (2007).

28 Massin, P., van der Werf, S. & Naffakh, N. Residue 627 of PB2 is a determinant of cold sensitivity in RNA replication of avian influenza viruses. *J Virol* **75**, 5398-5404 (2001).

29 Van Hoeven, N. *et al.* Human HA and polymerase subunit PB2 proteins confer transmission of an avian influenza virus through the air. *Proc Natl Acad Sci U S A* **106**, 3366-3371 (2009).

30 Kawaoka, Y. & Webster, R. G. Sequence requirements for cleavage activation of influenza virus hemagglutinin expressed in mammalian cells. *Proc Natl Acad Sci U S A* **85**, 324-328 (1988).

31 Subbarao, E. K., London, W. & Murphy, B. R. A single amino acid in the PB2 gene of influenza A virus is a determinant of host range. *J Virol* **67**, 1761-1764 (1993).

32 Chen, H. *et al.* The evolution of H5N1 influenza viruses in ducks in southern China. *Proc Natl Acad Sci U S A* **101**, 10452-10457 (2004).

33 Mehle, A. & Doudna, J. A. An inhibitory activity in human cells restricts the function of an avian-like influenza virus polymerase. *Cell Host Microbe* **4**, 111-122 (2008).

34 Chen, H. *et al.* Properties and dissemination of H5N1 viruses isolated during an influenza outbreak in migratory waterfowl in western China. *J Virol* **80**, 5976-5983 (2006).

35 Chen, H. *et al.* Avian flu: H5N1 virus outbreak in migratory waterfowl. *Nature* **436**, 191-192 (2005).

36 Hatta, M., Gao, P., Halfmann, P. & Kawaoka, Y. Molecular basis for high virulence of Hong Kong H5N1 influenza A viruses. *Science* **293**, 1840-1842 (2001).

37 de Jong, M. D. *et al.* Fatal outcome of human influenza A (H5N1) is associated with high viral load and hypercytokinemia. *Nat Med* **12**, 1203-1207 (2006).

38 de Wit, E. & Fouchier, R. A. Emerging influenza. *J Clin Virol* **41**, 1-6 (2008).

39 Fouchier, R. A. *et al.* Avian influenza A virus (H7N7) associated with human conjunctivitis and a fatal case of acute respiratory distress syndrome. *Proc Natl Acad Sci U S A* **101**, 1356-1361 (2004).

40 Maines, T. R. *et al.* Avian influenza (H5N1) viruses isolated from humans in Asia in 2004 exhibit increased virulence in mammals. *J Virol* **79**, 11788-11800 (2005).

41 Munster, V. J. *et al.* The molecular basis of the pathogenicity of the Dutch highly pathogenic human influenza A H7N7 viruses. *J Infect Dis* **196**, 258-265 (2007).

42 Shinya, K., Watanabe, S., Ito, T., Kasai, N. & Kawaoka, Y. Adaptation of an H7N7 equine influenza A virus in mice. *J Gen Virol* **88**, 547-553 (2007).

43 Fornek, J. L. *et al.* A single-amino-acid substitution in a polymerase protein of an H5N1 influenza virus is associated with systemic infection and impaired T-cell activation in mice. *J Virol* **83**, 11102-11115 (2009).

44 Kim, J. H. *et al.* Role of host-specific amino acids in the pathogenicity of avian H5N1 influenza viruses in mice. *J Gen Virol* **91**, 1284-1289 (2010).

45 Kuzuhara, T. *et al.* Structural basis of the influenza A virus RNA polymerase PB2 RNA-binding domain containing the pathogenicity-determinant lysine 627 residue. *J Biol Chem* **284**, 6855-6860 (2009).

46 Mase, M. *et al.* Recent H5N1 avian influenza A virus increases rapidly in virulence to mice after a single passage in mice. *J Gen Virol* **87**, 3655-3659 (2006).

47 Rigoni, M. *et al.* Pneumo- and neurotropism of avian origin Italian highly pathogenic avian influenza H7N1 isolates in experimentally infected mice. *Virology* **364**, 28-35 (2007).

48 Bogs, J. *et al.* Reversion of PB2-627E to -627K during replication of an H5N1 Clade 2.2 virus in mammalian hosts depends on the origin of the nucleoprotein. *J Virol* **85**, 10691-10698 (2011).

49 Bortz, E. *et al.* Host- and strain-specific regulation of influenza virus polymerase activity by interacting cellular proteins. *MBio* **2**, doi:10.1128/mBio.00151-11 (2011).

50 Le, Q. M., Sakai-Tagawa, Y., Ozawa, M., Ito, M. & Kawaoka, Y. Selection of H5N1 influenza virus PB2 during replication in humans. *J Virol* **83**, 5278-5281 (2009).

51 Aggarwal, S., Dewhurst, S., Takimoto, T. & Kim, B. Biochemical impact of the host adaptation-associated PB2 E627K mutation on the temperature-dependent RNA synthesis kinetics of influenza A virus polymerase complex. *J Biol Chem* **286**, 34504-34513 (2011).

52 Zhang, T. *et al.* NEDDylation of PB2 Reduces Its Stability and Blocks the Replication of Influenza A Virus. *Sci Rep* **7**, 43691 (2017).

53 Gao, Y. *et al.* Identification of amino acids in HA and PB2 critical for the transmission of H5N1 avian influenza viruses in a mammalian host. *PLoS Pathog* **5**, e1000709 (2009).

54 Ma, W. *et al.* The role of swine in the generation of novel influenza viruses. *Zoonoses Public Health* **56**, 326-337 (2009).

55 Gabriel, G. *et al.* The viral polymerase mediates adaptation of an avian influenza virus to a mammalian host. *Proc Natl Acad Sci U S A* **102**, 18590-18595 (2005).

56 Gabriel, G., Herwig, A. & Klenk, H. D. Interaction of polymerase subunit PB2 and NP with importin alpha1 is a determinant of host range of influenza A virus. *PLoS Pathog* **4**, e11 (2008).

57 Li, Z. *et al.* Molecular basis of replication of duck H5N1 influenza viruses in a mammalian mouse model. *J Virol* **79**, 12058-12064 (2005).

58 Gabriel, G. *et al.* Differential polymerase activity in avian and mammalian cells determines host range of influenza virus. *J Virol* **81**, 9601-9604 (2007).

59 Elgendy, E. M. *et al.* Identification of polymerase gene mutations that affect viral replication in H5N1 influenza viruses isolated from pigeons. *J Gen Virol* **98**, 6-17 (2017).

60 Marjuki, H. *et al.* Three amino acid changes in PB1-F2 of highly pathogenic H5N1 avian influenza virus affect pathogenicity in mallard ducks. *Arch Virol* **155**, 925-934 (2010).

61 Chen, Q. *et al.* Adaptive amino acid substitutions enhance the virulence of an H7N7 avian influenza virus isolated from wild waterfowl in mice. *Vet Microbiol* **177**, 18-24 (2015).

62 Hulse-Post, D. J. *et al.* Molecular changes in the polymerase genes (PA and PB1) associated with high pathogenicity of H5N1 influenza virus in mallard ducks. *J Virol* **81**, 8515-8524 (2007).

63 Yu, Z. *et al.* A PB1 T296R substitution enhance polymerase activity and confer a virulent phenotype to a 2009 pandemic H1N1 influenza virus in mice. *Virology* **486**, 180-186 (2015).

64 Chen, W. *et al.* A novel influenza A virus mitochondrial protein that induces cell death. *Nat Med* **7**, 1306-1312 (2001).

65 Wei, K. *et al.* Influenza A virus acquires enhanced pathogenicity and transmissibility after serial passages in swine. *J Virol* **88**, 11981-11994 (2014).

66 Xu, C. *et al.* Amino acids 473V and 598P of PB1 from an avian-origin influenza A virus contribute to polymerase activity, especially in mammalian cells. *J Gen Virol* **93**, 531-540 (2012).

67 Conenello, G. M., Zamarin, D., Perrone, L. A., Tumpey, T. & Palese, P. A single mutation in the PB1-F2 of H5N1 (HK/97) and 1918 influenza A viruses contributes to increased virulence. *PLoS Pathog* **3**, 1414-1421 (2007).

68 Schmolke, M. *et al.* Differential contribution of PB1-F2 to the virulence of highly pathogenic H5N1 influenza A virus in mammalian and avian species. *PLoS Pathog* **7**, e1002186 (2011).

69 Ozawa, M. *et al.* Impact of amino acid mutations in PB2, PB1-F2, and NS1 on the replication and pathogenicity of pandemic (H1N1) 2009 influenza viruses. *J Virol* **85**, 4596-4601 (2011).

70 Lee, I. *et al.* Single PA mutation as a high yield determinant of avian influenza vaccines. *Sci Rep* **7**, 40675 (2017).

71 Yamayoshi, S. *et al.* Virulence-affecting amino acid changes in the PA protein of H7N9 influenza A viruses. *J Virol* **88**, 3127-3134 (2014).

72 Tan, L. *et al.* A combination of HA and PA mutations enhances virulence in a mouse-adapted H6N6 influenza A virus. *J Virol* **88**, 14116-14125 (2014).

73 Yamaji, R. *et al.* Mammalian adaptive mutations of the PA protein of highly pathogenic avian H5N1 influenza virus. *J Virol* **89**, 4117-4125 (2015).

74 Sun, Y. *et al.* Naturally occurring mutations in the PA gene are key contributors to increased virulence of pandemic H1N1/09 influenza virus in mice. *J Virol* **88**, 4600-4604 (2014).

75 Wu, H. *et al.* Multiple amino acid substitutions involved in the adaptation of avian-origin influenza A (H10N7) virus in mice. *Arch Virol* **161**, 977-980 (2016).

76 Nam, J. H. *et al.* Rapid virulence shift of an H5N2 avian influenza virus during a single passage in mice. *Arch Virol*, doi:10.1007/s00705-017-3451-9 (2017).

77 Cheng, K. *et al.* PB2-E627K and PA-T97I substitutions enhance polymerase activity and confer a virulent phenotype to an H6N1 avian influenza virus in mice. *Virology* **468-470**, 207-213 (2014).

78 Zhao, Y. *et al.* Adaptive amino acid substitutions enhance the virulence of a novel human H7N9 influenza virus in mice. *Vet Microbiol* **187**, 8-14, doi:10.1016/j.vetmic.2016.02.027 (2016).

79 Yu, Z. *et al.* Multiple amino acid substitutions involved in the adaptation of H6N1 avian influenza virus in mice. *Vet Microbiol* **174**, 316-321 (2014).

80 Zhao, H. *et al.* Novel residues in the PA protein of avian influenza H7N7 virus affect virulence in mammalian hosts. *Virology* **498**, 1-8 (2016).

81 Subbarao, K. & Shaw, M. W. Molecular aspects of avian influenza (H5N1) viruses isolated from humans. *Rev Med Virol* **10**, 337-348 (2000).

82 Song, J., Xu, J., Shi, J., Li, Y. & Chen, H. Synergistic Effect of S224P and N383D Substitutions in the PA of H5N1 Avian Influenza Virus Contributes to Mammalian Adaptation. *Sci Rep* **5**, 10510 (2015).

83 Song, J. *et al.* The PA protein directly contributes to the virulence of H5N1 avian influenza viruses in domestic ducks. *J Virol* **85**, 2180-2188 (2011).

84 Elderfield, R. A. *et al.* Accumulation of human-adapting mutations during circulation of A(H1N1)pdm09 influenza virus in humans in the United Kingdom. *J Virol* **88**, 13269-13283 (2014).

85 Xu, G. *et al.* Prevailing PA Mutation K356R in Avian Influenza H9N2 Virus Increases Mammalian Replication and Pathogenicity. *J Virol* **90**, 8105-8114 (2016).

86 Yamada, S. *et al.* Haemagglutinin mutations responsible for the binding of H5N1 influenza A viruses to human-type receptors. *Nature* **444**, 378-382 (2006).

87 Zhu, Y. *et al.* Identification and characterization of a novel antigenic epitope in the hemagglutinin of the escape mutants of H9N2 avian influenza viruses. *Vet Microbiol* **178**, 144-149 (2015).

88 Wu, W. L. *et al.* Antigenic profile of avian H5N1 viruses in Asia from 2002 to 2007. *J Virol* **82**, 1798-1807 (2008).

89 Su, Y., Yang, H. Y., Zhang, B. J., Jia, H. L. & Tien, P. Analysis of a point mutation in H5N1 avian influenza virus hemagglutinin in relation to virus entry into live mammalian cells. *Arch Virol* **153**, 2253-2261 (2008).

90 Yu, Z. *et al.* Adaptive amino acid substitutions enhance the virulence of a reassortant H7N1 avian influenza virus isolated from wild waterfowl in mice. *Virology* **476**, 233-239 (2015).

91 Kaverin, N. V. *et al.* Pleiotropic effects of amino acid substitutions in H5 hemagglutinin of influenza A escape mutants. *Virus Res* **210**, 81-89 (2015).

92 Henningson, J. N. *et al.* Comparative virulence of wild-type H1N1pdm09 influenza A isolates in swine. *Vet Microbiol* **176**, 40-49 (2015).

93 Carbone, V. *et al.* Molecular Characterisation of the Haemagglutinin Glycan-Binding Specificity of Egg-Adapted Vaccine Strains of the Pandemic 2009 H1N1 Swine Influenza A Virus. *Molecules* **20**, 10415-10434 (2015).

94 Wang, W. *et al.* Glycosylation at 158N of the hemagglutinin protein and receptor binding specificity synergistically affect the antigenicity and immunogenicity of a live attenuated H5N1 A/Vietnam/1203/2004 vaccine virus in ferrets. *J Virol* **84**, 6570-6577 (2010).

95 Yang, H., Carney, P. J., Donis, R. O. & Stevens, J. Structure and receptor complexes of the hemagglutinin from a highly pathogenic H7N7 influenza virus. *J Virol* **86**, 8645-8652 (2012).

96 Auewarakul, P. *et al.* An avian influenza H5N1 virus that binds to a human-type receptor. *J Virol* **81**, 9950-9955 (2007).

97 Watanabe, Y. *et al.* Acquisition of human-type receptor binding specificity by new H5N1 influenza virus sublineages during their emergence in birds in Egypt. *PLoS Pathog* **7**, e1002068 (2011).

98 Kumar, S. R., Prabakaran, M., Ashok Raj, K. V., He, F. & Kwang, J. Amino Acid Substitutions Improve the Immunogenicity of H7N7HA Protein and Protect Mice against Lethal H7N7 Viral Challenge. *PLoS One* **10**, e0128940 (2015).

99 Yang, Z. Y. *et al.* Immunization by avian H5 influenza hemagglutinin mutants with altered receptor binding specificity. *Science* **317**, 825-828 (2007).

100 Imai, H. *et al.* The HA and NS genes of human H5N1 influenza A virus contribute to high virulence in ferrets. *PLoS Pathog* **6**, e1001106 (2010).

101 Kongchanagul, A. *et al.* Positive selection at the receptor-binding site of haemagglutinin H5 in viral sequences derived from human tissues. *J Gen Virol* **89**, 1805-1810 (2008).

102 Naughtin, M. *et al.* Neuraminidase inhibitor sensitivity and receptor-binding specificity of Cambodian clade 1 highly pathogenic H5N1 influenza virus. *Antimicrob Agents Chemother* **55**, 2004-2010 (2011).

103 Peng, X. *et al.* Amino acid substitutions occurring during adaptation of an emergent H5N6 avian influenza virus to mammals. *Arch Virol* **161**, 1665-1670 (2016).

104 Chutinimitkul, S. *et al.* In vitro assessment of attachment pattern and replication efficiency of H5N1 influenza A viruses with altered receptor specificity. *J Virol* **84**, 6825-6833 (2010).

105 Ilyushina, N. A., Govorkova, E. A., Gray, T. E., Bovin, N. V. & Webster, R. G. Human-Like Receptor Specificity Does Not Affect the Neuraminidase-Inhibitor Susceptibility of H5N1 Influenza Viruses. *Plos Pathogens* **4**, e1000043 (2008).

106 Imai, M. *et al.* Experimental adaptation of an influenza H5 HA confers respiratory droplet transmission to a reassortant H5 HA/H1N1 virus in ferrets. *Nature* **486**, 420-428 (2012).

107 Yen, H. L. *et al.* Changes in H5N1 influenza virus hemagglutinin receptor binding domain affect systemic spread. *Proc Natl Acad Sci U S A* **106**, 286-291 (2009).

108 Chen, L. M. *et al.* In vitro evolution of H5N1 avian influenza virus toward human-type receptor specificity. *Virology* **422**, 105-113 (2012).

109 Maines, T. R. *et al.* Effect of receptor binding domain mutations on receptor binding and transmissibility of avian influenza H5N1 viruses. *Virology* **413**, 139-147 (2011).

110 Matos-Patron, A., Byrd-Leotis, L., Steinhauer, D. A., Barclay, W. S. & Ayora-Talavera, G. Amino acid substitution D222N from fatal influenza infection affects receptor-binding properties of the influenza A(H1N1)pdm09 virus. *Virology* **484**, 15-21 (2015).

111 Wang, F. *et al.* Adaptation of avian influenza A (H6N1) virus from avian to human receptor-binding preference. *Embo j* **34**, 1661-1673 (2015).

112 Teng, Q. *et al.* A Single Mutation at Position 190 in Hemagglutinin Enhances Binding Affinity for Human Type Sialic Acid Receptor and Replication of H9N2 Avian Influenza Virus in Mice. *J Virol* **90**, 9806-9825 (2016).

113 Han, P. F. *et al.* H5N1 influenza A virus with K193E and G225E double mutations in haemagglutinin is attenuated and immunogenic in mice. *J Gen Virol* **96**, 2522-2530 (2015).

114 Suzuki, Y., Kato, H., Naeve, C. W. & Webster, R. G. Single-amino-acid substitution in an antigenic site of influenza virus hemagglutinin can alter the specificity of binding to cell membrane-associated gangliosides. *J Virol* **63**, 4298-4302 (1989).

115 Manz, B., Matrosovich, M., Bovin, N. & Schwemmle, M. A polymorphism in the hemagglutinin of the human isolate of a highly pathogenic H5N1 influenza virus determines organ tropism in mice. *J Virol* **84**, 8316-8321 (2010).

116 Ilyushina, N. A. *et al.* Adaptation of pandemic H1N1 influenza viruses in mice. *J Virol* **84**, 8607-8616 (2010).

117 Glaser, L. *et al.* A single amino acid substitution in 1918 influenza virus hemagglutinin changes receptor binding specificity. *J Virol* **79**, 11533-11536 (2005).

118 Stevens, J. *et al.* Glycan microarray analysis of the hemagglutinins from modern and pandemic influenza viruses reveals different receptor specificities. *J Mol Biol* **355**, 1143-1155 (2006).

119 Tumpey, T. M. *et al.* A two-amino acid change in the hemagglutinin of the 1918 influenza virus abolishes transmission. *Science* **315**, 655-659 (2007).

120 Robertson, J. S. *et al.* The development of vaccine viruses against pandemic A(H1N1) influenza. *Vaccine* **29**, 1836-1843 (2011).

121 Chen, Z. *et al.* Generation of live attenuated novel influenza virus A/California/7/09 (H1N1) vaccines with high yield in embryonated chicken eggs. *J Virol* **84**, 44-51 (2010).

122 Chutinimitkul, S. *et al.* Virulence-associated substitution D222G in the hemagglutinin of 2009 pandemic influenza A(H1N1) virus affects receptor binding. *J Virol* **84**, 11802-11813 (2010).

123 Harvey, R., Martin, A. C., Zambon, M. & Barclay, W. S. Restrictions to the adaptation of influenza a virus h5 hemagglutinin to the human host. *J Virol* **78**, 502-507 (2004).

124 He, L. *et al.* Two amino acid substitutions in the haemagglutinin of the 2009 pandemic H1N1 virus decrease direct-contact transmission in guinea pigs. *J Gen Virol* **95**, 2612-2617 (2014).

125 Gambaryan, A. *et al.* Evolution of the receptor binding phenotype of influenza A (H5) viruses. *Virology* **344**, 432-438 (2006).

126 Shinya, K. *et al.* A mutation in H5 haemagglutinin that conferred human receptor recognition is not maintained stably during duck passage. *J Gen Virol* **91**, 1461-1463 (2010).

127 Xu, W. *et al.* Genomic signature analysis of the recently emerged highly pathogenic A(H5N8) avian influenza virus: implying an evolutionary trend for bird-to-human transmission. *Microbes Infect* **19**, 597-604 (2017).

128 Sang, X. *et al.* Adaptation of H9N2 AIV in guinea pigs enables efficient transmission by direct contact and inefficient transmission by respiratory droplets. *Sci Rep* **5**, 15928 (2015).

129 Sang, X. *et al.* Rapid emergence of a PB2-E627K substitution confers a virulent phenotype to an H9N2 avian influenza virus during adoption in mice. *Arch Virol* **160**, 1267-1277 (2015).

130 Chai, N. *et al.* Two Escape Mechanisms of Influenza A Virus to a Broadly Neutralizing Stalk-Binding Antibody. *PLoS Pathog* **12**, e1005702 (2016).

131 Gaiotto, T. & Hufton, S. E. Cross-Neutralising Nanobodies Bind to a Conserved Pocket in the Hemagglutinin Stem Region Identified Using Yeast Display and Deep Mutational Scanning. *PLoS One* **11**, e0164296 (2016).

132 Krenn, B. M. *et al.* Single HA2 mutation increases the infectivity and immunogenicity of a live attenuated H5N1 intranasal influenza vaccine candidate lacking NS1. *PLoS One* **6**, e18577 (2011).

133 Reed, M. L. *et al.* Amino acid residues in the fusion peptide pocket regulate the pH of activation of the H5N1 influenza virus hemagglutinin protein. *J Virol* **83**, 3568-3580 (2009).

134 Yu, Z. *et al.* PB2 and HA mutations increase the virulence of highly pathogenic H5N5 clade 2.3.4.4 avian influenza virus in mice. *Arch Virol* **163**, 401-410 (2018).

135 Zhu, W., Zou, X., Zhou, J., Tang, J. & Shu, Y. Residues 41V and/or 210D in the NP protein enhance polymerase activities and potential replication of novel influenza (H7N9) viruses at low temperature. *Virol J* **12**, 71 (2015).

136 Chen, L. *et al.* Amino Acid Substitution K470R in the Nucleoprotein Increases the Virulence of H5N1 Influenza A Virus in Mammals. *Front Microbiol* **8**, 1308 (2017).

137 Li, J. *et al.* Differential nucleocytoplasmic shuttling of the nucleoprotein of influenza a viruses and association with host tropism. *Cell Microbiol* **19**, doi:10.1111/cmi.12692 (2017).

138 Narkpuk, J., Teeravechyan, S., Puthavathana, P., Jongkaewwattana, A. & Jaru-Ampornpan, P. Single nucleoprotein residue determines influenza A virus sensitivity to an intertypic suppression mechanism. *Virology* **506**, 99-109 (2017).

139 Eshaghi, A. *et al.* Multiple influenza A (H3N2) mutations conferring resistance to neuraminidase inhibitors in a bone marrow transplant recipient. *Antimicrob Agents Chemother* **58**, 7188-7197 (2014).

140 L'Huillier, A. G. *et al.* E119D Neuraminidase Mutation Conferring Pan-Resistance to Neuraminidase Inhibitors in an A(H1N1)pdm09 Isolate From a Stem-Cell Transplant Recipient. *J Infect Dis* **212**, 1726-1734 (2015).

141 Dapat, C. *et al.* Rare influenza A (H3N2) variants with reduced sensitivity to antiviral drugs. *Emerg Infect Dis* **16**, 493-496 (2010).

142 Okomo-Adhiambo, M. *et al.* Host cell selection of influenza neuraminidase variants: implications for drug resistance monitoring in A(H1N1) viruses. *Antiviral Res* **85**, 381-388 (2010).

143 Mishin, V. P. *et al.* The effect of the MDCK cell selected neuraminidase D151G mutation on the drug susceptibility assessment of influenza A(H3N2) viruses. *Antiviral Res* **101**, 93-96 (2014).

144 Correia, V., Santos, L. A., Giria, M., Almeida-Santos, M. M. & Rebelo-de-Andrade, H. Influenza A(H1N1)pdm09 resistance and cross-decreased susceptibility to oseltamivir and zanamivir antiviral drugs. *J Med Virol* **87**, 45-56 (2015).

145 DeVries, A. *et al.* Neuraminidase H275Y and hemagglutinin D222G mutations in a fatal case of 2009 pandemic influenza A (H1N1) virus infection. *Influenza Other Respir Viruses* **6**, e85-88 (2012).

146 Karthick, V. & Ramanathan, K. Insight into the oseltamivir resistance R292K mutation in H5N1 influenza virus: a molecular docking and molecular dynamics approach. *Cell Biochem Biophys* **68**, 291-299 (2014).

147 Wilson, J. R. *et al.* An influenza A virus (H7N9) anti-neuraminidase monoclonal antibody with prophylactic and therapeutic activity in vivo. *Antiviral Res* **135**, 48-55 (2016).

148 Mosaad, Z., Arafa, A., Hussein, H. A. & Shalaby, M. A. Mutation signature in neuraminidase gene of avian influenza H9N2/G1 in Egypt. *Virusdisease* **28**, 164-173 (2017).

149 Romero-Beltran, L. *et al.* Mutations at highly conserved residues in influenza A(H1N1)pdm09 virus affect neuraminidase activity. *Virus Res* **225**, 1-9 (2016).

150 Takashita, E. *et al.* Characterization of a large cluster of influenza A(H1N1)pdm09 viruses cross-resistant to oseltamivir and peramivir during the 2013-2014 influenza season in Japan. *Antimicrob Agents Chemother* **59**, 2607-2617 (2015).

151 Tu, V. *et al.* The I427T neuraminidase (NA) substitution, located outside the NA active site of an influenza A(H1N1)pdm09 variant with reduced susceptibility to NA inhibitors, alters NA properties and impairs viral fitness. *Antiviral Res* **137**, 6-13 (2017).

152 Fan, S. *et al.* Two amino acid residues in the matrix protein M1 contribute to the virulence difference of H5N1 avian influenza viruses in mice. *Virology* **384**, 28-32 (2009).

153 Zhang, K. *et al.* Two polar residues within C-terminal domain of M1 are critical for the formation of influenza A Virions. *Cell Microbiol* **17**, 1583-1593 (2015).

154 Jiao, P. *et al.* A single-amino-acid substitution in the NS1 protein changes the pathogenicity of H5N1 avian influenza viruses in mice. *J Virol* **82**, 1146-1154 (2008).

155 Kaewborisuth, C. *et al.* G45R on nonstructural protein 1 of influenza A virus contributes to virulence by increasing the expression of proinflammatory cytokines in mice. *Arch Virol* **162**, 45-55 (2017).

156 DeDiego, M. L., Nogales, A., Lambert-Emo, K., Martinez-Sobrido, L. & Topham, D. J. NS1 Protein Mutation I64T Affects Interferon Responses and Virulence of Circulating H3N2 Human Influenza A Viruses. *J Virol* **90**, 9693-9711 (2016).

157 Kanrai, P. *et al.* Identification of specific residues in avian influenza A virus NS1 that enhance viral replication and pathogenicity in mammalian systems. *J Gen Virol* **97**, 2135-2148 (2016).

158 Zheng, W. *et al.* Threonine 80 phosphorylation of non-structural protein 1 regulates the replication of influenza A virus by reducing the binding affinity with RIG-I. *Cell Microbiol* **19**, doi:10.1111/cmi.12643 (2017).

159 Long, J. X., Peng, D. X., Liu, Y. L., Wu, Y. T. & Liu, X. F. Virulence of H5N1 avian influenza virus enhanced by a 15-nucleotide deletion in the viral nonstructural gene. *Virus Genes* **36**, 471-478 (2008).

160 Seo, S. H., Hoffmann, E. & Webster, R. G. Lethal H5N1 influenza viruses escape host anti-viral cytokine responses. *Nat Med* **8**, 950-954 (2002).

161 Wang, B. X., Wei, L., Kotra, L. P., Brown, E. G. & Fish, E. N. A Conserved Residue, Tyrosine (Y) 84, in H5N1 Influenza A Virus NS1 Regulates IFN Signaling Responses to Enhance Viral Infection. *Viruses* **9**, doi:10.3390/v9050107 (2017).

162 Li, M. & Wang, B. Homology modeling and examination of the effect of the D92E mutation on the H5N1 nonstructural protein NS1 effector domain. *J Mol Model* **13**, 1237-1244 (2007).

163 Solorzano, A. *et al.* Mutations in the NS1 protein of swine influenza virus impair anti-interferon activity and confer attenuation in pigs. *J Virol* **79**, 7535-7543 (2005).

164 Kuo, R. L. & Krug, R. M. Influenza a virus polymerase is an integral component of the CPSF30-NS1A protein complex in infected cells. *J Virol* **83**, 1611-1616 (2009).

165 Spesock, A. *et al.* The virulence of 1997 H5N1 influenza viruses in the mouse model is increased by correcting a defect in their NS1 proteins. *J Virol* **85**, 7048-7058 (2011).

166 Wang, B. X., Brown, E. G. & Fish, E. N. Residues F103 and M106 within the influenza A virus NS1 CPSF4-binding region regulate interferon-stimulated gene translation initiation. *Virology* **508**, 170-179 (2017).

167 Dankar, S. K. *et al.* Influenza A virus NS1 gene mutations F103L and M106I increase replication and virulence. *Virol J* **8**, 13 (2011).

168 Min, J. Y., Li, S., Sen, G. C. & Krug, R. M. A site on the influenza A virus NS1 protein mediates both inhibition of PKR activation and temporal regulation of viral RNA synthesis. *Virology* **363**, 236-243 (2007).

169 Li, Z. *et al.* The NS1 gene contributes to the virulence of H5N1 avian influenza viruses. *J Virol* **80**, 11115-11123 (2006).

170 Nogales, A., Martinez-Sobrido, L. & Topham, D. J. NS1 Protein Amino Acid Changes D189N and V194I Affect Interferon Responses, Thermosensitivity, and Virulence of Circulating H3N2 Human Influenza A Viruses. *J Virol* **91**, doi:10.1128/jvi.01930-16 (2017).

171 Bornholdt, Z. A. & Prasad, B. V. X-ray structure of NS1 from a highly pathogenic H5N1 influenza virus. *Nature* **456**, 985-988 (2008).
