# Supplementary material for: Potential Pandemic of H7N9 Avian Influenza A Virus in Human
Source: Front Cell Infect Microbiol. 2018 Nov 23;8:414. doi: 10.3389/fcimb.2018.00414 (PMC6265602; doi:10.3389/fcimb.2018.00414)
Supplement: Supplementary Table 2 — The proportion of 63 amino acid substitutions that associated with AIVs to infect mammals in H7N9 and H9N2 viruses. 58 of them have very high proportions not only in human-isolates, but also in avian-isolated H7N9 AIVs. [file Table_2.DOCX]

**Supplementary table 2.** The frequency of 63 amino acid substitutions that associated with AIVs to infect mammals in H7N9 and H9N2 viruses. 58 of them have very high proportions not only in human-isolates, but also in avian-isolated H7N9 AIVs.

| **Gene** | **Amino acid changes** | **The 1^st^ wave** | | **The 2^nd^ wave** | | **The 3^rd^ wave** | | **The 4^th^ wave** | | **The 5^th^ wave** | |  |
| --- | --- | --- | --- | --- | --- | --- | --- | --- | --- | --- | --- | --- |
|  |  | **human** | **avian** | **human** | **avian** | **human** | **avian** | **human** | **avian** | **human** | **avian** |  |
| HA (H7N9) | I116M | 100.00% | 100.00% | 100.00% | 100.00% | 100.00% | 100.00% | 100.00% | 100.00% | 100.00% | 100.00% |  |
|  | T131A | 98.41% | 99.33% | 95.87% | 89.60% | 38.19% | 41.56% | 14.29% | 50.00% | 3.03% | 6.25% |  |
|  | N154D | 100.00% | 100.00% | 100.00% | 100.00% | 100.00% | 100.00% | 100.00% | 100.00% | 99.50% | 100.00% |  |
|  | S155N | 93.70% | 100.00% | 100.00% | 99.80% | 100.00% | 100.00% | 100.00% | 100.00% | 100.00% | 100.00% |  |
|  | T156A | 99.20% | 100% | 97.90% | 99.80% | 99.30% | 100% | 100% | 100% | 96.50% | 100% |  |
|  | G182V | 96.83% | 100.00% | 99.17% | 99.12% | 89.58% | 84.42% | 100.00% | 100.00% | 98.48% | 100.00% |  |
|  | I198V | 99.21% | 100.00% | 100.00% | 99.78% | 100.00% | 100.00% | 100.00% | 100.00% | 100.00% | 100.00% |  |
|  | S205Y | 99.20% | 100% | 100% | 100% | 100% | 100% | 100% | 100% | 100% | 100% |  |
|  | D221G | 99.20% | 100% | 98.80% | 100% | 97.20% | 100% | 100% | 100% | 99% | 100% |  |
|  | Q222L | 91.30% | 83.20% | 97.10% | 98.20% | 93.80% | 87% | 100% | 100% | 80.30% | 9.40% |  |
| NA (H7N9) | M26I | 94.26% | 97.90% | 99.59% | 93.41% | 100.00% | 93.67% | 100.00% | 100.00% | 100.00% | 93.75% |  |
|  | R143K | 100.00% | 100.00% | 100.00% | 100.00% | 100.00% | 100.00% | 100.00% | 100.00% | 100.00% | 100.00% |  |
|  | T223I | 100% | 100% | 100% | 100% | 99.30% | 100% | 100% | 100% | 99.40% | 100% |  |
|  | N390K | 100.00% | 100.00% | 100.00% | 99.78% | 100.00% | 100.00% | 100.00% | 100.00% | 100.00% | 100.00% |  |
| PA (H7N9) | V100A | 8.85% | 26.61% | 32.64% | 64.04% | 42.86% | 60.87% | 14.29% | 75.00% | 70.48% | 13.04% |  |
|  | V135A | 99.12% | 100.00% | 100.00% | 99.74% | 100.00% | 100.00% | 100.00% | 100.00% | 100.00% | 100.00% |  |
|  | H266R | 99.12% | 100.00% | 100.00% | 100.00% | 100.00% | 100.00% | 100.00% | 100.00% | 100.00% | 100.00% |  |
|  | F277S | 98.23% | 100.00% | 99.15% | 99.74% | 100.00% | 100.00% | 100.00% | 100.00% | 100.00% | 100.00% |  |
|  | C278Q | 99.12% | 100.00% | 100.00% | 100.00% | 100.00% | 100.00% | 85.71% | 100.00% | 100.00% | 100.00% |  |
|  | K356R | 99.12% | 97.20% | 77.54% | 73.23% | 94.66% | 95.65% | 100.00% | 100.00% | 100.00% | 95.65% |  |
|  | N383D | 99.12% | 100.00% | 100.00% | 98.69% | 100.00% | 100.00% | 100.00% | 100.00% | 100.00% | 95.65% |  |
|  | S409N | 93.81% | 84.68% | 92.89% | 86.61% | 93.23% | 93.48% | 100.00% | 100.00% | 98.80% | 95.65% |  |
|  | S/A515T | 99.12% | 100.00% | 100.00% | 100.00% | 100.00% | 100.00% | 100.00% | 100.00% | 100.00% | 100.00% |  |
|  | L653P | 100.00% | 98.13% | 100.00% | 99.48% | 100.00% | 100.00% | 100.00% | 100.00% | 100.00% | 100.00% |  |
| PA (H9N2) | V100A | 2.43% | | 16.82% | | 20.18% | | 5.26% | | 0.00% | |  |
|  | V135A | 100.00% | | 98.41% | | 98.96% | | 100.00% | | 100.00% | |  |
|  | H266R | 99.64% | | 99.38% | | 99.08% | | 100.00% | | 100.00% | |  |
|  | F277S | 97.75% | | 94.92% | | 100.00% | | 92.86% | | 88.89% | |  |
|  | C278Q | 100.00% | | 100.00% | | 100.00% | | 100.00% | | 100.00% | |  |
|  | K356R | 62.16% | | 76.19% | | 63.54% | | 52.38% | | 88.89% | |  |
|  | N383D | 100.00% | | 98.73% | | 100.00% | | 100.00% | | 100.00% | |  |
|  | S409N | 49.18% | | 83.08% | | 65.14% | | 65.85% | | 100.00% | |  |
|  | S/A515T | 99.93% | | 99.38% | | 100.00% | | 100.00% | | 100.00% | |  |
|  | L653P | 99.55% | | 97.78% | | 97.92% | | 100.00% | | 100.00% | |  |
| NP (H7N9) | V41I | 100.00% | 100.00% | 98.73% | 100.00% | 99.24% | 100.00% | 100.00% | 100.00% | 100.00% | 100.00% |  |
|  | D210E | 100.00% | 100.00% | 99.58% | 100.00% | 100.00% | 100.00% | 100.00% | 100.00% | 100.00% | 100.00% |  |
|  | F253I | 100.00% | 100.00% | 100.00% | 100.00% | 99.24% | 100.00% | 100.00% | 100.00% | 100.00% | 100.00% |  |
|  | I353V | 94.69% | 99.07% | 96.19% | 98.95% | 93.13% | 100.00% | 100.00% | 100.00% | 100.00% | 100.00% |  |
| NP (H9N2) | V41I | 90.05% | | 95.19% | | 96.81% | | 89.74% | | 100.00% | |  |
|  | D210E | 91.86% | | 97.76% | | 85.11% | | 71.79% | | 88.89%,, | |  |
|  | F253I | 100.00% | | 100.00% | | 100.00% | | 100.00% | | 100.00% | |  |
|  | I353V | 81.90% | | 86.86% | | 77.66% | | 66.67% | | 88.89% | |  |
| M1 (H7N9) | V15I/T | 95.97% | 90.32% | 100.00% | 99.74% | 93.66% | 100.00% | 100.00% | 100.00% | 99.41% | 100.00% |  |
|  | N30D | 100.00% | 99.19% | 100.00% | 100.00% | 100.00% | 100.00% | 100.00% | 100.00% | 100.00% | 100.00% |  |
|  | T215A | 100.00% | 100.00% | 100.00% | 100.00% | 100.00% | 100.00% | 100.00% | 100.00% | 100.00% | 100.00% |  |
| M1 (H9N2) | V15I/T | 84.87% | | 98.20% | | 93.52% | | 92.50% | | 100.00% | |  |
|  | N30D | 84.87% | | 98.20% | | 93.52% | | 92.50% | | 100.00% | |  |
|  | T215A | 99.21% | | 100.00% | | 100.00% | | 100.00% | | 100.00% | |  |
| M2 (H7N9) | S31N | 100.00% | 91.13% | 99.58% | 99.74% | 100.00% | 100.00% | 100.00% | 100.00% | 100.00% | 100.00% |  |
|  | L55F | 100.00% | 91.13% | 100.00% | 98.69% | 100.00% | 100.00% | 100.00% | 100.00% | 98.24% | 100.00% |  |
| M2 (H9N2) | S31N | 0.00% | | 93.09% | | 86.11% | | 85.00% | | 100.00% | |  |
|  | L55F | 81.83% | | 96.70% | | 90.74% | | 90.00% | | 100.00% | |  |
| NS1 (H7N9) | A/P42S | 100.00% | 100.00% | 100.00% | 100.00% | 100.00% | 100.00% | 100.00% | 100.00% | 100.00% | 100.00% |  |
|  | F103L | 100.00% | 100.00% | 100.00% | 99.21% | 99.24% | 100.00% | 100.00% | 100.00% | 99.40% | 100.00% |  |
|  | M106I | 100.00% | 100.00% | 100.00% | 99.74% | 100.00% | 100.00% | 100.00% | 100.00% | 100.00% | 100.00% |  |
|  | V149A | 100.00% | 100.00% | 100.00% | 100.00% | 100.00% | 100.00% | 100.00% | 100.00% | 100.00% | 100.00% |  |
|  | N200S | 99.19% | 100.00% | 98.75% | 97.12% | 97.18% | 95.74% | 85.71% | 100.00% | 96.47% | 100.00% |  |
| NS1 (H9N2) | A/P42S | 96.47% | | 99.41% | | 100.00% | | 100.00% | | 100.00% | |  |
|  | F103L | 82.88% | | 95.19% | | 73.40% | | 69.23% | | 88.89% | |  |
|  | M106I | 82.88% | | 91.99% | | 74.47% | | 53.85% | | 88.89% | |  |
|  | V149A | 100.00% | | 100.00% | | 100.00% | | 100.00% | | 100.00% | |  |
|  | N200S | 75.21% | | 94.96% | | 96.64% | | 100.00% | | 100.00% | |  |
| NS2 (H7N9) | T47A | 99.19% | 100.00% | 98.75% | 98.43% | 97.18% | 93.62% | 85.71% | 100.00% | 100.00% | 100.00% |  |
| NS2 (H9N2) | T47A | 77.27% | | 92.88% | | 96.64% | | 100.00% | | 100.00% | |  |
| PB1 (H7N9) | A3V | 98.29% | 87.60% | 96.65% | 100.00% | 90.91% | 100.00% | 100.00% | 100.00% | 100.00% | 100.00% |  |
|  | L13P | 100.00% | 98.35% | 96.65% | 100.00% | 94.41% | 100.00% | 100.00% | 100.00% | 100.00% | 100.00% |  |
|  | R207K | 95.73% | 100.00% | 100.00% | 100.00% | 100.00% | 100.00% | 100.00% | 100.00% | 100.00% | 100.00% |  |
|  | K328N | 96.58% | 99.17% | 86.19% | 98.16% | 95.80% | 89.13% | 100.00% | 100.00% | 99.40% | 100.00% |  |
|  | I368V | 85.47% | 88.43% | 99.58% | 98.43% | 99.30% | 100.00% | 100.00% | 100.00% | 96.39% | 100.00% |  |
|  | S375N/T | 94.02% | 100.00% | 100.00% | 99.48% | 99.30% | 100.00% | 100.00% | 100.00% | 98.80% | 100.00% |  |
|  | H436Y | 95.73% | 100.00% | 100.00% | 100.00% | 99.30% | 100.00% | 100.00% | 100.00% | 100.00% | 100.00% |  |
|  | A469T | 99.12% | 100.00% | 100.00% | 100.00% | 100.00% | 100.00% | 100.00% | 100.00% | 100.00% | 100.00% |  |
|  | L473V | 96.58% | 100.00% | 100.00% | 100.00% | 100.00% | 100.00% | 100.00% | 100.00% | 100.00% | 100.00% |  |
|  | V652A | 99.12% | 100.00% | 100.00% | 90.81% | 100.00% | 100.00% | 100.00% | 100.00% | 99.40% | 100.00% |  |
|  | M677T | 99.15% | 100.00% | 100.00% | 97.90% | 98.60% | 100.00% | 100.00% | 100.00% | 99.40% | 100.00% |  |
| PB1 (H9N2) | A3V | 84.84% | | 98.16% | | 98.13% | | 100.00% | | 77.78% | |  |
|  | L13P | 95.40% | | 99.69% | | 99.07% | | 100.00% | | 77.78% | |  |
|  | R207K | 98.71% | | 99.69% | | 99.07% | | 97.56% | | 100.00% | |  |
|  | K328N | 98.99% | | 94.48% | | 100.00% | | 100.00% | | 88.89% | |  |
|  | I368V | 31.61% | | 90.49% | | 72.90% | | 51.22% | | 77.78% | |  |
|  | S375N/T | 74.43% | | 95.40% | | 91.59% | | 87.80% | | 100.00% | |  |
|  | H436Y | 99.71% | | 99.69% | | 100.00% | | 100.00% | | 100.00% | |  |
|  | A469T | 100.00% | | 98.72% | | 100.00% | | 100.00% | | 100.00% | |  |
|  | L473V | 96.34% | | 99.39% | | 100.00% | | 100.00% | | 100.00% | |  |
|  | V652A | 99.10% | | 98.72% | | 97.87% | | 100.00% | | 100.00% | |  |
|  | M677T | 98.06% | | 100.00% | | 99.07% | | 100.00% | | 100.00% | |  |
| PB2 (H7N9) | T63I | 100.00% | 99.19% | 92.41% | 100.00% | 92.96% | 100.00% | 100.00% | 100.00% | 100.00% | 100.00% |  |
|  | L89V | 97.58% | 99.19% | 95.36% | 100.00% | 93.66% | 100.00% | 100.00% | 100.00% | 100.00% | 100.00% |  |
|  | K251R | 99.12% | 100.00% | 96.61% | 98.43% | 99.24% | 100.00% | 100.00% | 100.00% | 100.00% | 100.00% |  |
|  | G309D | 95.16% | 100.00% | 99.58% | 99.48% | 99.30% | 100.00% | 100.00% | 100.00% | 100.00% | 100.00% |  |
|  | T339K | 95.16% | 100.00% | 99.58% | 98.95% | 98.59% | 100.00% | 100.00% | 100.00% | 98.80% | 100.00% |  |
|  | Q368R | 95.16% | 100.00% | 99.16% | 95.80% | 99.30% | 100.00% | 100.00% | 100.00% | 100.00% | 100.00% |  |
|  | H447Q | 95.16% | 99.19% | 100.00% | 99.74% | 95.07% | 89.13% | 100.00% | 100.00% | 100.00% | 100.00% |  |
|  | I471T | 99.12% | 99.07% | 100.00% | 100.00% | 100.00% | 100.00% | 100.00% | 100.00% | 100.00% | 100.00% |  |
|  | R477G | 95.16% | 100.00% | 100.00% | 100.00% | 99.30% | 100.00% | 100.00% | 100.00% | 100.00% | 100.00% |  |
|  | I495V | 95.16% | 99.19% | 98.73% | 100.00% | 98.59% | 100.00% | 100.00% | 100.00% | 99.40% | 95.65% |  |
|  | K526R | 88.71% | 100.00% | 99.16% | 99.21% | 97.18% | 100.00% | 57.14% | 75.00% | 66.87% | 17.39% |  |
|  | E/D627K/N | 67.74% | 0.81% | 73.42% | 0.52% | 57.04% | 4.35% | 85.71% | 0.00% | 54.82% | 0.00% |  |
|  | D701N | 7.26% | 0.00% | 7.59% | 0.26% | 4.23% | 0.00% | 0.00% | 0.00% | 4.82% | 0.00% |  |
| PB2 (H9N2) | T63I | 74.88% | | 99.38% | | 91.07% | | 100.00% | | 100.00% | |  |
|  | L89V | 75.47% | | 99.69% | | 95.54% | | 100.00% | | 100.00% | |  |
|  | K251R | 99.55% | | 99.68% | | 98.94% | | 100.00% | | 100.00% | |  |
|  | G309D | 76.66% | | 98.77% | | 97.32% | | 100.00% | | 100.00% | |  |
|  | T339K | 78.07% | | 99.38% | | 99.11% | | 97.56% | | 100.00% | |  |
|  | Q368R | 92.37% | | 99.07% | | 98.21% | | 97.56% | | 100.00% | |  |
|  | H447Q | 96.64% | | 96.60% | | 100.00% | | 100.00% | | 100.00% | |  |
|  | I471T | 99.55% | | 99.68% | | 100.00% | | 100.00% | | 100.00% | |  |
|  | R477G | 97.83% | | 100.00% | | 100.00% | | 100.00% | | 100.00% | |  |
|  | I495V | 85.27% | | 94.14% | | 94.64% | | 100.00% | | 100.00% | |  |
|  | K526R | 3.25% | | 4.06% | | 5.56% | | 2.63% | | 0.00% | |  |
|  | E/D627K/N | 0.54% | | 0.93% | | 0.93% | | 33.33% | | 0.00% | |  |
|  | D701N | 0.00% | | 0.00% | | 25.00% | | 0.00% | | 0.00% | |  |
