# Supplementary material for: Potential Pandemic of H7N9 Avian Influenza A Virus in Human
Source: Front Cell Infect Microbiol. 2018 Nov 23;8:414. doi: 10.3389/fcimb.2018.00414 (PMC6265602; doi:10.3389/fcimb.2018.00414)
Supplement: Supplementary Table 3 — GenBank/GISAID accession numbers of viruses that used in this study. [file Table_3.DOCX]

Supplementary table 3: GenBank/GISAID accession numbers of viruses that used in this study.

|  |  | host | HA | NA | MP | NP | NS | PA | PB1 | PB2 |
| --- | --- | --- | --- | --- | --- | --- | --- | --- | --- | --- |
| A/turkey/Minnesota/38429/1988 | H7N9 | Avian |  |  | GU053164 | GU053166 | GU053167 | GU053168 | GU053169 |  |
| A/ruddy_turnstone/DE/1538/2000 | H7N9 | Avian |  |  | DQ021735 | HQ541732 | DQ021611 | HQ541733 | HQ541734 | HQ541735 |
| A/blue-winged_teal/Ohio/566/2006 | H7N9 | Avian |  |  | CY024819 | CY024821 | CY024822 | CY024823 | CY024824 | CY024825 |
| A/duck/Jiangxi/3096/2009 | H7N9 | Avian |  | KF259688 | KF259218 | KF259737 | KF259981 | KF260225 | KF260469 | KF260713 |
| A/duck/Jiangxi/3141/2009 | H7N9 | Avian |  | KF259689 | KF259219 | KF259738 | KF259982 | KF260226 | KF260470 | KF260714 |
| A/duck/Jiangxi/3152/2009 | H7N9 | Avian |  | KF259713 | KF259272 | KF259791 | KF260035 | KF260279 | KF260523 | KF260767 |
| A/duck/Jiangxi/3169/2009 | H7N9 | Avian |  | KF259690 | KF259220 | KF259739 | KF259983 | KF260227 | KF260471 | KF260715 |
| A/duck/Jiangxi/3190/2009 | H7N9 | Avian |  | KF259691 | KF259221 | KF259740 | KF259984 | KF260228 | KF260472 | KF260716 |
| A/duck/Jiangxi/3214/2009 | H7N9 | Avian |  | KF259692 | KF259222 | KF259741 | KF259985 | KF260229 | KF260473 | KF260717 |
| A/duck/Jiangxi/3230/2009 | H7N9 | Avian |  | KF259693 | KF259223 | KF259742 | KF259986 | KF260230 | KF260474 | KF260718 |
| A/duck/Jiangxi/3257/2009 | H7N9 | Avian |  | KF259714 | KF259273 | KF259792 | KF260036 | KF260280 | KF260524 | KF260768 |
| A/duck/Jiangxi/3283/2009 | H7N9 | Avian |  | KF259699 | KF259257 | KF259776 | KF260020 | KF260264 | KF260508 | KF260752 |
| A/duck/Jiangxi/3286/2009 | H7N9 | Avian |  | KF259694 | KF259224 | KF259743 | KF259987 | KF260231 | KF260475 | KF260719 |
| A/duck/Jiangxi/3292/2009 | H7N9 | Avian |  | KF259720 | KF259282 | KF259801 | KF260045 | KF260289 | KF260533 | KF260777 |
| A/chicken/Henan/100/2013 | H7N9 | Avian | KP185931 | KP185519 | KP185355 | KP185685 | KP185767 | KP185603 | KP185849 | KP185437 |
| A/chicken/Henan/102/2013 | H7N9 | Avian | KP185932 |  | KP185356 | KP185686 | KP185768 | KP185604 | KP185850 | KP185438 |
| A/chicken/Henan/103/2013 | H7N9 | Avian | KP185933 | KP185521 | KP185357 | KP185687 | KP185769 | KP185605 | KP185851 | KP185439 |
| A/chicken/Henan/106/2013 | H7N9 | Avian | KP185934 | KP185522 | KP185358 | KP185688 | KP185770 | KP185606 | KP185852 | KP185440 |
| A/chicken/Henan/107/2013 | H7N9 | Avian | KP185935 | KP185523 | KP185359 | KP185689 | KP185771 | KP185607 | KP185853 | KP185441 |
| A/chicken/Henan/109/2013 | H7N9 | Avian | KP185936 | KP185524 | KP185360 | KP185690 | KP185772 | KP185608 | KP185854 | KP185442 |
| A/chicken/Henan/112/2013 | H7N9 | Avian | KP185937 | KP185525 | KP185361 | KP185691 | KP185773 | KP185609 | KP185855 | KP185443 |
| A/chicken/Henan/114/2013 | H7N9 | Avian | KP185938 | KP185526 | KP185362 | KP185692 | KP185774 | KP185610 | KP185856 | KP185444 |
| A/chicken/Henan/115/2013 | H7N9 | Avian | KP185939 | KP185527 | KP185363 | KP185693 | KP185775 | KP185611 | KP185857 | KP185445 |
| A/chicken/Henan/117/2013 | H7N9 | Avian | KP185940 | KP185528 | KP185364 | KP185694 | KP185776 | KP185612 | KP185858 | KP185446 |
| A/chicken/Henan/120/2013 | H7N9 | Avian | KP185941 | KP185529 | KP185365 | KP185695 | KP185777 | KP185613 | KP185859 | KP185447 |
| A/chicken/Henan/141/2013 | H7N9 | Avian | KP185942 | KP185530 | KP185366 | KP185696 | KP185778 | KP185614 | KP185860 | KP185448 |
| A/chicken/Henan/89/2013 | H7N9 | Avian | KP185943 | KP185531 | KP185367 | KP185697 | KP185779 | KP185615 | KP185861 | KP185449 |
| A/chicken/Henan/93/2013 | H7N9 | Avian | KP185944 | KP185532 | KP185368 | KP185698 | KP185780 | KP185616 | KP185862 | KP185450 |
| A/chicken/Henan/96/2013 | H7N9 | Avian | KP185945 | KP185533 | KP185369 | KP185699 | KP185781 | KP185617 | KP185863 | KP185451 |
| A/chicken/Henan/97/2013 | H7N9 | Avian | KP185946 | KP185534 | KP185370 | KP185700 | KP185782 | KP185618 | KP185864 | KP185452 |
| A/chicken/Henan/99/2013 | H7N9 | Avian | KP185947 | KP185535 | KP185371 | KP185701 | KP185783 | KP185619 | KP185865 | KP185453 |
| A/chicken/Henan/F246/2013 | H7N9 | Avian | KP185948 | KP185536 | KP185372 | KP185702 | KP185784 | KP185620 | KP185866 | KP185454 |
| A/chicken/Henan/F248/2013 | H7N9 | Avian | KP185949 | KP185537 | KP185373 | KP185703 | KP185785 | KP185621 | KP185867 | KP185455 |
| A/chicken/Rizhao/515/2013 | H7N9 | Avian | KF259042 | KF259730 | KF259458 | KF259977 | KF260221 | KF260465 | KF260709 | KF260953 |
| A/chicken/Rizhao/713/2013 | H7N9 | Avian | KF259058 | KF259734 |  |  |  |  |  |  |
| A/chicken/Rizhao/715/2013 | H7N9 | Avian | KF259059 | KF259735 |  |  |  |  |  |  |
| A/chicken/Rizhao/719b/2013 | H7N9 | Avian | KF297293 | KF297317 |  |  |  |  |  |  |
| A/chicken/Rizhao/865/2013 | H7N9 | Avian | KF259060 | KF259736 |  |  |  |  |  |  |
| A/chicken/Rizhao/867/2013 | H7N9 | Avian | KF259043 | KF259731 | KF259459 | KF259978 | KF260222 | KF260466 | KF260710 | KF260954 |
| A/chicken/Rizhao/871/2013 | H7N9 | Avian | KF259044 | KF259732 | KF259460 | KF259979 | KF260223 | KF260467 | KF260711 | KF260955 |
| A/chicken/Rizhao/875/2013 | H7N9 | Avian | KF259045 | KF259733 | KF259461 | KF259980 | KF260224 | KF260468 | KF260712 | KF260956 |
| A/Chicken/Guangdong/GZ068/2015 | H7N9 | avian | EPI_ISL_176834 | EPI_ISL_176834 | EPI_ISL_176834 | EPI_ISL_176834 | EPI_ISL_176834 | EPI_ISL_176834 | EPI_ISL_176834 | EPI_ISL_176834 |
| A/Chicken/Guangdong/HZ098/2015 | H7N9 | avian | EPI_ISL_176824 | EPI_ISL_176824 | EPI_ISL_176824 | EPI_ISL_176824 | EPI_ISL_176824 | EPI_ISL_176824 | EPI_ISL_176824 | EPI_ISL_176824 |
| A/shoveler/Egypt/00215-NAMRU3/2007 | H7N9 | avian |  |  | EPI_ISL_120201 | EPI_ISL_120201 | EPI_ISL_120201 | EPI_ISL_120201 | EPI_ISL_120201 | EPI_ISL_120201 |
| A/Duck/Guangdong/DG103/2015 | H7N9 | avian | EPI_ISL_176828 | EPI_ISL_176828 | EPI_ISL_176828 | EPI_ISL_176828 | EPI_ISL_176828 | EPI_ISL_176828 | EPI_ISL_176828 | EPI_ISL_176828 |
| A/northern_shoverl/Mississippi/11OS145/2011 | H7N9 | Avian |  |  | CY133650 | CY133652 | CY133653 | CY133654 | CY133655 | CY133656 |
| A/chicken/China/028/2014 | H7N9 | Avian | KU318989 | KU318991 | KU318992 | KU318990 | KU318993 | KU318988 | KU318987 | KU318986 |
| A/American_green-winged_teal/Idaho/AH0006121/2015 | H7N9 | Avian |  |  | KY551303 | KY551301 | KY551304 | KY551299 | KY551298 | KY551297 |
| A/duck/Zhejiang/LS02/2014 | H7N9 | Avian | KP657909 | KP657911 | KP657912 | KP657910 | KP657913 | KP657908 | KP657907 | KP657906 |
| A/chicken/Huai'an/007/2014 | H7N9 | Avian | KP864448 | KP864452 | KX215921 | KX215931 | KX215940 | KX215951 | KX215961 | KX215971 |
| A/Chicken/Guangdong/DG120/2015 | H7N9 | avian | EPI_ISL_176832 | EPI_ISL_176832 | EPI_ISL_176832 | EPI_ISL_176832 | EPI_ISL_176832 | EPI_ISL_176832 | EPI_ISL_176832 | EPI_ISL_176832 |
| A/northern_shoveler/California/AH0076755/2016 | H7N9 | Avian |  |  | KY550836 | KY550834 | KY550837 | KY550832 | KY550831 | KY550830 |
| A/wild_duck/Korea/SH19-50/2010 | H7N9 | Avian |  | KC609798 | KC609938 | KC609910 | KC609966 | KC609882 | KC609854 | KC609826 |
| A/chicken/Taizhou/TZJF02/2015 | H7N9 | Avian | KU143281 | KU143374 | KU143332 | KU143419 | KU143460 | KU143505 | KU143549 | KU143594 |
| A/chicken/Wenzhou/HATSLG01/2015 | H7N9 | Avian | KU143283 | KU143377 | KU143334 | KU143421 | KU143462 | KU143506 | KU143550 | KU143592 |
| A/chicken/Wenzhou/RAQL01/2015 | H7N9 | Avian | KU143278 | KU143375 | KU143330 | KU143416 | KU143459 | KU143504 | KU143545 | KU143588 |
| A/chicken/Wenzhou/RAQL18/2015 | H7N9 | Avian | KU143279 | KU143376 | KU143331 | KU143418 | KU143465 | KU143503 | KU143547 | KU143590 |
| A/chicken/Wenzhou/WZTSLG02/2015 | H7N9 | Avian | KU143284 | KU143379 | KU143333 | KU143420 | KU143461 | KU143507 | KU143551 | KU143591 |
| A/duck/Wenzhou/RAQL10/2015 | H7N9 | Avian | KU143280 | KU143373 | KU143336 | KU143417 | KU143463 | KU143502 | KU143546 | KU143589 |
| A/duck/Wenzhou/YJYF24/2015 | H7N9 | Avian | KU143282 | KU143378 | KU143335 | KU143422 | KU143464 | KU143508 | KU143548 | KU143593 |
| A/chicken/Shanghai/PD-CN-02/2014 | H7N9 | Avian | KJ549786 | KJ549788 | KJ549789 | KJ549787 | KJ549790 | KJ549785 | KJ549784 | KJ549783 |
| A/cinnamon_teal/California/AH0079011/2016 | H7N9 | Avian |  |  | KY550805 | KY550803 | KY550806 | KY550801 | KY550800 | KY550799 |
| A/Anas_crecca/Spain/1460/2008 | H7N9 | Avian |  |  | HQ244410 | HQ244408 | HQ244411 | HQ244406 | HQ244405 | HQ244404 |
| A/silky_chicken/Hong_Kong/1772-3/2014 | H7N9 | avian | EPI_ISL_155815 | EPI_ISL_155815 | EPI_ISL_155815 | EPI_ISL_155815 | EPI_ISL_155815 | EPI_ISL_155815 | EPI_ISL_155815 | EPI_ISL_155815 |
| A/chicken/Ganzhou/GZ79/2016 | H7N9 | Avian | KY415641 | KY415729 | KY415685 | KY415773 | KY415949 | KY415817 | KY415861 | KY415905 |
| A/chicken/Longquan/LQ78/2016 | H7N9 | Avian | KY415630 | KY415718 | KY415674 | KY415762 | KY415938 | KY415806 | KY415850 | KY415894 |
| A/nothern_shoveler/California/AH0079371/2016 | H7N9 | Avian |  |  | KY550812 | KY550811 | KY550813 | KY550809 | KY550808 | KY550807 |
| A/Chicken/Guangdong/CZ145/2015 | H7N9 | avian | EPI_ISL_176835 | EPI_ISL_176835 | EPI_ISL_176835 | EPI_ISL_176835 | EPI_ISL_176835 | EPI_ISL_176835 | EPI_ISL_176835 | EPI_ISL_176835 |
| A/goose/Czech_Republic/1848-K9/2009 | H7N9 | Avian |  |  | GQ404575 | GU060483 | GU060485 | GU060481 | GU060480 | GU060479 |
| A/goose/Czech_Republic/1848-T14/2009 | H7N9 | Avian |  |  | HQ244418 | HQ244416 | HQ244419 | HQ244414 | HQ244413 | HQ244412 |
| A/chicken/Huai'an/041/2014 | H7N9 | Avian | KP864450 | KP864445 |  |  |  |  |  |  |
| A/duck/Huai'an/041/2014 | H7N9 | Avian |  |  |  |  |  | KX215952 | KX215962 | KX215972 |
| A/Chicken/Guangdong/SW153/2015 | H7N9 | avian | EPI_ISL_176836 | EPI_ISL_176836 | EPI_ISL_176836 | EPI_ISL_176836 | EPI_ISL_176836 | EPI_ISL_176836 | EPI_ISL_176836 | EPI_ISL_176836 |
| A/chicken/Huai'an/003/2015 | H7N9 | Avian | KP864442 | KP864454 | KX215920 | KX215930 | KX215939 | KX215950 | KX215960 | KX215970 |
| A/blue-winged_teal/Guatemala/CIP049-01/2008 | H7N9 | Avian |  |  | CY067673 | CY067671 | CY067674 | CY067669 | CY067668 | CY067667 |
| A/chicken/Huai'an/053/2014 | H7N9 | Avian | KP864451 | KP864453 |  |  |  |  |  |  |
| A/duck/Huai'an/053/2014 | H7N9 | Avian |  |  |  |  |  | KX215953 | KX215963 | KX215973 |
| A/Chicken/Guangdong/SW154/2015 | H7N9 | avian | EPI_ISL_176837 | EPI_ISL_176837 | EPI_ISL_176837 | EPI_ISL_176837 | EPI_ISL_176837 | EPI_ISL_176837 | EPI_ISL_176837 | EPI_ISL_176837 |
| A/chicken/Jiangxi/9497/2014 | H7N9 | Avian | KP417562 | KP417564 | KP417565 | KP417563 | KP417566 | KP417561 | KP417560 | KP417559 |
| A/chicken/Jiangxi/9508/2014 | H7N9 | Avian | KP414859 | KP414861 | KP414862 | KP414860 | KP414863 | KP414858 | KP414857 | KP414856 |
| A/chicken/Jiangxi/9513/2014 | H7N9 | Avian | KP417414 | KP417416 | KP417417 | KP417415 | KP417418 | KP417413 | KP417412 | KP417411 |
| A/chicken/Jiangxi/9530/2014 | H7N9 | Avian | KP417570 | KP417572 | KP417573 | KP417571 | KP417574 | KP417569 | KP417568 | KP417567 |
| A/chicken/Jiangxi/9534/2014 | H7N9 | Avian | KP414867 | KP414869 | KP414870 | KP414868 | KP414871 | KP414866 | KP414865 | KP414864 |
| A/chicken/Jiangxi/9558/2014 | H7N9 | Avian | KP414875 | KP414877 | KP414878 | KP414876 | KP414879 | KP414874 | KP414873 | KP414872 |
| A/silkie_chicken/Jiangxi/9469/2014 | H7N9 | Avian | KP414835 | KP414837 | KP414838 | KP414836 | KP414839 | KP414834 | KP414833 | KP414832 |
| A/silkie_chicken/Jiangxi/9472/2014 | H7N9 | Avian | KP414843 | KP414845 | KP414846 | KP414844 | KP414847 | KP414842 | KP414841 | KP414840 |
| A/silkie_chicken/Jiangxi/9476/2014 | H7N9 | Avian | KP414851 | KP414853 | KP414854 | KP414852 | KP414855 | KP414850 | KP414849 | KP414848 |
| A/chicken/Jiangxi/10552/2014 | H7N9 | Avian | KP414883 | KP414885 | KP414886 | KP414884 | KP414887 | KP414882 | KP414881 | KP414880 |
| A/chicken/Jiangxi/10573/2014 | H7N9 | Avian | KP414891 | KP414893 | KP414894 | KP414892 | KP414895 | KP414890 | KP414889 | KP414888 |
| A/chicken/Jiangxi/10870/2014 | H7N9 | Avian | KP414907 | KP414909 | KP414910 | KP414908 | KP414911 | KP414906 | KP414905 | KP414904 |
| A/chicken/Jiangxi/10871/2014 | H7N9 | Avian | KP414915 | KP414917 | KP414918 | KP414916 | KP414919 | KP414914 | KP414913 | KP414912 |
| A/chicken/Jiangxi/10873/2014 | H7N9 | Avian | KP414923 | KP414925 | KP414926 | KP414924 | KP414927 | KP414922 | KP414921 | KP414920 |
| A/chicken/Jiangxi/10874/2014 | H7N9 | Avian | KP414931 | KP414933 | KP414934 | KP414932 | KP414935 | KP414930 | KP414929 | KP414928 |
| A/chicken/Jiangxi/10875/2014 | H7N9 | Avian | KP414939 | KP414941 | KP414942 | KP414940 | KP414943 | KP414938 | KP414937 | KP414936 |
| A/chicken/Jiangxi/10877/2014 | H7N9 | Avian | KP414947 | KP414949 | KP414950 | KP414948 | KP414951 | KP414946 | KP414945 | KP414944 |
| A/chicken/Jiangxi/10882/2014 | H7N9 | Avian | KP414955 | KP414957 | KP414958 | KP414956 | KP414959 | KP414954 | KP414953 | KP414952 |
| A/chicken/Jiangxi/10885/2014 | H7N9 | Avian | KP414963 | KP414965 | KP414966 | KP414964 | KP414967 | KP414962 | KP414961 | KP414960 |
| A/chicken/Jiangxi/10894/2014 | H7N9 | Avian | KP414971 | KP414973 | KP414974 | KP414972 | KP414975 | KP414970 | KP414969 | KP414968 |
| A/chicken/Jiangxi/10895/2014 | H7N9 | Avian | KP414979 | KP414981 | KP414982 | KP414980 | KP414983 | KP414978 | KP414977 | KP414976 |
| A/chicken/Jiangxi/10896/2014 | H7N9 | Avian | KP414987 | KP414989 | KP414990 | KP414988 | KP414991 | KP414986 | KP414985 | KP414984 |
| A/chicken/Jiangxi/10897/2014 | H7N9 | Avian | KP414995 | KP414997 | KP414998 | KP414996 | KP414999 | KP414994 | KP414993 | KP414992 |
| A/chicken/Jiangxi/10929/2014 | H7N9 | Avian | KP415003 | KP415005 | KP415006 | KP415004 | KP415007 | KP415002 | KP415001 | KP415000 |
| A/chicken/Jiangxi/10939/2014 | H7N9 | Avian | KP415011 | KP415013 | KP415014 | KP415012 | KP415015 | KP415010 | KP415009 | KP415008 |
| A/chicken/Jiangxi/10943/2014 | H7N9 | Avian | KP415019 | KP415021 | KP415022 | KP415020 | KP415023 | KP415018 | KP415017 | KP415016 |
| A/chicken/Jiangxi/10945/2014 | H7N9 | Avian | KP415027 | KP415029 | KP415030 | KP415028 | KP415031 | KP415026 | KP415025 | KP415024 |
| A/chicken/Jiangxi/10946/2014 | H7N9 | Avian | KP415035 | KP415037 | KP415038 | KP415036 | KP415039 | KP415034 | KP415033 | KP415032 |
| A/chicken/Jiangxi/10947/2014 | H7N9 | Avian | KP415043 | KP415045 | KP415046 | KP415044 | KP415047 | KP415042 | KP415041 | KP415040 |
| A/chicken/Jiangxi/10948/2014 | H7N9 | Avian | KP415051 | KP415053 | KP415054 | KP415052 | KP415055 | KP415050 | KP415049 | KP415048 |
| A/chicken/Jiangxi/10950/2014 | H7N9 | Avian | KP415059 | KP415061 | KP415062 | KP415060 | KP415063 | KP415058 | KP415057 | KP415056 |
| A/chicken/Jiangxi/10953/2014 | H7N9 | Avian | KP415067 | KP415069 | KP415070 | KP415068 | KP415071 | KP415066 | KP415065 | KP415064 |
| A/chicken/Jiangxi/10954/2014 | H7N9 | Avian | KP415075 | KP415077 | KP415078 | KP415076 | KP415079 | KP415074 | KP415073 | KP415072 |
| A/chicken/Jiangxi/10955/2014 | H7N9 | Avian | KP415083 | KP415085 | KP415086 | KP415084 | KP415087 | KP415082 | KP415081 | KP415080 |
| A/chicken/Jiangxi/10956/2014 | H7N9 | Avian | KP415091 | KP415093 | KP415094 | KP415092 | KP415095 | KP415090 | KP415089 | KP415088 |
| A/chicken/Jiangxi/10957/2014 | H7N9 | Avian | KP415099 | KP415101 | KP415102 | KP415100 | KP415103 | KP415098 | KP415097 | KP415096 |
| A/chicken/Jiangxi/10958/2014 | H7N9 | Avian | KP415107 | KP415109 | KP415110 | KP415108 | KP415111 | KP415106 | KP415105 | KP415104 |
| A/chicken/Jiangxi/10959/2014 | H7N9 | Avian | KP415115 | KP415117 | KP415118 | KP415116 | KP415119 | KP415114 | KP415113 | KP415112 |
| A/chicken/Jiangxi/10961/2014 | H7N9 | Avian | KP415123 | KP415125 | KP415126 | KP415124 | KP415127 | KP415122 | KP415121 | KP415120 |
| A/chicken/Jiangxi/10962/2014 | H7N9 | Avian | KP415131 | KP415133 | KP415134 | KP415132 | KP415135 | KP415130 | KP415129 | KP415128 |
| A/chicken/Jiangxi/10963/2014 | H7N9 | Avian | KP415139 | KP415141 | KP415142 | KP415140 | KP415143 | KP415138 | KP415137 | KP415136 |
| A/chicken/Jiangxi/10964/2014 | H7N9 | Avian | KP415147 | KP415149 | KP415150 | KP415148 | KP415151 | KP415146 | KP415145 | KP415144 |
| A/chicken/Jiangxi/10965/2014 | H7N9 | Avian | KP415155 | KP415157 | KP415158 | KP415156 | KP415159 | KP415154 | KP415153 | KP415152 |
| A/chicken/Shenzhen/138/2014 | H7N9 | Avian | KP416510 | KP416512 | KP416513 | KP416511 | KP416514 | KP416509 | KP416508 | KP416507 |
| A/chicken/Dongguan/169/2014 | H7N9 | Avian | KP415428 | KP415430 | KP415431 | KP415429 | KP415432 | KP415427 | KP415426 | KP415425 |
| A/chicken/Dongguan/173/2014 | H7N9 | Avian | KP415436 | KP415438 | KP415439 | KP415437 | KP415440 | KP415435 | KP415434 | KP415433 |
| A/chicken/Dongguan/178/2014 | H7N9 | Avian | KP415924 | KP415926 | KP415927 | KP415925 | KP415928 | KP415923 | KP415922 | KP415921 |
| A/chicken/Dongguan/189/2014 | H7N9 | Avian | KP415444 | KP415446 | KP415447 | KP415445 | KP415448 | KP415443 | KP415442 | KP415441 |
| A/chicken/Dongguan/191/2014 | H7N9 | Avian | KP415932 | KP415934 | KP415935 | KP415933 | KP415936 | KP415931 | KP415930 | KP415929 |
| A/chicken/Dongguan/210/2014 | H7N9 | Avian | KP415940 | KP415942 | KP415943 | KP415941 | KP415944 | KP415939 | KP415938 | KP415937 |
| A/chicken/Dongguan/213/2014 | H7N9 | Avian | KP415948 | KP415950 | KP415951 | KP415949 | KP415952 | KP415947 | KP415946 | KP415945 |
| A/chicken/Dongguan/237/2014 | H7N9 | Avian | KP415956 | KP415958 | KP415959 | KP415957 | KP415960 | KP415955 | KP415954 | KP415953 |
| A/chicken/Dongguan/248/2014 | H7N9 | Avian | KP415964 | KP415966 | KP415967 | KP415965 | KP415968 | KP415963 | KP415962 | KP415961 |
| A/chicken/Dongguan/262/2014 | H7N9 | Avian | KP415972 | KP415974 | KP415975 | KP415973 | KP415976 | KP415971 | KP415970 | KP415969 |
| A/chicken/Dongguan/397/2014 | H7N9 | Avian | KP416558 | KP416560 | KP416561 | KP416559 | KP416562 | KP416557 | KP416556 | KP416555 |
| A/chicken/Dongguan/449/2014 | H7N9 | Avian | KP415462 | KP415464 | KP415465 | KP415463 | KP415466 | KP415461 | KP415460 | KP415459 |
| A/chicken/Dongguan/518/2014 | H7N9 | Avian | KP415470 | KP415472 | KP415473 | KP415471 | KP415474 | KP415469 | KP415468 | KP415467 |
| A/chicken/Dongguan/536/2014 | H7N9 | Avian | KP415478 | KP415480 | KP415481 | KP415479 | KP415482 | KP415477 | KP415476 | KP415475 |
| A/chicken/Dongguan/568/2014 | H7N9 | Avian | KP415486 | KP415488 | KP415489 | KP415487 | KP415490 | KP415485 | KP415484 | KP415483 |
| A/chicken/Dongguan/575/2014 | H7N9 | Avian | KP416502 | KP416504 | KP416505 | KP416503 | KP416506 | KP416501 | KP416500 | KP416499 |
| A/chicken/Dongguan/584/2014 | H7N9 | Avian | KP415494 | KP415496 | KP415497 | KP415495 | KP415498 | KP415493 | KP415492 | KP415491 |
| A/chicken/Dongguan/695/2014 | H7N9 | Avian | KP415531 | KP415533 | KP415534 | KP415532 | KP415535 | KP415530 | KP415529 | KP415528 |
| A/chicken/Dongguan/709/2014 | H7N9 | Avian | KP416007 | KP416009 | KP416010 | KP416008 | KP416011 | KP416006 | KP416005 | KP416004 |
| A/chicken/Dongguan/711/2014 | H7N9 | Avian | KP416015 | KP416017 | KP416018 | KP416016 | KP416019 | KP416014 | KP416013 | KP416012 |
| A/chicken/Dongguan/744/2014 | H7N9 | Avian | KP416023 | KP416025 | KP416026 | KP416024 | KP416027 | KP416022 | KP416021 | KP416020 |
| A/chicken/Dongguan/748/2014 | H7N9 | Avian | KP416031 | KP416033 | KP416034 | KP416032 | KP416035 | KP416030 | KP416029 | KP416028 |
| A/chicken/Dongguan/749/2014 | H7N9 | Avian | KP416039 | KP416041 | KP416042 | KP416040 | KP416043 | KP416038 | KP416037 | KP416036 |
| A/chicken/Dongguan/803/2014 | H7N9 | Avian | KP416072 | KP416074 | KP416075 | KP416073 | KP416076 | KP416071 | KP416070 | KP416069 |
| A/chicken/Dongguan/815/2014 | H7N9 | Avian | KP416080 | KP416082 | KP416083 | KP416081 | KP416084 | KP416079 | KP416078 | KP416077 |
| A/chicken/Dongguan/835/2014 | H7N9 | Avian | KP416088 | KP416090 | KP416091 | KP416089 | KP416092 | KP416087 | KP416086 | KP416085 |
| A/chicken/Dongguan/836/2014 | H7N9 | Avian | KP416096 | KP416098 | KP416099 | KP416097 | KP416100 | KP416095 | KP416094 | KP416093 |
| A/chicken/Dongguan/843/2014 | H7N9 | Avian | KP416104 | KP416106 | KP416107 | KP416105 | KP416108 | KP416103 | KP416102 | KP416101 |
| A/chicken/Dongguan/850/2014 | H7N9 | Avian | KP416112 | KP416114 | KP416115 | KP416113 | KP416116 | KP416111 | KP416110 | KP416109 |
| A/chicken/Dongguan/851/2014 | H7N9 | Avian | KP416120 | KP416122 | KP416123 | KP416121 | KP416124 | KP416119 | KP416118 | KP416117 |
| A/chicken/Dongguan/856/2014 | H7N9 | Avian | KP415539 | KP415541 | KP415542 | KP415540 | KP415543 | KP415538 | KP415537 | KP415536 |
| A/chicken/Dongguan/864/2014 | H7N9 | Avian | KP416128 | KP416130 | KP416131 | KP416129 | KP416132 | KP416127 | KP416126 | KP416125 |
| A/chicken/Dongguan/874/2014 | H7N9 | Avian | KP416136 | KP416138 | KP416139 | KP416137 | KP416140 | KP416135 | KP416134 | KP416133 |
| A/chicken/Dongguan/899/2014 | H7N9 | Avian | KP416144 | KP416146 | KP416147 | KP416145 | KP416148 | KP416143 | KP416142 | KP416141 |
| A/chicken/Dongguan/934/2014 | H7N9 | Avian | KP416152 | KP416154 | KP416155 | KP416153 | KP416156 | KP416151 | KP416150 | KP416149 |
| A/Chicken/Jilin/13188/2014 | H7N9 | avian | EPI_ISL_161671 | EPI_ISL_161671 | EPI_ISL_161671 | EPI_ISL_161671 | EPI_ISL_161671 | EPI_ISL_161671 | EPI_ISL_161671 | EPI_ISL_161671 |
| A/Chicken/Jilin/13199/2014 | H7N9 | avian | EPI_ISL_161672 | EPI_ISL_161672 | EPI_ISL_161672 | EPI_ISL_161672 | EPI_ISL_161672 | EPI_ISL_161672 | EPI_ISL_161672 | EPI_ISL_161672 |
| A/silkie_chicken/Dongguan/157/2014 | H7N9 | Avian | KP415420 | KP415422 | KP415423 | KP415421 | KP415424 | KP415419 | KP415418 | KP415417 |
| A/silkie_chicken/Dongguan/523/2014 | H7N9 | Avian | KP416494 | KP416496 | KP416497 | KP416495 | KP416498 | KP416493 | KP416492 | KP416491 |
| A/silkie_chicken/Dongguan/635/2014 | H7N9 | Avian | KP415502 | KP415504 | KP415505 | KP415503 | KP415506 | KP415501 | KP415500 | KP415499 |
| A/silkie_chicken/Dongguan/656/2014 | H7N9 | Avian | KP415510 | KP415512 | KP415513 | KP415511 | KP415514 | KP415509 | KP415508 | KP415507 |
| A/wild_bird/Korea/A14/2011 | H7N9 | Avian |  | JN244222 | JN244134 | JN244160 | JN244264 | JN244178 | JN244189 | JN244104 |
| A/wild_bird/Korea/A3/2011 | H7N9 | Avian |  | JN244223 | JN244135 | JN244158 | JN244265 | JN244179 | JN244190 | JN244105 |
| A/wild_bird/Korea/A9/2011 | H7N9 | Avian |  | JN244225 | JN244136 | JN244161 | JN244267 | JN244181 | JN244193 | JN244118 |
| A/chicken/Dongguan/1009/2014 | H7N9 | Avian | KP416230 | KP416232 | KP416233 | KP416231 | KP416234 | KP416229 | KP416228 | KP416227 |
| A/chicken/Dongguan/1022/2014 | H7N9 | Avian | KP416238 | KP416240 | KP416241 | KP416239 | KP416242 | KP416237 | KP416236 | KP416235 |
| A/chicken/Dongguan/1051/2014 | H7N9 | Avian | KP415618 | KP415620 | KP415621 | KP415619 | KP415622 | KP415617 | KP415616 | KP415615 |
| A/chicken/Dongguan/1057/2014 | H7N9 | Avian | KP415626 | KP415628 | KP415629 | KP415627 | KP415630 | KP415625 | KP415624 | KP415623 |
| A/chicken/Dongguan/1075/2014 | H7N9 | Avian | KP415634 | KP415636 | KP415637 | KP415635 | KP415638 | KP415633 | KP415632 | KP415631 |
| A/chicken/Dongguan/1091/2014 | H7N9 | Avian | KP415908 | KP415910 | KP415911 | KP415909 | KP415912 | KP415907 | KP415906 | KP415905 |
| A/chicken/Dongguan/1096/2014 | H7N9 | Avian | KP417438 | KP417440 | KP417441 | KP417439 | KP417442 | KP417437 | KP417436 | KP417435 |
| A/chicken/Dongguan/1100/2014 | H7N9 | Avian | KP416582 | KP416584 | KP416585 | KP416583 | KP416586 | KP416581 | KP416580 | KP416579 |
| A/chicken/Dongguan/1108/2014 | H7N9 | Avian | KP415916 | KP415918 | KP415919 | KP415917 | KP415920 | KP415915 | KP415914 | KP415913 |
| A/chicken/Dongguan/1124/2014 | H7N9 | Avian | KP416246 | KP416248 | KP416249 | KP416247 | KP416250 | KP416245 | KP416244 | KP416243 |
| A/chicken/Dongguan/1143/2014 | H7N9 | Avian | KP415642 | KP415644 | KP415645 | KP415643 | KP415646 | KP415641 | KP415640 | KP415639 |
| A/chicken/Dongguan/1177/2014 | H7N9 | Avian | KP415650 | KP415652 | KP415653 | KP415651 | KP415654 | KP415649 | KP415648 | KP415647 |
| A/chicken/Dongguan/1188/2014 | H7N9 | Avian | KP416263 | KP416265 | KP416266 | KP416264 | KP416267 | KP416262 | KP416261 | KP416260 |
| A/chicken/Dongguan/1230/2014 | H7N9 | Avian | KP416271 | KP416273 | KP416274 | KP416272 | KP416275 | KP416270 | KP416269 | KP416268 |
| A/chicken/Dongguan/1297/2014 | H7N9 | Avian | KP415682 | KP415684 | KP415685 | KP415683 | KP415686 | KP415681 | KP415680 | KP415679 |
| A/chicken/Dongguan/1303/2014 | H7N9 | Avian | KP416296 | KP416298 | KP416299 | KP416297 | KP416300 | KP416295 | KP416294 | KP416293 |
| A/chicken/Dongguan/1307/2014 | H7N9 | Avian | KP416304 | KP416306 | KP416307 | KP416305 | KP416308 | KP416303 | KP416302 | KP416301 |
| A/chicken/Dongguan/1312/2014 | H7N9 | Avian | KP415690 | KP415692 | KP415693 | KP415691 | KP415694 | KP415689 | KP415688 | KP415687 |
| A/chicken/Dongguan/1314/2014 | H7N9 | Avian | KP416312 | KP416314 | KP416315 | KP416313 | KP416316 | KP416311 | KP416310 | KP416309 |
| A/chicken/Dongguan/1318/2014 | H7N9 | Avian | KP416320 | KP416322 | KP416323 | KP416321 | KP416324 | KP416319 | KP416318 | KP416317 |
| A/chicken/Dongguan/1358/2014 | H7N9 | Avian | KP417446 | KP417448 | KP417449 | KP417447 | KP417450 | KP417445 | KP417444 | KP417443 |
| A/chicken/Dongguan/1374/2014 | H7N9 | Avian | KP415698 | KP415700 | KP415701 | KP415699 | KP415702 | KP415697 | KP415696 | KP415695 |
| A/chicken/Dongguan/1382/2014 | H7N9 | Avian | KP416349 | KP416351 | KP416352 | KP416350 | KP416353 | KP416348 | KP416347 | KP416346 |
| A/chicken/Dongguan/1393/2014 | H7N9 | Avian | KP416357 | KP416359 | KP416360 | KP416358 | KP416361 | KP416356 | KP416355 | KP416354 |
| A/chicken/Dongguan/1401/2014 | H7N9 | Avian | KP416365 | KP416367 | KP416368 | KP416366 | KP416369 | KP416364 | KP416363 | KP416362 |
| A/chicken/Dongguan/1421/2014 | H7N9 | Avian | KP416386 | KP416388 | KP416389 | KP416387 | KP416390 | KP416385 | KP416384 | KP416383 |
| A/chicken/Dongguan/1433/2014 | H7N9 | Avian | KP416394 | KP416396 | KP416397 | KP416395 | KP416398 | KP416393 | KP416392 | KP416391 |
| A/chicken/Dongguan/1456/2014 | H7N9 | Avian | KP415766 | KP415768 | KP415769 | KP415767 | KP415770 | KP415765 | KP415764 | KP415763 |
| A/chicken/Dongguan/1459/2014 | H7N9 | Avian | KP415774 | KP415776 | KP415777 | KP415775 | KP415778 | KP415773 | KP415772 | KP415771 |
| A/chicken/Dongguan/1494/2014 | H7N9 | Avian | KP415792 | KP415794 | KP415795 | KP415793 | KP415796 | KP415791 | KP415790 | KP415789 |
| A/chicken/Dongguan/1505/2014 | H7N9 | Avian | KP415800 | KP415802 | KP415803 | KP415801 | KP415804 | KP415799 | KP415798 | KP415797 |
| A/chicken/Dongguan/1506/2014 | H7N9 | Avian | KP415808 | KP415810 | KP415811 | KP415809 | KP415812 | KP415807 | KP415806 | KP415805 |
| A/chicken/Dongguan/1526/2014 | H7N9 | Avian | KP415858 | KP415860 | KP415861 | KP415859 | KP415862 | KP415857 | KP415856 | KP415855 |
| A/chicken/Dongguan/1527/2014 | H7N9 | Avian | KP416414 | KP416416 | KP416417 | KP416415 | KP416418 | KP416413 | KP416412 | KP416411 |
| A/chicken/Dongguan/1533/2014 | H7N9 | Avian | KP416422 | KP416424 | KP416425 | KP416423 | KP416426 | KP416421 | KP416420 | KP416419 |
| A/chicken/Dongguan/1548/2014 | H7N9 | Avian | KP416430 | KP416432 | KP416433 | KP416431 | KP416434 | KP416429 | KP416428 | KP416427 |
| A/chicken/Dongguan/1619/2014 | H7N9 | Avian | KP415866 | KP415868 | KP415869 | KP415867 | KP415870 | KP415865 | KP415864 | KP415863 |
| A/chicken/Dongguan/1666/2014 | H7N9 | Avian | KP415883 | KP415885 | KP415886 | KP415884 | KP415887 | KP415882 | KP415881 | KP415880 |
| A/chicken/Dongguan/1673/2014 | H7N9 | Avian | KP415900 | KP415902 | KP415903 | KP415901 | KP415904 | KP415899 | KP415898 | KP415897 |
| A/chicken/Dongguan/1690/2014 | H7N9 | Avian | KP416454 | KP416456 | KP416457 | KP416455 | KP416458 | KP416453 | KP416452 | KP416451 |
| A/chicken/Dongguan/1697/2014 | H7N9 | Avian | KP416475 | KP416477 | KP416478 | KP416476 | KP416479 | KP416474 | KP416473 | KP416472 |
| A/Chicken/Jilin/05/2014 | H7N9 | avian | EPI_ISL_161670 | EPI_ISL_161670 | EPI_ISL_161670 | EPI_ISL_161670 | EPI_ISL_161670 | EPI_ISL_161670 | EPI_ISL_161670 | EPI_ISL_161670 |
| A/Chicken/Jilin/13202/2014 | H7N9 | avian | EPI_ISL_161673 | EPI_ISL_161673 | EPI_ISL_161673 | EPI_ISL_161673 | EPI_ISL_161673 | EPI_ISL_161673 | EPI_ISL_161673 | EPI_ISL_161673 |
| A/Chicken/Jilin/13203/2014 | H7N9 | avian | EPI_ISL_161674 | EPI_ISL_161674 | EPI_ISL_161674 | EPI_ISL_161674 | EPI_ISL_161674 | EPI_ISL_161674 | EPI_ISL_161674 | EPI_ISL_161674 |
| A/Chicken/Jilin/13205/2014 | H7N9 | avian | EPI_ISL_161675 | EPI_ISL_161675 | EPI_ISL_161675 | EPI_ISL_161675 | EPI_ISL_161675 | EPI_ISL_161675 | EPI_ISL_161675 | EPI_ISL_161675 |
| A/silkie_chicken/Dongguan/1264/2014 | H7N9 | Avian | KP415658 | KP415660 | KP415661 | KP415659 | KP415662 | KP415657 | KP415656 | KP415655 |
| A/silkie_chicken/Dongguan/1268/2014 | H7N9 | Avian | KP415666 | KP415668 | KP415669 | KP415667 | KP415670 | KP415665 | KP415664 | KP415663 |
| A/silkie_chicken/Dongguan/1271/2014 | H7N9 | Avian | KP416288 | KP416290 | KP416291 | KP416289 | KP416292 | KP416287 | KP416286 | KP416285 |
| A/silkie_chicken/Dongguan/1274/2014 | H7N9 | Avian | KP415674 | KP415676 | KP415677 | KP415675 | KP415678 | KP415673 | KP415672 | KP415671 |
| A/silkie_chicken/Dongguan/1448/2014 | H7N9 | Avian | KP415732 | KP415734 | KP415735 | KP415733 | KP415736 | KP415731 | KP415730 | KP415729 |
| A/silkie_chicken/Dongguan/1450/2014 | H7N9 | Avian | KP415750 | KP415752 | KP415753 | KP415751 | KP415754 | KP415749 | KP415748 | KP415747 |
| A/silkie_chicken/Dongguan/1451/2014 | H7N9 | Avian | KP415758 | KP415760 | KP415761 | KP415759 | KP415762 | KP415757 | KP415756 | KP415755 |
| A/silkie_chicken/Dongguan/1516/2014 | H7N9 | Avian | KP415816 | KP415818 | KP415819 | KP415817 | KP415820 | KP415815 | KP415814 | KP415813 |
| A/silkie_chicken/Dongguan/1519/2014 | H7N9 | Avian | KP416590 | KP416592 | KP416593 | KP416591 | KP416594 | KP416589 | KP416588 | KP416587 |
| A/silkie_chicken/Dongguan/1641/2014 | H7N9 | Avian | KP416438 | KP416440 | KP416441 | KP416439 | KP416442 | KP416437 | KP416436 | KP416435 |
| A/silkie_chicken/Dongguan/953/2014 | H7N9 | Avian | KP416566 | KP416568 | KP416569 | KP416567 | KP416570 | KP416565 | KP416564 | KP416563 |
| A/silkie_chicken/Dongguan/963/2014 | H7N9 | Avian | KP415581 | KP415583 | KP415584 | KP415582 | KP415585 | KP415580 | KP415579 | KP415578 |
| A/silkie_chicken/Dongguan/967/2014 | H7N9 | Avian | KP416169 | KP416171 | KP416172 | KP416170 | KP416173 | KP416168 | KP416167 | KP416166 |
| A/silkie_chicken/Dongguan/969/2014 | H7N9 | Avian | KP416189 | KP416191 | KP416192 | KP416190 | KP416193 | KP416188 | KP416187 | KP416186 |
| A/silkie_chicken/Dongguan/979/2014 | H7N9 | Avian | KP416197 | KP416199 | KP416200 | KP416198 | KP416201 | KP416196 | KP416195 | KP416194 |
| A/silkie_chicken/Dongguan/981/2014 | H7N9 | Avian | KP416205 | KP416207 | KP416208 | KP416206 | KP416209 | KP416204 | KP416203 | KP416202 |
| A/silkie_chicken/Dongguan/986/2014 | H7N9 | Avian | KP416213 | KP416215 | KP416216 | KP416214 | KP416217 | KP416212 | KP416211 | KP416210 |
| A/silkie_chicken/Dongguan/988/2014 | H7N9 | Avian | KP415602 | KP415604 | KP415605 | KP415603 | KP415606 | KP415601 | KP415600 | KP415599 |
| A/silkie_chicken/Dongguan/991/2014 | H7N9 | Avian | KP415610 | KP415612 | KP415613 | KP415611 | KP415614 | KP415609 | KP415608 | KP415607 |
| A/silkie_chicken/Dongguan/997/2014 | H7N9 | Avian | KP416574 | KP416576 | KP416577 | KP416575 | KP416578 | KP416573 | KP416572 | KP416571 |
| A/chicken/Quzhou/2/2015 | H7N9 | Avian | KR351260 | KR351261 | KR351262 | KR351263 | KR351264 |  |  |  |
| A/silkie_chicken/Shantou/1406/2014 | H7N9 | Avian | KP416518 | KP416520 | KP416521 | KP416519 | KP416522 | KP416517 | KP416516 | KP416515 |
| A/chicken/Tennessee/17-007147-1/2017 | H7N9 | Avian | MF357740 | MF357742 | MF357743 | MF357741 | MF357744 | MF357739 | MF357738 | MF357737 |
| A/chicken/Tennessee/17-007147-2/2017 | H7N9 | Avian |  | KY818813 | KY818814 | KY818812 | KY818815 | KY818810 | KY818809 | KY818808 |
| A/chicken/Tennessee/17-007147-3/2017 | H7N9 | Avian | MF357748 | MF357750 | MF357751 | MF357749 | MF357752 | MF357747 | MF357746 | MF357745 |
| A/chicken/Tennessee/17-007147-4/2017 | H7N9 | Avian | MF357756 | MF357758 | MF357759 | MF357757 | MF357760 | MF357755 | MF357754 | MF357753 |
| A/chicken/Tennessee/17-007147-5/2017 | H7N9 | Avian | MF357764 | MF357766 | MF357767 | MF357765 | MF357768 | MF357763 | MF357762 | MF357761 |
| A/chicken/Tennessee/17-007147-6/2017 | H7N9 | Avian | MF357772 | MF357774 | MF357775 | MF357773 | MF357776 | MF357771 | MF357770 | MF357769 |
| A/chicken/Tennessee/17-007147-7/2017 | H7N9 | Avian | MF357780 | MF357782 | MF357783 | MF357781 | MF357784 | MF357779 | MF357778 | MF357777 |
| A/blue-winged_teal/Guatemala/CIP049-02/2008 | H7N9 | Avian |  |  | CY067681 | CY067679 | CY067682 | CY067677 | CY067676 | CY067675 |
| A/chicken/Shantou/1550/2014 | H7N9 | Avian | KP416526 | KP416528 | KP416529 | KP416527 | KP416530 | KP416525 | KP416524 | KP416523 |
| A/chicken/Shantou/1552/2014 | H7N9 | Avian | KP416534 | KP416536 | KP416537 | KP416535 | KP416538 | KP416533 | KP416532 | KP416531 |
| A/chicken/Shantou/1554/2014 | H7N9 | Avian | KP416542 | KP416544 | KP416545 | KP416543 | KP416546 | KP416541 | KP416540 | KP416539 |
| A/chicken/Shantou/1556/2014 | H7N9 | Avian | KP416550 | KP416552 | KP416553 | KP416551 | KP416554 | KP416549 | KP416548 | KP416547 |
| A/chicken/Tennessee/17-007429-3/2017 | H7N9 | Avian | MF357788 | MF357790 | MF357791 | MF357789 | MF357792 | MF357787 | MF357786 | MF357785 |
| A/chicken/Tennessee/17-007429-7/2017 | H7N9 | Avian | MF357796 | MF357798 | MF357799 | MF357797 | MF357800 | MF357795 | MF357794 | MF357793 |
| A/chicken/Tennessee/17-007431-3/2017 | H7N9 | Avian |  | KY818821 | KY818822 | KY818820 | KY818823 | KY818818 | KY818817 | KY818816 |
| A/guinea_fowl/Alabama/17-008272-2/2017 | H7N9 | Avian |  | KY818829 | KY818830 | KY818828 | KY818831 | KY818826 | KY818825 | KY818824 |
| A/chicken/Tennessee/17-008152-1/2017 | H7N9 | Avian | MF357804 | MF357806 | MF357807 | MF357805 | MF357808 | MF357803 | MF357802 | MF357801 |
| A/chicken/Tennessee/17-008279-2/2017 | H7N9 | Avian | MF357812 | MF357814 | MF357815 | MF357813 | MF357816 | MF357811 | MF357810 | MF357809 |
| A/chicken/Tennessee/17-008279-4/2017 | H7N9 | Avian |  | KY818837 | KY818838 | KY818836 | KY818839 | KY818834 | KY818833 | KY818832 |
| A/chicken/Tennessee/17-008279-5/2017 | H7N9 | Avian | MF357820 | MF357822 | MF357823 | MF357821 | MF357824 | MF357819 | MF357818 | MF357817 |
| A/chicken/Tennessee/17-008279-6/2017 | H7N9 | Avian | MF357828 | MF357830 | MF357831 | MF357829 | MF357832 | MF357827 | MF357826 | MF357825 |
| A/guinea_fowl/Alabama/17-008645-2/2017 | H7N9 | Avian | MF357844 | MF357846 | MF357847 | MF357845 | MF357848 | MF357843 | MF357842 | MF357841 |
| A/guinea_fowl/Alabama/17-008646-1/2017 | H7N9 | Avian | MF357852 | MF357854 | MF357855 | MF357853 | MF357856 | MF357851 | MF357850 | MF357849 |
| A/duck/Alabama/17-008643-2/2017 | H7N9 | Avian | MF357836 | MF357838 | MF357839 | MF357837 | MF357840 | MF357835 | MF357834 | MF357833 |
| A/chicken/Jiangxi/12200/2014 | H7N9 | Avian | KP417406 | KP417408 | KP417409 | KP417407 | KP417410 | KP417405 | KP417404 | KP417403 |
| A/chicken/Jiangxi/12201/2014 | H7N9 | Avian | KP417578 | KP417580 | KP417581 | KP417579 | KP417582 | KP417577 | KP417576 | KP417575 |
| A/chicken/Jiangxi/12206/2014 | H7N9 | Avian | KP417237 | KP417239 | KP417240 | KP417238 | KP417241 | KP417236 | KP417235 | KP417234 |
| A/chicken/Jiangxi/12208/2014 | H7N9 | Avian | KP417586 | KP417588 | KP417589 | KP417587 | KP417590 | KP417585 | KP417584 | KP417583 |
| A/chicken/Jiangxi/12210/2014 | H7N9 | Avian | KP417245 | KP417247 | KP417248 | KP417246 | KP417249 | KP417244 | KP417243 | KP417242 |
| A/chicken/Jiangxi/12216/2014 | H7N9 | Avian | KP417253 | KP417255 | KP417256 | KP417254 | KP417257 | KP417252 | KP417251 | KP417250 |
| A/chicken/Jiangxi/12217/2014 | H7N9 | Avian | KP417594 | KP417596 | KP417597 | KP417595 | KP417598 | KP417593 | KP417592 | KP417591 |
| A/chicken/Jiangxi/12219/2014 | H7N9 | Avian | KP417602 | KP417604 | KP417605 | KP417603 | KP417606 | KP417601 | KP417600 | KP417599 |
| A/chicken/Jiangxi/12221/2014 | H7N9 | Avian | KP417261 | KP417263 | KP417264 | KP417262 | KP417265 | KP417260 | KP417259 | KP417258 |
| A/chicken/Jiangxi/12222/2014 | H7N9 | Avian | KP417610 | KP417612 | KP417613 | KP417611 | KP417614 | KP417609 | KP417608 | KP417607 |
| A/chicken/Jiangxi/12223/2014 | H7N9 | Avian | KP417269 | KP417271 | KP417272 | KP417270 | KP417273 | KP417268 | KP417267 | KP417266 |
| A/chicken/Jiangxi/12232/2014 | H7N9 | Avian | KP417618 | KP417620 | KP417621 | KP417619 | KP417622 | KP417617 | KP417616 | KP417615 |
| A/chicken/Jiangxi/12239/2014 | H7N9 | Avian | KP417626 | KP417628 | KP417629 | KP417627 | KP417630 | KP417625 | KP417624 | KP417623 |
| A/chicken/Jiangxi/12240/2014 | H7N9 | Avian | KP417341 | KP417343 | KP417344 | KP417342 | KP417345 | KP417340 | KP417339 | KP417338 |
| A/chicken/Jiangxi/12243/2014 | H7N9 | Avian | KP417634 | KP417636 | KP417637 | KP417635 | KP417638 | KP417633 | KP417632 | KP417631 |
| A/chicken/Jiangxi/12245/2014 | H7N9 | Avian | KP417277 | KP417279 | KP417280 | KP417278 | KP417281 | KP417276 | KP417275 | KP417274 |
| A/chicken/Jiangxi/12247/2014 | H7N9 | Avian | KP417642 | KP417644 | KP417645 | KP417643 | KP417646 | KP417641 | KP417640 | KP417639 |
| A/chicken/Jiangxi/12248/2014 | H7N9 | Avian | KP417650 | KP417652 | KP417653 | KP417651 | KP417654 | KP417649 | KP417648 | KP417647 |
| A/chicken/Jiangxi/12249/2014 | H7N9 | Avian | KP417285 | KP417287 | KP417288 | KP417286 | KP417289 | KP417284 | KP417283 | KP417282 |
| A/chicken/Jiangxi/12251/2014 | H7N9 | Avian | KP417658 | KP417660 | KP417661 | KP417659 | KP417662 | KP417657 | KP417656 | KP417655 |
| A/chicken/Jiangxi/12254/2014 | H7N9 | Avian | KP417293 | KP417295 | KP417296 | KP417294 | KP417297 | KP417292 | KP417291 | KP417290 |
| A/chicken/Jiangxi/12256/2014 | H7N9 | Avian | KP417666 | KP417668 | KP417669 | KP417667 | KP417670 | KP417665 | KP417664 | KP417663 |
| A/chicken/Jiangxi/12260/2014 | H7N9 | Avian | KP417674 | KP417676 | KP417677 | KP417675 | KP417678 | KP417673 | KP417672 | KP417671 |
| A/chicken/Jiangxi/12261/2014 | H7N9 | Avian | KP417301 | KP417303 | KP417304 | KP417302 | KP417305 | KP417300 | KP417299 | KP417298 |
| A/chicken/Jiangxi/12264/2014 | H7N9 | Avian | KP417682 | KP417684 | KP417685 | KP417683 | KP417686 | KP417681 | KP417680 | KP417679 |
| A/chicken/Jiangxi/12265/2014 | H7N9 | Avian | KP417690 | KP417692 | KP417693 | KP417691 | KP417694 | KP417689 | KP417688 | KP417687 |
| A/chicken/Jiangxi/12273/2014 | H7N9 | Avian | KP417698 | KP417700 | KP417701 | KP417699 | KP417702 | KP417697 | KP417696 | KP417695 |
| A/chicken/Jiangxi/12274/2014 | H7N9 | Avian | KP417309 | KP417311 | KP417312 | KP417310 | KP417313 | KP417308 | KP417307 | KP417306 |
| A/chicken/Alabama/17-008901-1/2017 | H7N9 | Avian | MF357732 | MF357734 | MF357735 | MF357733 | MF357736 | MF357731 | MF357730 | MF357729 |
| A/chicken/Alabama/17-008899-10/2017 | H7N9 | Avian | MF357716 | MF357718 | MF357719 | MF357717 | MF357720 | MF357715 | MF357714 | MF357713 |
| A/chicken/Alabama/17-008899-11/2017 | H7N9 | Avian | MF357724 | MF357726 | MF357727 | MF357725 | MF357728 | MF357723 | MF357722 | MF357721 |
| A/wild_duck/Korea/CSM42-1/2011 | H7N9 | Avian |  | KC609808 | KC609948 | KC609920 | KC609976 | KC609892 | KC609864 | KC609836 |
| A/wild_duck/Korea/CSM42-34/2011 | H7N9 | Avian |  | KC609809 | KC609949 | KC609921 | KC609977 | KC609893 | KC609865 | KC609837 |
| A/wild_duck/Korea/MHC35-25/2011 | H7N9 | Avian |  | KC609804 | KC609944 | KC609916 | KC609972 | KC609888 | KC609860 | KC609832 |
| A/wild_duck/Korea/MHC35-41/2011 | H7N9 | Avian |  | KC609803 | KC609943 | KC609915 | KC609971 | KC609887 | KC609859 | KC609831 |
| A/wild_duck/Korea/MHC39-13/2011 | H7N9 | Avian |  | KC609805 | KC609945 | KC609917 | KC609973 | KC609889 | KC609861 | KC609833 |
| A/wild_duck/Korea/MHC39-26/2011 | H7N9 | Avian |  | KC609806 | KC609946 | KC609918 | KC609974 | KC609890 | KC609862 | KC609834 |
| A/chicken/Jiangxi/12768/2014 | H7N9 | Avian | KP417317 | KP417319 | KP417320 | KP417318 | KP417321 | KP417316 | KP417315 | KP417314 |
| A/silkie_chicken/Shantou/2050/2014 | H7N9 | Avian | KP418151 | KP418153 | KP418154 | KP418152 | KP418155 | KP418150 | KP418149 | KP418148 |
| A/silkie_chicken/Shantou/2054/2014 | H7N9 | Avian | KP418159 | KP418161 | KP418162 | KP418160 | KP418163 | KP418158 | KP418157 | KP418156 |
| A/silkie_chicken/Shantou/2056/2014 | H7N9 | Avian | KP418167 | KP418169 | KP418170 | KP418168 | KP418171 | KP418166 | KP418165 | KP418164 |
| A/blue-winged_teal/Louisiana/UGAI15-1367/2015 | H7N9 | Avian |  | KY013766 | KY013767 | KY013765 | KY013768 | KY013763 | KY013762 | KY013761 |
| A/blue-winged_teal/Louisiana/UGAI15-1692/2015 | H7N9 | Avian |  | KY013774 | KY013775 | KY013773 | KY013776 | KY013771 | KY013770 | KY013769 |
| A/chicken/Anhui-Chuzhou/01/2013 | H7N9 | avian | EPI_ISL_141161 | EPI_ISL_141161 | EPI_ISL_141161 | EPI_ISL_141161 | EPI_ISL_141161 | EPI_ISL_141161 | EPI_ISL_141161 | EPI_ISL_141161 |
| A/chicken/Jiangxi/13207/2014 | H7N9 | Avian | KP417325 | KP417327 | KP417328 | KP417326 | KP417329 | KP417324 | KP417323 | KP417322 |
| A/chicken/Jiangxi/13209/2014 | H7N9 | Avian | KP417333 | KP417335 | KP417336 | KP417334 | KP417337 | KP417332 | KP417331 | KP417330 |
| A/chicken/Jiangxi/13210/2014 | H7N9 | Avian | KP417706 | KP417708 | KP417709 | KP417707 | KP417710 | KP417705 | KP417704 | KP417703 |
| A/chicken/Jiangxi/13220/2014 | H7N9 | Avian | KP417349 | KP417351 | KP417352 | KP417350 | KP417353 | KP417348 | KP417347 | KP417346 |
| A/chicken/Jiangxi/13223/2014 | H7N9 | Avian | KP417357 | KP417359 | KP417360 | KP417358 | KP417361 | KP417356 | KP417355 | KP417354 |
| A/chicken/Jiangxi/13230/2014 | H7N9 | Avian | KP417365 | KP417367 | KP417368 | KP417366 | KP417369 | KP417364 | KP417363 | KP417362 |
| A/chicken/Jiangxi/13250/2014 | H7N9 | Avian | KP417382 | KP417384 | KP417385 | KP417383 | KP417386 | KP417381 | KP417380 | KP417379 |
| A/chicken/Jiangxi/13252/2014 | H7N9 | Avian | KP417714 | KP417716 | KP417717 | KP417715 | KP417718 | KP417713 | KP417712 | KP417711 |
| A/chicken/Jiangxi/13255/2014 | H7N9 | Avian | KP417390 | KP417392 | KP417393 | KP417391 | KP417394 | KP417389 | KP417388 | KP417387 |
| A/chicken/Jiangxi/13268/2014 | H7N9 | Avian | KP417398 | KP417400 | KP417401 | KP417399 | KP417402 | KP417397 | KP417396 | KP417395 |
| A/chicken/Jiangxi/13269/2014 | H7N9 | Avian | KP417722 | KP417724 | KP417725 | KP417723 | KP417726 | KP417721 | KP417720 | KP417719 |
| A/chicken/Jiangsu/K27/2013 | H7N9 | avian | EPI_ISL_139495 |  |  |  |  |  |  |  |
| A/chicken/Jiangsu/K89/2013 | H7N9 | avian | EPI_ISL_139496 |  |  |  |  |  |  |  |
| A/chicken/Suzhou/04020102/2013 | H7N9 | Avian | KT779567 | KT779580 | KT779624 | KT779612 | KT779628 | KT779605 | KT779603 | KT779596 |
| A/Pigeon/Shanghai/S1069/2013 | H7N9 | avian | EPI_ISL_138985 | EPI_ISL_138985 | EPI_ISL_138985 | EPI_ISL_138985 | EPI_ISL_138985 | EPI_ISL_138985 | EPI_ISL_138985 | EPI_ISL_138985 |
| A/Chicken/Shanghai/S1053/2013 | H7N9 | avian | EPI_ISL_138983 | EPI_ISL_138983 | EPI_ISL_138983 | EPI_ISL_138983 | EPI_ISL_138983 | EPI_ISL_138983 | EPI_ISL_138983 | EPI_ISL_138983 |
| A/chicken/Shanghai/S1055/2013 | H7N9 | avian | EPI_ISL_142928 | EPI_ISL_142928 | EPI_ISL_142928 | EPI_ISL_142928 | EPI_ISL_142928 | EPI_ISL_142928 | EPI_ISL_142928 | EPI_ISL_142928 |
| A/chicken/Shanghai/S1076/2013 | H7N9 | avian | EPI_ISL_142927 | EPI_ISL_142927 | EPI_ISL_142927 | EPI_ISL_142927 | EPI_ISL_142927 | EPI_ISL_142927 | EPI_ISL_142927 | EPI_ISL_142927 |
| A/chicken/Shanghai/S1077/2013 | H7N9 | avian | EPI_ISL_142926 | EPI_ISL_142926 | EPI_ISL_142926 | EPI_ISL_142926 | EPI_ISL_142926 | EPI_ISL_142926 | EPI_ISL_142926 | EPI_ISL_142926 |
| A/chicken/Shanghai/S1078/2013 | H7N9 | avian | EPI_ISL_142925 | EPI_ISL_142925 | EPI_ISL_142925 | EPI_ISL_142925 | EPI_ISL_142925 | EPI_ISL_142925 | EPI_ISL_142925 | EPI_ISL_142925 |
| A/chicken/Shanghai/S1079/2013 | H7N9 | avian | EPI_ISL_142924 | EPI_ISL_142924 | EPI_ISL_142924 | EPI_ISL_142924 | EPI_ISL_142924 | EPI_ISL_142924 | EPI_ISL_142924 | EPI_ISL_142924 |
| A/chicken/Shanghai/S1080/2013 | H7N9 | avian | EPI_ISL_142923 | EPI_ISL_142923 | EPI_ISL_142923 | EPI_ISL_142923 | EPI_ISL_142923 | EPI_ISL_142923 | EPI_ISL_142923 | EPI_ISL_142923 |
| A/chicken/Shanghai/S1358/2013 | H7N9 | avian | EPI_ISL_142922 | EPI_ISL_142922 | EPI_ISL_142922 | EPI_ISL_142922 | EPI_ISL_142922 | EPI_ISL_142922 | EPI_ISL_142922 | EPI_ISL_142922 |
| A/chicken/Shanghai/S1410/2013 | H7N9 | avian | EPI_ISL_142921 | EPI_ISL_142921 | EPI_ISL_142921 | EPI_ISL_142921 | EPI_ISL_142921 | EPI_ISL_142921 | EPI_ISL_142921 | EPI_ISL_142921 |
| A/chicken/Shanghai/S1413/2013 | H7N9 | avian | EPI_ISL_142920 | EPI_ISL_142920 | EPI_ISL_142920 | EPI_ISL_142920 | EPI_ISL_142920 | EPI_ISL_142920 | EPI_ISL_142920 | EPI_ISL_142920 |
| A/chicken/Wuxi/04030201/2013 | H7N9 | Avian | KT779566 | KT779579 | KT779622 | KT779613 | KT779633 | KT779606 | KT779600 | KT779595 |
| A/pigeon/Shanghai/S1421/2013 | H7N9 | avian | EPI_ISL_142903 | EPI_ISL_142903 | EPI_ISL_142903 | EPI_ISL_142903 | EPI_ISL_142903 | EPI_ISL_142903 | EPI_ISL_142903 | EPI_ISL_142903 |
| A/pigeon/Shanghai/S1423/2013 | H7N9 | avian | EPI_ISL_142902 | EPI_ISL_142902 | EPI_ISL_142902 | EPI_ISL_142902 | EPI_ISL_142902 | EPI_ISL_142902 | EPI_ISL_142902 | EPI_ISL_142902 |
| A/chicken/Wuxi/0405005/2013 | H7N9 | Avian | KT779570 | KT779585 | KT779621 | KT779611 | KT779629 | KT779607 | KT779599 | KT779592 |
| A/duck/Wuxi/0405006/2013 | H7N9 | Avian | KT779573 | KT779584 | KT779620 | KT779614 | KT779631 | KT779609 | KT779601 | KT779593 |
| A/pigeon/Wuxi/0405007/2013 | H7N9 | Avian | KT779569 | KT779582 | KT779619 |  | KT779630 | KT779608 | KT779602 | KT779594 |
| A/chicken/zhejiang/JX148/2014 | H7N9 | avian | EPI_ISL_235293 |  |  |  |  |  |  |  |
| A/goose/Jiangsu/1027/2013 | H7N9 | Avian | KP455978 | KP455980 | KP455981 | KP455979 | KP455982 | KP455977 | KP455976 | KP455975 |
| A/chicken/Jiangxi/13493/2014 | H7N9 | Avian | KP417730 | KP417732 | KP417733 | KP417731 | KP417734 | KP417729 | KP417728 | KP417727 |
| A/chicken/Jiangxi/13507/2014 | H7N9 | Avian | KP417748 | KP417750 | KP417751 | KP417749 | KP417752 | KP417747 | KP417746 | KP417745 |
| A/chicken/Jiangxi/13512/2014 | H7N9 | Avian | KP417756 | KP417758 | KP417759 | KP417757 | KP417760 | KP417755 | KP417754 | KP417753 |
| A/chicken/Jiangxi/13521/2014 | H7N9 | Avian | KP417784 | KP417786 | KP417787 | KP417785 | KP417788 | KP417783 | KP417782 | KP417781 |
| A/chicken/Jiangxi/13524/2014 | H7N9 | Avian | KP417792 | KP417794 | KP417795 | KP417793 | KP417796 | KP417791 | KP417790 | KP417789 |
| A/chicken/Jiangxi/13530/2014 | H7N9 | Avian | KP417829 | KP417831 | KP417832 | KP417830 | KP417833 | KP417828 | KP417827 | KP417826 |
| A/chicken/Jiangxi/13536/2014 | H7N9 | Avian | KP417837 | KP417839 | KP417840 | KP417838 | KP417841 | KP417836 | KP417835 | KP417834 |
| A/chicken/Jiangxi/13537/2014 | H7N9 | Avian | KP417845 | KP417847 | KP417848 | KP417846 | KP417849 | KP417844 | KP417843 | KP417842 |
| A/chicken/Jiangxi/13543/2014 | H7N9 | Avian | KP417862 | KP417864 | KP417865 | KP417863 | KP417866 | KP417861 | KP417860 | KP417859 |
| A/chicken/Jiangxi/13544/2014 | H7N9 | Avian | KP417870 | KP417872 | KP417873 | KP417871 | KP417874 | KP417869 | KP417868 | KP417867 |
| A/chicken/Jiangxi/13548/2014 | H7N9 | Avian | KP417878 | KP417880 | KP417881 | KP417879 | KP417882 | KP417877 | KP417876 | KP417875 |
| A/chicken/Jiangxi/13551/2014 | H7N9 | Avian | KP417886 | KP417888 | KP417889 | KP417887 | KP417890 | KP417885 | KP417884 | KP417883 |
| A/chicken/Jiangxi/13556/2014 | H7N9 | Avian | KP417905 | KP417907 | KP417908 | KP417906 | KP417909 | KP417904 | KP417903 | KP417902 |
| A/chicken/Jiangxi/13564/2014 | H7N9 | Avian | KP417913 | KP417915 | KP417916 | KP417914 | KP417917 | KP417912 | KP417911 | KP417910 |
| A/chicken/Jiangxi/12486/2013 | H7N9 | Avian | KP414064 | KP414066 | KP414067 | KP414065 | KP414068 | KP414063 | KP414062 | KP414061 |
| A/chicken/Jiangxi/12492/2013 | H7N9 | Avian | KP414072 | KP414074 | KP414075 | KP414073 | KP414076 | KP414071 | KP414070 | KP414069 |
| A/chicken/Jiangxi/12544/2013 | H7N9 | Avian | KP414090 | KP414092 | KP414093 | KP414091 | KP414094 | KP414089 | KP414088 | KP414087 |
| A/chicken/Jiangxi/12554/2013 | H7N9 | Avian | KP414098 | KP414100 | KP414101 | KP414099 | KP414102 | KP414097 | KP414096 | KP414095 |
| A/chicken/Jiangxi/12564/2013 | H7N9 | Avian | KP417041 | KP417043 | KP417044 | KP417042 | KP417045 | KP417040 | KP417039 | KP417038 |
| A/Chicken/Huzhou/C72/2013 | H7N9 | avian | EPI_ISL_144706 | EPI_ISL_144706 | EPI_ISL_144706 |  | EPI_ISL_144706 |  |  |  |
| A/Chicken/Nanjing/759/2013 | H7N9 | avian | EPI_ISL_159063 | EPI_ISL_159063 | EPI_ISL_159063 | EPI_ISL_159063 | EPI_ISL_159063 | EPI_ISL_159063 | EPI_ISL_159063 | EPI_ISL_159063 |
| A/Chicken/Nanjing/761/2013 | H7N9 | avian | EPI_ISL_159062 | EPI_ISL_159062 | EPI_ISL_159062 | EPI_ISL_159062 | EPI_ISL_159062 | EPI_ISL_159062 | EPI_ISL_159062 | EPI_ISL_159062 |
| A/Chicken/Suzhou/097-1/2013 | H7N9 | avian | EPI_ISL_159064 | EPI_ISL_159064 | EPI_ISL_159064 | EPI_ISL_159064 | EPI_ISL_159064 | EPI_ISL_159064 | EPI_ISL_159064 | EPI_ISL_159064 |
| A/guinea_fowl/Nebraska/17096/2011 | H7N9 | Avian |  |  | KF715109 | KF715107 | KF715110 | KF715105 | KF715104 | KF715103 |
| A/chicken/Hangzhou/48-1/2013 | H7N9 | avian | EPI_ISL_139906 | EPI_ISL_139906 | EPI_ISL_139906 | EPI_ISL_139906 | EPI_ISL_139906 | EPI_ISL_139906 | EPI_ISL_139906 | EPI_ISL_139906 |
| A/chicken/Hangzhou/50-1/2013 | H7N9 | avian | EPI_ISL_139907 | EPI_ISL_139907 | EPI_ISL_139907 | EPI_ISL_139907 | EPI_ISL_139907 | EPI_ISL_139907 | EPI_ISL_139907 | EPI_ISL_139907 |
| A/chicken/Zhejiang/SD019/2013 | H7N9 | avian | EPI_ISL_142918 | EPI_ISL_142918 | EPI_ISL_142918 | EPI_ISL_142918 | EPI_ISL_142918 | EPI_ISL_142918 | EPI_ISL_142918 | EPI_ISL_142918 |
| A/chicken/Zhejiang/SD033/2013 | H7N9 | avian | EPI_ISL_142917 | EPI_ISL_142917 | EPI_ISL_142917 | EPI_ISL_142917 | EPI_ISL_142917 | EPI_ISL_142917 | EPI_ISL_142917 | EPI_ISL_142917 |
| A/chicken/Jiangxi/13491/2014 | H7N9 | Avian | KP417454 | KP417456 | KP417457 | KP417455 | KP417458 | KP417453 | KP417452 | KP417451 |
| A/chicken/Jiangxi/13496/2014 | H7N9 | Avian | KP417462 | KP417464 | KP417465 | KP417463 | KP417466 | KP417461 | KP417460 | KP417459 |
| A/chicken/Jiangxi/13502/2014 | H7N9 | Avian | KP417470 | KP417472 | KP417473 | KP417471 | KP417474 | KP417469 | KP417468 | KP417467 |
| A/chicken/Jiangxi/13510/2014 | H7N9 | Avian | KP417478 | KP417480 | KP417481 | KP417479 | KP417482 | KP417477 | KP417476 | KP417475 |
| A/chicken/Jiangxi/13513/2014 | H7N9 | Avian | KP417486 | KP417488 | KP417489 | KP417487 | KP417490 | KP417485 | KP417484 | KP417483 |
| A/chicken/Jiangxi/13518/2014 | H7N9 | Avian | KP417494 | KP417496 | KP417497 | KP417495 | KP417498 | KP417493 | KP417492 | KP417491 |
| A/chicken/Jiangxi/13519/2014 | H7N9 | Avian | KP417502 | KP417504 | KP417505 | KP417503 | KP417506 | KP417501 | KP417500 | KP417499 |
| A/chicken/Jiangxi/13538/2014 | H7N9 | Avian | KP417521 | KP417523 | KP417524 | KP417522 | KP417525 | KP417520 | KP417519 | KP417518 |
| A/chicken/Jiangxi/13546/2014 | H7N9 | Avian | KP417529 | KP417531 | KP417532 | KP417530 | KP417533 | KP417528 | KP417527 | KP417526 |
| A/chicken/Jiangxi/13553/2014 | H7N9 | Avian | KP417537 | KP417539 | KP417540 | KP417538 | KP417541 | KP417536 | KP417535 | KP417534 |
| A/goose/Nebraska/17097/2011 | H7N9 | Avian |  |  | KF715116 | KF715115 | KF715117 | KF715113 | KF715112 | KF715111 |
| A/chicken/Jiangxi/14023/2014 | H7N9 | Avian | KP417921 | KP417923 | KP417924 | KP417922 | KP417925 | KP417920 | KP417919 | KP417918 |
| A/chicken/Jiangxi/14033/2014 | H7N9 | Avian | KP417929 | KP417931 | KP417932 | KP417930 | KP417933 | KP417928 | KP417927 | KP417926 |
| A/chicken/Guangdong/G1381/2014 | H7N9 | Avian | KP765998 |  |  |  |  |  |  |  |
| A/chicken/Guangdong/G1391/2014 | H7N9 | Avian | KP765999 | KP766111 |  |  |  |  |  |  |
| A/chicken/Guangdong/G1392/2014 | H7N9 | Avian | KP766000 | KP766112 |  |  |  |  |  |  |
| A/chicken/Guangdong/G1395/2014 | H7N9 | Avian |  | KP766113 |  |  |  |  |  |  |
| A/chicken/Guangdong/G1396/2014 | H7N9 | Avian | KP766001 | KP766114 |  |  |  |  |  |  |
| A/chicken/Guangdong/G1519/2014 | H7N9 | Avian | KP766002 | KP766115 |  |  |  |  |  |  |
| A/chicken/Guangdong/G1521/2014 | H7N9 | Avian | KP766003 | KP766116 |  |  |  |  |  |  |
| A/chicken/Guangdong/G1523/2014 | H7N9 | Avian | KP766004 | KP766117 |  |  |  |  |  |  |
| A/chicken/Guangdong/G1524/2014 | H7N9 | Avian | KP766005 | KP766118 |  |  |  |  |  |  |
| A/chicken/Jiangsu/S002/2013 | H7N9 | avian | EPI_ISL_142933 | EPI_ISL_142933 | EPI_ISL_142933 | EPI_ISL_142933 | EPI_ISL_142933 | EPI_ISL_142933 | EPI_ISL_142933 | EPI_ISL_142933 |
| A/chicken/Jiangsu/SC035/2013 | H7N9 | avian | EPI_ISL_142932 | EPI_ISL_142932 | EPI_ISL_142932 | EPI_ISL_142932 | EPI_ISL_142932 | EPI_ISL_142932 | EPI_ISL_142932 | EPI_ISL_142932 |
| A/chicken/Jiangsu/SC099/2013 | H7N9 | avian | EPI_ISL_142931 | EPI_ISL_142931 | EPI_ISL_142931 | EPI_ISL_142931 | EPI_ISL_142931 | EPI_ISL_142931 | EPI_ISL_142931 | EPI_ISL_142931 |
| A/chicken/Jiangsu/SC537/2013 | H7N9 | Avian | CY146940 | EPI_ISL_142930 | EPI_ISL_142930 | EPI_ISL_142930 | EPI_ISL_142930 | EPI_ISL_142930 | EPI_ISL_142930 | EPI_ISL_142930 |
| A/duck/Anhui/SC702/2013 | H7N9 | avian | EPI_ISL_142916 | EPI_ISL_142916 | EPI_ISL_142916 | EPI_ISL_142916 | EPI_ISL_142916 | EPI_ISL_142916 | EPI_ISL_142916 | EPI_ISL_142916 |
| A/duck/Zhejiang/SC410/2013 | H7N9 | avian | EPI_ISL_142915 | EPI_ISL_142915 | EPI_ISL_142915 | EPI_ISL_142915 | EPI_ISL_142915 | EPI_ISL_142915 | EPI_ISL_142915 | EPI_ISL_142915 |
| A/chicken/Guangdong/G1598/2014 | H7N9 | Avian | KP766006 | KP766119 |  |  |  |  |  |  |
| A/chicken/Shantou/2537/2014 | H7N9 | Avian | KP418038 | KP418040 | KP418041 | KP418039 | KP418042 | KP418037 | KP418036 | KP418035 |
| A/chicken/Shantou/2538/2014 | H7N9 | Avian | KP418046 | KP418048 | KP418049 | KP418047 | KP418050 | KP418045 | KP418044 | KP418043 |
| A/chicken/Shantou/2539/2014 | H7N9 | Avian | KP418054 | KP418056 | KP418057 | KP418055 | KP418058 | KP418053 | KP418052 | KP418051 |
| A/chicken/Shantou/2546/2014 | H7N9 | Avian | KP418062 | KP418064 | KP418065 | KP418063 | KP418066 | KP418061 | KP418060 | KP418059 |
| A/chicken/Shantou/2550/2014 | H7N9 | Avian | KP418070 | KP418072 | KP418073 | KP418071 | KP418074 | KP418069 | KP418068 | KP418067 |
| A/chicken/Shantou/2556/2014 | H7N9 | Avian | KP418078 | KP418080 | KP418081 | KP418079 | KP418082 | KP418077 | KP418076 | KP418075 |
| A/chicken/Shantou/2562/2014 | H7N9 | Avian | KP418086 | KP418088 | KP418089 | KP418087 | KP418090 | KP418085 | KP418084 | KP418083 |
| A/wild_pigeon/Jiangsu/SD001/2013 | H7N9 | avian | EPI_ISL_142901 | EPI_ISL_142901 | EPI_ISL_142901 | EPI_ISL_142901 | EPI_ISL_142901 | EPI_ISL_142901 | EPI_ISL_142901 | EPI_ISL_142901 |
| A/homing_pigeon/Jiangsu/SD184/2013 | H7N9 | avian | EPI_ISL_142904 | EPI_ISL_142904 | EPI_ISL_142904 | EPI_ISL_142904 | EPI_ISL_142904 | EPI_ISL_142904 | EPI_ISL_142904 | EPI_ISL_142904 |
| A/chicken/Jiangxi/14479/2014 | H7N9 | Avian | KP417937 | KP417939 | KP417940 | KP417938 | KP417941 | KP417936 | KP417935 | KP417934 |
| A/chicken/Jiangxi/14482/2014 | H7N9 | Avian | KP417945 | KP417947 | KP417948 | KP417946 | KP417949 | KP417944 | KP417943 | KP417942 |
| A/chicken/Jiangxi/14513/2014 | H7N9 | Avian | KP417982 | KP417984 | KP417985 | KP417983 | KP417986 | KP417981 | KP417980 | KP417979 |
| A/chicken/Jiangxi/14515/2014 | H7N9 | Avian | KP417990 | KP417992 | KP417993 | KP417991 | KP417994 | KP417989 | KP417988 | KP417987 |
| A/chicken/Jiangxi/14517/2014 | H7N9 | Avian | KP417998 | KP418000 | KP418001 | KP417999 | KP418002 | KP417997 | KP417996 | KP417995 |
| A/chicken/Jiangxi/14518/2014 | H7N9 | Avian | KP418006 | KP418008 | KP418009 | KP418007 | KP418010 | KP418005 | KP418004 | KP418003 |
| A/chicken/Jiangxi/14530/2014 | H7N9 | Avian | KP418014 | KP418016 | KP418017 | KP418015 | KP418018 | KP418013 | KP418012 | KP418011 |
| A/chicken/Jiangxi/14554/2014 | H7N9 | Avian | KP418030 | KP418032 | KP418033 | KP418031 | KP418034 | KP418029 | KP418028 | KP418027 |
| A/wild_duck/Mongolia/1-241/2008 | H7N9 | Avian |  |  | JN029688 | JN029687 | JN029689 | JN029685 | JN029684 | JN029683 |
| A/spot-billed_duck/Korea/447/2011 | H7N9 | Avian |  | JN244224 | JN244142 | JN244157 | JN244268 | JN244184 | JN244192 | JN244107 |
| A/chicken/Jiangsu/1021/2013 | H7N9 | Avian | KF938946 | KF938945 |  |  |  |  |  |  |
| A/chicken/Shanghai/017/2013 | H7N9 | Avian | KF542876 | KF542880 | KF542878 | KF542882 | KF542884 | KF542886 | KF542888 | KF542890 |
| A/chicken/Shanghai/019/2013 | H7N9 | Avian | KF542877 | KF542881 | KF542879 | KF542883 | KF542885 | KF542887 | KF542889 | KF542891 |
| A/chicken/Suzhou/040201H/2013 | H7N9 | Avian | KM879365 | KM879367 | KM879368 | KM879366 | KM879369 | KM879364 | KM879363 | KM879362 |
| A/chicken/Suzhou/040207GH05/2013 | H7N9 | Avian | KR905368 | KR905369 |  |  | KR905370 |  |  |  |
| A/chicken/Wuxi/04030201G/2013 | H7N9 | Avian | KR905377 | KR905378 |  |  | KR905379 |  |  |  |
| A/chicken/Wuxi/0405005G/2013 | H7N9 | Avian | KR905371 | KR905372 |  |  | KR905373 |  |  |  |
| A/chicken/Zhejiang/C481/2013 | H7N9 | Avian | KF042103 | KF042091 | KF042107 | KF042083 | KF042099 | KF042087 | KF042095 | KF042111 |
| A/chicken/Zhejiang/C483/2013 | H7N9 | Avian | KF042104 | KF042092 | KF042108 | KF042084 | KF042100 | KF042088 | KF042096 | KF042112 |
| A/chicken/Zhejiang/DTID-ZJU01/2013 | H7N9 | Avian | KC899669 | KC899671 | KC899672 | KC899670 | KC899673 | KC899668 | KC899667 | KC899666 |
| A/duck/Sunan/040802G/2013 | H7N9 | Avian | KM879373 | KM879375 | KM879376 | KM879374 | KM879377 | KM879372 | KM879371 | KM879370 |
| A/duck/Wuxi/04030204GH/2013 | H7N9 | Avian | KR905380 | KR905381 |  |  | KR905382 |  |  |  |
| A/duck/Wuxi/0405006G/2013 | H7N9 | Avian | KR905374 | KR905375 |  |  | KR905376 |  |  |  |
| A/pigeon/Wuxi/0405007G/2013 | H7N9 | Avian | KM879381 | KM879383 | KM879384 | KM879382 | KM879385 | KM879380 | KM879379 | KM879378 |
| A/pigeon/Zhejiang/P1/2013 | H7N9 | Avian | KF042101 | KF042089 | KF042105 | KF042081 | KF042097 | KF042085 | KF042093 | KF042109 |
| A/pigeon/Zhejiang/P2/2013 | H7N9 | Avian | KF042102 | KF042090 | KF042106 | KF042082 | KF042098 | KF042086 | KF042094 | KF042110 |
| A/chicken/Zhejiang/SD007/2013 | H7N9 | avian | EPI_ISL_142919 | EPI_ISL_142919 | EPI_ISL_142919 | EPI_ISL_142919 | EPI_ISL_142919 | EPI_ISL_142919 | EPI_ISL_142919 | EPI_ISL_142919 |
| A/chicken/Anhui/A1065/2014 | H7N9 | Avian | KP765952 | KP766083 |  |  |  |  |  |  |
| A/chicken/Anhui/A1070/2014 | H7N9 | Avian | KP765953 | KP766084 |  |  |  |  |  |  |
| A/chicken/Anhui/A1071/2014 | H7N9 | Avian | KP765954 | KP766085 |  |  |  |  |  |  |
| A/chicken/Anhui/A1072/2014 | H7N9 | Avian | KP765955 | KP766086 |  |  |  |  |  |  |
| A/chicken/Anhui/A1073/2014 | H7N9 | Avian | KP765956 | KP766087 |  |  |  |  |  |  |
| A/chicken/Anhui/A1091/2014 | H7N9 | Avian | KP765958 | KP766088 |  |  |  |  |  |  |
| A/chicken/Anhui/A1092/2014 | H7N9 | Avian | KP765959 | KP766089 |  |  |  |  |  |  |
| A/chicken/Anhui/A1098/2014 | H7N9 | Avian | KP765963 | KP766090 |  |  |  |  |  |  |
| A/chicken/Anhui/A1101/2014 | H7N9 | Avian | KP765965 | KP766091 |  |  |  |  |  |  |
| A/chicken/Anhui/A1103/2014 | H7N9 | Avian | KP765966 | KP766092 |  |  |  |  |  |  |
| A/chicken/Anhui/A1104/2014 | H7N9 | Avian | KP765967 | KP766093 |  |  |  |  |  |  |
| A/chicken/Anhui/A1106/2014 | H7N9 | Avian | KP765969 | KP766094 |  |  |  |  |  |  |
| A/chicken/Anhui/A1108/2014 | H7N9 | Avian | KP765970 | KP766095 |  |  |  |  |  |  |
| A/chicken/Anhui/A1109/2014 | H7N9 | Avian | KP765971 | KP766096 |  |  |  |  |  |  |
| A/chicken/Anhui/A1110/2014 | H7N9 | Avian | KP765972 | KP766097 |  |  |  |  |  |  |
| A/chicken/Anhui/A1113/2014 | H7N9 | Avian | KP765973 | KP766098 |  |  |  |  |  |  |
| A/chicken/Anhui/A1118/2014 | H7N9 | Avian | KP765978 | KP766099 |  |  |  |  |  |  |
| A/chicken/Anhui/A1119/2014 | H7N9 | Avian | KP765979 | KP766100 |  |  |  |  |  |  |
| A/chicken/Anhui/A1151/2014 | H7N9 | Avian | KP765980 | KP766101 |  |  |  |  |  |  |
| A/chicken/Anhui/A1155/2014 | H7N9 | Avian | KP765982 | KP766102 |  |  |  |  |  |  |
| A/chicken/Anhui/A1158/2014 | H7N9 | Avian | KP765984 | KP766103 |  |  |  |  |  |  |
| A/chicken/Anhui/A1160/2014 | H7N9 | Avian | KP765986 | KP766104 |  |  |  |  |  |  |
| A/chicken/Anhui/A1165/2014 | H7N9 | Avian | KP765989 | KP766105 |  |  |  |  |  |  |
| A/chicken/Anhui/A1166/2014 | H7N9 | Avian | KP765990 | KP766106 |  |  |  |  |  |  |
| A/chicken/Anhui/A1176/2014 | H7N9 | Avian | KP765992 | KP766107 |  |  |  |  |  |  |
| A/chicken/Anhui/A1392/2014 | H7N9 | Avian | KP765995 | KP766108 |  |  |  |  |  |  |
| A/chicken/Anhui/A1624/2014 | H7N9 | Avian | KP765996 | KP766109 |  |  |  |  |  |  |
| A/duck/Anhui/A1618/2014 | H7N9 | Avian | KP766037 | KP766135 |  |  |  |  |  |  |
| A/chicken/Heinan/ZZ01/2017 | H7N9 | Avian | MF319554 | MF319556 | MF319555 | MF319557 | MF319558 | MF319559 | MF319560 | MF319561 |
| A/chicken/Jiangxi/15044/2014 | H7N9 | Avian | KP418094 | KP418096 | KP418097 | KP418095 | KP418098 | KP418093 | KP418092 | KP418091 |
| A/goose/Jiangsu/J1111/2014 | H7N9 | Avian | KP766038 | KP766136 |  |  |  |  |  |  |
| A/chicken/Jiangsu/J1213/2014 | H7N9 | Avian | KP766007 | KP766120 |  |  |  |  |  |  |
| A/chicken/Jiangsu/J1216/2014 | H7N9 | Avian | KP766008 | KP766121 |  |  |  |  |  |  |
| A/chicken/Jiangsu/J1219/2014 | H7N9 | Avian | KP766010 | KP766122 |  |  |  |  |  |  |
| A/chicken/Jiangsu/J1220/2014 | H7N9 | Avian | KP766011 | KP766123 |  |  |  |  |  |  |
| A/chicken/Jiangsu/J1221/2014 | H7N9 | Avian | KP766012 | KP766124 |  |  |  |  |  |  |
| A/chicken/Jiangsu/J1224/2014 | H7N9 | Avian | KP766013 | KP766125 |  |  |  |  |  |  |
| A/chicken/Jiangsu/J1225/2014 | H7N9 | Avian | KP766014 | KP766126 |  |  |  |  |  |  |
| A/chicken/Jiangsu/J1229/2014 | H7N9 | Avian | KP766015 | KP766127 |  |  |  |  |  |  |
| A/chicken/Jiangsu/J1231/2014 | H7N9 | Avian | KP766016 | KP766128 |  |  |  |  |  |  |
| A/chicken/Jiangsu/WJ-14/2015 | H7N9 | Avian |  | MF276768 |  |  |  |  |  |  |
| A/chicken/Guangdong/SD641/2013 | H7N9 | avian | EPI_ISL_142934 | EPI_ISL_142934 | EPI_ISL_142934 | EPI_ISL_142934 | EPI_ISL_142934 | EPI_ISL_142934 | EPI_ISL_142934 | EPI_ISL_142934 |
| A/chicken/Jiangxi/SD001/2013 | H7N9 | avian | EPI_ISL_142929 | EPI_ISL_142929 | CY146951 | EPI_ISL_142929 | EPI_ISL_142929 | EPI_ISL_142929 | EPI_ISL_142929 | EPI_ISL_142929 |
| A/chicken/Guangdong/G1/2013 | H7N9 | Avian | KJ395947 | KJ395953 | KJ395989 | KJ395959 | KJ395965 | KJ395971 | KJ395977 | KJ395983 |
| A/chicken/Guangdong/G2/2013 | H7N9 | Avian | KJ395948 | KJ395954 | KJ395990 | KJ395960 | KJ395966 | KJ395972 | KJ395978 | KJ395984 |
| A/chicken/Guangdong/G3/2013 | H7N9 | Avian | KJ395949 | KJ395955 | KJ395991 | KJ395961 | KJ395967 | KJ395973 | KJ395979 | KJ395985 |
| A/chicken/Hangzhou/174/2013 | H7N9 | Avian | KP412424 | KP412426 | KP412427 | KP412425 | KP412428 | KP412423 | KP412422 | KP412421 |
| A/chicken/Jiangxi/15524/2014 | H7N9 | Avian | KP418133 | KP418135 | KP418136 | KP418134 | KP418137 | KP418132 | KP418131 | KP418130 |
| A/chicken/Shantou/3057/2014 | H7N9 | Avian | KP418175 | KP418177 | KP418178 | KP418176 | KP418179 | KP418174 | KP418173 | KP418172 |
| A/tree_sparrow/Shanghai/01/2013 | H7N9 | Avian | KJ508890 | KJ508892 | KF609530 | KF609528 | KF609531 | KJ508889 | KF609525 | KJ508887 |
| A/chicken/Shanghai/S1254/2014 | H7N9 | Avian | KP766031 | KP766129 |  |  |  |  |  |  |
| A/chicken/Shanghai/S1267/2014 | H7N9 | Avian |  | KP766130 |  |  |  |  |  |  |
| A/chicken/Shanghai/S1414/2014 | H7N9 | Avian | KP766034 | KP766131 |  |  |  |  |  |  |
| A/chicken/Shanghai/S1439/2014 | H7N9 | Avian | KP766035 | KP766132 |  |  |  |  |  |  |
| A/chicken/Shanghai/S1458/2014 | H7N9 | Avian |  | KP766133 |  |  |  |  |  |  |
| A/chicken/Guangdong/3135/2013 | H7N9 | Avian | KJ395945 | KJ395951 | KJ395987 | KJ395957 | KJ395963 | KJ395969 | KJ395975 | KJ395981 |
| A/chicken/Guangdong/3640/2013 | H7N9 | Avian | KJ395946 | KJ395952 | KJ395988 | KJ395958 | KJ395964 | KJ395970 | KJ395976 | KJ395982 |
| A/chicken/Guangdong/SD1/2013 | H7N9 | Avian | KJ395950 | KJ395956 | KJ395992 | KJ395962 | KJ395968 | KJ395974 | KJ395980 | KJ395986 |
| A/ruddy_turnstone/Delaware/AI00-1538/2000 | H7N9 | Avian |  |  | CY186011 | CY186013 | CY186014 | CY186015 | CY186016 | CY186017 |
| A/ruddy_turnstone/Delaware_Bay/220/1995 | H7N9 | Avian |  |  | CY127254 | CY127256 | CY127257 | CY127258 | CY127259 | CY127260 |
| A/emperor_goose/Alaska/44063-061/2006 | H7N9 | Avian |  |  | JX081204 | JX081087 | JX080984 | JX080933 | JX080858 | JX080849 |
| A/chicken/Jiangxi/18008/2014 | H7N9 | Avian | KP418254 | KP418256 | KP418257 | KP418255 | KP418258 | KP418253 | KP418252 | KP418251 |
| A/chicken/Netherlands/16007311-037041/2016 | H7N9 | avian |  |  | EPI_ISL_224581 | EPI_ISL_224581 | EPI_ISL_224581 | EPI_ISL_224581 | EPI_ISL_224581 | EPI_ISL_224581 |
| A/chicken/Jiangxi/18449/2014 | H7N9 | Avian | KP418281 | KP418283 | KP418284 | KP418282 | KP418285 | KP418280 | KP418279 | KP418278 |
| A/chicken/Jiangxi/18482/2014 | H7N9 | Avian | KP418354 | KP418356 | KP418357 | KP418355 | KP418358 | KP418353 | KP418352 | KP418351 |
| A/chicken/Jiangxi/18487/2014 | H7N9 | Avian | KP418386 | KP418388 | KP418389 | KP418387 | KP418390 | KP418385 | KP418384 | KP418383 |
| A/chicken/Jiangxi/18513/2014 | H7N9 | Avian | KP418436 | KP418438 | KP418439 | KP418437 | KP418440 | KP418435 | KP418434 | KP418433 |
| A/chicken/Jiangxi/18515/2014 | H7N9 | Avian | KP418444 | KP418446 | KP418447 | KP418445 | KP418448 | KP418443 | KP418442 | KP418441 |
| A/chicken/Zhangzhou/8585/2014 | H7N9 | Avian | KP418551 | KP418553 | KP418554 | KP418552 | KP418555 | KP418550 | KP418549 | KP418548 |
| A/chicken/Zhangzhou/8829/2014 | H7N9 | Avian | KP418559 | KP418561 | KP418562 | KP418560 | KP418563 | KP418558 | KP418557 | KP418556 |
| A/chicken/Shantou/4325/2014 | H7N9 | Avian | KP418183 | KP418185 | KP418186 | KP418184 | KP418187 | KP418182 | KP418181 | KP418180 |
| A/turkey/Minnesota/31900/2011 | H7N9 | Avian |  |  | KF715093 | KF715091 | KF715094 |  | KF715089 |  |
| A/Chicken/GD-ZS_124/H7N9/2014 | H7N9 | avian | EPI_ISL_198404 | EPI_ISL_198404 |  |  | EPI_ISL_198404 |  |  |  |
| A/Chicken/GD-ZS_712/H7N9/2014 | H7N9 | avian | EPI_ISL_198405 | EPI_ISL_198405 |  |  | EPI_ISL_198405 |  |  |  |
| A/Chicken/GD-ZS_714/H7N9/2014 | H7N9 | avian | EPI_ISL_198406 | EPI_ISL_198406 |  |  | EPI_ISL_198406 |  |  |  |
| A/Chicken/GD-ZS_718/H7N9/2014 | H7N9 | avian | EPI_ISL_198424 | EPI_ISL_198424 |  |  | EPI_ISL_198424 |  |  |  |
| A/Chicken/GD-ZS_720/H7N9/2014 | H7N9 | avian | EPI_ISL_198425 | EPI_ISL_198425 |  |  | EPI_ISL_198425 |  |  |  |
| A/Chicken/GD-ZS_727/H7N9/2014 | H7N9 | avian | EPI_ISL_198426 | EPI_ISL_198426 |  |  | EPI_ISL_198426 |  |  |  |
| A/turkey/Minnesota/32710/2011 | H7N9 | Avian |  |  | KF715101 | KF715099 | KF715102 | KF715097 | KF715096 | KF715095 |
| A/chicken/Guangzhou/1/2013 | H7N9 | Avian | KJ415825 |  |  |  |  |  |  |  |
| A/turkey/Minnesota/1/1988 | H7N9 | Avian |  |  | CY014787 | CY014789 | CY014790 | CY014791 | CY014792 | CY014793 |
| A/chicken/Shantou/4816/2014 | H7N9 | Avian | KP418200 | KP418202 | KP418203 | KP418201 | KP418204 | KP418199 | KP418198 | KP418197 |
| A/chicken/Shantou/4824/2014 | H7N9 | Avian | KP418208 | KP418210 | KP418211 | KP418209 | KP418212 | KP418207 | KP418206 | KP418205 |
| A/chicken/Shantou/4832/2014 | H7N9 | Avian | KP418216 | KP418218 | KP418219 | KP418217 | KP418220 | KP418215 | KP418214 | KP418213 |
| A/chicken/Shantou/4833/2014 | H7N9 | Avian | KP418224 | KP418226 | KP418227 | KP418225 | KP418228 | KP418223 | KP418222 | KP418221 |
| A/turkey/Minnesota/14135-2/2009 | H7N9 | Avian |  |  | KF715079 | KF715077 | KF715080 | KF715075 | KF715074 | KF715073 |
| A/turkey/Minnesota/14659/2009 | H7N9 | Avian |  |  | KF771192 | KF771190 | KF771193 | KF771188 | KF771187 | KF771186 |
| A/mallard/Alberta/177/2004 | H7N9 | Avian |  |  | KX827374 | KX827376 | KX827372 | KX827378 | KX851962 | KX827375 |
| A/turkey/Minnesota/15591/2009 | H7N9 | Avian |  |  | KF771200 | KF771198 | KF771201 |  | KF771195 |  |
| A/turkey/Minnesota/18421/2009 | H7N9 | Avian |  |  | KF771208 | KF771206 | KF771209 |  | KF771203 | KF771202 |
| A/mallard/Minnesota/AI09-3770/2009 | H7N9 | Avian |  |  | CY186003 | CY186005 | CY186006 | CY186007 | CY186008 | CY186009 |
| A/turkey/Minnesota/20400/2009 | H7N9 | Avian |  |  | KF771216 | KF771214 | KF771217 | KF771212 | KF771211 |  |
| A/chicken/Shaoxing/2417/2013 | H7N9 | Avian | KP413726 | KP413728 | KP413729 | KP413727 | KP413730 | KP413725 | KP413724 | KP413723 |
| A/chicken/Huzhou/3765/2013 | H7N9 | Avian | KP413734 | KP413736 | KP413737 | KP413735 | KP413738 | KP413733 | KP413732 | KP413731 |
| A/chicken/Huzhou/3791/2013 | H7N9 | Avian | KP413742 | KP413744 | KP413745 | KP413743 | KP413746 | KP413741 | KP413740 | KP413739 |
| A/chicken/Huzhou/3802/2013 | H7N9 | Avian | KP413750 | KP413752 | KP413753 | KP413751 | KP413754 | KP413749 | KP413748 | KP413747 |
| A/chicken/Huzhou/4045/2013 | H7N9 | Avian | KP413786 | KP413788 | KP413789 | KP413787 | KP413790 | KP413785 | KP413784 | KP413783 |
| A/chicken/Huzhou/4067/2013 | H7N9 | Avian | KP413794 | KP413796 | KP413797 | KP413795 | KP413798 | KP413793 | KP413792 | KP413791 |
| A/chicken/Huzhou/4073/2013 | H7N9 | Avian | KP413811 | KP413813 | KP413814 | KP413812 | KP413815 | KP413810 | KP413809 | KP413808 |
| A/chicken/Huzhou/4074/2013 | H7N9 | Avian | KP413819 | KP413821 | KP413822 | KP413820 | KP413823 | KP413818 | KP413817 | KP413816 |
| A/chicken/Huzhou/4076/2013 | H7N9 | Avian | KP413837 | KP413839 | KP413840 | KP413838 | KP413841 | KP413836 | KP413835 | KP413834 |
| A/chicken/Huzhou/4083/2013 | H7N9 | Avian | KP413864 | KP413866 | KP413867 | KP413865 | KP413868 | KP413863 | KP413862 | KP413861 |
| A/chicken/Huzhou/4141/2013 | H7N9 | Avian | KP413872 | KP413874 | KP413875 | KP413873 | KP413876 | KP413871 | KP413870 | KP413869 |
| A/chicken/Huzhou/4169/2013 | H7N9 | Avian | KP413880 | KP413882 | KP413883 | KP413881 | KP413884 | KP413879 | KP413878 | KP413877 |
| A/silkie_chicken/Huzhou/4213/2013 | H7N9 | Avian | KP413907 | KP413909 | KP413910 | KP413908 | KP413911 | KP413906 | KP413905 | KP413904 |
| A/chicken/Jiaxing/4490/2013 | H7N9 | Avian | KP413940 | KP413942 | KP413943 | KP413941 | KP413944 | KP413939 | KP413938 | KP413937 |
| A/chicken/Shaoxing/5086/2013 | H7N9 | Avian | KP413958 | KP413960 | KP413961 | KP413959 | KP413962 | KP413957 | KP413956 | KP413955 |
| A/chicken/Shaoxing/5087/2013 | H7N9 | Avian | KP413966 | KP413968 | KP413969 | KP413967 | KP413970 | KP413965 | KP413964 | KP413963 |
| A/chicken/Shaoxing/5136/2013 | H7N9 | Avian | KP413982 | KP413984 | KP413985 | KP413983 | KP413986 | KP413981 | KP413980 | KP413979 |
| A/chicken/Shaoxing/5146/2013 | H7N9 | Avian | KP413990 | KP413992 | KP413993 | KP413991 | KP413994 | KP413989 | KP413988 | KP413987 |
| A/chicken/Shaoxing/5186/2013 | H7N9 | Avian | KP413998 | KP414000 | KP414001 | KP413999 | KP414002 | KP413997 | KP413996 | KP413995 |
| A/chicken/Shaoxing/5201/2013 | H7N9 | Avian | KP414006 | KP414008 | KP414009 | KP414007 | KP414010 | KP414005 | KP414004 | KP414003 |
| A/chicken/Shaoxing/5224/2013 | H7N9 | Avian | KP414014 | KP414016 | KP414017 | KP414015 | KP414018 | KP414013 | KP414012 | KP414011 |
| A/chicken/Shaoxing/5227/2013 | H7N9 | Avian | KP414022 | KP414024 | KP414025 | KP414023 | KP414026 | KP414021 | KP414020 | KP414019 |
| A/silkie_chicken/Shaoxing/5130/2013 | H7N9 | Avian | KP413974 | KP413976 | KP413977 | KP413975 | KP413978 | KP413973 | KP413972 | KP413971 |
| A/silkie_chicken/Shaoxing/5235/2013 | H7N9 | Avian | KP414030 | KP414032 | KP414033 | KP414031 | KP414034 | KP414029 | KP414028 | KP414027 |
| A/chicken/Shaoxing/5479/2013 | H7N9 | Avian | KP414048 | KP414050 | KP414051 | KP414049 | KP414052 | KP414047 | KP414046 | KP414045 |
| A/chicken/Jiangsu/J3489/2014 | H7N9 | Avian | KP766045 | KP766137 |  |  |  |  |  |  |
| A/chicken/Jiangsu/J3494/2014 | H7N9 | Avian | KP766046 | KP766138 |  |  |  |  |  |  |
| A/chicken/Jiangsu/J3748/2014 | H7N9 | Avian |  | KP766139 |  |  |  |  |  |  |
| A/chicken/Jiangsu/J3899/2014 | H7N9 | Avian | KP766048 | KP766140 |  |  |  |  |  |  |
| A/chicken/Shanghai/S3061/2014 | H7N9 | Avian | KP766049 | KP766141 |  |  |  |  |  |  |
| A/chicken/Shanghai/S3064/2014 | H7N9 | Avian | KP766050 | KP766142 |  |  |  |  |  |  |
| A/chicken/Shanghai/S3065/2014 | H7N9 | Avian | KP766051 | KP766143 |  |  |  |  |  |  |
| A/chicken/Shanghai/S3075/2014 | H7N9 | Avian | KP766052 | KP766144 |  |  |  |  |  |  |
| A/chicken/Shanghai/S3084/2014 | H7N9 | Avian | KP766053 | KP766145 |  |  |  |  |  |  |
| A/chicken/Shanghai/S3085/2014 | H7N9 | Avian | KP766054 | KP766146 |  |  |  |  |  |  |
| A/chicken/Shanghai/S3086/2014 | H7N9 | Avian | KP766055 | KP766147 |  |  |  |  |  |  |
| A/chicken/Shanghai/S3087/2014 | H7N9 | Avian | KP766056 | KP766148 |  |  |  |  |  |  |
| A/chicken/Shanghai/S3088/2014 | H7N9 | Avian | KP766057 | KP766149 |  |  |  |  |  |  |
| A/chicken/Shanghai/S3090/2014 | H7N9 | Avian | KP766058 | KP766150 |  |  |  |  |  |  |
| A/chicken/Shanghai/S3140/2014 | H7N9 | Avian | KP766060 | KP766151 |  |  |  |  |  |  |
| A/chicken/Shanghai/S3167/2014 | H7N9 | Avian | KP766061 | KP766152 |  |  |  |  |  |  |
| A/chicken/Shanghai/S3169/2014 | H7N9 | Avian | KP766062 | KP766153 |  |  |  |  |  |  |
| A/chicken/Shanghai/S3180/2014 | H7N9 | Avian | KP766063 | KP766154 |  |  |  |  |  |  |
| A/chicken/Shanghai/S3301/2014 | H7N9 | Avian | KP766064 | KP766155 |  |  |  |  |  |  |
| A/chicken/Shanghai/S3306/2014 | H7N9 | Avian | KP766065 | KP766156 |  |  |  |  |  |  |
| A/chicken/Shanghai/S3307/2014 | H7N9 | Avian | KP766066 | KP766157 |  |  |  |  |  |  |
| A/chicken/Shanghai/S3308/2014 | H7N9 | Avian | KP766067 |  |  |  |  |  |  |  |
| A/chicken/Shanghai/S3309/2014 | H7N9 | Avian | KP766068 | KP766159 |  |  |  |  |  |  |
| A/chicken/Shanghai/S3313/2014 | H7N9 | Avian | KP766069 | KP766160 |  |  |  |  |  |  |
| A/chicken/Shanghai/S3322/2014 | H7N9 | Avian | KP766070 | KP766161 |  |  |  |  |  |  |
| A/chicken/Shanghai/S3325/2014 | H7N9 | Avian |  | KP766162 |  |  |  |  |  |  |
| A/chicken/Shanghai/S3326/2014 | H7N9 | Avian | KP766072 | KP766163 |  |  |  |  |  |  |
| A/chicken/Shanghai/S3328/2014 | H7N9 | Avian | KP766073 | KP766164 |  |  |  |  |  |  |
| A/chicken/Shanghai/S3330/2014 | H7N9 | Avian | KP766077 | KP766165 |  |  |  |  |  |  |
| A/chicken/Shanghai/S3630/2014 | H7N9 | Avian | KP766074 | KP766166 |  |  |  |  |  |  |
| A/chicken/Shanghai/S3880/2014 | H7N9 | Avian | KP766075 | KP766167 |  |  |  |  |  |  |
| A/duck/Jiangsu/J3619/2014 | H7N9 | Avian | KP766076 | KP766168 |  |  |  |  |  |  |
| A/Chicken/Guangdong/DG478/2014 | H7N9 | avian | EPI_ISL_176816 | EPI_ISL_176816 | EPI_ISL_176816 | EPI_ISL_176816 | EPI_ISL_176816 | EPI_ISL_176816 | EPI_ISL_176816 | EPI_ISL_176816 |
| A/Chicken/Guangdong/DG479/2014 | H7N9 | avian | EPI_ISL_176817 | EPI_ISL_176817 | EPI_ISL_176817 | EPI_ISL_176817 | EPI_ISL_176817 | EPI_ISL_176817 | EPI_ISL_176817 | EPI_ISL_176817 |
| A/Duck/Guangdong/DG527/2014 | H7N9 | avian | EPI_ISL_176818 | EPI_ISL_176818 | EPI_ISL_176818 | EPI_ISL_176818 | EPI_ISL_176818 | EPI_ISL_176818 | EPI_ISL_176818 | EPI_ISL_176818 |
| A/turkey/Minnesota/31676/2009 | H7N9 | Avian |  |  | KF715087 | KF715085 | KF715088 | KF715083 | KF715082 | KF715081 |
| A/Chicken/Guangdong/DG592/2014 | H7N9 | avian | EPI_ISL_176819 | EPI_ISL_176819 | EPI_ISL_176819 | EPI_ISL_176819 | EPI_ISL_176819 | EPI_ISL_176819 | EPI_ISL_176819 | EPI_ISL_176819 |
| A/chicken/Shenzhen/801/2013 | H7N9 | Avian | KP414194 | KP414196 | KP414197 | KP414195 | KP414198 | KP414193 | KP414192 | KP414191 |
| A/chicken/Shenzhen/898/2013 | H7N9 | Avian | KP413198 | KP413200 | KP413201 | KP413199 | KP413202 | KP413197 | KP413196 | KP413195 |
| A/silkie_chicken/Shenzhen/918/2013 | H7N9 | Avian | KP413206 | KP413208 | KP413209 | KP413207 | KP413210 | KP413205 | KP413204 | KP413203 |
| A/silkie_chicken/Shenzhen/919/2013 | H7N9 | Avian | KP413214 | KP413216 | KP413217 | KP413215 | KP413218 | KP413213 | KP413212 | KP413211 |
| A/chicken/Shenzhen/727/2013 | H7N9 | Avian | KP413166 | KP413168 | KP413169 | KP413167 | KP413170 | KP413165 | KP413164 | KP413163 |
| A/chicken/Shenzhen/742/2013 | H7N9 | Avian | KP413174 | KP413176 | KP413177 | KP413175 | KP413178 | KP413173 | KP413172 | KP413171 |
| A/chicken/Shenzhen/747/2013 | H7N9 | Avian | KP413182 | KP413184 | KP413185 | KP413183 | KP413186 | KP413181 | KP413180 | KP413179 |
| A/chicken/Shenzhen/749/2013 | H7N9 | Avian | KP413190 | KP413192 | KP413193 | KP413191 | KP413194 | KP413189 | KP413188 | KP413187 |
| A/duck/Bangladesh/26980/2015 | H7N9 | Avian |  | KY635689 | KY635666 | KY635442 | KY635659 | KY635690 | KY635811 | KY635459 |
| A/duck/Bangladesh/26992/2015 | H7N9 | Avian |  | KY635541 | KY635753 | KY635633 | KY635550 | KY635802 | KY635621 | KY635525 |
| A/duck/Bangladesh/27042/2015 | H7N9 | Avian |  | KY635739 | KY635509 | KY635733 | KY635772 | KY635827 | KY635641 | KY635516 |
| A/mallard/Sweden/1621/2002 | H7N9 | Avian |  |  | CY186293 | CY186295 | CY186296 | CY186297 | CY186298 | CY186299 |
| A/mallard/Sweden/91/2002 | H7N9 | Avian |  |  | KF695257 | KF695255 | KF695258 | KF695253 | KF695252 | KF695251 |
| A/chicken/Shenzhen/1665/2013 | H7N9 | Avian | KP413232 | KP413234 | KP413235 | KP413233 | KP413236 | KP413231 | KP413230 | KP413229 |
| A/Chicken/Guangdong/DG593/2014 | H7N9 | avian | EPI_ISL_176820 | EPI_ISL_176820 | EPI_ISL_176820 | EPI_ISL_176820 | EPI_ISL_176820 | EPI_ISL_176820 | EPI_ISL_176820 | EPI_ISL_176820 |
| A/chicken/Shenzhen/2110/2013 | H7N9 | Avian | KP413240 | KP413242 | KP413243 | KP413241 | KP413244 | KP413239 | KP413238 | KP413237 |
| A/chicken/Shenzhen/2201/2013 | H7N9 | Avian | KP413273 | KP413275 | KP413276 | KP413274 | KP413277 | KP413272 | KP413271 | KP413270 |
| A/chicken/Shenzhen/2293/2013 | H7N9 | Avian | KP413281 | KP413283 | KP413284 | KP413282 | KP413285 | KP413280 | KP413279 | KP413278 |
| A/silkie_chicken/Shenzhen/2134/2013 | H7N9 | Avian | KP413257 | KP413259 | KP413260 | KP413258 | KP413261 | KP413256 | KP413255 | KP413254 |
| A/silkie_chicken/Shenzhen/2139/2013 | H7N9 | Avian | KP413265 | KP413267 | KP413268 | KP413266 | KP413269 | KP413264 | KP413263 | KP413262 |
| A/chicken/Dongguan/2912/2013 | H7N9 | Avian | KP413289 | KP413291 | KP413292 | KP413290 | KP413293 | KP413288 | KP413287 | KP413286 |
| A/chicken/Dongguan/3112/2013 | H7N9 | Avian | KP413305 | KP413307 | KP413308 | KP413306 | KP413309 | KP413304 | KP413303 | KP413302 |
| A/chicken/Dongguan/3141/2013 | H7N9 | Avian | KP413313 | KP413315 | KP413316 | KP413314 | KP413317 | KP413312 | KP413311 | KP413310 |
| A/chicken/Dongguan/3145/2013 | H7N9 | Avian | KP413321 | KP413323 | KP413324 | KP413322 | KP413325 | KP413320 | KP413319 | KP413318 |
| A/chicken/Dongguan/3146/2013 | H7N9 | Avian | KP413329 | KP413331 | KP413332 | KP413330 | KP413333 | KP413328 | KP413327 | KP413326 |
| A/chicken/Dongguan/3219/2013 | H7N9 | Avian | KP413345 | KP413347 | KP413348 | KP413346 | KP413349 | KP413344 | KP413343 | KP413342 |
| A/silkie_chicken/Dongguan/3049/2013 | H7N9 | Avian | KP413297 | KP413299 | KP413300 | KP413298 | KP413301 | KP413296 | KP413295 | KP413294 |
| A/silkie_chicken/Dongguan/3166/2013 | H7N9 | Avian | KP413337 | KP413339 | KP413340 | KP413338 | KP413341 | KP413336 | KP413335 | KP413334 |
| A/silkie_chicken/Dongguan/3275/2013 | H7N9 | Avian | KP413371 | KP413373 | KP413374 | KP413372 | KP413375 | KP413370 | KP413369 | KP413368 |
| A/silkie_chicken/Dongguan/3281/2013 | H7N9 | Avian | KP413379 | KP413381 | KP413382 | KP413380 | KP413383 | KP413378 | KP413377 | KP413376 |
| A/silkie_chicken/Dongguan/3284/2013 | H7N9 | Avian | KP413387 | KP413389 | KP413390 | KP413388 | KP413391 | KP413386 | KP413385 | KP413384 |
| A/chicken/Dongguan/3418/2013 | H7N9 | Avian | KP413395 | KP413397 | KP413398 | KP413396 | KP413399 | KP413394 | KP413393 | KP413392 |
| A/chicken/Dongguan/3438/2013 | H7N9 | Avian | KP413403 | KP413405 | KP413406 | KP413404 | KP413407 | KP413402 | KP413401 | KP413400 |
| A/chicken/Dongguan/3464/2013 | H7N9 | Avian | KP413411 | KP413413 | KP413414 | KP413412 | KP413415 | KP413410 | KP413409 | KP413408 |
| A/chicken/Dongguan/3487/2013 | H7N9 | Avian | KP413419 | KP413421 | KP413422 | KP413420 | KP413423 | KP413418 | KP413417 | KP413416 |
| A/chicken/Dongguan/3488/2013 | H7N9 | Avian | KP413427 | KP413429 | KP413430 | KP413428 | KP413431 | KP413426 | KP413425 | KP413424 |
| A/chicken/Dongguan/3491/2013 | H7N9 | Avian | KP413435 | KP413437 | KP413438 | KP413436 | KP413439 | KP413434 | KP413433 | KP413432 |
| A/chicken/Dongguan/3544/2013 | H7N9 | Avian | KP413483 | KP413485 | KP413486 | KP413484 | KP413487 | KP413482 | KP413481 | KP413480 |
| A/chicken/Dongguan/3563/2013 | H7N9 | Avian | KP413491 | KP413493 | KP413494 | KP413492 | KP413495 | KP413490 | KP413489 | KP413488 |
| A/chicken/Dongguan/3582/2013 | H7N9 | Avian | KP413499 | KP413501 | KP413502 | KP413500 | KP413503 | KP413498 | KP413497 | KP413496 |
| A/chicken/Dongguan/3894/2013 | H7N9 | Avian | KP413563 | KP413565 | KP413566 | KP413564 | KP413567 | KP413562 | KP413561 | KP413560 |
| A/chicken/Dongguan/3917/2013 | H7N9 | Avian | KP413571 | KP413573 | KP413574 | KP413572 | KP413575 | KP413570 | KP413569 | KP413568 |
| A/chicken/Dongguan/3935/2013 | H7N9 | Avian | KP413579 | KP413581 | KP413582 | KP413580 | KP413583 | KP413578 | KP413577 | KP413576 |
| A/chicken/Dongguan/3945/2013 | H7N9 | Avian | KP413587 | KP413589 | KP413590 | KP413588 | KP413591 | KP413586 | KP413585 | KP413584 |
| A/chicken/Dongguan/3972/2013 | H7N9 | Avian | KP413595 | KP413597 | KP413598 | KP413596 | KP413599 | KP413594 | KP413593 | KP413592 |
| A/chicken/Dongguan/4037/2013 | H7N9 | Avian | KP413619 | KP413621 | KP413622 | KP413620 | KP413623 | KP413618 | KP413617 | KP413616 |
| A/chicken/Dongguan/4040/2013 | H7N9 | Avian | KP414219 | KP414221 | KP414222 | KP414220 | KP414223 | KP414218 | KP414217 | KP414216 |
| A/chicken/Dongguan/4048/2013 | H7N9 | Avian | KP413627 | KP413629 | KP413630 | KP413628 | KP413631 | KP413626 | KP413625 | KP413624 |
| A/chicken/Dongguan/4063/2013 | H7N9 | Avian | KP413635 | KP413637 | KP413638 | KP413636 | KP413639 | KP413634 | KP413633 | KP413632 |
| A/chicken/Dongguan/4064/2013 | H7N9 | Avian | KP414211 | KP414213 | KP414214 | KP414212 | KP414215 | KP414210 | KP414209 | KP414208 |
| A/chicken/Dongguan/4094/2013 | H7N9 | Avian | KP413643 | KP413645 | KP413646 | KP413644 | KP413647 | KP413642 | KP413641 | KP413640 |
| A/chicken/Dongguan/4102/2013 | H7N9 | Avian | KP413651 | KP413653 | KP413654 | KP413652 | KP413655 | KP413650 | KP413649 | KP413648 |
| A/chicken/Dongguan/4114/2013 | H7N9 | Avian | KP413659 | KP413661 | KP413662 | KP413660 | KP413663 | KP413658 | KP413657 | KP413656 |
| A/chicken/Dongguan/4119/2013 | H7N9 | Avian | KP414186 | KP414188 | KP414189 | KP414187 | KP414190 | KP414185 | KP414184 | KP414183 |
| A/chicken/Dongguan/4195/2013 | H7N9 | Avian | KP413700 | KP413702 | KP413703 | KP413701 | KP413704 | KP413699 | KP413698 | KP413697 |
| A/chicken/Dongguan/4251/2013 | H7N9 | Avian | KP413708 | KP413710 | KP413711 | KP413709 | KP413712 | KP413707 | KP413706 | KP413705 |
| A/chicken/Shenzhen/3733/2013 | H7N9 | Avian | KP413523 | KP413525 | KP413526 | KP413524 | KP413527 | KP413522 | KP413521 | KP413520 |
| A/chicken/Shenzhen/3734/2013 | H7N9 | Avian | KP413531 | KP413533 | KP413534 | KP413532 | KP413535 | KP413530 | KP413529 | KP413528 |
| A/chicken/Shenzhen/3780/2013 | H7N9 | Avian | KP413539 | KP413541 | KP413542 | KP413540 | KP413543 | KP413538 | KP413537 | KP413536 |
| A/silkie_chicken/Dongguan/3520/2013 | H7N9 | Avian | KP413443 | KP413445 | KP413446 | KP413444 | KP413447 | KP413442 | KP413441 | KP413440 |
| A/silkie_chicken/Dongguan/3522/2013 | H7N9 | Avian | KP413451 | KP413453 | KP413454 | KP413452 | KP413455 | KP413450 | KP413449 | KP413448 |
| A/silkie_chicken/Dongguan/3525/2013 | H7N9 | Avian | KP413459 | KP413461 | KP413462 | KP413460 | KP413463 | KP413458 | KP413457 | KP413456 |
| A/silkie_chicken/Dongguan/3526/2013 | H7N9 | Avian | KP413467 | KP413469 | KP413470 | KP413468 | KP413471 | KP413466 | KP413465 | KP413464 |
| A/silkie_chicken/Dongguan/3528/2013 | H7N9 | Avian | KP413475 | KP413477 | KP413478 | KP413476 | KP413479 | KP413474 | KP413473 | KP413472 |
| A/silkie_chicken/Dongguan/3605/2013 | H7N9 | Avian | KP413507 | KP413509 | KP413510 | KP413508 | KP413511 | KP413506 | KP413505 | KP413504 |
| A/silkie_chicken/Dongguan/3606/2013 | H7N9 | Avian | KP413515 | KP413517 | KP413518 | KP413516 | KP413519 | KP413514 | KP413513 | KP413512 |
| A/silkie_chicken/Dongguan/3980/2013 | H7N9 | Avian | KP413603 | KP413605 | KP413606 | KP413604 | KP413607 | KP413602 | KP413601 | KP413600 |
| A/silkie_chicken/Dongguan/3990/2013 | H7N9 | Avian | KP413611 | KP413613 | KP413614 | KP413612 | KP413615 | KP413610 | KP413609 | KP413608 |
| A/silkie_chicken/Dongguan/4126/2013 | H7N9 | Avian | KP413667 | KP413669 | KP413670 | KP413668 | KP413671 | KP413666 | KP413665 | KP413664 |
| A/silkie_chicken/Dongguan/4127/2013 | H7N9 | Avian | KP413675 | KP413677 | KP413678 | KP413676 | KP413679 | KP413674 | KP413673 | KP413672 |
| A/silkie_chicken/Dongguan/4129/2013 | H7N9 | Avian | KP413692 | KP413694 | KP413695 | KP413693 | KP413696 | KP413691 | KP413690 | KP413689 |
| A/silkie_chicken/Shenzhen/3781/2013 | H7N9 | Avian | KP413547 | KP413549 | KP413550 | KP413548 | KP413551 | KP413546 | KP413545 | KP413544 |
| A/silkie_chicken/Shenzhen/3782/2013 | H7N9 | Avian | KP413555 | KP413557 | KP413558 | KP413556 | KP413559 | KP413554 | KP413553 | KP413552 |
| A/wild_duck/Korea/SH20-27/2008 | H7N9 | Avian |  |  | KC609935 | KC609907 | KC609963 | KC609879 | KC609851 | KC609823 |
| A/wild_duck/Korea/SH19-27/2010 | H7N9 | Avian |  | KC609799 | KC609939 | KC609911 | KC609967 | KC609883 | KC609855 | KC609827 |
| A/wild_duck/Korea/SH19-44/2010 | H7N9 | Avian |  | KC609800 | KC609940 | KC609912 | KC609968 | KC609884 | KC609856 | KC609828 |
| A/wild_duck/Korea/SH19-47/2010 | H7N9 | Avian |  | KC609801 | KC609941 | KC609913 | KC609969 | KC609885 | KC609857 | KC609829 |
| A/chicken/Zhejiang/DTID-ZJU06/2013 | H7N9 | Avian | KM879322 | KM879324 | KM879325 | KM879323 | KM879326 | KM879321 | KM879320 | KM879319 |
| A/Chicken/GD-ZS_123/H7N9/2014 | H7N9 | avian |  | EPI_ISL_198403 |  |  |  |  |  |  |
| A/teal/Ukraine/12177-NAMRU3/2005 | H7N9 | avian |  |  |  |  |  | EPI_ISL_120209 | EPI_ISL_120209 | EPI_ISL_120209 |
| A/environment/Korea/MA-410/2016 | H7N9 | environment |  | KY067458 |  |  |  |  |  |  |
| A/environment/Guangdong/22351/2015 | H7N9 | environment | EPI_ISL_192490 | EPI_ISL_192490 | EPI_ISL_192490 | EPI_ISL_192490 | EPI_ISL_192490 | EPI_ISL_192490 | EPI_ISL_192490 | EPI_ISL_192490 |
| A/environment/Zhejiang/07819/2014 | H7N9 | environment | EPI_ISL_192433 | EPI_ISL_192433 | EPI_ISL_192433 | EPI_ISL_192433 | EPI_ISL_192433 | EPI_ISL_192433 | EPI_ISL_192433 | EPI_ISL_192433 |
| A/environment/Guangdong/21286/2015 | H7N9 | environment | EPI_ISL_192481 | EPI_ISL_192481 | EPI_ISL_192481 | EPI_ISL_192481 | EPI_ISL_192481 | EPI_ISL_192481 | EPI_ISL_192481 | EPI_ISL_192481 |
| A/environment/Guangdong/DG104/2015 | H7N9 | environment | EPI_ISL_176830 | EPI_ISL_176830 | EPI_ISL_176830 | EPI_ISL_176830 | EPI_ISL_176830 | EPI_ISL_176830 | EPI_ISL_176830 | EPI_ISL_176830 |
| A/environment/Shandong-Linyi/EV01/2015 | H7N9 | environment | EPI_ISL_192496 | EPI_ISL_192496 | EPI_ISL_192496 | EPI_ISL_192496 | EPI_ISL_192496 | EPI_ISL_192496 | EPI_ISL_192496 | EPI_ISL_192496 |
| A/environment/Guangdong/21123/2015 | H7N9 | environment | EPI_ISL_192482 | EPI_ISL_192482 | EPI_ISL_192482 | EPI_ISL_192482 | EPI_ISL_192482 | EPI_ISL_192482 | EPI_ISL_192482 | EPI_ISL_192482 |
| A/environment/Hunan/07626/2015 | H7N9 | environment | EPI_ISL_192489 | EPI_ISL_192489 | EPI_ISL_192489 | EPI_ISL_192489 | EPI_ISL_192489 | EPI_ISL_192489 | EPI_ISL_192489 | EPI_ISL_192489 |
| A/environment/Shanghai/PD-JZ-01/2014 | H7N9 | environment | KJ549794 | KJ549796 | KJ549797 | KJ549795 | KJ549798 | KJ549793 | KJ549792 | KJ549791 |
| A/environment/Hangzhou/249/2014 | H7N9 | environment | EPI_ISL_169590 |  |  |  |  |  |  |  |
| A/environment/Guangzhou/1/2014 | H7N9 | environment | KJ415824 |  |  |  |  |  |  |  |
| A/environment/Hunan/07836/2014 | H7N9 | environment | EPI_ISL_192437 | EPI_ISL_192437 | EPI_ISL_192437 | EPI_ISL_192437 | EPI_ISL_192437 | EPI_ISL_192437 | EPI_ISL_192437 | EPI_ISL_192437 |
| A/environment/Guangdong/21275/2015 | H7N9 | environment | EPI_ISL_192480 | EPI_ISL_192480 | EPI_ISL_192480 | EPI_ISL_192480 | EPI_ISL_192480 | EPI_ISL_192480 | EPI_ISL_192480 | EPI_ISL_192480 |
| A/environment/GD-ST_101/H7N9/2014-2 | H7N9 | environment | EPI_ISL_198427 | EPI_ISL_198427 | EPI_ISL_198427 | EPI_ISL_198427 | EPI_ISL_198427 |  |  |  |
| A/environment/Hunan/20986/2015 | H7N9 | environment | EPI_ISL_192483 | EPI_ISL_192483 | EPI_ISL_192483 | EPI_ISL_192483 | EPI_ISL_192483 | EPI_ISL_192483 | EPI_ISL_192483 | EPI_ISL_192483 |
| A/environment/Guangdong/21231/2015 | H7N9 | environment | EPI_ISL_192479 | EPI_ISL_192479 | EPI_ISL_192479 | EPI_ISL_192479 | EPI_ISL_192479 | EPI_ISL_192479 | EPI_ISL_192479 | EPI_ISL_192479 |
| A/environment/Korea/W410/2011 | H7N9 | environment |  | KX297785 | KX297769 | KX297801 | KX297817 | KX297831 | KX297845 | KX297859 |
| A/environment/ChongqingHC/2-20/2017 | H7N9 | environment | EPI_ISL_258245 | EPI_ISL_258245 |  |  |  |  |  |  |
| A/environment/Guangzhou/XN07390/2014 | H7N9 | environment | KP326319 |  |  |  |  |  |  |  |
| A/environment/Guangzhou/XN07874/2014 | H7N9 | environment | KP326320 |  |  |  |  |  |  |  |
| A/environment/Shandong/ZZ001/2014 | H7N9 | environment | EPI_ISL_192353 | EPI_ISL_192353 | EPI_ISL_192353 | EPI_ISL_192353 | EPI_ISL_192353 | EPI_ISL_192353 | EPI_ISL_192353 | EPI_ISL_192353 |
| A/environment/Guangdong/DG127/2015 | H7N9 | environment | EPI_ISL_176833 | EPI_ISL_176833 | EPI_ISL_176833 | EPI_ISL_176833 | EPI_ISL_176833 | EPI_ISL_176833 | EPI_ISL_176833 | EPI_ISL_176833 |
| A/environment/Zhejiang/2/2014 | H7N9 | environment | EPI_ISL_163322 | EPI_ISL_163322 | EPI_ISL_163322 | EPI_ISL_163322 | EPI_ISL_163322 | EPI_ISL_163322 | EPI_ISL_163322 | EPI_ISL_163322 |
| A/environment/GD-ZS_21/H7N9/2014-3 | H7N9 | environment | EPI_ISL_198428 | EPI_ISL_198428 | EPI_ISL_198428 | EPI_ISL_198428 | EPI_ISL_198428 | EPI_ISL_198428 | EPI_ISL_198428 | EPI_ISL_198428 |
| A/environment/Anhui/33240/2015 | H7N9 | environment | EPI_ISL_192475 | EPI_ISL_192475 | EPI_ISL_192475 | EPI_ISL_192475 | EPI_ISL_192475 | EPI_ISL_192475 | EPI_ISL_192475 | EPI_ISL_192475 |
| A/environment/Anhui/33248/2015 | H7N9 | environment | EPI_ISL_192474 | EPI_ISL_192474 | EPI_ISL_192474 | EPI_ISL_192474 | EPI_ISL_192474 | EPI_ISL_192474 | EPI_ISL_192474 | EPI_ISL_192474 |
| A/environment/Anhui/33250/2015 | H7N9 | environment | EPI_ISL_192473 | EPI_ISL_192473 | EPI_ISL_192473 | EPI_ISL_192473 | EPI_ISL_192473 | EPI_ISL_192473 | EPI_ISL_192473 | EPI_ISL_192473 |
| A/environment/Shandong-Taian/EV01/2015 | H7N9 | environment | EPI_ISL_192485 | EPI_ISL_192485 | EPI_ISL_192485 | EPI_ISL_192485 | EPI_ISL_192485 | EPI_ISL_192485 | EPI_ISL_192485 | EPI_ISL_192485 |
| A/environment/Shandong-Taian/EV02/2015 | H7N9 | environment | EPI_ISL_192486 | EPI_ISL_192486 | EPI_ISL_192486 | EPI_ISL_192486 | EPI_ISL_192486 | EPI_ISL_192486 | EPI_ISL_192486 | EPI_ISL_192486 |
| A/environment/Shandong-Taian/EV03/2015 | H7N9 | environment | EPI_ISL_192500 | EPI_ISL_192500 | EPI_ISL_192500 | EPI_ISL_192500 | EPI_ISL_192500 | EPI_ISL_192500 | EPI_ISL_192500 | EPI_ISL_192500 |
| A/environment/Bangladesh/917/2012 | H7N9 | environment |  |  | EPI_ISL_165812 | EPI_ISL_165812 | EPI_ISL_165812 | EPI_ISL_165812 | EPI_ISL_165812 | EPI_ISL_165812 |
| A/environment/Guangzhou/XN12917/2014 | H7N9 | environment | KP326321 |  |  |  |  |  |  |  |
| A/environment/Nanjing/2913/2013 | H7N9 | environment | KC896763 | KC896765 | KC896764 | KC896766 | KC896767 | KC896768 | KC896769 | KC896770 |
| A/environment/Shanghai/RL01/2013 | H7N9 | environment | KJ572520 | KJ572521 | KJ572522 |  | KJ572523 | KJ572519 |  |  |
| A/environment/GD-MZ_238/H7N9/2014-4 | H7N9 | environment | EPI_ISL_198429 | EPI_ISL_198429 | EPI_ISL_198429 | EPI_ISL_198429 | EPI_ISL_198429 | EPI_ISL_198429 | EPI_ISL_198429 | EPI_ISL_198429 |
| A/environment/Wuxi/1/2013 | H7N9 | environment | KF150608 | KF150610 | KF150611 | KF150609 | KF150612 | KF150607 | KF150606 | KF150605 |
| A/environment/Shanghai/S1088/2013 | H7N9 | environment | EPI_ISL_138984 | EPI_ISL_138984 | EPI_ISL_138984 | EPI_ISL_138984 | EPI_ISL_138984 | EPI_ISL_138984 | EPI_ISL_138984 | EPI_ISL_138984 |
| A/environment/Shanghai/S1436/2013 | H7N9 | environment | EPI_ISL_142908 | EPI_ISL_142908 | EPI_ISL_142908 | EPI_ISL_142908 | EPI_ISL_142908 | EPI_ISL_142908 | EPI_ISL_142908 | EPI_ISL_142908 |
| A/environment/Shanghai/S1437/2013 | H7N9 | environment | EPI_ISL_142907 | EPI_ISL_142907 | EPI_ISL_142907 | EPI_ISL_142907 | EPI_ISL_142907 | EPI_ISL_142907 | EPI_ISL_142907 | EPI_ISL_142907 |
| A/environment/Shanghai/S1438/2013 | H7N9 | environment | EPI_ISL_142906 | EPI_ISL_142906 | EPI_ISL_142906 | EPI_ISL_142906 | EPI_ISL_142906 | EPI_ISL_142906 | EPI_ISL_142906 | EPI_ISL_142906 |
| A/environment/Shanghai/S1439/2013 | H7N9 | environment | EPI_ISL_142905 | EPI_ISL_142905 | EPI_ISL_142905 | EPI_ISL_142905 | EPI_ISL_142905 | EPI_ISL_142905 | EPI_ISL_142905 | EPI_ISL_142905 |
| A/environment/Shanghai/TY01/2013 | H7N9 | environment | KF918667 | KF918669 | KF918670 | KF918668 | KF918671 | KF918666 | KF918665 | KF918664 |
| A/environment/Hangzhou/34/2013 | H7N9 | environment | KF001519 | KF001520 |  |  |  |  |  |  |
| A/environment/Hangzhou/34-1/2013 | H7N9 | environment | EPI_ISL_139500 | EPI_ISL_139500 | EPI_ISL_139500 | EPI_ISL_139500 | EPI_ISL_139500 | EPI_ISL_139500 | EPI_ISL_139500 | EPI_ISL_139500 |
| A/environment/Hangzhou/37/2013 | H7N9 | environment | EPI_ISL_139905 | EPI_ISL_139905 | EPI_ISL_139905 | EPI_ISL_139905 | EPI_ISL_139905 | EPI_ISL_139905 | EPI_ISL_139905 | EPI_ISL_139905 |
| A/environment/Suzhou/14/2013 | H7N9 | environment | KF034887 | KF034889 | KF034890 | KF034888 | KF034891 | KF034886 | KF034885 | KF034884 |
| A/environment/Zhenjiang/4/2013 | H7N9 | environment | KF007012 | KF007014 | KF007015 | KF007013 | KF007016 | KF007011 | KF007010 | KF007009 |
| A/environment/Suzhou/8/2013 | H7N9 | environment | KF007004 | KF007006 | KF007007 | KF007005 | KF007008 | KF007003 | KF007002 | KF007001 |
| A/environment/Hangzhou/109-1/2013 | H7N9 | environment | EPI_ISL_139908 | EPI_ISL_139908 | EPI_ISL_139908 | EPI_ISL_139908 | EPI_ISL_139908 | EPI_ISL_139908 | EPI_ISL_139908 | EPI_ISL_139908 |
| A/environment/Henan/SC232/2013 | H7N9 | environment | EPI_ISL_142913 | EPI_ISL_142913 | EPI_ISL_142913 | EPI_ISL_142913 | EPI_ISL_142913 | EPI_ISL_142913 | EPI_ISL_142913 | EPI_ISL_142913 |
| A/environment/Henan/SD429/2013 | H7N9 | environment | EPI_ISL_142912 | EPI_ISL_142912 | EPI_ISL_142912 | EPI_ISL_142912 | EPI_ISL_142912 | EPI_ISL_142912 | EPI_ISL_142912 | EPI_ISL_142912 |
| A/environment/Guangdong/25/2013 | H7N9 | environment | KF667746 | KF667748 | KF667749 | KF667747 | KF667750 | KF667745 |  | KF667744 |
| A/environment/Guangdong/30/2013 | H7N9 | environment | KF667751 | KF667753 | KF667754 | KF667752 | KF667755 |  |  |  |
| A/environment/Guangdong/C13281025/2013 | H7N9 | environment | EPI_ISL_151429 | EPI_ISL_151429 | EPI_ISL_151429 | EPI_ISL_151429 | EPI_ISL_151429 | EPI_ISL_151429 | EPI_ISL_151429 | EPI_ISL_151429 |
| A/environment/Guangdong/C13281030/2013 | H7N9 | environment | EPI_ISL_151430 | EPI_ISL_151430 | EPI_ISL_151430 | EPI_ISL_151430 | EPI_ISL_151430 | EPI_ISL_151430 | EPI_ISL_151430 | EPI_ISL_151430 |
| A/environment/Shandong/1/2013 | H7N9 | environment | EPI_ISL_141189 | EPI_ISL_141189 | EPI_ISL_141189 | EPI_ISL_141189 | EPI_ISL_141189 | EPI_ISL_141189 | EPI_ISL_141189 | EPI_ISL_141189 |
| A/environment/Hunan/39719/2015 | H7N9 | environment | EPI_ISL_192470 | EPI_ISL_192470 | EPI_ISL_192470 | EPI_ISL_192470 | EPI_ISL_192470 | EPI_ISL_192470 | EPI_ISL_192470 | EPI_ISL_192470 |
| A/environment/Fujian/SC337/2013 | H7N9 | environment | EPI_ISL_142914 | EPI_ISL_142914 | EPI_ISL_142914 | EPI_ISL_142914 | EPI_ISL_142914 | EPI_ISL_142914 | EPI_ISL_142914 | EPI_ISL_142914 |
| A/environment/GD-ZS_162/H7N9/2014-5 | H7N9 | environment | EPI_ISL_198430 | EPI_ISL_198430 |  | EPI_ISL_198430 | EPI_ISL_198430 | EPI_ISL_198430 | EPI_ISL_198430 | EPI_ISL_198430 |
| A/environment/Anhui/39280/2015 | H7N9 | environment | EPI_ISL_192477 | EPI_ISL_192477 | EPI_ISL_192477 | EPI_ISL_192477 | EPI_ISL_192477 | EPI_ISL_192477 | EPI_ISL_192477 | EPI_ISL_192477 |
| A/environment/Shandong/SD038/2013 | H7N9 | environment | EPI_ISL_142911 | EPI_ISL_142911 | EPI_ISL_142911 | EPI_ISL_142911 | EPI_ISL_142911 | EPI_ISL_142911 | EPI_ISL_142911 | EPI_ISL_142911 |
| A/environment/Shandong/SD039/2013 | H7N9 | environment | EPI_ISL_142910 | EPI_ISL_142910 | EPI_ISL_142910 | EPI_ISL_142910 | EPI_ISL_142910 | EPI_ISL_142910 | EPI_ISL_142910 | EPI_ISL_142910 |
| A/environment/Shandong/SD049/2013 | H7N9 | environment | EPI_ISL_142909 | EPI_ISL_142909 | EPI_ISL_142909 | EPI_ISL_142909 | EPI_ISL_142909 | EPI_ISL_142909 | EPI_ISL_142909 | EPI_ISL_142909 |
| A/environment/Guangdong-Guangzhou/XN01070/2013 | H7N9 | environment | EPI_ISL_192352 | EPI_ISL_192352 | EPI_ISL_192352 | EPI_ISL_192352 | EPI_ISL_192352 | EPI_ISL_192352 | EPI_ISL_192352 | EPI_ISL_192352 |
| A/environment/Guangdong/47403/2014 | H7N9 | environment | EPI_ISL_192468 | EPI_ISL_192468 | EPI_ISL_192468 | EPI_ISL_192468 | EPI_ISL_192468 | EPI_ISL_192468 | EPI_ISL_192468 | EPI_ISL_192468 |
| A/environment/Shandong/YT001/2014 | H7N9 | environment | EPI_ISL_192389 | EPI_ISL_192389 | EPI_ISL_192389 | EPI_ISL_192389 | EPI_ISL_192389 | EPI_ISL_192389 | EPI_ISL_192389 | EPI_ISL_192389 |
| A/environment/Shandong/YT002/2014 | H7N9 | environment | EPI_ISL_192379 | EPI_ISL_192379 | EPI_ISL_192379 | EPI_ISL_192379 | EPI_ISL_192379 | EPI_ISL_192379 | EPI_ISL_192379 | EPI_ISL_192379 |
| A/environment/Shandong/YT003/2014 | H7N9 | environment | EPI_ISL_192378 | EPI_ISL_192378 | EPI_ISL_192378 | EPI_ISL_192378 | EPI_ISL_192378 | EPI_ISL_192378 | EPI_ISL_192378 | EPI_ISL_192378 |
| A/environment/Shandong/YT004/2014 | H7N9 | environment | EPI_ISL_192388 | EPI_ISL_192388 | EPI_ISL_192388 | EPI_ISL_192388 | EPI_ISL_192388 | EPI_ISL_192388 | EPI_ISL_192388 | EPI_ISL_192388 |
| A/environment/Shandong/YT005/2014 | H7N9 | environment | EPI_ISL_192387 | EPI_ISL_192387 | EPI_ISL_192387 | EPI_ISL_192387 | EPI_ISL_192387 | EPI_ISL_192387 | EPI_ISL_192387 | EPI_ISL_192387 |
| A/environment/Shandong/YT007/2014 | H7N9 | environment | EPI_ISL_192386 | EPI_ISL_192386 | EPI_ISL_192386 | EPI_ISL_192386 | EPI_ISL_192386 | EPI_ISL_192386 | EPI_ISL_192386 | EPI_ISL_192386 |
| A/environment/Shandong/YT009/2014 | H7N9 | environment | EPI_ISL_192385 | EPI_ISL_192385 | EPI_ISL_192385 | EPI_ISL_192385 | EPI_ISL_192385 | EPI_ISL_192385 | EPI_ISL_192385 | EPI_ISL_192385 |
| A/environment/GD-SZ_1/H7N9/2013 | H7N9 | environment | EPI_ISL_198378 |  |  |  |  |  |  |  |
| A/environment/GD-SZ_228/H7N9/2013 | H7N9 | environment | EPI_ISL_198381 | EPI_ISL_198381 |  |  | EPI_ISL_198381 |  |  |  |
| A/environment/GD-SZ_236/H7N9/2013 | H7N9 | environment | EPI_ISL_198382 | EPI_ISL_198382 |  |  | EPI_ISL_198382 |  |  |  |
| A/environment/GD-SZ_237/H7N9/2013 | H7N9 | environment | EPI_ISL_198383 | EPI_ISL_198383 |  |  |  |  |  |  |
| A/environment/GD-SZ_293/H7N9/2013 | H7N9 | environment | EPI_ISL_198384 |  |  |  |  |  |  |  |
| A/environment/GD-SZ_294/H7N9/2013 | H7N9 | environment | EPI_ISL_198385 | EPI_ISL_198385 |  |  |  |  |  |  |
| A/environment/GD-SZ_295/H7N9/2013 | H7N9 | environment | EPI_ISL_198386 | EPI_ISL_198386 |  |  |  |  |  |  |
| A/environment/GD-SZ_296/H7N9/2013 | H7N9 | environment | EPI_ISL_198387 | EPI_ISL_198387 |  |  |  |  |  |  |
| A/environment/GD-YJ_25003/H7N9/2013 | H7N9 | environment |  | EPI_ISL_198388 |  |  |  | EPI_ISL_198388 | EPI_ISL_198388 | EPI_ISL_198388 |
| A/environment/GD-YJ_3/H7N9/2013 | H7N9 | environment | EPI_ISL_198389 |  |  |  |  |  |  |  |
| A/environment/GD-YJ_307/H7N9/2013 | H7N9 | environment | EPI_ISL_198390 | EPI_ISL_198390 |  |  |  |  |  |  |
| A/environment/GD-YJ_327/H7N9/2013 | H7N9 | environment | EPI_ISL_198391 | EPI_ISL_198391 |  |  |  |  |  |  |
| A/environment/GD-YJ_329/H7N9/2013 | H7N9 | environment | EPI_ISL_198392 | EPI_ISL_198392 |  |  |  |  |  |  |
| A/environment/GD-YJ_338/H7N9/2013 | H7N9 | environment | EPI_ISL_198393 | EPI_ISL_198393 |  |  |  |  |  |  |
| A/environment/GD-YJ_359/H7N9/2013 | H7N9 | environment | EPI_ISL_198394 | EPI_ISL_198394 |  |  |  |  |  |  |
| A/environment/GD-YJ_364/H7N9/2013 | H7N9 | environment | EPI_ISL_198395 | EPI_ISL_198395 |  |  |  |  |  |  |
| A/environment/GD-YJ_365/H7N9/2013 | H7N9 | environment | EPI_ISL_198396 | EPI_ISL_198396 |  |  |  |  |  |  |
| A/environment/GD-YJ_366/H7N9/2013 | H7N9 | environment | EPI_ISL_198397 | EPI_ISL_198397 |  |  |  |  |  |  |
| A/environment/GD-YJ_368/H7N9/2013 | H7N9 | environment | EPI_ISL_198398 | EPI_ISL_198398 |  |  |  |  |  |  |
| A/environment/GD-YJ_370/H7N9/2013 | H7N9 | environment | EPI_ISL_198399 | EPI_ISL_198399 |  |  |  |  |  |  |
| A/environment/GD-YJ_371/H7N9/2013 | H7N9 | environment | EPI_ISL_198400 | EPI_ISL_198400 |  |  |  |  |  |  |
| A/environment/GD-YJ_373/H7N9/2013 | H7N9 | environment | EPI_ISL_198401 | EPI_ISL_198401 |  |  |  |  |  |  |
| A/environment/GD-YJ_379/H7N9/2013 | H7N9 | environment | EPI_ISL_198402 | EPI_ISL_198402 |  |  |  |  |  |  |
| A/environment/GD-JY_145/H7N9/2014 | H7N9 | environment |  | EPI_ISL_198407 |  |  |  |  |  |  |
| A/environment/GD-JY_201/H7N9/2014 | H7N9 | environment | EPI_ISL_198408 | EPI_ISL_198408 |  |  | EPI_ISL_198408 |  |  |  |
| A/environment/GD-PY_298/H7N9/2014 | H7N9 | environment |  | EPI_ISL_198409 |  |  |  |  |  |  |
| A/environment/GD-PY_299/H7N9/2014 | H7N9 | environment |  | EPI_ISL_198410 |  |  |  |  |  |  |
| A/environment/GD-PY_301/H7N9/2014 | H7N9 | environment |  | EPI_ISL_198411 |  |  |  |  |  |  |
| A/environment/GD-PY_352/H7N9/2014 | H7N9 | environment |  | EPI_ISL_198412 |  |  |  |  |  |  |
| A/environment/GD-PY_560/H7N9/2014 | H7N9 | environment |  | EPI_ISL_198414 |  |  |  |  |  |  |
| A/environment/GD-ST_100/H7N9/2014 | H7N9 | environment | EPI_ISL_198431 |  |  |  |  |  |  |  |
| A/environment/GD-ST_98/H7N9/2014 | H7N9 | environment | EPI_ISL_198379 | EPI_ISL_198379 |  |  |  |  |  |  |
| A/environment/GD-ST_99/H7N9/2014 | H7N9 | environment | EPI_ISL_198380 | EPI_ISL_198380 |  |  |  |  |  |  |
| A/environment/GD-ZS_2151/H7N9/2014 | H7N9 | environment | EPI_ISL_198416 | EPI_ISL_198416 |  |  | EPI_ISL_198416 |  |  |  |
| A/environment/GD-ZS_2154/H7N9/2014 | H7N9 | environment | EPI_ISL_198417 | EPI_ISL_198417 |  |  | EPI_ISL_198417 |  |  |  |
| A/environment/GD-ZS_2155/H7N9/2014 | H7N9 | environment | EPI_ISL_198418 | EPI_ISL_198418 | EPI_ISL_198418 | EPI_ISL_198418 | EPI_ISL_198418 | EPI_ISL_198418 | EPI_ISL_198418 | EPI_ISL_198418 |
| A/environment/GD-ZS_478/H7N9/2014 | H7N9 | environment |  | EPI_ISL_198420 |  |  |  |  |  |  |
| A/environment/GD-ZS_704/H7N9/2014 | H7N9 | environment | EPI_ISL_198421 |  |  |  | EPI_ISL_198421 |  |  |  |
| A/environment/GD-ZS_712/H7N9/2014 | H7N9 | environment |  | EPI_ISL_198423 |  |  |  |  |  |  |
| A/environment/Hebei/1/2013 | H7N9 | environment | KP861978 | KP861979 |  |  |  |  |  |  |
| A/environment/Xinjiang/73033/2014 | H7N9 | environment | EPI_ISL_192390 | EPI_ISL_192390 | EPI_ISL_192390 | EPI_ISL_192390 | EPI_ISL_192390 | EPI_ISL_192390 | EPI_ISL_192390 | EPI_ISL_192390 |
| A/environment/Bangladesh/1008/2010 | H7N9 | environment |  |  | EPI_ISL_165811 | EPI_ISL_165811 | EPI_ISL_165811 | EPI_ISL_165811 | EPI_ISL_165811 | EPI_ISL_165811 |
| A/environment/Shenzhen/1/2013 | H7N9 | environment | KF922737 |  |  |  |  |  |  |  |
| A/environment/Xinjiang/00344/2014 | H7N9 | environment | EPI_ISL_192319 | EPI_ISL_192319 | EPI_ISL_192319 | EPI_ISL_192319 | EPI_ISL_192319 | EPI_ISL_192319 | EPI_ISL_192319 | EPI_ISL_192319 |
| A/environment/Xinjiang/00347/2014 | H7N9 | environment | EPI_ISL_192320 | EPI_ISL_192320 | EPI_ISL_192320 | EPI_ISL_192320 | EPI_ISL_192320 | EPI_ISL_192320 | EPI_ISL_192320 | EPI_ISL_192320 |
| A/environment/Jiangsu/98337/2014 | H7N9 | environment | EPI_ISL_192340 | EPI_ISL_192340 | EPI_ISL_192340 | EPI_ISL_192340 | EPI_ISL_192340 | EPI_ISL_192340 | EPI_ISL_192340 | EPI_ISL_192340 |
| A/environment/Guangdong/C16283222/2016 | H7N9 | environment | EPI_ISL_249310 | EPI_ISL_249310 | EPI_ISL_249310 | EPI_ISL_249310 | EPI_ISL_249310 | EPI_ISL_249310 | EPI_ISL_249310 | EPI_ISL_249310 |
| A/environment/Jiangsu/03174/2013 | H7N9 | environment | EPI_ISL_192452 | EPI_ISL_192452 | EPI_ISL_192452 | EPI_ISL_192452 | EPI_ISL_192452 | EPI_ISL_192452 | EPI_ISL_192452 | EPI_ISL_192452 |
| A/environment/Jiangsu/09386/2013 | H7N9 | environment | EPI_ISL_192404 | EPI_ISL_192404 | EPI_ISL_192404 | EPI_ISL_192404 | EPI_ISL_192404 | EPI_ISL_192404 | EPI_ISL_192404 | EPI_ISL_192404 |
| A/environment/Guangdong/25003/2013 | H7N9 | environment | EPI_ISL_192467 | EPI_ISL_192467 | EPI_ISL_192467 | EPI_ISL_192467 | EPI_ISL_192467 | EPI_ISL_192467 | EPI_ISL_192467 | EPI_ISL_192467 |
| A/environmental/Guangdong/00307/2013 | H7N9 | environment | EPI_ISL_192396 | EPI_ISL_192396 | EPI_ISL_192396 | EPI_ISL_192396 | EPI_ISL_192396 | EPI_ISL_192396 | EPI_ISL_192396 | EPI_ISL_192396 |
| A/environmental/Guangdong/00329/2013 | H7N9 | environment | EPI_ISL_192399 | EPI_ISL_192399 | EPI_ISL_192399 | EPI_ISL_192399 | EPI_ISL_192399 | EPI_ISL_192399 | EPI_ISL_192399 | EPI_ISL_192399 |
| A/environment/Zhejiang/07813/2013 | H7N9 | environment | EPI_ISL_192434 | EPI_ISL_192434 | EPI_ISL_192434 | EPI_ISL_192434 | EPI_ISL_192434 | EPI_ISL_192434 | EPI_ISL_192434 | EPI_ISL_192434 |
| A/environment/Guangxi/06891/2014 | H7N9 | environment | EPI_ISL_192491 | EPI_ISL_192491 | EPI_ISL_192491 | EPI_ISL_192491 | EPI_ISL_192491 | EPI_ISL_192491 | EPI_ISL_192491 | EPI_ISL_192491 |
| A/environment/Guangdong/02621/2013 | H7N9 | environment | EPI_ISL_192456 | EPI_ISL_192456 | EPI_ISL_192456 | EPI_ISL_192456 | EPI_ISL_192456 | EPI_ISL_192456 | EPI_ISL_192456 | EPI_ISL_192456 |
| A/environment/Guangdong/02622/2013 | H7N9 | environment | EPI_ISL_192457 | EPI_ISL_192457 | EPI_ISL_192457 | EPI_ISL_192457 | EPI_ISL_192457 | EPI_ISL_192457 | EPI_ISL_192457 | EPI_ISL_192457 |
| A/environment/Jiangsu/03137/2013 | H7N9 | environment | EPI_ISL_192453 | EPI_ISL_192453 | EPI_ISL_192453 | EPI_ISL_192453 | EPI_ISL_192453 | EPI_ISL_192453 | EPI_ISL_192453 | EPI_ISL_192453 |
| A/environment/Fujian/23611/2014 | H7N9 | environment | EPI_ISL_192476 | EPI_ISL_192476 | EPI_ISL_192476 | EPI_ISL_192476 | EPI_ISL_192476 | EPI_ISL_192476 | EPI_ISL_192476 | EPI_ISL_192476 |
| A/Anhui/1-DEWH730/2013 | H7N9 | Human | CY187618 | CY187620 | CY187619 | CY187621 | CY187622 | CY187623 | CY187624 | CY187625 |
| A/Anhui/DEWH72-01/2013 | H7N9 | Human | CY181513 | CY181515 | CY181514 | CY181516 | CY181517 | CY181518 | CY181519 | CY181520 |
| A/Anhui/DEWH72-02/2013 | H7N9 | Human | CY181521 | CY181523 | CY181522 | CY181524 | CY181525 | CY181526 | CY181527 | CY181528 |
| A/Anhui/DEWH72-03/2013 | H7N9 | Human | CY181529 | CY181531 | CY181530 | CY181532 | CY181533 | CY181534 | CY181535 | CY181536 |
| A/Anhui/DEWH72-04/2013 | H7N9 | Human | CY181537 | CY181539 | CY181538 | CY181540 | CY181541 | CY181542 | CY181543 | CY181544 |
| A/Anhui/DEWH72-05/2013 | H7N9 | Human | CY181545 | CY181547 | CY181546 | CY181548 | CY181549 | CY181550 | CY181551 | CY181552 |
| A/Anhui/DEWH72-06/2013 | H7N9 | Human | CY181553 | CY181555 | CY181554 | CY181556 | CY181557 | CY181558 | CY181559 | CY181560 |
| A/Anhui/DEWH72-07/2013 | H7N9 | Human | CY181561 | CY181563 | CY181562 | CY181564 | CY181565 | CY181566 | CY181567 | CY181568 |
| A/Anhui/DEWH72-08/2013 | H7N9 | Human | CY181569 | CY181571 | CY181570 | CY181572 | CY181573 | CY181574 | CY181575 | CY181576 |
| A/Anhui/DEWH72-09/2013 | H7N9 | Human | CY181577 | CY181579 | CY181578 | CY181580 | CY181581 | CY181582 | CY181583 | CY181584 |
| A/Taiwan/1/2013 | H7N9 | Human | KF018039 |  |  | KF018040 | KF018036 |  | KF018038 | KF018037 |
| A/GD-10/2014/H7N9/2014-01-03 | H7N9 | human | EPI_ISL_198670 | EPI_ISL_198670 | EPI_ISL_198670 | EPI_ISL_198670 | EPI_ISL_198670 | EPI_ISL_198670 | EPI_ISL_198670 | EPI_ISL_198670 |
| A/GD-12/2014/H7N9/2014-01-03 | H7N9 | human | EPI_ISL_198671 | EPI_ISL_198671 | EPI_ISL_198671 | EPI_ISL_198671 | EPI_ISL_198671 | EPI_ISL_198671 | EPI_ISL_198671 | EPI_ISL_198671 |
| A/GD-13/2014/H7N9/2014-01-03 | H7N9 | human | EPI_ISL_198672 | EPI_ISL_198672 | EPI_ISL_198672 | EPI_ISL_198672 | EPI_ISL_198672 | EPI_ISL_198672 | EPI_ISL_198672 | EPI_ISL_198672 |
| A/Shanghai/01/2014 | H7N9 | Human | KJ411975 | KJ411977 | KJ411976 | KJ411978 | KJ411979 | KJ411980 | KJ411981 | KJ411982 |
| A/Shanghai/Mix1/2014 | H7N9 | Human | KJ946417 | KJ946418 | KP058488 | KP058487 | KP058489 | KP058486 | KP058485 | KP058484 |
| A/GD-1/2015/H7N9/2015-01-03 | H7N9 | human | EPI_ISL_198721 | EPI_ISL_198721 | EPI_ISL_198721 | EPI_ISL_198721 | EPI_ISL_198721 | EPI_ISL_198721 | EPI_ISL_198721 | EPI_ISL_198721 |
| A/Guangdong/8H324/2017 | H7N9 | human | EPI_ISL_263365 | EPI_ISL_263365 | EPI_ISL_263365 | EPI_ISL_263365 | EPI_ISL_263365 | EPI_ISL_263365 | EPI_ISL_263365 | EPI_ISL_263365 |
| A/Guangdong/HP001/2017 | H7N9 | Human | KY643843 | KY643847 | KY643849 | KY643845 | KY643851 | KY643841 | KY643839 | KY643837 |
| A/Guangdong/SP440/2017 | H7N9 | Human | KY643844 | KY643848 | KY643850 | KY643846 | KY643852 | KY643842 | KY643840 | KY643838 |
| A/Zhejiang/2/2017 | H7N9 | human | EPI_ISL_242855 | EPI_ISL_242855 | EPI_ISL_242855 | EPI_ISL_242855 | EPI_ISL_242855 | EPI_ISL_242855 | EPI_ISL_242855 | EPI_ISL_242855 |
| A/Hong_Kong/125/2017 | H7N9 | human | EPI_ISL_259269 | EPI_ISL_259269 | EPI_ISL_259269 | EPI_ISL_259269 | EPI_ISL_259269 | EPI_ISL_259269 | EPI_ISL_259269 | EPI_ISL_259269 |
| A/Hong_Kong/VB17002884/2017 | H7N9 | human | EPI_ISL_240829 | EPI_ISL_240829 | EPI_ISL_240829 | EPI_ISL_240829 | EPI_ISL_240829 | EPI_ISL_240829 | EPI_ISL_240829 | EPI_ISL_240829 |
| A/GD-10/2015/H7N9/2015-01-06 | H7N9 | human | EPI_ISL_198723 | EPI_ISL_198723 | EPI_ISL_198723 | EPI_ISL_198723 | EPI_ISL_198723 | EPI_ISL_198723 | EPI_ISL_198723 | EPI_ISL_198723 |
| A/GD-2/2015/H7N9/2015-01-06 | H7N9 | human | EPI_ISL_198722 | EPI_ISL_198722 | EPI_ISL_198722 | EPI_ISL_198722 | EPI_ISL_198722 | EPI_ISL_198722 | EPI_ISL_198722 | EPI_ISL_198722 |
| A/Anhui/13439/2017 | H7N9 | human | EPI_ISL_258023 | EPI_ISL_258023 | EPI_ISL_258023 | EPI_ISL_258023 | EPI_ISL_258023 | EPI_ISL_258023 | EPI_ISL_258023 | EPI_ISL_258023 |
| A/GD-18/2014/H7N9/2014-01-07 | H7N9 | human | EPI_ISL_198673 | EPI_ISL_198673 | EPI_ISL_198673 | EPI_ISL_198673 | EPI_ISL_198673 | EPI_ISL_198673 | EPI_ISL_198673 | EPI_ISL_198673 |
| A/GD-19/2014/H7N9/2014-01-07 | H7N9 | human | EPI_ISL_198674 | EPI_ISL_198674 | EPI_ISL_198674 | EPI_ISL_198674 | EPI_ISL_198674 | EPI_ISL_198674 | EPI_ISL_198674 | EPI_ISL_198674 |
| A/Hong_Kong/734/2014 | H7N9 | human | EPI_ISL_153338 | EPI_ISL_153338 | EPI_ISL_153338 | EPI_ISL_153338 | EPI_ISL_153338 | EPI_ISL_153338 | EPI_ISL_153338 | EPI_ISL_153338 |
| A/Zhejiang/1/2017 | H7N9 | human | EPI_ISL_242856 | EPI_ISL_242856 | EPI_ISL_242856 | EPI_ISL_242856 | EPI_ISL_242856 | EPI_ISL_242856 | EPI_ISL_242856 | EPI_ISL_242856 |
| A/Hong_Kong/214/2017 | H7N9 | human | EPI_ISL_242275 | EPI_ISL_242275 | EPI_ISL_242275 | EPI_ISL_242275 | EPI_ISL_242275 | EPI_ISL_242275 | EPI_ISL_242275 | EPI_ISL_242275 |
| A/GD-24/2014/H7N9/2014-01-10 | H7N9 | human | EPI_ISL_198675 | EPI_ISL_198675 | EPI_ISL_198675 | EPI_ISL_198675 | EPI_ISL_198675 | EPI_ISL_198675 | EPI_ISL_198675 | EPI_ISL_198675 |
| A/GD-26/2014/H7N9/2014-01-10 | H7N9 | human | EPI_ISL_198676 | EPI_ISL_198676 | EPI_ISL_198676 | EPI_ISL_198676 | EPI_ISL_198676 | EPI_ISL_198676 | EPI_ISL_198676 | EPI_ISL_198676 |
| A/GD-29/2014/H7N9/2014-01-11 | H7N9 | human | EPI_ISL_198677 | EPI_ISL_198677 | EPI_ISL_198677 | EPI_ISL_198677 | EPI_ISL_198677 | EPI_ISL_198677 | EPI_ISL_198677 | EPI_ISL_198677 |
| A/GD-31/2014/H7N9/2014-01-11 | H7N9 | human | EPI_ISL_198678 | EPI_ISL_198678 | EPI_ISL_198678 | EPI_ISL_198678 | EPI_ISL_198678 | EPI_ISL_198678 | EPI_ISL_198678 | EPI_ISL_198678 |
| A/Shenzhen/SP-Z93/2014 | H7N9 | Human | KP414106 | KP414108 | KP414109 | KP414107 | KP414110 | KP414105 | KP414104 | KP414103 |
| A/GD-17/2015/H7N9/2015-01-11 | H7N9 | human | EPI_ISL_198725 | EPI_ISL_198725 | EPI_ISL_198725 | EPI_ISL_198725 | EPI_ISL_198725 | EPI_ISL_198725 | EPI_ISL_198725 | EPI_ISL_198725 |
| A/GD-18/2015/H7N9/2015-01-11 | H7N9 | human | EPI_ISL_198724 | EPI_ISL_198724 | EPI_ISL_198724 | EPI_ISL_198724 | EPI_ISL_198724 | EPI_ISL_198724 | EPI_ISL_198724 | EPI_ISL_198724 |
| A/GD-33/2014/H7N9/2014-01-12 | H7N9 | human | EPI_ISL_198679 | EPI_ISL_198679 | EPI_ISL_198679 | EPI_ISL_198679 | EPI_ISL_198679 | EPI_ISL_198679 | EPI_ISL_198679 | EPI_ISL_198679 |
| A/GD-34/2014/H7N9/2014-01-12 | H7N9 | human | EPI_ISL_198680 | EPI_ISL_198680 | EPI_ISL_198680 | EPI_ISL_198680 | EPI_ISL_198680 | EPI_ISL_198680 | EPI_ISL_198680 | EPI_ISL_198680 |
| A/GD-35/2014/H7N9/2014-01-12 | H7N9 | human | EPI_ISL_198682 | EPI_ISL_198682 | EPI_ISL_198682 | EPI_ISL_198682 | EPI_ISL_198682 | EPI_ISL_198682 | EPI_ISL_198682 | EPI_ISL_198682 |
| A/GD-36/2014/H7N9/2014-01-12 | H7N9 | human | EPI_ISL_198681 | EPI_ISL_198681 | EPI_ISL_198681 | EPI_ISL_198681 | EPI_ISL_198681 | EPI_ISL_198681 | EPI_ISL_198681 | EPI_ISL_198681 |
| A/Hangzhou/10-1/2014 | H7N9 | human | EPI_ISL_157592 | EPI_ISL_157592 |  |  |  |  |  |  |
| A/Anhui/13423/2017 | H7N9 | human | EPI_ISL_258012 | EPI_ISL_258012 | EPI_ISL_258012 | EPI_ISL_258012 | EPI_ISL_258012 | EPI_ISL_258012 | EPI_ISL_258012 | EPI_ISL_258012 |
| A/Anhui/13431/2017 | H7N9 | human | EPI_ISL_258018 | EPI_ISL_258018 | EPI_ISL_258018 | EPI_ISL_258018 | EPI_ISL_258018 | EPI_ISL_258018 | EPI_ISL_258018 | EPI_ISL_258018 |
| A/Anhui/13441/2017 | H7N9 | human | EPI_ISL_258024 | EPI_ISL_258024 | EPI_ISL_258024 | EPI_ISL_258024 | EPI_ISL_258024 | EPI_ISL_258024 | EPI_ISL_258024 | EPI_ISL_258024 |
| A/Anhui/13442/2017 | H7N9 | human | EPI_ISL_258025 | EPI_ISL_258025 | EPI_ISL_258025 | EPI_ISL_258025 | EPI_ISL_258025 | EPI_ISL_258025 | EPI_ISL_258025 | EPI_ISL_258025 |
| A/Jiangsu/11581/2017 | H7N9 | human | EPI_ISL_258002 | EPI_ISL_258002 | EPI_ISL_258002 | EPI_ISL_258002 | EPI_ISL_258002 | EPI_ISL_258002 | EPI_ISL_258002 | EPI_ISL_258002 |
| A/Shenzhen/SP-W1/2014 | H7N9 | Human | KP414138 | KP414140 | KP414141 | KP414139 | KP414142 | KP414137 | KP414136 | KP414135 |
| A/Qingyuan/GIRD01/2017 | H7N9 | Human | KY621542 | KY621544 | KY621545 | KY621543 | KY621546 | KY621541 | KY621540 | KY621539 |
| A/Qingyuan/GIRD1/2017 | H7N9 | human | EPI_ISL_249102 | EPI_ISL_249102 | EPI_ISL_249102 | EPI_ISL_249102 | EPI_ISL_249102 | EPI_ISL_249102 | EPI_ISL_249102 | EPI_ISL_249102 |
| A/Yunnan/13501/2017 | H7N9 | human | EPI_ISL_257994 | EPI_ISL_257994 | EPI_ISL_257994 | EPI_ISL_257994 | EPI_ISL_257994 | EPI_ISL_257994 | EPI_ISL_257994 | EPI_ISL_257994 |
| A/GD-43/2014/H7N9/2014-01-15 | H7N9 | human | EPI_ISL_198683 | EPI_ISL_198683 | EPI_ISL_198683 | EPI_ISL_198683 | EPI_ISL_198683 | EPI_ISL_198683 | EPI_ISL_198683 | EPI_ISL_198683 |
| A/GD-20/2015/H7N9/2015-01-15 | H7N9 | human | EPI_ISL_198726 | EPI_ISL_198726 | EPI_ISL_198726 | EPI_ISL_198726 | EPI_ISL_198726 | EPI_ISL_198726 | EPI_ISL_198726 | EPI_ISL_198726 |
| A/GD-46/2014/H7N9/2014-01-16 | H7N9 | human | EPI_ISL_198684 | EPI_ISL_198684 | EPI_ISL_198684 | EPI_ISL_198684 | EPI_ISL_198684 | EPI_ISL_198684 | EPI_ISL_198684 | EPI_ISL_198684 |
| A/Shenzhen/SP4/2014 | H7N9 | Human | KP414114 | KP414116 | KP414117 | KP414115 | KP414118 | KP414113 | KP414112 |  |
| A/British_Columbia/1/2015 | H7N9 | human | EPI_ISL_171342 | EPI_ISL_171342 | EPI_ISL_171342 | EPI_ISL_171342 | EPI_ISL_171342 | EPI_ISL_171342 | EPI_ISL_171342 | EPI_ISL_171342 |
| A/GD-21/2015/H7N9/2015-01-16 | H7N9 | human | EPI_ISL_198727 | EPI_ISL_198727 | EPI_ISL_198727 | EPI_ISL_198727 | EPI_ISL_198727 | EPI_ISL_198727 | EPI_ISL_198727 | EPI_ISL_198727 |
| A/Shanghai/PD-01/2014 | H7N9 | Human | KJ195792 | KJ195794 | KJ195795 | KJ195793 | KJ195796 | KJ549800 | KJ195791 | KJ549799 |
| A/Shanghai/PD-02/2014 | H7N9 | Human | KJ195797 | KJ195799 | KJ195800 | KJ195798 | KJ195801 | KJ549803 | KJ549802 | KJ549801 |
| A/Fujian/22/2015 | H7N9 | human | EPI_ISL_223680 | EPI_ISL_223680 | EPI_ISL_223680 | EPI_ISL_223680 | EPI_ISL_223680 | EPI_ISL_223680 | EPI_ISL_223680 | EPI_ISL_223680 |
| A/Anhui/13432/2017 | H7N9 | human | EPI_ISL_258019 | EPI_ISL_258019 | EPI_ISL_258019 | EPI_ISL_258019 | EPI_ISL_258019 | EPI_ISL_258019 | EPI_ISL_258019 | EPI_ISL_258019 |
| A/Anhui/13433/2017 | H7N9 | human | EPI_ISL_258020 | EPI_ISL_258020 | EPI_ISL_258020 | EPI_ISL_258020 | EPI_ISL_258020 | EPI_ISL_258020 | EPI_ISL_258020 | EPI_ISL_258020 |
| A/Hangzhou/17-1/2014 | H7N9 | human | EPI_ISL_157594 | EPI_ISL_157594 |  |  |  |  |  |  |
| A/Hangzhou/19/2014 | H7N9 | human | EPI_ISL_163997 | EPI_ISL_163997 | EPI_ISL_163997 | EPI_ISL_163997 | EPI_ISL_163997 | EPI_ISL_163997 | EPI_ISL_163997 | EPI_ISL_163997 |
| A/Jiaxing/20/2014 | H7N9 | human | EPI_ISL_163998 | EPI_ISL_163998 | EPI_ISL_163998 | EPI_ISL_163998 | EPI_ISL_163998 | EPI_ISL_163998 | EPI_ISL_163998 | EPI_ISL_163998 |
| A/Shenzhen/SP16/2014 | H7N9 | Human | KP414146 | KP414148 | KP414149 | KP414147 | KP414150 | KP414145 | KP414144 | KP414143 |
| A/GD-48/2014/H7N9/2014-01-19 | H7N9 | human | EPI_ISL_198685 | EPI_ISL_198685 | EPI_ISL_198685 | EPI_ISL_198685 | EPI_ISL_198685 | EPI_ISL_198685 | EPI_ISL_198685 | EPI_ISL_198685 |
| A/GD-44/2015/H7N9/2015-01-19 | H7N9 | human | EPI_ISL_198728 | EPI_ISL_198728 | EPI_ISL_198728 | EPI_ISL_198728 | EPI_ISL_198728 | EPI_ISL_198728 | EPI_ISL_198728 | EPI_ISL_198728 |
| A/Hangzhou/30/2014 | H7N9 | human | EPI_ISL_163999 | EPI_ISL_163999 | EPI_ISL_163999 | EPI_ISL_163999 | EPI_ISL_163999 | EPI_ISL_163999 | EPI_ISL_163999 | EPI_ISL_163999 |
| A/Shenzhen/SP17/2014 | H7N9 | Human | KP414122 | KP414124 | KP414125 | KP414123 | KP414126 | KP414121 | KP414120 | KP414119 |
| A/Shenzhen/SP26/2014 | H7N9 | Human | KP414130 | KP414132 | KP414133 | KP414131 | KP414134 | KP414129 | KP414128 | KP414127 |
| A/Anhui/13421/2017 | H7N9 | human | EPI_ISL_258011 | EPI_ISL_258011 | EPI_ISL_258011 | EPI_ISL_258011 | EPI_ISL_258011 | EPI_ISL_258011 | EPI_ISL_258011 | EPI_ISL_258011 |
| A/Anhui/13446/2017 | H7N9 | human | EPI_ISL_258027 | EPI_ISL_258027 | EPI_ISL_258027 | EPI_ISL_258027 | EPI_ISL_258027 | EPI_ISL_258027 | EPI_ISL_258027 | EPI_ISL_258027 |
| A/GD-27/2015/H7N9/2015-01-21 | H7N9 | human | EPI_ISL_198729 | EPI_ISL_198729 | EPI_ISL_198729 | EPI_ISL_198729 | EPI_ISL_198729 | EPI_ISL_198729 | EPI_ISL_198729 | EPI_ISL_198729 |
| A/Henan/11156/2017 | H7N9 | human | EPI_ISL_258007 | EPI_ISL_258007 | EPI_ISL_258007 | EPI_ISL_258007 | EPI_ISL_258007 | EPI_ISL_258007 | EPI_ISL_258007 | EPI_ISL_258007 |
| A/Shenzhen/SP38/2014 | H7N9 | Human | KP414154 | KP414156 | KP414157 | KP414155 | KP414158 | KP414153 | KP414152 | KP414151 |
| A/GD-30/2015/H7N9/2015-01-22 | H7N9 | human | EPI_ISL_198731 | EPI_ISL_198731 | EPI_ISL_198731 | EPI_ISL_198731 | EPI_ISL_198731 | EPI_ISL_198731 | EPI_ISL_198731 | EPI_ISL_198731 |
| A/GD-43/2015/H7N9/2015-01-22 | H7N9 | human | EPI_ISL_198730 | EPI_ISL_198730 | EPI_ISL_198730 | EPI_ISL_198730 | EPI_ISL_198730 | EPI_ISL_198730 | EPI_ISL_198730 | EPI_ISL_198730 |
| A/Hangzhou/35/2014 | H7N9 | human | EPI_ISL_164000 | EPI_ISL_164000 | EPI_ISL_164000 | EPI_ISL_164000 | EPI_ISL_164000 | EPI_ISL_164000 | EPI_ISL_164000 | EPI_ISL_164000 |
| A/Hangzhou/38/2014 | H7N9 | human | EPI_ISL_164001 | EPI_ISL_164001 | EPI_ISL_164001 | EPI_ISL_164001 | EPI_ISL_164001 | EPI_ISL_164001 | EPI_ISL_164001 | EPI_ISL_164001 |
| A/Hangzhou/39/2014 | H7N9 | human | EPI_ISL_164002 | EPI_ISL_164002 | EPI_ISL_164002 | EPI_ISL_164002 | EPI_ISL_164002 | EPI_ISL_164002 | EPI_ISL_164002 | EPI_ISL_164002 |
| A/Shenzhen/SP44/2014 | H7N9 | Human | KP414162 | KP414164 | KP414165 | KP414163 | KP414166 | KP414161 | KP414160 | KP414159 |
| A/Shenzhen/SP48/2014 | H7N9 | Human | KP414170 | KP414172 | KP414173 | KP414171 | KP414174 | KP414169 | KP414168 | KP414167 |
| A/Fujian/S03/2015 | H7N9 | Human | KY286427 | KY286429 | KY286430 | KY286428 | KY286431 | KY286426 | KY286425 | KY286424 |
| A/Hong_Kong/2550/2015 | H7N9 | human | EPI_ISL_170981 | EPI_ISL_170981 | EPI_ISL_170981 | EPI_ISL_170981 | EPI_ISL_170981 | EPI_ISL_170981 | EPI_ISL_170981 | EPI_ISL_170981 |
| A/Huai'an/001/2015 | H7N9 | Human | KP864456 | KP864447 | KX215924 | KX215934 | KX215943 | KX215954 | KX215964 | KX215974 |
| A/Shantou/2001/2015 | H7N9 | human | EPI_ISL_175280 | EPI_ISL_175280 | EPI_ISL_175280 | EPI_ISL_175280 | EPI_ISL_175280 |  | EPI_ISL_175280 |  |
| A/Fujian/8/2017 | H7N9 | human | EPI_ISL_257985 | EPI_ISL_257985 | EPI_ISL_257985 | EPI_ISL_257985 | EPI_ISL_257985 | EPI_ISL_257985 | EPI_ISL_257985 | EPI_ISL_257985 |
| A/Guangdong/17SF032/2017 | H7N9 | human | EPI_ISL_267758 | EPI_ISL_267758 | EPI_ISL_267758 | EPI_ISL_267758 | EPI_ISL_267758 | EPI_ISL_267758 | EPI_ISL_267758 | EPI_ISL_267758 |
| A/Jiangsu/11550/2017 | H7N9 | human | EPI_ISL_258005 | EPI_ISL_258005 | EPI_ISL_258005 | EPI_ISL_258005 | EPI_ISL_258005 | EPI_ISL_258005 | EPI_ISL_258005 | EPI_ISL_258005 |
| A/Hangzhou/40/2014 | H7N9 | human | EPI_ISL_164003 | EPI_ISL_164003 | EPI_ISL_164003 | EPI_ISL_164003 | EPI_ISL_164003 | EPI_ISL_164003 | EPI_ISL_164003 | EPI_ISL_164003 |
| A/Shantou/2002/2015 | H7N9 | human | EPI_ISL_175281 | EPI_ISL_175281 | EPI_ISL_175281 | EPI_ISL_175281 | EPI_ISL_175281 |  | EPI_ISL_175281 | EPI_ISL_175281 |
| A/Fujian/14/2017 | H7N9 | human | EPI_ISL_257982 | EPI_ISL_257982 | EPI_ISL_257982 | EPI_ISL_257982 | EPI_ISL_257982 | EPI_ISL_257982 | EPI_ISL_257982 | EPI_ISL_257982 |
| A/Shenzhen/SP49/2014 | H7N9 | Human | KP414178 | KP414180 | KP414181 | KP414179 | KP414182 | KP414177 | KP414176 | KP414175 |
| A/Shenzhen/SP58/2014 | H7N9 | Human | KP416598 | KP416600 | KP416601 | KP416599 | KP416602 | KP416597 | KP416596 | KP416595 |
| A/GD-42/2015/H7N9/2015-01-25 | H7N9 | human | EPI_ISL_198732 | EPI_ISL_198732 | EPI_ISL_198732 | EPI_ISL_198732 | EPI_ISL_198732 | EPI_ISL_198732 | EPI_ISL_198732 | EPI_ISL_198732 |
| A/Guangzhou/1/2014 | H7N9 | Human | KJ415822 |  |  |  |  |  |  |  |
| A/Guangzhou/2/2014 | H7N9 | Human | KJ415823 |  |  |  |  |  |  |  |
| A/GD-45/2015/H7N9/2015-01-26 | H7N9 | human | EPI_ISL_198733 | EPI_ISL_198733 | EPI_ISL_198733 | EPI_ISL_198733 | EPI_ISL_198733 | EPI_ISL_198733 | EPI_ISL_198733 | EPI_ISL_198733 |
| A/Hunan/02650/2016 | H7N9 | human | EPI_ISL_256214 | EPI_ISL_256214 | EPI_ISL_256214 | EPI_ISL_256214 | EPI_ISL_256214 | EPI_ISL_256214 | EPI_ISL_256214 | EPI_ISL_256214 |
| A/GD-62/2014/H7N9/2014-01-27 | H7N9 | human | EPI_ISL_198686 | EPI_ISL_198686 | EPI_ISL_198686 | EPI_ISL_198686 | EPI_ISL_198686 | EPI_ISL_198686 | EPI_ISL_198686 | EPI_ISL_198686 |
| A/GD-50/2015/H7N9/2015-01-27 | H7N9 | human | EPI_ISL_198736 | EPI_ISL_198736 | EPI_ISL_198736 | EPI_ISL_198736 | EPI_ISL_198736 | EPI_ISL_198736 | EPI_ISL_198736 | EPI_ISL_198736 |
| A/GD-54/2015/H7N9/2015-01-27 | H7N9 | human | EPI_ISL_198734 | EPI_ISL_198734 | EPI_ISL_198734 | EPI_ISL_198734 | EPI_ISL_198734 | EPI_ISL_198734 | EPI_ISL_198734 | EPI_ISL_198734 |
| A/GD-55/2015/H7N9/2015-01-27 | H7N9 | human | EPI_ISL_198735 | EPI_ISL_198735 | EPI_ISL_198735 | EPI_ISL_198735 | EPI_ISL_198735 | EPI_ISL_198735 | EPI_ISL_198735 | EPI_ISL_198735 |
| A/Anhui/13447/2017 | H7N9 | human | EPI_ISL_258028 | EPI_ISL_258028 | EPI_ISL_258028 | EPI_ISL_258028 | EPI_ISL_258028 | EPI_ISL_258028 | EPI_ISL_258028 | EPI_ISL_258028 |
| A/Liaoning/13454/2017 | H7N9 | human | EPI_ISL_258010 | EPI_ISL_258010 | EPI_ISL_258010 | EPI_ISL_258010 | EPI_ISL_258010 | EPI_ISL_258010 | EPI_ISL_258010 | EPI_ISL_258010 |
| A/Hong_Kong/2212982/2014 | H7N9 | human | EPI_ISL_154553 | EPI_ISL_154553 | EPI_ISL_154553 | EPI_ISL_154553 | EPI_ISL_154553 | EPI_ISL_154553 | EPI_ISL_154553 | EPI_ISL_154553 |
| A/GD-65/2014/H7N9/2014-01-29 | H7N9 | human | EPI_ISL_198687 | EPI_ISL_198687 | EPI_ISL_198687 | EPI_ISL_198687 | EPI_ISL_198687 | EPI_ISL_198687 | EPI_ISL_198687 | EPI_ISL_198687 |
| A/GD-66/2014/H7N9/2014-01-29 | H7N9 | human | EPI_ISL_198688 | EPI_ISL_198688 | EPI_ISL_198688 | EPI_ISL_198688 | EPI_ISL_198688 | EPI_ISL_198688 | EPI_ISL_198688 | EPI_ISL_198688 |
| A/Hangzhou/49/2014 | H7N9 | human | EPI_ISL_164004 | EPI_ISL_164004 | EPI_ISL_164004 | EPI_ISL_164004 | EPI_ISL_164004 | EPI_ISL_164004 | EPI_ISL_164004 | EPI_ISL_164004 |
| A/Jiangsu/11553/2017 | H7N9 | human | EPI_ISL_258004 | EPI_ISL_258004 | EPI_ISL_258004 | EPI_ISL_258004 | EPI_ISL_258004 | EPI_ISL_258004 | EPI_ISL_258004 | EPI_ISL_258004 |
| A/Jiangsu/11583/2017 | H7N9 | human | EPI_ISL_257998 | EPI_ISL_257998 | EPI_ISL_257998 | EPI_ISL_257998 | EPI_ISL_257998 | EPI_ISL_257998 | EPI_ISL_257998 | EPI_ISL_257998 |
| A/GD-69/2014/H7N9/2014-01-30 | H7N9 | human | EPI_ISL_198689 | EPI_ISL_198689 | EPI_ISL_198689 | EPI_ISL_198689 | EPI_ISL_198689 | EPI_ISL_198689 | EPI_ISL_198689 | EPI_ISL_198689 |
| A/GD-71/2014/H7N9/2014-01-30 | H7N9 | human | EPI_ISL_198691 | EPI_ISL_198691 | EPI_ISL_198691 | EPI_ISL_198691 | EPI_ISL_198691 | EPI_ISL_198691 | EPI_ISL_198691 | EPI_ISL_198691 |
| A/GD-81/2014/H7N9/2014-01-30 | H7N9 | human | EPI_ISL_198690 | EPI_ISL_198690 | EPI_ISL_198690 | EPI_ISL_198690 | EPI_ISL_198690 | EPI_ISL_198690 | EPI_ISL_198690 | EPI_ISL_198690 |
| A/Shenzhen/SP60/2014 | H7N9 | Human | KP416606 | KP416608 | KP416609 | KP416607 | KP416610 | KP416605 | KP416604 | KP416603 |
| A/GD-51/2015/H7N9/2015-01-30 | H7N9 | human | EPI_ISL_198737 | EPI_ISL_198737 | EPI_ISL_198737 | EPI_ISL_198737 | EPI_ISL_198737 | EPI_ISL_198737 | EPI_ISL_198737 | EPI_ISL_198737 |
| A/GD-74/2014/H7N9/2014-01-31 | H7N9 | human | EPI_ISL_198693 | EPI_ISL_198693 | EPI_ISL_198693 | EPI_ISL_198693 | EPI_ISL_198693 | EPI_ISL_198693 | EPI_ISL_198693 | EPI_ISL_198693 |
| A/GD-75/2014/H7N9/2014-01-31 | H7N9 | human | EPI_ISL_198692 | EPI_ISL_198692 | EPI_ISL_198692 | EPI_ISL_198692 | EPI_ISL_198692 | EPI_ISL_198692 | EPI_ISL_198692 | EPI_ISL_198692 |
| A/Anhui/13434/2017 | H7N9 | human | EPI_ISL_258021 | EPI_ISL_258021 | EPI_ISL_258021 | EPI_ISL_258021 | EPI_ISL_258021 | EPI_ISL_258021 | EPI_ISL_258021 | EPI_ISL_258021 |
| A/Jiangsu/11585/2017 | H7N9 | human | EPI_ISL_257991 | EPI_ISL_257991 | EPI_ISL_257991 | EPI_ISL_257991 | EPI_ISL_257991 | EPI_ISL_257991 | EPI_ISL_257991 | EPI_ISL_257991 |
| A/Jiangsu/11556/2017 | H7N9 | human | EPI_ISL_258000 | EPI_ISL_258000 | EPI_ISL_258000 | EPI_ISL_258000 | EPI_ISL_258000 | EPI_ISL_258000 | EPI_ISL_258000 | EPI_ISL_258000 |
| A/GD-82/2014/H7N9/2014-02-02 | H7N9 | human | EPI_ISL_198694 | EPI_ISL_198694 | EPI_ISL_198694 | EPI_ISL_198694 | EPI_ISL_198694 | EPI_ISL_198694 | EPI_ISL_198694 | EPI_ISL_198694 |
| A/Shaoxing/70/2014 | H7N9 | human | EPI_ISL_164005 | EPI_ISL_164005 | EPI_ISL_164005 | EPI_ISL_164005 | EPI_ISL_164005 | EPI_ISL_164005 | EPI_ISL_164005 | EPI_ISL_164005 |
| A/GD-53/2015/H7N9/2015-02-03 | H7N9 | human | EPI_ISL_198738 | EPI_ISL_198738 | EPI_ISL_198738 | EPI_ISL_198738 | EPI_ISL_198738 | EPI_ISL_198738 | EPI_ISL_198738 | EPI_ISL_198738 |
| A/GD-57/2015/H7N9/2015-02-03 | H7N9 | human | EPI_ISL_198739 | EPI_ISL_198739 | EPI_ISL_198739 | EPI_ISL_198739 | EPI_ISL_198739 | EPI_ISL_198739 | EPI_ISL_198739 | EPI_ISL_198739 |
| A/Huai'an/002/2015 | H7N9 | Human | KP864460 | KP864444 | KX215925 | KX215935 | KX215944 | KX215955 | KX215965 | KX215975 |
| A/Fujian/11/2017 | H7N9 | human | EPI_ISL_257984 | EPI_ISL_257984 | EPI_ISL_257984 | EPI_ISL_257984 | EPI_ISL_257984 | EPI_ISL_257984 | EPI_ISL_257984 | EPI_ISL_257984 |
| A/Guangxi/2/2017 | H7N9 | human | EPI_ISL_268515 |  | EPI_ISL_268515 | EPI_ISL_268515 | EPI_ISL_268515 | EPI_ISL_268515 | EPI_ISL_268515 | EPI_ISL_268515 |
| A/Jiangsu/11554/2017 | H7N9 | human | EPI_ISL_257999 | EPI_ISL_257999 | EPI_ISL_257999 | EPI_ISL_257999 | EPI_ISL_257999 | EPI_ISL_257999 | EPI_ISL_257999 | EPI_ISL_257999 |
| A/Jiangsu/11555/2017 | H7N9 | human | EPI_ISL_258034 | EPI_ISL_258034 | EPI_ISL_258034 | EPI_ISL_258034 | EPI_ISL_258034 | EPI_ISL_258034 | EPI_ISL_258034 | EPI_ISL_258034 |
| A/Jiangsu/11586/2017 | H7N9 | human | EPI_ISL_257997 | EPI_ISL_257997 | EPI_ISL_257997 | EPI_ISL_257997 | EPI_ISL_257997 | EPI_ISL_257997 | EPI_ISL_257997 | EPI_ISL_257997 |
| A/Jiangsu/11587/2017 | H7N9 | human | EPI_ISL_257992 | EPI_ISL_257992 | EPI_ISL_257992 | EPI_ISL_257992 | EPI_ISL_257992 | EPI_ISL_257992 | EPI_ISL_257992 | EPI_ISL_257992 |
| A/Jiangsu/11588/2017 | H7N9 | human | EPI_ISL_257996 | EPI_ISL_257996 | EPI_ISL_257996 | EPI_ISL_257996 | EPI_ISL_257996 | EPI_ISL_257996 | EPI_ISL_257996 | EPI_ISL_257996 |
| A/Jiangxi/10683/2017 | H7N9 | human | EPI_ISL_258006 | EPI_ISL_258006 | EPI_ISL_258006 | EPI_ISL_258006 | EPI_ISL_258006 | EPI_ISL_258006 | EPI_ISL_258006 | EPI_ISL_258006 |
| A/GD-69/2015/H7N9/2015-02-04 | H7N9 | human | EPI_ISL_198740 | EPI_ISL_198740 | EPI_ISL_198740 | EPI_ISL_198740 | EPI_ISL_198740 | EPI_ISL_198740 | EPI_ISL_198740 | EPI_ISL_198740 |
| A/Changsha/26/2017 | H7N9 | Human | MF370246 | MF370248 | MF370249 | MF370247 | MF370250 | MF370245 | MF370244 | MF370243 |
| A/Fujian/12/2017 | H7N9 | human | EPI_ISL_257983 | EPI_ISL_257983 | EPI_ISL_257983 | EPI_ISL_257983 | EPI_ISL_257983 | EPI_ISL_257983 | EPI_ISL_257983 | EPI_ISL_257983 |
| A/Henan/11157/2017 | H7N9 | human | EPI_ISL_258003 | EPI_ISL_258003 | EPI_ISL_258003 | EPI_ISL_258003 | EPI_ISL_258003 | EPI_ISL_258003 | EPI_ISL_258003 | EPI_ISL_258003 |
| A/Shenzhen/SP62/2014 | H7N9 | Human | KP416622 | KP416624 | KP416625 | KP416623 | KP416626 | KP416621 | KP416620 | KP416619 |
| A/Anhui/13449/2017 | H7N9 | human | EPI_ISL_258029 | EPI_ISL_258029 | EPI_ISL_258029 | EPI_ISL_258029 | EPI_ISL_258029 | EPI_ISL_258029 | EPI_ISL_258029 | EPI_ISL_258029 |
| A/GD-98/2014/H7N9/2014-02-06 | H7N9 | human | EPI_ISL_198695 | EPI_ISL_198695 | EPI_ISL_198695 | EPI_ISL_198695 | EPI_ISL_198695 | EPI_ISL_198695 | EPI_ISL_198695 | EPI_ISL_198695 |
| A/Jiangsu/11589/2017 | H7N9 | human | EPI_ISL_257993 | EPI_ISL_257993 | EPI_ISL_257993 | EPI_ISL_257993 | EPI_ISL_257993 | EPI_ISL_257993 | EPI_ISL_257993 | EPI_ISL_257993 |
| A/GD-101/2014/H7N9/2014-02-07 | H7N9 | human | EPI_ISL_198696 | EPI_ISL_198696 | EPI_ISL_198696 | EPI_ISL_198696 | EPI_ISL_198696 | EPI_ISL_198696 | EPI_ISL_198696 | EPI_ISL_198696 |
| A/GD-72/2015/H7N9/2015-02-07 | H7N9 | human | EPI_ISL_198741 | EPI_ISL_198741 | EPI_ISL_198741 | EPI_ISL_198741 | EPI_ISL_198741 | EPI_ISL_198741 | EPI_ISL_198741 | EPI_ISL_198741 |
| A/Shantou/2010/2015 | H7N9 | human | EPI_ISL_175286 |  | EPI_ISL_175286 | EPI_ISL_175286 | EPI_ISL_175286 |  | EPI_ISL_175286 | EPI_ISL_175286 |
| A/Henan/11158/2017 | H7N9 | human | EPI_ISL_258009 | EPI_ISL_258009 | EPI_ISL_258009 | EPI_ISL_258009 | EPI_ISL_258009 | EPI_ISL_258009 | EPI_ISL_258009 | EPI_ISL_258009 |
| A/Jiangsu/11557/2017 | H7N9 | human | EPI_ISL_258001 | EPI_ISL_258001 | EPI_ISL_258001 | EPI_ISL_258001 | EPI_ISL_258001 | EPI_ISL_258001 | EPI_ISL_258001 | EPI_ISL_258001 |
| A/Jiangsu/11590/2017 | H7N9 | human | EPI_ISL_257995 | EPI_ISL_257995 | EPI_ISL_257995 | EPI_ISL_257995 | EPI_ISL_257995 | EPI_ISL_257995 | EPI_ISL_257995 | EPI_ISL_257995 |
| A/Zhejiang/LS01/2014 | H7N9 | Human | KM374042 | KM374044 | KM374045 | KM374043 | KM374046 | KM374041 | KM374040 | KM374039 |
| A/GD-68/2015/H7N9/2015-02-08 | H7N9 | human | EPI_ISL_198742 | EPI_ISL_198742 | EPI_ISL_198742 | EPI_ISL_198742 | EPI_ISL_198742 | EPI_ISL_198742 | EPI_ISL_198742 | EPI_ISL_198742 |
| A/Henan/11159/2017 | H7N9 | human | EPI_ISL_258008 | EPI_ISL_258008 | EPI_ISL_258008 | EPI_ISL_258008 | EPI_ISL_258008 | EPI_ISL_258008 | EPI_ISL_258008 | EPI_ISL_258008 |
| A/Hangzhou/112/2014 | H7N9 | human | EPI_ISL_164006 | EPI_ISL_164006 | EPI_ISL_164006 | EPI_ISL_164006 | EPI_ISL_164006 | EPI_ISL_164006 | EPI_ISL_164006 | EPI_ISL_164006 |
| A/Shantou/2003/2015 | H7N9 | human | EPI_ISL_173403 | EPI_ISL_173403 | EPI_ISL_173403 | EPI_ISL_173403 | EPI_ISL_173403 |  | EPI_ISL_173403 | EPI_ISL_173403 |
| A/Guangdong/17SF037/2017 | H7N9 | human | EPI_ISL_267760 | EPI_ISL_267760 | EPI_ISL_267760 | EPI_ISL_267760 | EPI_ISL_267760 | EPI_ISL_267760 | EPI_ISL_267760 | EPI_ISL_267760 |
| A/Yunnan/0129/2017 | H7N9 | Human | KY910837 | KY910839 | KY910838 | KY910840 | KY910841 | KY910842 | KY910843 | KY910844 |
| A/Yunnan/13500/2017 | H7N9 | human | EPI_ISL_258032 | EPI_ISL_258032 | EPI_ISL_258032 | EPI_ISL_258032 | EPI_ISL_258032 | EPI_ISL_258032 | EPI_ISL_258032 | EPI_ISL_258032 |
| A/GD-103/2014/H7N9/2014-02-10 | H7N9 | human | EPI_ISL_198697 | EPI_ISL_198697 | EPI_ISL_198697 | EPI_ISL_198697 | EPI_ISL_198697 | EPI_ISL_198697 | EPI_ISL_198697 | EPI_ISL_198697 |
| A/Hangzhou/118/2014 | H7N9 | human | EPI_ISL_164007 | EPI_ISL_164007 | EPI_ISL_164007 | EPI_ISL_164007 | EPI_ISL_164007 | EPI_ISL_164007 | EPI_ISL_164007 | EPI_ISL_164007 |
| A/GD-76/2015/H7N9/2015-02-10 | H7N9 | human | EPI_ISL_198745 | EPI_ISL_198745 | EPI_ISL_198745 | EPI_ISL_198745 | EPI_ISL_198745 | EPI_ISL_198745 | EPI_ISL_198745 | EPI_ISL_198745 |
| A/GD-80/2015/H7N9/2015-02-10 | H7N9 | human | EPI_ISL_198743 | EPI_ISL_198743 | EPI_ISL_198743 | EPI_ISL_198743 | EPI_ISL_198743 | EPI_ISL_198743 | EPI_ISL_198743 | EPI_ISL_198743 |
| A/GD-81/2015/H7N9/2015-02-10 | H7N9 | human | EPI_ISL_198744 | EPI_ISL_198744 | EPI_ISL_198744 | EPI_ISL_198744 | EPI_ISL_198744 | EPI_ISL_198744 | EPI_ISL_198744 | EPI_ISL_198744 |
| A/GD-104/2014/H7N9/2014-02-11 | H7N9 | human | EPI_ISL_198698 | EPI_ISL_198698 | EPI_ISL_198698 | EPI_ISL_198698 | EPI_ISL_198698 | EPI_ISL_198698 | EPI_ISL_198698 | EPI_ISL_198698 |
| A/GD-105/2014/H7N9/2014-02-11 | H7N9 | human | EPI_ISL_198699 | EPI_ISL_198699 | EPI_ISL_198699 | EPI_ISL_198699 | EPI_ISL_198699 | EPI_ISL_198699 | EPI_ISL_198699 | EPI_ISL_198699 |
| A/Malaysia/228/2014 | H7N9 | human | EPI_ISL_157350 | EPI_ISL_157291 |  |  |  |  |  |  |
| A/Hong_Kong/3263/2014 | H7N9 | human | EPI_ISL_156471 | EPI_ISL_156471 | EPI_ISL_156471 | EPI_ISL_156471 | EPI_ISL_156471 | EPI_ISL_156471 | EPI_ISL_156471 | EPI_ISL_156471 |
| A/Hong_Kong/VB16021618/2016 | H7N9 | human | EPI_ISL_212471 | EPI_ISL_212471 | EPI_ISL_212471 | EPI_ISL_212471 | EPI_ISL_212471 | EPI_ISL_212471 | EPI_ISL_212471 | EPI_ISL_212471 |
| A/environment/GD-CH_588/H7N9/2014 | H7N9 | human |  |  |  |  |  | EPI_ISL_198432 |  |  |
| A/GD-109/2014/H7N9/2014-02-13 | H7N9 | human |  |  |  |  |  | EPI_ISL_198700 | EPI_ISL_198700 | EPI_ISL_198700 |
| A/GD-110/2014/H7N9/2014-02-13 | H7N9 | human | EPI_ISL_198701 | EPI_ISL_198701 | EPI_ISL_198701 | EPI_ISL_198701 | EPI_ISL_198701 | EPI_ISL_198701 | EPI_ISL_198701 | EPI_ISL_198701 |
| A/Huai'an/062/2014 | H7N9 | Human | KP864455 | KP864449 | KX215926 | KX215936 | KX215945 | KX215956 | KX215966 | KX215976 |
| A/Anhui/13424/2017 | H7N9 | human | EPI_ISL_258013 | EPI_ISL_258013 | EPI_ISL_258013 | EPI_ISL_258013 | EPI_ISL_258013 | EPI_ISL_258013 | EPI_ISL_258013 | EPI_ISL_258013 |
| A/Anhui/13425/2017 | H7N9 | human | EPI_ISL_258014 | EPI_ISL_258014 | EPI_ISL_258014 | EPI_ISL_258014 | EPI_ISL_258014 | EPI_ISL_258014 | EPI_ISL_258014 | EPI_ISL_258014 |
| A/Anhui/13426/2017 | H7N9 | human | EPI_ISL_258015 | EPI_ISL_258015 | EPI_ISL_258015 | EPI_ISL_258015 | EPI_ISL_258015 | EPI_ISL_258015 | EPI_ISL_258015 | EPI_ISL_258015 |
| A/Anhui/13427/2017 | H7N9 | human | EPI_ISL_258016 | EPI_ISL_258016 | EPI_ISL_258016 | EPI_ISL_258016 | EPI_ISL_258016 | EPI_ISL_258016 | EPI_ISL_258016 | EPI_ISL_258016 |
| A/Shenzhen/SP75/2014 | H7N9 | Human | KP416614 | KP416616 | KP416617 | KP416615 | KP416618 | KP416613 | KP416612 | KP416611 |
| A/Shantou/2004/2015 | H7N9 | human | EPI_ISL_175282 | EPI_ISL_175282 | EPI_ISL_175282 | EPI_ISL_175282 | EPI_ISL_175282 |  | EPI_ISL_175282 | EPI_ISL_175282 |
| A/Anhui/13428/2017 | H7N9 | human | EPI_ISL_258017 | EPI_ISL_258017 | EPI_ISL_258017 | EPI_ISL_258017 | EPI_ISL_258017 | EPI_ISL_258017 | EPI_ISL_258017 | EPI_ISL_258017 |
| A/Guangdong/17SF039/2017 | H7N9 | human | EPI_ISL_267761 | EPI_ISL_267761 | EPI_ISL_267761 | EPI_ISL_267761 | EPI_ISL_267761 | EPI_ISL_267761 | EPI_ISL_267761 | EPI_ISL_267761 |
| A/Shantou/2009/2015 | H7N9 | human | EPI_ISL_174514 | EPI_ISL_174514 | EPI_ISL_174514 | EPI_ISL_174514 | EPI_ISL_174514 |  |  | EPI_ISL_174514 |
| A/Fujian/13558/2017 | H7N9 | human | EPI_ISL_258033 | EPI_ISL_258033 | EPI_ISL_258033 | EPI_ISL_258033 | EPI_ISL_258033 | EPI_ISL_258033 | EPI_ISL_258033 | EPI_ISL_258033 |
| A/Fujian/17/2017 | H7N9 | human | EPI_ISL_257981 | EPI_ISL_257981 | EPI_ISL_257981 | EPI_ISL_257981 | EPI_ISL_257981 | EPI_ISL_257981 | EPI_ISL_257981 | EPI_ISL_257981 |
| A/GD-112/2014/H7N9/2014-02-18 | H7N9 | human | EPI_ISL_198702 | EPI_ISL_198702 | EPI_ISL_198702 | EPI_ISL_198702 | EPI_ISL_198702 | EPI_ISL_198702 | EPI_ISL_198702 | EPI_ISL_198702 |
| A/GD-114/2014/H7N9/2014-02-18 | H7N9 | human | EPI_ISL_198703 | EPI_ISL_198703 | EPI_ISL_198703 | EPI_ISL_198703 | EPI_ISL_198703 | EPI_ISL_198703 | EPI_ISL_198703 | EPI_ISL_198703 |
| A/Guangxi/13452/2017 | H7N9 | human | EPI_ISL_268499 | EPI_ISL_268499 | EPI_ISL_268499 | EPI_ISL_268499 | EPI_ISL_268499 | EPI_ISL_268499 | EPI_ISL_268499 | EPI_ISL_268499 |
| A/GD-119/2014/H7N9/2014-02-19 | H7N9 | human | EPI_ISL_198704 | EPI_ISL_198704 | EPI_ISL_198704 | EPI_ISL_198704 | EPI_ISL_198704 | EPI_ISL_198704 | EPI_ISL_198704 | EPI_ISL_198704 |
| A/GD-120/2014/H7N9/2014-02-19 | H7N9 | human | EPI_ISL_198705 | EPI_ISL_198705 | EPI_ISL_198705 | EPI_ISL_198705 | EPI_ISL_198705 | EPI_ISL_198705 | EPI_ISL_198705 | EPI_ISL_198705 |
| A/GD-121/2014/H7N9/2014-02-19 | H7N9 | human | EPI_ISL_198706 | EPI_ISL_198706 | EPI_ISL_198706 | EPI_ISL_198706 | EPI_ISL_198706 | EPI_ISL_198706 | EPI_ISL_198706 | EPI_ISL_198706 |
| A/Sichuan/12218/2017 | H7N9 | human | EPI_ISL_257986 | EPI_ISL_257986 | EPI_ISL_257986 | EPI_ISL_257986 | EPI_ISL_257986 | EPI_ISL_257986 | EPI_ISL_257986 | EPI_ISL_257986 |
| A/Sichuan/12219/2017 | H7N9 | human | EPI_ISL_257987 | EPI_ISL_257987 | EPI_ISL_257987 | EPI_ISL_257987 | EPI_ISL_257987 | EPI_ISL_257987 | EPI_ISL_257987 | EPI_ISL_257987 |
| A/Anhui/13444/2017 | H7N9 | human | EPI_ISL_258026 | EPI_ISL_258026 | EPI_ISL_258026 | EPI_ISL_258026 | EPI_ISL_258026 | EPI_ISL_258026 | EPI_ISL_258026 | EPI_ISL_258026 |
| A/Hubei/11944/2017 | H7N9 | human | EPI_ISL_257988 | EPI_ISL_257988 | EPI_ISL_257988 | EPI_ISL_257988 | EPI_ISL_257988 | EPI_ISL_257988 | EPI_ISL_257988 | EPI_ISL_257988 |
| A/Hubei/11950/2017 | H7N9 | human | EPI_ISL_257989 | EPI_ISL_257989 | EPI_ISL_257989 | EPI_ISL_257989 | EPI_ISL_257989 | EPI_ISL_257989 | EPI_ISL_257989 | EPI_ISL_257989 |
| A/Hubei/11951/2017 | H7N9 | human | EPI_ISL_257990 | EPI_ISL_257990 | EPI_ISL_257990 | EPI_ISL_257990 | EPI_ISL_257990 | EPI_ISL_257990 | EPI_ISL_257990 | EPI_ISL_257990 |
| A/Shantou/2005/2015 | H7N9 | human | EPI_ISL_175283 | EPI_ISL_175283 | EPI_ISL_175283 | EPI_ISL_175283 | EPI_ISL_175283 |  |  |  |
| A/Shantou/2007/2015 | H7N9 | human | EPI_ISL_175285 | EPI_ISL_175285 |  |  |  |  |  |  |
| A/Henan/13705/2017 | H7N9 | human | EPI_ISL_258030 | EPI_ISL_258030 | EPI_ISL_258030 | EPI_ISL_258030 | EPI_ISL_258030 | EPI_ISL_258030 | EPI_ISL_258030 | EPI_ISL_258030 |
| A/Henan/13706/2017 | H7N9 | human | EPI_ISL_258031 | EPI_ISL_258031 | EPI_ISL_258031 | EPI_ISL_258031 | EPI_ISL_258031 | EPI_ISL_258031 | EPI_ISL_258031 | EPI_ISL_258031 |
| A/GD-116/2014/H7N9/2014-02-22 | H7N9 | human |  | EPI_ISL_198707 |  |  |  | EPI_ISL_198707 | EPI_ISL_198707 | EPI_ISL_198707 |
| A/GD-117/2014/H7N9/2014-02-22 | H7N9 | human | EPI_ISL_198708 | EPI_ISL_198708 | EPI_ISL_198708 | EPI_ISL_198708 | EPI_ISL_198708 | EPI_ISL_198708 | EPI_ISL_198708 | EPI_ISL_198708 |
| A/Hong_Kong/8130773/2015 | H7N9 | human | EPI_ISL_173033 | EPI_ISL_173033 | EPI_ISL_173033 | EPI_ISL_173033 | EPI_ISL_173033 | EPI_ISL_173033 | EPI_ISL_173033 | EPI_ISL_173033 |
| A/Guangdong/17SF033/2017 | H7N9 | human | EPI_ISL_267759 | EPI_ISL_267759 | EPI_ISL_267759 | EPI_ISL_267759 | EPI_ISL_267759 | EPI_ISL_267759 | EPI_ISL_267759 | EPI_ISL_267759 |
| A/Guangxi/3/2017 | H7N9 | human | EPI_ISL_268512 | EPI_ISL_268512 | EPI_ISL_268512 | EPI_ISL_268512 | EPI_ISL_268512 | EPI_ISL_268512 | EPI_ISL_268512 | EPI_ISL_268512 |
| A/Guangxi/13450/2017 | H7N9 | human | EPI_ISL_268498 | EPI_ISL_268498 | EPI_ISL_268498 | EPI_ISL_268498 | EPI_ISL_268498 | EPI_ISL_268498 | EPI_ISL_268498 | EPI_ISL_268498 |
| A/Huai'an/065/2014 | H7N9 | Human | KP864457 | KP864458 | KX215927 | KX215937 | KX215946 | KX215957 | KX215967 | KX215977 |
| A/Zhejiang/7/2014 | H7N9 | human | EPI_ISL_163320 | EPI_ISL_163320 | EPI_ISL_163320 | EPI_ISL_163320 | EPI_ISL_163320 | EPI_ISL_163320 | EPI_ISL_163320 | EPI_ISL_163320 |
| A/Shantou/2006/2015 | H7N9 | human | EPI_ISL_175284 | EPI_ISL_175284 | EPI_ISL_175284 | EPI_ISL_175284 | EPI_ISL_175284 |  |  | EPI_ISL_175284 |
| A/GD-124/2015/H7N9/2015-02-26 | H7N9 | human | EPI_ISL_198748 | EPI_ISL_198748 | EPI_ISL_198748 | EPI_ISL_198748 | EPI_ISL_198748 | EPI_ISL_198748 | EPI_ISL_198748 | EPI_ISL_198748 |
| A/GD-91/2015/H7N9/2015-02-26 | H7N9 | human | EPI_ISL_198747 | EPI_ISL_198747 | EPI_ISL_198747 | EPI_ISL_198747 | EPI_ISL_198747 | EPI_ISL_198747 | EPI_ISL_198747 | EPI_ISL_198747 |
| A/GD-92/2015/H7N9/2015-02-26 | H7N9 | human | EPI_ISL_198746 | EPI_ISL_198746 | EPI_ISL_198746 | EPI_ISL_198746 | EPI_ISL_198746 | EPI_ISL_198746 | EPI_ISL_198746 | EPI_ISL_198746 |
| A/Minhang/S01/2013 | H7N9 | Human | KY286419 | KY286421 | KY286422 | KY286420 | KY286423 | KY286418 | KY286417 | KY286416 |
| A/Zhejiang/8/2014 | H7N9 | human | EPI_ISL_163321 | EPI_ISL_163321 | EPI_ISL_163321 | EPI_ISL_163321 | EPI_ISL_163321 | EPI_ISL_163321 | EPI_ISL_163321 | EPI_ISL_163321 |
| A/Guangxi/18891/2017 | H7N9 | human | EPI_ISL_268501 | EPI_ISL_268501 | EPI_ISL_268501 | EPI_ISL_268501 | EPI_ISL_268501 | EPI_ISL_268501 | EPI_ISL_268501 | EPI_ISL_268501 |
| A/Guangxi/18892/2017 | H7N9 | human | EPI_ISL_268502 | EPI_ISL_268502 | EPI_ISL_268502 | EPI_ISL_268502 | EPI_ISL_268502 | EPI_ISL_268502 | EPI_ISL_268502 | EPI_ISL_268502 |
| A/Guangxi/4/2017 | H7N9 | human | EPI_ISL_268524 | EPI_ISL_268524 | EPI_ISL_268524 | EPI_ISL_268524 | EPI_ISL_268524 | EPI_ISL_268524 | EPI_ISL_268524 | EPI_ISL_268524 |
| A/Guangxi/18888/2017 | H7N9 | human | EPI_ISL_268500 | EPI_ISL_268500 | EPI_ISL_268500 | EPI_ISL_268500 | EPI_ISL_268500 | EPI_ISL_268500 | EPI_ISL_268500 | EPI_ISL_268500 |
| A/Guangxi/5/2017 | H7N9 | human | EPI_ISL_268523 | EPI_ISL_268523 | EPI_ISL_268523 | EPI_ISL_268523 | EPI_ISL_268523 | EPI_ISL_268523 | EPI_ISL_268523 | EPI_ISL_268523 |
| A/GD-123/2014/H7N9/2014-03-01 | H7N9 | human | EPI_ISL_198709 | EPI_ISL_198709 | EPI_ISL_198709 | EPI_ISL_198709 | EPI_ISL_198709 | EPI_ISL_198709 | EPI_ISL_198709 | EPI_ISL_198709 |
| A/Hong_Kong/4495/2014 | H7N9 | human | EPI_ISL_157702 | EPI_ISL_157702 | EPI_ISL_157702 | EPI_ISL_157702 | EPI_ISL_157702 | EPI_ISL_157702 | EPI_ISL_157702 | EPI_ISL_157702 |
| A/Shantou/2011/2015 | H7N9 | human | EPI_ISL_175287 | EPI_ISL_175287 |  | EPI_ISL_175287 |  |  |  | EPI_ISL_175287 |
| A/Huai'an/074/2014 | H7N9 | Human | KP864459 | KP864446 | KX215928 | KX215938 | KX215947 | KX215958 | KX215968 | KX215978 |
| A/Guangxi/18895/2017 | H7N9 | human | EPI_ISL_268503 | EPI_ISL_268503 | EPI_ISL_268503 | EPI_ISL_268503 | EPI_ISL_268503 | EPI_ISL_268503 | EPI_ISL_268503 | EPI_ISL_268503 |
| A/Guangxi/6/2017 | H7N9 | human | EPI_ISL_268525 | EPI_ISL_268525 | EPI_ISL_268525 | EPI_ISL_268525 | EPI_ISL_268525 | EPI_ISL_268525 | EPI_ISL_268525 | EPI_ISL_268525 |
| A/Chongqing/1/2017 | H7N9 | human | EPI_ISL_257341 | EPI_ISL_257341 |  |  |  |  |  |  |
| A/Guangxi/18897/2017 | H7N9 | human | EPI_ISL_268504 | EPI_ISL_268504 | EPI_ISL_268504 | EPI_ISL_268504 | EPI_ISL_268504 | EPI_ISL_268504 | EPI_ISL_268504 | EPI_ISL_268504 |
| A/Guangxi/7/2017 | H7N9 | human | EPI_ISL_268526 | EPI_ISL_268526 | EPI_ISL_268526 | EPI_ISL_268526 | EPI_ISL_268526 | EPI_ISL_268526 | EPI_ISL_268526 | EPI_ISL_268526 |
| A/Henan/14905/2017 | H7N9 | human | EPI_ISL_258036 | EPI_ISL_258036 | EPI_ISL_258036 | EPI_ISL_258036 | EPI_ISL_258036 | EPI_ISL_258036 | EPI_ISL_258036 | EPI_ISL_258036 |
| A/Henan/15337/2017 | H7N9 | human | EPI_ISL_258035 | EPI_ISL_258035 | EPI_ISL_258035 | EPI_ISL_258035 | EPI_ISL_258035 | EPI_ISL_258035 | EPI_ISL_258035 | EPI_ISL_258035 |
| A/Shanghai/02/2013 | H7N9 | Human | KF021597 | KF021599 | KF021600 | KF021598 | KF021601 | KF021596 | KF021595 | KF021594 |
| A/Shanghai/4664T/2013 | H7N9 | Human | KC853228 | KC853231 | KC853227 | KC853225 | KC853232 | KC853226 | KC853230 | KC853229 |
| A/GD-95/2015/H7N9/2015-03-05 | H7N9 | human | EPI_ISL_198749 | EPI_ISL_198749 | EPI_ISL_198749 | EPI_ISL_198749 | EPI_ISL_198749 | EPI_ISL_198749 | EPI_ISL_198749 | EPI_ISL_198749 |
| A/GD-124/2014/H7N9/2014-03-06 | H7N9 | human | EPI_ISL_198710 | EPI_ISL_198710 | EPI_ISL_198710 | EPI_ISL_198710 | EPI_ISL_198710 | EPI_ISL_198710 | EPI_ISL_198710 | EPI_ISL_198710 |
| A/Guangdong/17SF059/2017 | H7N9 | human | EPI_ISL_267762 | EPI_ISL_267762 | EPI_ISL_267762 | EPI_ISL_267762 | EPI_ISL_267762 | EPI_ISL_267762 | EPI_ISL_267762 | EPI_ISL_267762 |
| A/Hong_Kong/VB17037915/2017 | H7N9 | human | EPI_ISL_253626 | EPI_ISL_253626 | EPI_ISL_253626 | EPI_ISL_253626 | EPI_ISL_253626 | EPI_ISL_253626 | EPI_ISL_253626 | EPI_ISL_253626 |
| A/GD-125/2014/H7N9/2014-03-09 | H7N9 | human | EPI_ISL_198711 | EPI_ISL_198711 | EPI_ISL_198711 | EPI_ISL_198711 | EPI_ISL_198711 | EPI_ISL_198711 | EPI_ISL_198711 | EPI_ISL_198711 |
| A/GD-126/2014/H7N9/2014-03-10 | H7N9 | human | EPI_ISL_198712 | EPI_ISL_198712 | EPI_ISL_198712 | EPI_ISL_198712 | EPI_ISL_198712 | EPI_ISL_198712 | EPI_ISL_198712 | EPI_ISL_198712 |
| A/Shantou/1001/2014 | H7N9 | human | EPI_ISL_162618 | EPI_ISL_162618 | EPI_ISL_162618 | EPI_ISL_162618 | EPI_ISL_162618 | EPI_ISL_162618 | EPI_ISL_162618 | EPI_ISL_162618 |
| A/GD-120/2015/H7N9/2015-03-10 | H7N9 | human | EPI_ISL_198750 | EPI_ISL_198750 | EPI_ISL_198750 | EPI_ISL_198750 | EPI_ISL_198750 | EPI_ISL_198750 | EPI_ISL_198750 | EPI_ISL_198750 |
| A/Shantou/2008/2015 | H7N9 | human | EPI_ISL_174513 | EPI_ISL_174513 | EPI_ISL_174513 | EPI_ISL_174513 | EPI_ISL_174513 |  | EPI_ISL_174513 | EPI_ISL_174513 |
| A/Guangdong/17SF062/2017 | H7N9 | human | EPI_ISL_267763 | EPI_ISL_267763 | EPI_ISL_267763 | EPI_ISL_267763 | EPI_ISL_267763 | EPI_ISL_267763 | EPI_ISL_267763 | EPI_ISL_267763 |
| A/Shenzhen/SP113/2014 | H7N9 | Human | KP416630 | KP416632 | KP416633 | KP416631 | KP416634 | KP416629 | KP416628 | KP416627 |
| A/Changsha/41/2017 | H7N9 | Human | MF370251 | MF370252 | MF370253 |  | MF370254 |  |  |  |
| A/Guangdong/17SF064/2017 | H7N9 | human | EPI_ISL_267764 | EPI_ISL_267764 | EPI_ISL_267764 | EPI_ISL_267764 | EPI_ISL_267764 | EPI_ISL_267764 | EPI_ISL_267764 | EPI_ISL_267764 |
| A/Guangxi/18902/2017 | H7N9 | human | EPI_ISL_268505 | EPI_ISL_268505 | EPI_ISL_268505 | EPI_ISL_268505 | EPI_ISL_268505 | EPI_ISL_268505 | EPI_ISL_268505 | EPI_ISL_268505 |
| A/Chongqing/2/2017 | H7N9 | human | EPI_ISL_257729 | EPI_ISL_257729 |  |  |  |  |  |  |
| A/Changsha/44/2017 | H7N9 | Human | MF370255 | MF370256 | MF370257 |  | MF370258 |  |  |  |
| A/Guangxi/18906/2017 | H7N9 | human | EPI_ISL_268506 | EPI_ISL_268506 | EPI_ISL_268506 | EPI_ISL_268506 | EPI_ISL_268506 | EPI_ISL_268506 | EPI_ISL_268506 | EPI_ISL_268506 |
| A/Shenzhen/SP116/2014 | H7N9 | Human | KP416638 | KP416640 | KP416641 | KP416639 | KP416642 | KP416637 | KP416636 | KP416635 |
| A/Guangxi/18908/2017 | H7N9 | human | EPI_ISL_268507 | EPI_ISL_268507 | EPI_ISL_268507 | EPI_ISL_268507 | EPI_ISL_268507 | EPI_ISL_268507 | EPI_ISL_268507 | EPI_ISL_268507 |
| A/Hong_Kong/8113530/2014 | H7N9 | human | EPI_ISL_157703 | EPI_ISL_157703 | EPI_ISL_157703 | EPI_ISL_157703 | EPI_ISL_157703 | EPI_ISL_157703 | EPI_ISL_157703 | EPI_ISL_157703 |
| A/Changsha/58/2017 | H7N9 | Human | MF370259 | MF370260 | MF370261 |  | MF370262 |  |  |  |
| A/Shanghai/07/2013 | H7N9 | human | EPI_ISL_141160 | EPI_ISL_141160 | EPI_ISL_141160 | EPI_ISL_141160 | EPI_ISL_141160 | EPI_ISL_141160 | EPI_ISL_141160 | EPI_ISL_141160 |
| A/Shenzhen/SP118/2014 | H7N9 | Human | KP416646 | KP416648 | KP416649 | KP416647 | KP416650 | KP416645 | KP416644 | KP416643 |
| A/Guangxi/18910/2017 | H7N9 | human | EPI_ISL_268508 | EPI_ISL_268508 | EPI_ISL_268508 | EPI_ISL_268508 | EPI_ISL_268508 | EPI_ISL_268508 | EPI_ISL_268508 | EPI_ISL_268508 |
| A/Guangxi/18911/2017 | H7N9 | human | EPI_ISL_268509 | EPI_ISL_268509 | EPI_ISL_268509 | EPI_ISL_268509 | EPI_ISL_268509 | EPI_ISL_268509 | EPI_ISL_268509 | EPI_ISL_268509 |
| A/Changsha/1/2013 | H7N9 | Human | KF420296 | KF420298 | KF420300 | KF420302 | KF420304 | KF420306 | KF420308 | KF420310 |
| A/Shantou/1002/2014 | H7N9 | human | EPI_ISL_162619 | EPI_ISL_162619 | EPI_ISL_162619 | EPI_ISL_162619 | EPI_ISL_162619 | EPI_ISL_162619 | EPI_ISL_162619 | EPI_ISL_162619 |
| A/Shenzhen/SP126/2014 | H7N9 | Human | KP417422 | KP417424 | KP417425 | KP417423 | KP417426 | KP417421 | KP417420 | KP417419 |
| A/Hangzhou/1/2013 | H7N9 | human | EPI_ISL_138977 | EPI_ISL_138977 | EPI_ISL_138977 | EPI_ISL_138977 | EPI_ISL_138977 | EPI_ISL_138977 | EPI_ISL_138977 | EPI_ISL_138977 |
| A/Hong_Kong/VB16049808/2016 | H7N9 | human | EPI_ISL_215688 | EPI_ISL_215688 | EPI_ISL_215688 | EPI_ISL_215688 | EPI_ISL_215688 | EPI_ISL_215688 | EPI_ISL_215688 | EPI_ISL_215688 |
| A/Hangzhou/2/2013 | H7N9 | human | EPI_ISL_139498 | EPI_ISL_139498 | EPI_ISL_139498 | EPI_ISL_139498 | EPI_ISL_139498 | EPI_ISL_139498 | EPI_ISL_139498 | EPI_ISL_139498 |
| A/Nanjing/1/2013 | H7N9 | Human | KC896774 | KC896776 | KC896777 | KC896775 | KC896778 | KC896773 | KC896772 | KC896771 |
| A/GD-136/2014/H7N9/2014-03-29 | H7N9 | human | EPI_ISL_198713 | EPI_ISL_198713 | EPI_ISL_198713 | EPI_ISL_198713 | EPI_ISL_198713 | EPI_ISL_198713 | EPI_ISL_198713 | EPI_ISL_198713 |
| A/Wuxi/1/2013 | H7N9 | Human | KF034911 | KF034913 | KF034914 | KF034912 | KF034915 | KF034910 | KF034909 | KF034908 |
| A/Wuxi/2/2013 | H7N9 | Human | KF034919 | KF034921 | KF034922 | KF034920 | KF034923 | KF034918 | KF034917 | KF034916 |
| A/Anhui/04/2013 | H7N9 | human | EPI_ISL_157287 | EPI_ISL_157287 | EPI_ISL_157287 | EPI_ISL_157287 | EPI_ISL_157287 | EPI_ISL_157287 | EPI_ISL_157287 | EPI_ISL_157287 |
| A/Hangzhou/3/2013 | H7N9 | human | EPI_ISL_139499 | EPI_ISL_139499 | EPI_ISL_139499 | EPI_ISL_139499 | EPI_ISL_139499 | EPI_ISL_139499 | EPI_ISL_139499 | EPI_ISL_139499 |
| A/Shanghai/CN02/2013 | H7N9 | Human | KF918659 | KF918661 | KF918662 | KF918660 | KF918663 | KF918658 | KF918657 | KF918656 |
| A/Shenzhen/SP139/2014 | H7N9 | Human | KP417430 | KP417432 | KP417433 | KP417431 | KP417434 | KP417429 | KP417428 | KP417427 |
| A/Huzhou/1/2013 | H7N9 | human | EPI_ISL_144699 | EPI_ISL_144699 | EPI_ISL_144699 |  | EPI_ISL_144699 |  |  |  |
| A/Shanghai/JS01/2013 | H7N9 | Human | KF609511 | KF609513 | KF609514 | KF609512 | KF609515 | KF609510 | KF609509 | KF609508 |
| A/Zhejiang/DTID-ZJU01/2013 | H7N9 | Human | KJ633809 | KJ633810 | MF039637 | KC885957 | KC885960 | KJ633807 | KC885961 | KC885962 |
| A/Hong_Kong/5581/2014 | H7N9 | human | EPI_ISL_159477 | EPI_ISL_159477 | EPI_ISL_159477 | EPI_ISL_159477 | EPI_ISL_159477 | EPI_ISL_159477 | EPI_ISL_159477 | EPI_ISL_159477 |
| A/Shanghai/ION/2013 | H7N9 | Human | KP969031 | KP969032 | KP969037 | KP969033 | KP969038 | KP969036 | KP969035 | KP969034 |
| A/Nanjing/2/2013 | H7N9 | Human | KF007044 | KF007046 | KF007047 | KF007045 | KF007048 | KF007043 | KF007042 | KF007041 |
| A/GD-138/2014/H7N9/2014-04-05 | H7N9 | human | EPI_ISL_198714 | EPI_ISL_198714 | EPI_ISL_198714 | EPI_ISL_198714 | EPI_ISL_198714 | EPI_ISL_198714 | EPI_ISL_198714 | EPI_ISL_198714 |
| A/GD-139/2014/H7N9/2014-04-06 | H7N9 | human | EPI_ISL_198715 | EPI_ISL_198715 | EPI_ISL_198715 |  | EPI_ISL_198715 | EPI_ISL_198715 | EPI_ISL_198715 | EPI_ISL_198715 |
| A/Nanjing/4/2013 | H7N9 | Human | KF007052 | KF007054 | KF007055 | KF007053 | KF007056 | KF007051 | KF007050 | KF007049 |
| A/Wuxi/3/2013 | H7N9 | Human | KF007068 | KF007070 | KF007071 | KF007069 | KF007072 | KF007067 | KF007066 | KF007065 |
| A/Wuxi/4/2013 | H7N9 | Human | KF007076 | KF007078 | KF007079 | KF007077 | KF007080 | KF007075 | KF007074 | KF007073 |
| A/Zhenjiang/1/2013 | H7N9 | Human | KF007060 | KF007062 | KF007063 | KF007061 | KF007064 | KF007059 | KF007058 | KF007057 |
| A/Hong_Kong/5731/2014 | H7N9 | human | EPI_ISL_160290 | EPI_ISL_160290 | EPI_ISL_160290 | EPI_ISL_160290 | EPI_ISL_160290 | EPI_ISL_160290 | EPI_ISL_160290 | EPI_ISL_160290 |
| A/Nanjing/f0874/2013 | H7N9 | human | EPI_ISL_143658 | EPI_ISL_143658 | EPI_ISL_143658 | EPI_ISL_143658 | EPI_ISL_143658 | EPI_ISL_143658 | EPI_ISL_143658 | EPI_ISL_143658 |
| A/Suzhou/3/2013 | H7N9 | Human | KF007108 | KF007110 | KF007111 | KF007109 | KF007112 | KF007107 | KF007106 | KF007105 |
| A/Hangzhou/169/2013 | H7N9 | human | EPI_ISL_139903 | EPI_ISL_139903 | EPI_ISL_139903 | EPI_ISL_139903 | EPI_ISL_139903 | EPI_ISL_139903 | EPI_ISL_139903 | EPI_ISL_139903 |
| A/Hangzhou/173/2013 | H7N9 | human | EPI_ISL_139904 | EPI_ISL_139904 | EPI_ISL_139904 | EPI_ISL_139904 | EPI_ISL_139904 | EPI_ISL_139904 | EPI_ISL_139904 | EPI_ISL_139904 |
| A/Huzhou/3/2013 | H7N9 | human | EPI_ISL_144700 | EPI_ISL_144700 | EPI_ISL_144700 |  | EPI_ISL_144700 |  |  |  |
| A/Nanjing/6/2013 | H7N9 | Human | KF007116 | KF007118 | KF007119 | KF007117 | KF007120 | KF007115 | KF007114 | KF007113 |
| A/Shanghai/CN01/2013 | H7N9 | Human | KF609519 |  | KF609522 | KF609520 | KF609523 | KF609518 | KF609517 | KF609516 |
| A/Shantou/1003/2014 | H7N9 | human | EPI_ISL_162620 | EPI_ISL_162620 | EPI_ISL_162620 | EPI_ISL_162620 | EPI_ISL_162620 | EPI_ISL_162620 | EPI_ISL_162620 | EPI_ISL_162620 |
| A/Huzhou/5/2013 | H7N9 | human | EPI_ISL_144702 | EPI_ISL_144702 | EPI_ISL_144702 |  | EPI_ISL_144702 |  |  |  |
| A/Suzhou/5/2013 | H7N9 | Human | KF007140 | KF007142 | KF007143 | KF007141 | KF007144 | KF007139 | KF007138 | KF007137 |
| A/Huzhou/4/2013 | H7N9 | human | EPI_ISL_144701 | EPI_ISL_144701 | EPI_ISL_144701 |  | EPI_ISL_144701 |  |  |  |
| A/Huzhou/6/2013 | H7N9 | human | EPI_ISL_144740 |  | EPI_ISL_144740 |  | EPI_ISL_144740 |  |  |  |
| A/Nanjing/7/2013 | H7N9 | Human | KF007148 | KF007150 | KF007151 | KF007149 | KF007152 | KF007147 | KF007146 | KF007145 |
| A/Hong_Kong/8122430/2014 | H7N9 | human | EPI_ISL_159478 | EPI_ISL_159478 | EPI_ISL_159478 | EPI_ISL_159478 | EPI_ISL_159478 | EPI_ISL_159478 | EPI_ISL_159478 | EPI_ISL_159478 |
| A/Changsha/70/2017 | H7N9 | Human | MF370263 | MF370264 | MF370265 |  | MF370266 |  |  |  |
| A/Beijing/1/2013 | H7N9 | Human | KJ476633 | KJ476635 | KJ476636 | KJ476634 | KJ476637 | KJ476632 | KJ476631 | KJ476630 |
| A/Beijing/2/2013 | H7N9 | Human | KJ476641 | KJ476643 | KJ476644 | KJ476642 | KJ476645 | KJ476640 | KJ476639 | KJ476638 |
| A/Beijing/3/2013 | H7N9 | Human | KJ476649 | KJ476651 | KJ476652 | KJ476650 | KJ476653 | KJ476648 | KJ476647 | KJ476646 |
| A/Hangzhou/254/2013 | H7N9 | human | EPI_ISL_141775 | EPI_ISL_141775 | EPI_ISL_141775 | EPI_ISL_141775 | EPI_ISL_141775 | EPI_ISL_141775 | EPI_ISL_141775 | EPI_ISL_141775 |
| A/Shanghai/MH01/2013 | H7N9 | Human | KF609503 | KF609505 | KF609506 | KF609504 | KF609507 | KF609502 | KF609501 | KF609500 |
| A/Hangzhou/327/2013 | H7N9 | human | EPI_ISL_141776 | EPI_ISL_141776 | EPI_ISL_141776 | EPI_ISL_141776 | EPI_ISL_141776 | EPI_ISL_141776 | EPI_ISL_141776 | EPI_ISL_141776 |
| A/Shantou/1004/2014 | H7N9 | human | EPI_ISL_162621 | EPI_ISL_162621 | EPI_ISL_162621 | EPI_ISL_162621 | EPI_ISL_162621 | EPI_ISL_162621 | EPI_ISL_162621 | EPI_ISL_162621 |
| A/Hong_Kong/VB16064646/2016 | H7N9 | human | EPI_ISL_217947 | EPI_ISL_217947 | EPI_ISL_217947 | EPI_ISL_217947 | EPI_ISL_217947 | EPI_ISL_217947 | EPI_ISL_217947 | EPI_ISL_217947 |
| A/Huzhou/10/2013 | H7N9 | human | EPI_ISL_144711 |  |  |  |  |  |  |  |
| A/Jiangsu/1/2013 | H7N9 | Human | KF278745 | KF226115 | KF226114 | KF226116 | KF226117 | KF226118 | KF226119 | KF226120 |
| A/Jiangsu/2/2013 | H7N9 | Human | KF226105 | KF226107 | KF226106 | KF226108 | KF226109 | KF226110 | KF226111 | KF226112 |
| A/Zhejiang/DTID-ZJU02/2013 | H7N9 | Human | KF500922 | KF500923 |  |  |  |  |  | KF500921 |
| A/Zhejiang/DTID-ZJU02K/2013 | H7N9 | Human |  |  |  |  |  |  |  | KP126937 |
| A/Zhejiang/DTID-ZJU02N/2013 | H7N9 | Human |  |  |  |  |  |  |  | KP126938 |
| A/Zhejiang/DTID-ZJU02R/2013 | H7N9 | Human |  |  |  |  |  |  |  | KP126936 |
| A/Zhejiang/DTID-ZJU05/2013 | H7N9 | Human | KF500925 | KF500926 |  |  |  |  |  | KF500924 |
| A/Zhejiang/DTID-ZJU08/2013 | H7N9 | Human | KF500928 | KF500929 |  |  |  |  |  | KF500927 |
| A/Zhejiang/HZ1/2013 | H7N9 | Human | KF055470 | KF055467 | KF055471 | KF055465 | KF055469 | KF055466 | KF055468 | KF055472 |
| A/Zhejiang/KLED32/2013 | H7N9 | Human | KF193404 |  | KF193405 |  | KF193406 |  |  |  |
| A/Taiwan/S02076/2013 | H7N9 | Human | KF018045 | KF018047 | KF018048 | KF018046 | KF018049 | KF018044 | KF018043 | KF018042 |
| A/Taiwan/T02081/2013 | H7N9 | Human | KF018053 | KF018055 | KF018056 | KF018054 | KF018057 | KF018052 | KF018051 | KF018050 |
| A/Quzhou/2/2015 | H7N9 | Human | KR351273 | KR351274 | KR351275 | KR351276 | KR351277 | KR351278 | KR351279 | KR351280 |
| A/Fujian/1/2013 | H7N9 | Human | KC994453 | KC994454 | KC994455 | KF061026 | KC994456 | KF061025 | KF061024 | KF061023 |
| A/Nanchang/1/2013 | H7N9 | Human | KF261988 | KF469231 | KF277192 | KF277193 | KF277194 | KF277195 | KF469232 | KF277196 |
| A/Zhejiang/20/2013 | H7N9 | human | EPI_ISL_148727 | EPI_ISL_148727 | EPI_ISL_148727 | EPI_ISL_148727 | EPI_ISL_148727 | EPI_ISL_148727 | EPI_ISL_148727 | EPI_ISL_148727 |
| A/Taiwan/4-CGMH1/2014 | H7N9 | Human | KM244511 | KM244513 | KM244514 | KM244512 | KM244515 | KM244510 | KM244509 | KM244508 |
| A/Taiwan/4-CGMH2/2014 | H7N9 | Human | KM244519 | KM244521 | KM244522 | KM244520 | KM244523 | KM244518 | KM244517 | KM244516 |
| A/Xuzhou/1/2013 | H7N9 | Human | KF150616 | KF150618 | KF150619 | KF150617 | KF150620 | KF150615 | KF150614 | KF150613 |
| A/Quzhou/1/2015 | H7N9 | Human | KR351265 | KR351266 | KR351267 | KR351268 | KR351269 | KR351270 | KR351271 | KR351272 |
| A/Jiangsu/Wuxi04/2013 | H7N9 | human | EPI_ISL_220945 | EPI_ISL_220945 | EPI_ISL_220945 | EPI_ISL_220945 | EPI_ISL_220945 | EPI_ISL_220945 | EPI_ISL_220945 | EPI_ISL_220945 |
| A/Nanjing/M2/2013 | H7N9 | human | EPI_ISL_141601 | EPI_ISL_141601 | EPI_ISL_141601 | EPI_ISL_141601 | EPI_ISL_141601 | EPI_ISL_141601 | EPI_ISL_141601 | EPI_ISL_141601 |
| A/Fujian/02/2013 | H7N9 | human | EPI_ISL_157288 | EPI_ISL_157288 | EPI_ISL_157288 | EPI_ISL_157288 | EPI_ISL_157288 | EPI_ISL_157288 | EPI_ISL_157288 | EPI_ISL_157288 |
| A/Changsha/2/2013 | H7N9 | Human | KF420297 | KF420299 | KF420301 | KF420303 | KF420305 | KF420307 | KF420309 | KF420311 |
| A/Shanghai/patient1/2013 | H7N9 | human | EPI_ISL_141911 | EPI_ISL_141911 | EPI_ISL_141911 | EPI_ISL_141911 | EPI_ISL_141911 |  |  |  |
| A/Shanghai/Patient2/2013 | H7N9 | human | EPI_ISL_142041 | EPI_ISL_142041 | EPI_ISL_142041 | EPI_ISL_142041 | EPI_ISL_142041 |  |  |  |
| A/Shanghai/Patient3/2013_ | H7N9 | human | EPI_ISL_142045 | EPI_ISL_142045 | EPI_ISL_142045 |  | EPI_ISL_142045 |  |  |  |
| A/Shanghai/Patient4/2013 | H7N9 | human | EPI_ISL_142043 |  | EPI_ISL_142043 | EPI_ISL_142043 |  |  |  |  |
| A/Shanghai/Patient5/2013 | H7N9 | human |  | EPI_ISL_142073 |  |  |  |  |  |  |
| A/Shanghai/Patient6/2013 | H7N9 | human |  | EPI_ISL_142044 |  |  |  |  |  |  |
| A/GD-151/2014/H7N9/2014-05-06 | H7N9 | human | EPI_ISL_198716 | EPI_ISL_198716 | EPI_ISL_198716 | EPI_ISL_198716 | EPI_ISL_198716 | EPI_ISL_198716 | EPI_ISL_198716 | EPI_ISL_198716 |
| A/GD-153/2014/H7N9/2014-05-08 | H7N9 | human | EPI_ISL_198717 | EPI_ISL_198717 | EPI_ISL_198717 | EPI_ISL_198717 | EPI_ISL_198717 | EPI_ISL_198717 |  | EPI_ISL_198717 |
| A/Shanghai/5190T/2013 | H7N9 | Human | KF997831 | KF997832 | KF997834 | KF997833 | KF997835 | KF997836 | KF997837 | KF997838 |
| A/Huai'an/083/2014 | H7N9 | Human | KP864443 | KP864441 | KX215919 | KX215929 | KX215948 | KX215949 | KX215959 | KX215969 |
| A/GD-154/2014/H7N9/2014-05-28 | H7N9 | human | EPI_ISL_198718 | EPI_ISL_198718 | EPI_ISL_198718 | EPI_ISL_198718 | EPI_ISL_198718 | EPI_ISL_198718 | EPI_ISL_198718 | EPI_ISL_198718 |
| A/Guangdong/17SF003/2016 | H7N9 | human | EPI_ISL_266963 | EPI_ISL_266963 |  |  |  |  |  |  |
| A/Guangdong/17SF003/2016_NIBRG-375 | H7N9 | human | EPI_ISL_266964 | EPI_ISL_266964 |  |  |  |  |  |  |
| A/Huizhou/01/2013 | H7N9 | Human | KF667739 | KF667741 | KF667742 | KF667740 | KF667743 | KF667738 | KF667737 | KF667736 |
| A/Guangdong/1/2013 | H7N9 | Human | KF662943 | KF662949 | KF662950 | KF662948 | KF662947 | KF662946 | KF662945 | KF662944 |
| A/Zhejiang/22/2013 | H7N9 | human | EPI_ISL_148728 | EPI_ISL_148728 | EPI_ISL_148728 | EPI_ISL_148728 | EPI_ISL_148728 | EPI_ISL_148728 | EPI_ISL_148728 | EPI_ISL_148728 |
| A/Zhejiang/DTID-ZJU10/2013 | H7N9 | human | EPI_ISL_148744 | EPI_ISL_148744 | EPI_ISL_148744 | EPI_ISL_148744 | EPI_ISL_148744 | EPI_ISL_148744 | EPI_ISL_148744 | EPI_ISL_148744 |
| A/Guangdong/02/2013 | H7N9 | Human | KF922738 | KF894892 | KF944962 | KF944961 | KF944963 | KF944960 | KF944959 | KF944958 |
| A/Guangdong/6/2013 | H7N9 | Human | KX509956 | KX509958 | KX509959 | KX509957 | KX509960 | KX509955 | KX509954 | KX509953 |
| A/GD-155/2014/H7N9/2014-11-19 | H7N9 | human | EPI_ISL_198719 | EPI_ISL_198719 | EPI_ISL_198719 | EPI_ISL_198719 | EPI_ISL_198719 | EPI_ISL_198719 | EPI_ISL_198719 | EPI_ISL_198719 |
| A/Guangdong/DG-02/2013 | H7N9 | human | EPI_ISL_159070 | EPI_ISL_159070 | EPI_ISL_159070 | EPI_ISL_159070 | EPI_ISL_159070 | EPI_ISL_159070 | EPI_ISL_159070 | EPI_ISL_159070 |
| A/GD-156/2014/H7N9/2014-11-25 | H7N9 | human | EPI_ISL_198720 | EPI_ISL_198720 | EPI_ISL_198720 | EPI_ISL_198720 | EPI_ISL_198720 | EPI_ISL_198720 | EPI_ISL_198720 | EPI_ISL_198720 |
| A/Zhejiang/17/2014 | H7N9 | human | EPI_ISL_169131 | EPI_ISL_169131 | EPI_ISL_169131 | EPI_ISL_169131 | EPI_ISL_169131 | EPI_ISL_169131 | EPI_ISL_169131 | EPI_ISL_169131 |
| A/Zhejiang/33/2014 | H7N9 | human | EPI_ISL_192299 | EPI_ISL_192299 | EPI_ISL_192299 | EPI_ISL_192299 | EPI_ISL_192299 | EPI_ISL_192299 | EPI_ISL_192299 | EPI_ISL_192299 |
| A/Hong_Kong/470129/2013 | H7N9 | Human | KF952508 | KF952507 | KF952506 | KF952509 | KF952510 | KF952513 | KF952512 | KF952511 |
| A/Hong_Kong/5942/2013 | H7N9 | human | EPI_ISL_151417 | EPI_ISL_151417 | EPI_ISL_151417 | EPI_ISL_151417 | EPI_ISL_151417 | EPI_ISL_151417 | EPI_ISL_151417 | EPI_ISL_151417 |
| A/Zhejiang/7/2016 | H7N9 | human | EPI_ISL_242854 | EPI_ISL_242854 | EPI_ISL_242854 | EPI_ISL_242854 | EPI_ISL_242854 | EPI_ISL_242854 | EPI_ISL_242854 | EPI_ISL_242854 |
| A/Guangdong/03/2013 | H7N9 | Human | KJ023067 | KJ023069 | KJ023070 | KJ023068 | KJ023071 |  |  |  |
| A/Guangdong/04/2013 | H7N9 | Human | KJ023074 | KJ023076 | KJ023077 | KJ023075 | KJ023078 | KJ023073 | KJ023072 |  |
| A/Guangdong/05/2013 | H7N9 | Human | KJ023082 | KJ023084 | KJ023085 | KJ023083 | KJ023086 | KJ023081 | KJ023080 | KJ023079 |
| A/Hong_Kong/VB16184091/2016 | H7N9 | human | EPI_ISL_239994 | EPI_ISL_239994 | EPI_ISL_239994 | EPI_ISL_239994 | EPI_ISL_239994 | EPI_ISL_239994 | EPI_ISL_239994 | EPI_ISL_239994 |
| A/Guangdong/7/2013 | H7N9 | Human | KX523685 | KX523687 | KX523688 | KX523686 | KX523689 | KX523684 | KX523683 | KX523682 |
| A/Guangdong/DG-03/2013 | H7N9 | human | EPI_ISL_159071 | EPI_ISL_159071 | EPI_ISL_159071 | EPI_ISL_159071 | EPI_ISL_159071 | EPI_ISL_159071 | EPI_ISL_159071 | EPI_ISL_159071 |
| A/Guangdong/YJ-04/2013 | H7N9 | human | EPI_ISL_159072 | EPI_ISL_159072 | EPI_ISL_159072 | EPI_ISL_159072 | EPI_ISL_159072 | EPI_ISL_159072 | EPI_ISL_159072 | EPI_ISL_159072 |
| A/Guangdong/YJ-05/2013 | H7N9 | human | EPI_ISL_159073 | EPI_ISL_159073 | EPI_ISL_159073 | EPI_ISL_159073 | EPI_ISL_159073 | EPI_ISL_159073 | EPI_ISL_159073 | EPI_ISL_159073 |
| A/Zhejiang/15/2016 | H7N9 | human | EPI_ISL_242858 | EPI_ISL_242858 | EPI_ISL_242858 | EPI_ISL_242858 | EPI_ISL_242858 | EPI_ISL_242858 | EPI_ISL_242858 | EPI_ISL_242858 |
| A/Zhejiang/16/2016 | H7N9 | human | EPI_ISL_242857 | EPI_ISL_242857 | EPI_ISL_242857 | EPI_ISL_242857 | EPI_ISL_242857 | EPI_ISL_242857 | EPI_ISL_242857 | EPI_ISL_242857 |
| A/Zhejiang/14/2016 | H7N9 | human | EPI_ISL_242859 | EPI_ISL_242859 | EPI_ISL_242859 | EPI_ISL_242859 | EPI_ISL_242859 | EPI_ISL_242859 | EPI_ISL_242859 | EPI_ISL_242859 |
| A/Zhejiang/13/2016 | H7N9 | human | EPI_ISL_242860 | EPI_ISL_242860 | EPI_ISL_242860 | EPI_ISL_242860 | EPI_ISL_242860 | EPI_ISL_242860 | EPI_ISL_242860 | EPI_ISL_242860 |
| A/Hong_Kong/8194273/2014 | H7N9 | human | EPI_ISL_169442 | EPI_ISL_169442 | EPI_ISL_169442 | EPI_ISL_169442 | EPI_ISL_169442 | EPI_ISL_169442 | EPI_ISL_169442 | EPI_ISL_169442 |
| A/Zhejiang/34/2014 | H7N9 | human | EPI_ISL_192295 | EPI_ISL_192295 | EPI_ISL_192295 | EPI_ISL_192295 | EPI_ISL_192295 | EPI_ISL_192295 | EPI_ISL_192295 | EPI_ISL_192295 |
| A/Zhejiang/12/2016 | H7N9 | human | EPI_ISL_242861 | EPI_ISL_242861 | EPI_ISL_242861 | EPI_ISL_242861 | EPI_ISL_242861 | EPI_ISL_242861 | EPI_ISL_242861 | EPI_ISL_242861 |
| A/Zhejiang/35/2014 | H7N9 | human | EPI_ISL_192294 | EPI_ISL_192294 | EPI_ISL_192294 | EPI_ISL_192294 | EPI_ISL_192294 | EPI_ISL_192294 | EPI_ISL_192294 | EPI_ISL_192294 |
| A/Taiwan/3/2013 | H7N9 | human | EPI_ISL_166324 | EPI_ISL_166324 | EPI_ISL_166324 | EPI_ISL_166324 | EPI_ISL_166324 | EPI_ISL_166324 | EPI_ISL_166324 | EPI_ISL_166324 |
| A/Zhejiang/36/2014 | H7N9 | human | EPI_ISL_192292 | EPI_ISL_192292 | EPI_ISL_192292 | EPI_ISL_192292 | EPI_ISL_192292 | EPI_ISL_192292 | EPI_ISL_192292 | EPI_ISL_192292 |
| A/Hong_Kong/VB16189623/2016 | H7N9 | human | EPI_ISL_240520 | EPI_ISL_240520 | EPI_ISL_240520 | EPI_ISL_240520 | EPI_ISL_240520 | EPI_ISL_240520 | EPI_ISL_240520 | EPI_ISL_240520 |
| A/Anhui/13436/2016 | H7N9 | human | EPI_ISL_258022 | EPI_ISL_258022 | EPI_ISL_258022 | EPI_ISL_258022 | EPI_ISL_258022 | EPI_ISL_258022 | EPI_ISL_258022 | EPI_ISL_258022 |
| A/green-winged_teal/Wisconsin/228/1976 | H9N2 | Avian |  |  | CY180140 | CY180142 | CY180143 | CY180144 | CY180145 | CY180146 |
| A/duck/Hong_Kong/702/1979 | H9N2 | Avian |  |  | CY031262 | CY031260 | CY031263 | CY031258 | CY031257 | CY031256 |
| A/duck/Hong_Kong/702/1979-chicken_adapted | H9N2 | Avian |  |  | CY031270 | CY031268 | CY031271 | CY031266 | CY031265 | CY031264 |
| A/duck/Hong_Kong/702/1979-quail_adapted | H9N2 | Avian |  |  | CY031278 | CY031284 | CY031279 | CY031274 | CY031281 | CY031272 |
| A/turkey/TX/4-1-81/1981 | H9N2 | Avian |  |  | EU982317 | EU982319 | EU982320 | EU982321 | EU982322 | EU982323 |
| A/turkey/TX/10-49-89/1989 | H9N2 | Avian |  |  | EU982309 | EU982311 | EU982312 | EU982313 | EU982314 | EU982315 |
| A/chicken/Korea/MS96-CE6/1996 | H9N2 | Avian |  |  | GU053187 | GU053189 | GU053190 | GU053191 | GU053192 | GU053193 |
| A/chicken/Shandong/6/96 | H9N2 | Avian |  |  | AF508700 | AF508613 | AF508722 | DQ064511 | DQ064538 | AF508657 |
| A/chicken/Shandong/7/96 | H9N2 | Avian |  |  | DQ064404 | DQ064458 | DQ064485 | DQ064512 | DQ064539 | DQ064566 |
| A/chicken/Guangdong/5/97 | H9N2 | Avian |  |  | DQ064387 | DQ064441 | DQ064468 | DQ064495 | DQ064522 | DQ064549 |
| A/chicken/Guangdong/6/97 | H9N2 | Avian |  |  | DQ064389 | DQ064443 | DQ064470 | DQ064497 | DQ064524 | DQ064551 |
| A/chicken/Hong_Kong/G9/1997 | H9N2 | Avian |  |  | KF188369 | KF188367 | KF188370 | KF188365 | KF188364 | KF188363 |
| A/chicken/Osaka/aq48/97 | H9N2 | Avian |  |  | AB256669 | AB256667 | AB256670 | AB256665 | AB256664 | AB256663 |
| A/chicken/Shenzhen/9/97 | H9N2 | Avian |  |  | DQ064381 | DQ064435 | DQ064462 | DQ064489 | DQ064516 | DQ064569 |
| A/duck/Hong_Kong/Y439/1997 | H9N2 | Avian |  |  | KF188268 | KF188266 | KF188269 | KF188264 | KF188263 | KF188262 |
| A/duck/Nanjing/1/97 | H9N2 | Avian |  |  | DQ064400 | DQ064454 | DQ064481 | AF508682 | AF508638 | DQ064562 |
| A/duck/Nanjing/2/97 | H9N2 | Avian |  |  | DQ064401 | DQ064455 | DQ064482 | DQ064509 | DQ064536 | DQ064563 |
| A/parakeet/Chiba/1/97 | H9N2 | Avian |  |  | AB049165 | AB049161 | AB049167 | AB049157 | AB049155 | AB049153 |
| A/chicken/Beijing/8/98 | H9N2 | Avian |  |  | AF508693 | DQ064461 | DQ064488 | AF508671 | DQ064542 | AF508649 |
| A/chicken/Henan/5/98 | H9N2 | Avian |  |  | DQ064397 | DQ064451 | DQ064478 | DQ064505 | DQ064532 | DQ064559 |
| A/Chicken/Shanghai/F/98 | H9N2 | Avian |  |  | AY253755 | AY253753 | AY253756 | AY253752 | AY253751 | AY253750 |
| A/chicken/Shijiazhuang/2/98 | H9N2 | Avian |  |  | DQ064406 | DQ064460 | DQ064487 | DQ064514 | DQ064541 | DQ064568 |
| A/duck/Hokkaido/49/98 | H9N2 | Avian |  |  | AB473941 | AB473940 | AB473942 | AB473939 | AB473938 | AB473937 |
| A/parakeet/Narita/92A/98 | H9N2 | Avian |  |  | AB049166 | AB049162 | AB049168 | AB049158 | AB049156 | AB049154 |
| A/chicken/Gansu/2/99 | H9N2 | Avian |  |  | EF070737 | EF070735 | EF070736 | EF070738 | EF070739 | EF070740 |
| A/chicken/Guangxi/10/99 | H9N2 | Avian |  |  | DQ064390 | DQ064444 | DQ064471 | DQ064498 | DQ064525 | DQ064552 |
| A/chicken/Guangxi/4/1999 | H9N2 | Avian |  |  | EU081865 | EU081867 | EU081868 | EU081869 | EU081870 | EU081871 |
| A/chicken/Guangxi/9/99 | H9N2 | Avian |  |  | DQ064391 | DQ064445 | DQ064472 | DQ064499 | DQ064526 | DQ064553 |
| A/chicken/Guangxi/KMIII/1999 | H9N2 | Avian |  |  | HQ117889 | HQ117887 | HQ117890 | HQ117885 | HQ117884 | HQ117883 |
| A/chicken/Ningxia/4/99 | H9N2 | Avian |  |  | DQ064402 | DQ064456 | DQ064483 | DQ064510 | DQ064537 | DQ064564 |
| A/duck/Hokkaido/9/99 | H9N2 | Avian |  |  | AB262466 | AB262464 | AB262467 | AB262462 | AB262461 | AB262460 |
| A/guineafowl/Hong_Kong/WF10_CIP046_RGAO031/1999 | H9N2 | Avian |  |  | KX859313 | KX859351 | KX859329 | KX859342 | KX859441 | KX859312 |
| A/chicken/Fujian/25/00 | H9N2 | Avian |  |  | DQ064382 | DQ064436 | DQ064463 | DQ064490 | DQ064517 | DQ064544 |
| A/chicken/Guangdong/10/00 | H9N2 | Avian |  |  | DQ064383 | AF508606 | AF508715 | DQ064491 | DQ064518 | AF508650 |
| A/chicken/Guangdong/4/00 | H9N2 | Avian |  |  | DQ064385 | DQ064439 | DQ064466 | DQ064493 | DQ064520 | DQ064548 |
| A/chicken/Guangxi/6/2000 | H9N2 | Avian |  |  | EU086238 | EU086240 | EU086241 | EU086242 | EU086243 | EU086244 |
| A/chicken/Hebei/31/00 | H9N2 | Avian |  |  | DQ064392 | DQ064446 | DQ064473 | DQ064500 | DQ064527 | DQ064554 |
| A/chicken/Heilongjiang/35/00 | H9N2 | Avian |  |  | DQ064393 | DQ064447 | DQ064474 | DQ064501 | DQ064528 | DQ064555 |
| A/chicken/Henan/26/00 | H9N2 | Avian |  |  | DQ064395 | DQ064449 | DQ064476 | DQ064503 | DQ064530 | DQ064557 |
| A/chicken/Israel/90658/2000 | H9N2 | Avian |  |  | EF492272 | EF492330 | DQ683047 | EF492370 | EF492399 | EF492428 |
| A/chicken/Jiangsu/1/00 | H9N2 | Avian |  |  | DQ064399 | DQ064453 | DQ064480 | DQ064507 | DQ064534 | DQ064561 |
| A/Korea/KBNP-0028/2000 | H9N2 | Avian |  |  | EF620903 | EF620901 | EF620904 | EF620899 | EF620898 | EF620897 |
| A/chicken/Dubai/339/2001 | H9N2 | Avian |  |  | KF188355 | KF188353 | KF188356 | KF188351 | KF188350 | KF188349 |
| A/chicken/Guangdong/47/01 | H9N2 | Avian |  |  | DQ064386 | DQ064440 | DQ064467 | DQ064494 | DQ064521 | DQ064547 |
| A/chicken/Guangdong/56/01 | H9N2 | Avian |  |  | DQ064388 | DQ064442 | DQ064469 | DQ064496 | DQ064523 | DQ064550 |
| A/chicken/Heilongjiang/48/01 | H9N2 | Avian |  |  | DQ064394 | DQ064448 | DQ064475 | DQ064502 | DQ064529 | DQ064556 |
| A/chicken/Israel/786/2001 | H9N2 | Avian |  |  | EF492269 | EF492312 | DQ683032 | EF492352 | EF492381 | EF492410 |
| A/chicken/Jilin/53/01 | H9N2 | Avian |  |  | DQ064398 | DQ064452 | DQ064479 | DQ064506 | DQ064533 | DQ064560 |
| A/chicken/Kobe/aq26/2001 | H9N2 | Avian |  |  | AB256685 | AB256683 | AB256686 | AB256681 | AB256680 | AB256679 |
| A/chicken/Korea/01310/2001 | H9N2 | Avian |  |  | JX094857 | JX094856 | JX094858 | JX094855 | JX094854 | JX094853 |
| A/chicken/Osaka/aq19/2001 | H9N2 | Avian |  |  | AB256741 | AB256739 | AB256742 | AB256737 | AB256736 | AB256735 |
| A/chicken/Osaka/aq58/2001 | H9N2 | Avian |  |  | AB256693 | AB256691 | AB256694 | AB256689 | AB256688 | AB256687 |
| A/chicken/Osaka/aq69/2001 | H9N2 | Avian |  |  | AB256701 | AB256699 | AB256702 | AB256697 | AB256696 | AB256695 |
| A/chicken/Shanghai/10/01 | H9N2 | Avian |  |  | DQ064405 | DQ064459 | DQ064486 | DQ064513 | DQ064540 | DQ064567 |
| A/chicken/Yokohama/aq120/2001 | H9N2 | Avian |  |  | AB256733 | AB256731 | AB256734 | AB256729 | AB256728 | AB256727 |
| A/chicken/Yokohama/aq135/2001 | H9N2 | Avian |  |  | AB256725 | AB256723 | AB256726 | AB256721 | AB256720 | AB256719 |
| A/chicken/Yokohama/aq144/2001 | H9N2 | Avian |  |  | AB256749 | AB256747 | AB256750 | AB256745 | AB256744 | AB256743 |
| A/chicken/Yokohama/aq55/2001 | H9N2 | Avian |  |  | AB256677 | AB256675 | AB256678 | AB256673 | AB256672 | AB256671 |
| A/duck/Malaysia/2001 | H9N2 | Avian |  |  | CY073803 | CY073801 | CY073804 | CY073799 | CY073798 | CY073797 |
| A/turkey/Israel/810/2001 | H9N2 | Avian |  |  | EF492270 | EF492314 | DQ683033 | EF492354 | EF492383 | EF492412 |
| A/chicken/Emirates/R66/2002 | H9N2 | Avian |  |  | CY076726 | CY076724 | CY076727 | CY076722 | CY076721 | CY076720 |
| A/chicken/Guangdong/21/02 | H9N2 | Avian |  |  | DQ064384 | DQ064438 | DQ064465 | DQ064492 | DQ064519 | DQ064546 |
| A/chicken/Henan/43/02 | H9N2 | Avian |  |  | DQ064396 | DQ064450 | DQ064477 | DQ064504 | DQ064531 | DQ064558 |
| A/chicken/Jiangsu/7/2002 | H9N2 | Avian |  |  | FJ384754 | FJ384744 | FJ384755 | FJ384758 | FJ384749 | FJ384748 |
| A/chicken/Yokohama/aq134/2002 | H9N2 | Avian |  |  | AB256717 | AB256715 | AB256718 | AB256713 | AB256712 | AB256711 |
| A/chicken/Yokohama/aq45/2002 | H9N2 | Avian |  |  | AB256709 | AB256707 | AB256710 | AB256705 | AB256704 | AB256703 |
| A/turkey/Israel/1013/2002 | H9N2 | Avian |  |  | EF492257 | EF492319 | DQ683036 | EF492359 | EF492388 | EF492417 |
| A/turkey/Israel/619/2002 | H9N2 | Avian |  |  | EF492268 | EF492310 | DQ683031 | EF492350 | EF492379 | EF492408 |
| A/chicken/Hong_Kong/TP38/2003 | H9N2 | Avian |  |  | KF188307 | KF188305 | KF188308 | KF188303 | KF188302 | KF188301 |
| A/chicken/Israel/1376/2003 | H9N2 | Avian |  |  | EF492260 | EF492322 | DQ683039 | EF492362 | EF492391 | EF492420 |
| A/chicken/Israel/1475/2003 | H9N2 | Avian |  |  | EF492262 | EF492324 | DQ683041 | EF492364 | EF492393 | EF492422 |
| A/guineafowl/HongKong/NT101/2003 | H9N2 | Avian |  |  | KF188385 | KF188383 | KF188386 | KF188381 | KF188380 | KF188379 |
| A/turkey/Israel/1209/2003 | H9N2 | Avian |  |  | EF492258 | EF492320 | DQ683037 | EF492360 | EF492389 | EF492418 |
| A/chicken/Israel/1808/2004 | H9N2 | Avian |  |  | EF492265 | EF492327 | DQ683044 | EF492367 | EF492396 | EF492425 |
| A/chicken/Israel/1953/2004 | H9N2 | Avian |  |  | EF492266 | EF492328 | DQ683045 | EF492368 | EF492397 | EF492426 |
| A/chicken/Israel/1966/2004 | H9N2 | Avian |  |  | EF492267 | EF492329 | DQ683046 | EF492369 | EF492398 | EF492427 |
| A/chicken/Korea/S21/2004 | H9N2 | Avian |  |  | EU662948 | EU662953 | EU662954 | EU662956 | EU662958 | EU662960 |
| A/duck/Hubei/W1/2004 | H9N2 | Avian |  |  | DQ465403 | DQ465401 | DQ465404 | DQ465399 | DQ465398 | DQ465397 |
| A/avian/Israel/584/2005 | H9N2 | Avian |  |  | EF492251 | EF492309 | EF492337 | EF492349 | EF492378 | EF492407 |
| A/bird/Guangxi/62/2005 | H9N2 | Avian |  |  | EU086247 | EU086252 | EU086255 | EU086257 | EU086279 | EU086281 |
| A/bird/Guangxi/82/2005 | H9N2 | Avian |  |  | EU086268 | EU086273 | EU086276 | EU086278 | EU086280 | EU086282 |
| A/bird/Guangxi/83/2005 | H9N2 | Avian |  |  | EU086269 | EU086274 | EU086277 | EU086293 | EU086296 | EU086300 |
| A/chicken/Guangxi/37/2005 | H9N2 | Avian |  |  | EU086227 | EU086229 | EU086230 | EU086231 | EU086232 | EU086233 |
| A/chicken/Guangxi/55/2005 | H9N2 | Avian |  |  | EU086248 | EU086253 | EU086256 | EU086258 | EU086261 | EU086263 |
| A/chicken/Israel/282/2005 | H9N2 | Avian |  |  | EF492248 | EF492306 | EF492334 | EF492346 | EF492375 | EF492404 |
| A/chicken/Israel/554/2005 | H9N2 | Avian |  |  | EF492250 | EF492308 | EF492336 | EF492348 | EF492377 | EF492406 |
| A/chicken/Israel/793/2005 | H9N2 | Avian |  |  | EF492253 | EF492313 | EF492339 | EF492353 | EF492382 | EF492411 |
| A/chicken/Israel/853/2005 | H9N2 | Avian |  |  | EF492254 | EF492315 | EF492340 | EF492355 | EF492384 | EF492413 |
| A/chicken/Israel/909/2005 | H9N2 | Avian |  |  | EF492256 | EF492317 | EF492331 | EF492357 | EF492386 | EF492415 |
| A/duck/Guangxi/51/2005 | H9N2 | Avian |  |  | EU086235 | EU086251 | EU086254 | EU086259 | EU086260 | EU086262 |
| A/Eurasian_wigeon/Netherlands/3/2005 | H9N2 | Avian |  |  | CY043857 | CY043859 | CY043860 | CY043861 | CY043862 | CY043863 |
| A/turkey/Israel/425/2005 | H9N2 | Avian |  |  | EF492249 | EF492307 | EF492335 | EF492347 | EF492376 | EF492405 |
| A/turkey/Israel/747/2005 | H9N2 | Avian |  |  | EF492252 | EF492311 | EF492338 | EF492351 | EF492380 | EF492409 |
| A/turkey/Israel/884/2005 | H9N2 | Avian |  |  | EF492255 | EF492316 | EF492341 | EF492356 | EF492385 | EF492414 |
| A/turkey/Israel/89/2005 | H9N2 | Avian |  |  | EF492246 | EF492304 | DQ683026 | EF492344 | EF492373 | EF492402 |
| A/avian/Saudi_Arabia/910134/2006 | H9N2 | Avian |  |  | GU050280 | GU050282 | GU050283 | GU050284 | GU050285 | GU050286 |
| A/bird/Guangxi/A1/2006 | H9N2 | Avian |  |  | EU086286 | EU086290 | EU086291 | EU086295 | EU086298 | EU086301 |
| A/bird/Guangxi/H1/2006 | H9N2 | Avian |  |  | EU086305 | EU086308 | EU086311 | EU086312 | EU086315 | EU086317 |
| A/chicken/Hebei/L1/2006 | H9N2 | Avian |  |  | EU532036 | EU532044 | EU532055 | EU532061 | EU882861 | EU914201 |
| A/chicken/Israel/178/2006 | H9N2 | Avian |  |  | EF492247 | EF492305 | EF492333 | EF492345 | EF492374 | EF492403 |
| A/pink-footed_goose/Netherlands/1/2006 | H9N2 | Avian |  |  | CY041267 | CY041269 | CY041270 | CY041271 | CY041272 | CY041273 |
| A/quail/Guangxi/B1/2006 | H9N2 | Avian |  |  | EU086285 | EU086289 | EU086292 | EU086294 | EU086297 | EU086299 |
| A/Bewick's_swan/Netherlands/5/2007 | H9N2 | Avian |  |  | CY041275 | CY041277 | CY041278 | CY041279 | CY041280 | CY041281 |
| A/chicken/Hong_Kong/NT286/2007 | H9N2 | Avian |  |  | KF259327 | KF259846 | KF260090 | KF260334 | KF260578 | KF260822 |
| A/chicken/Hong_Kong/YU797/2007 | H9N2 | Avian |  |  | KF259332 | KF259851 | KF260095 | KF260339 | KF260583 | KF260827 |
| A/chicken/Hubei/C1/2007 | H9N2 | Avian |  |  | EU365374 | EU365372 | EU365375 | EU365370 | EU365369 | EU365368 |
| A/chicken/Zhejiang/HJ/2007 | H9N2 | Avian |  |  | FJ581432 | FJ581428 | FJ581433 | FJ581430 | FJ581435 | FJ581429 |
| A/duck/Chiba/1/2007 | H9N2 | Avian |  |  | AB874678 | AB874676 | AB874679 | AB874674 | AB874673 | AB874672 |
| A/pheasant/Hong_Kong/WF288W/2007 | H9N2 | Avian |  |  | KF259336 | KF259855 | KF260099 | KF260343 | KF260587 | KF260831 |
| A/pigeon/Hong_Kong/WF255/2007 | H9N2 | Avian |  |  | KF259333 | KF259852 | KF260096 | KF260340 | KF260584 | KF260828 |
| A/rosy-billed_pochard/Argentina/CIP051-559/2007 | H9N2 | Avian |  |  | CY111593 | CY111591 | CY111594 | CY111589 | CY111588 | CY111587 |
| A/silkie_chicken/Hong_Kong/NT382/2007 | H9N2 | Avian |  |  | KF259338 | KF259857 | KF260101 | KF260345 | KF260589 | KF260833 |
| A/chicken/Hong_Kong/NT155/2008 | H9N2 | Avian |  |  | KF259341 | KF259860 | KF260104 | KF260348 | KF260592 | KF260836 |
| A/chicken/Hong_Kong/YSC82/2008 | H9N2 | Avian |  |  | KF259345 | KF259864 | KF260108 | KF260352 | KF260596 | KF260840 |
| A/chicken/Hong_Kong/YU341/2008 | H9N2 | Avian |  |  | KF259346 | KF259865 | KF260109 | KF260353 | KF260597 | KF260841 |
| A/quail/Bangladesh/907/2009 | H9N2 | Avian |  |  | KF188252 | KF188250 | KF188253 | KF188248 | KF188247 | KF188246 |
| A/chicken/Hong_Kong/CRA45/2010 | H9N2 | Avian |  |  | KF259370 | KF259889 | KF260133 | KF260377 | KF260621 | KF260865 |
| A/chicken/Hong_Kong/CRA45W/2010 | H9N2 | Avian |  |  | KF259371 | KF259890 | KF260134 | KF260378 | KF260622 | KF260866 |
| A/chicken/Hong_Kong/TSTB25W/2010 | H9N2 | Avian |  |  | KF259368 | KF259887 | KF260131 | KF260375 | KF260619 | KF260863 |
| A/chicken/Hong_Kong/CSW445W/2011 | H9N2 | Avian |  |  | KF259384 | KF259903 | KF260147 | KF260391 | KF260635 | KF260879 |
| A/chicken/Hong_Kong/CSW530/2011 | H9N2 | Avian |  |  | KF259388 | KF259907 | KF260151 | KF260395 | KF260639 | KF260883 |
| A/chicken/Hong_Kong/CSW533/2011 | H9N2 | Avian |  |  | KF259389 | KF259908 | KF260152 | KF260396 | KF260640 | KF260884 |
| A/chicken/Hong_Kong/JV75/2011 | H9N2 | Avian |  |  | KF259376 | KF259895 | KF260139 | KF260383 | KF260627 | KF260871 |
| A/chicken/Hong_Kong/NT10/2011 | H9N2 | Avian |  |  | KF259372 | KF259891 | KF260135 | KF260379 | KF260623 | KF260867 |
| A/chicken/Hong_Kong/NT148W/2011 | H9N2 | Avian |  |  | KF259377 | KF259896 | KF260140 | KF260384 | KF260628 | KF260872 |
| A/chicken/Hong_Kong/NT149W/2011 | H9N2 | Avian |  |  | KF259378 | KF259897 | KF260141 | KF260385 | KF260629 | KF260873 |
| A/chicken/Hong_Kong/NT180W/2011 | H9N2 | Avian |  |  | KF259380 | KF259899 | KF260143 | KF260387 | KF260631 | KF260875 |
| A/chicken/Hong_Kong/NT305W/2011 | H9N2 | Avian |  |  | KF259383 | KF259902 | KF260146 | KF260390 | KF260634 | KF260878 |
| A/chicken/Hong_Kong/TC18/2011 | H9N2 | Avian |  |  | KF259373 | KF259892 | KF260136 | KF260380 | KF260624 | KF260868 |
| A/chicken/Hong_Kong/TSTB32W/2011 | H9N2 | Avian |  |  | KF259381 | KF259900 | KF260144 | KF260388 | KF260632 | KF260876 |
| A/chicken/Hong_Kong/YO151W/2011 | H9N2 | Avian |  |  | KF259382 | KF259901 | KF260145 | KF260389 | KF260633 | KF260877 |
| A/chicken/Hong_Kong/YO173W/2011 | H9N2 | Avian |  |  | KF259387 | KF259906 | KF260150 | KF260394 | KF260638 | KF260882 |
| A/chicken/Hong_Kong/YO28/2011 | H9N2 | Avian |  |  | KF259374 | KF259893 | KF260137 | KF260381 | KF260625 | KF260869 |
| A/chicken/Hong_Kong/YU158/2011 | H9N2 | Avian |  |  | KF259375 | KF259894 | KF260138 | KF260382 | KF260626 | KF260870 |
| A/chicken/Hong_Kong/YU250W/2011 | H9N2 | Avian |  |  | KF259379 | KF259898 | KF260142 | KF260386 | KF260630 | KF260874 |
| A/chicken/Hong_Kong/YU551W/2011 | H9N2 | Avian |  |  | KF259385 | KF259904 | KF260148 | KF260392 | KF260636 | KF260880 |
| A/ck/Guizhou/6/2011 | H9N2 | Avian |  |  | KP185395 | KP185725 | KP185807 | KP185643 | KP185889 | KP185477 |
| A/silkie_chicken/Hong_Kong/YU595W/2011 | H9N2 | Avian |  |  | KF259386 | KF259905 | KF260149 | KF260393 | KF260637 | KF260881 |
| A/chicken/Hong_Kong/FY105W/2012 | H9N2 | Avian |  |  | KF259402 | KF259921 | KF260165 | KF260409 | KF260653 | KF260897 |
| A/chicken/Hong_Kong/FY6W/2012 | H9N2 | Avian |  |  | KF259391 | KF259910 | KF260154 | KF260398 | KF260642 | KF260886 |
| A/chicken/Hong_Kong/HH117/2012 | H9N2 | Avian |  |  | KF259399 | KF259918 | KF260162 | KF260406 | KF260650 | KF260894 |
| A/chicken/Hong_Kong/JV17/2012 | H9N2 | Avian |  |  | KF259396 | KF259915 | KF260159 | KF260403 | KF260647 | KF260891 |
| A/chicken/Hong_Kong/JV231W/2012 | H9N2 | Avian |  |  | KF259405 | KF259924 | KF260168 | KF260412 | KF260656 | KF260900 |
| A/chicken/Hong_Kong/NT266/2012 | H9N2 | Avian |  |  | KF259404 | KF259923 | KF260167 | KF260411 | KF260655 | KF260899 |
| A/chicken/Hong_Kong/TC8/2012 | H9N2 | Avian |  |  | KF259390 | KF259909 | KF260153 | KF260397 | KF260641 | KF260885 |
| A/chicken/Hong_Kong/WF1W/2012 | H9N2 | Avian |  |  | KF259394 | KF259913 | KF260157 | KF260401 | KF260645 | KF260889 |
| A/chicken/Hong_Kong/WF3/2012 | H9N2 | Avian |  |  | KF259393 | KF259912 | KF260156 | KF260400 | KF260644 | KF260888 |
| A/chicken/Hong_Kong/YO117W/2012 | H9N2 | Avian |  |  | KF259403 | KF259922 | KF260166 | KF260410 | KF260654 | KF260898 |
| A/chicken/Hong_Kong/YO12W/2012 | H9N2 | Avian |  |  | KF259395 | KF259914 | KF260158 | KF260402 | KF260646 | KF260890 |
| A/chicken/Hong_Kong/YO6/2012 | H9N2 | Avian |  |  | KF259392 | KF259911 | KF260155 | KF260399 | KF260643 | KF260887 |
| A/chicken/Hong_Kong/YU148/2012 | H9N2 | Avian |  |  | KF259397 | KF259916 | KF260160 | KF260404 | KF260648 | KF260892 |
| A/chicken/Hong_Kong/YU150W/2012 | H9N2 | Avian |  |  | KF259398 | KF259917 | KF260161 | KF260405 | KF260649 | KF260893 |
| A/chicken/Hong_Kong/YU262W/2012 | H9N2 | Avian |  |  | KF259400 | KF259919 | KF260163 | KF260407 | KF260651 | KF260895 |
| A/chicken/Hong_Kong/YU344/2012 | H9N2 | Avian |  |  | KF259401 | KF259920 | KF260164 | KF260408 | KF260652 | KF260896 |
| A/chicken/Fujian/SIC4/2013 | H9N2 | Avian |  |  | KX598689 | KX598647 | KX598731 | KX598605 | KX598563 | KX598521 |
| A/chicken/Fujian/SIC5/2013 | H9N2 | Avian |  |  | KX598696 | KX598654 | KX598738 | KX598612 | KX598570 | KX598528 |
| A/chicken/Guangxi/SIC6/2013 | H9N2 | Avian |  |  | KX598687 | KX598645 | KX598729 | KX598603 | KX598561 | KX598519 |
| A/chicken/Hubei/SIC7/2013 | H9N2 | Avian |  |  | KX598686 | KX598644 | KX598728 | KX598602 | KX598560 | KX598518 |
| A/chicken/Rizhao/1313/2013 | H9N2 | Avian |  |  | KF259454 | KF259973 | KF260217 | KF260461 | KF260705 | KF260949 |
| A/chicken/Rizhao/1339/2013 | H9N2 | Avian |  |  | KF259455 | KF259974 | KF260218 | KF260462 | KF260706 | KF260950 |
| A/chicken/Rizhao/1363/2013 | H9N2 | Avian |  |  | KF259456 | KF259975 | KF260219 | KF260463 | KF260707 | KF260951 |
| A/chicken/Rizhao/1436/2013 | H9N2 | Avian |  |  | KF259457 | KF259976 | KF260220 | KF260464 | KF260708 | KF260952 |
| A/chicken/Rizhao/249/2013 | H9N2 | Avian |  |  | KF259448 | KF259967 | KF260211 | KF260455 | KF260699 | KF260943 |
| A/chicken/Rizhao/437/2013 | H9N2 | Avian |  |  | KF259449 | KF259968 | KF260212 | KF260456 | KF260700 | KF260944 |
| A/chicken/Rizhao/55/2013 | H9N2 | Avian |  |  | KF259446 | KF259965 | KF260209 | KF260453 | KF260697 | KF260941 |
| A/chicken/Rizhao/651/2013 | H9N2 | Avian |  |  | KF259450 | KF259969 | KF260213 | KF260457 | KF260701 | KF260945 |
| A/chicken/Rizhao/723/2013 | H9N2 | Avian |  |  | KF259451 | KF259970 | KF260214 | KF260458 | KF260702 | KF260946 |
| A/chicken/Rizhao/811/2013 | H9N2 | Avian |  |  | KF259452 | KF259971 | KF260215 | KF260459 | KF260703 | KF260947 |
| A/chicken/Rizhao/85/2013 | H9N2 | Avian |  |  | KF259447 | KF259966 | KF260210 | KF260454 | KF260698 | KF260942 |
| A/chicken/Rizhao/853/2013 | H9N2 | Avian |  |  | KF259453 | KF259972 | KF260216 | KF260460 | KF260704 | KF260948 |
| A/chicken/Wenzhou/253/2013 | H9N2 | Avian |  |  | KF259437 | KF259956 | KF260200 | KF260444 | KF260688 | KF260932 |
| A/chicken/Wenzhou/598/2013 | H9N2 | Avian |  |  | KF259436 | KF259955 | KF260199 | KF260443 | KF260687 | KF260931 |
| A/chicken/Wenzhou/606/2013 | H9N2 | Avian |  |  | KF259438 | KF259957 | KF260201 | KF260445 | KF260689 | KF260933 |
| A/chicken/Wenzhou/614/2013 | H9N2 | Avian |  |  | KF259439 | KF259958 | KF260202 | KF260446 | KF260690 | KF260934 |
| A/quail/Zhejiang/A1/2013 | H9N2 | Avian |  |  | KU042405 | KU042299 | KU042458 | KU042193 | KU042140 | KU042087 |
| A/silkie_chicken/Wenzhou/812/2013 | H9N2 | Avian |  |  | KF259441 | KF259960 | KF260204 | KF260448 | KF260692 | KF260936 |
| A/chicken/Fujian/SIC16/2014 | H9N2 | Avian |  |  | KX598690 | KX598648 | KX598732 | KX598606 | KX598564 | KX598522 |
| A/chicken/Guangdong/SIC17/2014 | H9N2 | Avian |  |  | KX598703 | KX598661 | KX598745 | KX598619 | KX598577 | KX598535 |
| A/chicken/Guangdong/SIC18/2014 | H9N2 | Avian |  |  | KX598704 | KX598662 | KX598746 | KX598620 | KX598578 | KX598536 |
| A/chicken/Guangxi/SIC19/2014 | H9N2 | Avian |  |  | KX598701 | KX598659 | KX598743 | KX598617 | KX598575 | KX598533 |
| A/chicken/Guangxi/SIC20/2014 | H9N2 | Avian |  |  | KX598702 | KX598660 | KX598744 | KX598618 | KX598576 | KX598534 |
| A/chicken/Shandong/SIC21/2014 | H9N2 | Avian |  |  | KX598692 | KX598650 | KX598734 | KX598608 | KX598566 | KX598524 |
| A/goose/MN/5733-1/1980 | H9N2 | Avian |  |  | CY005874 | CY005876 | CY005877 | CY005878 | CY005879 | CY005880 |
| A/chicken/Hebei/4/2008 | H9N2 | Avian |  |  | FJ499466 | FJ499469 | FJ499465 | FJ499470 | FJ499467 | FJ499468 |
| A/chicken/Hebei/TS/2008 | H9N2 | Avian |  |  | KC821177 | KC821140 | KC821251 | KC821103 | KC821070 | KC821029 |
| A/chicken/Fujian/01/2009 | H9N2 | Avian |  |  | KJ426493 | KJ426517 | KJ426277 | KJ000697 | KJ426299 | KJ426311 |
| A/chicken/Henan/DY/2009 | H9N2 | Avian |  |  | KC821182 | KC821145 | KC821256 | KC821108 | KC821071 | KC821034 |
| A/chicken/Shandong/sd01/2010 | H9N2 | Avian |  |  | KC821186 | KC821151 | KC821260 | KC821116 | KC821075 | KC821042 |
| A/chicken/El_Fayoum/CAI25/2011 | H9N2 | Avian |  |  | CY126233 | CY126235 | CY126236 | CY126237 | CY126238 | CY126239 |
| A/chicken/Guangdong/01/2011 | H9N2 | Avian |  |  | KC821198 | KC821161 | KC821273 | KC821128 | KC821091 | KC821059 |
| A/chicken/Guangdong/01/2008 | H9N2 | Avian |  |  | KJ426499 | KJ426523 | KJ426283 | KJ426293 | KJ426305 | KJ426317 |
| A/chicken/Guangdong/V/2008 | H9N2 | Avian |  |  | JQ639789 | JQ639787 | JQ639790 | JQ639785 | JQ639784 | JQ639783 |
| A/chicken/Egypt/S7018B/2013 | H9N2 | Avian |  |  | KF881508 | KF881506 | KF881509 | KF881505 | KF881504 | KF881503 |
| A/chicken/Egypt/S7022D/2013 | H9N2 | Avian |  |  | KF881643 | KF881641 | KF881644 | KF881639 | KF881638 | KF881637 |
| A/chicken/Egypt/S7025E/2013 | H9N2 | Avian |  |  | KF881389 | KF881387 | KF881390 | KF881385 | KF881384 | KF881383 |
| A/egret/Hunan/1/2012 | H9N2 | Avian |  |  | JX437684 | JX437689 | JX437687 | JX437688 | JX437686 | JX437691 |
| A/chicken/Jiangxi/1202/2014 | H9N2 | Avian |  |  | KP285352 | KP285350 | KP285353 | KP285348 | KP285347 | KP285346 |
| A/chicken/Shandong/yt0106/2012 | H9N2 | Avian |  |  | KM609603 | KM609683 | KM609723 | KM609763 | KM609803 | KM609843 |
| A/chicken/Henan/Z01/2007 | H9N2 | Avian |  |  | KJ426503 | KJ426527 | KJ426287 | KJ426297 | KJ426309 | KJ426321 |
| A/chicken/Jiangsu/NTTZ/2013 | H9N2 | avian |  |  | EPI_ISL_145390 | EPI_ISL_145390 | EPI_ISL_145390 | EPI_ISL_145390 | EPI_ISL_145390 | EPI_ISL_145390 |
| A/chicken/Shandong/qd0107/2013 | H9N2 | Avian |  |  | KM609621 | KM609701 | KM609741 | KM609781 | KM609821 | KM609861 |
| A/chicken/Pakistan/UDL-01/2008 | H9N2 | Avian |  |  | CY038461 | CY038459 | CY038462 | CY038457 | CY038456 | CY038455 |
| A/chicken/Henan/1.2/2008 | H9N2 | Avian |  |  | FJ534541 | FJ534544 | FJ534540 | FJ534545 | FJ534542 | FJ534543 |
| A/chicken/Qingdao/001/2014 | H9N2 | Avian |  |  | KT449592 | KT449632 | KT449652 | KT449672 | KT449692 | KT449712 |
| A/chicken/Qingdao/002/2014 | H9N2 | Avian |  |  | KT449593 | KT449633 | KT449653 | KT449673 | KT449693 | KT449713 |
| A/chicken/Qingdao/003/2014 | H9N2 | Avian |  |  | KT449594 | KT449634 | KT449654 | KT449674 | KT449694 | KT449714 |
| A/chicken/Qingdao/004/2014 | H9N2 | Avian |  |  | KT449595 | KT449635 | KT449655 | KT449675 | KT449695 | KT449715 |
| A/chicken/Qingdao/005/2014 | H9N2 | Avian |  |  | KT449596 | KT449636 | KT449656 | KT449676 | KT449696 | KT449716 |
| A/chicken/Qingdao/006/2014 | H9N2 | Avian |  |  | KT449597 | KT449637 | KT449657 | KT449677 | KT449697 | KT449717 |
| A/chicken/Qingdao/007/2014 | H9N2 | Avian |  |  | KT449598 | KT449638 | KT449658 | KT449678 | KT449698 | KT449718 |
| A/chicken/Qingdao/008/2014 | H9N2 | Avian |  |  | KT449599 | KT449639 | KT449659 | KT449679 | KT449699 | KT449719 |
| A/chicken/Qingdao/009/2014 | H9N2 | Avian |  |  | KT449600 | KT449640 | KT449660 | KT449680 | KT449700 | KT449720 |
| A/chicken/Qingdao/010/2014 | H9N2 | Avian |  |  | KT449601 | KT449641 | KT449661 | KT449681 | KT449701 | KT449721 |
| A/chicken/Qingdao/011/2014 | H9N2 | Avian |  |  | KT449602 | KT449642 | KT449662 | KT449682 | KT449702 | KT449722 |
| A/chicken/Qingdao/012/2014 | H9N2 | Avian |  |  | KT449603 | KT449643 | KT449663 | KT449683 | KT449703 | KT449723 |
| A/chicken/Qingdao/013/2014 | H9N2 | Avian |  |  | KT449604 | KT449644 | KT449664 | KT449684 | KT449704 | KT449724 |
| A/chicken/Qingdao/014/2014 | H9N2 | Avian |  |  | KT449605 | KT449645 | KT449665 | KT449685 | KT449705 | KT449725 |
| A/chicken/Qingdao/015/2014 | H9N2 | Avian |  |  | KT449606 | KT449646 | KT449666 | KT449686 | KT449706 | KT449726 |
| A/chicken/Qingdao/017/2014 | H9N2 | Avian |  |  | KT449608 | KT449648 | KT449668 | KT449688 | KT449708 | KT449728 |
| A/chicken/Qingdao/018/2014 | H9N2 | Avian |  |  | KT449609 | KT449649 | KT449669 | KT449689 | KT449709 | KT449729 |
| A/chicken/Qingdao/019/2014 | H9N2 | Avian |  |  | KT449610 | KT449650 | KT449670 | KT449690 | KT449710 | KT449730 |
| A/chicken/Qingdao/020/2014 | H9N2 | Avian |  |  | KT449611 | KT449651 | KT449671 | KT449691 | KT449711 | KT449731 |
| A/duck/Qingdao/016/2014 | H9N2 | Avian |  |  | KT449607 | KT449647 | KT449667 | KT449687 | KT449707 | KT449727 |
| A/chicken/Jiangsu/01/2007 | H9N2 | Avian |  |  | KJ426504 | KJ426528 | KJ426288 | KJ426298 | KJ426310 | KJ426322 |
| A/chicken/India/80998/2008 | H9N2 | Avian |  |  | KM519968 | KM519966 | KM519969 | KM519964 | KM519963 | KM519962 |
| A/northern_shoveler/Arkansas/11OS386/2011 | H9N2 | Avian |  |  | CY133634 | CY133636 | CY133637 | CY133638 | CY133639 | CY133640 |
| A/chicken/Jiangsu/XZ38/2012 | H9N2 | avian |  |  | EPI_ISL_145363 | EPI_ISL_145363 | EPI_ISL_145363 | EPI_ISL_145363 | EPI_ISL_145363 | EPI_ISL_145363 |
| A/chicken/Jiangxi/506/2013 | H9N2 | Avian |  |  | KP285856 | KP285854 | KP285857 | KP285852 | KP285851 | KP285850 |
| A/chicken/Egypt/F9516B/2014 | H9N2 | Avian |  |  | KX000835 | KX000765 | KX000709 | KX000776 | KX000762 | KX000870 |
| A/chicken/Egypt/D7099/2013 | H9N2 | Avian |  |  | KF881620 | KF881618 | KF881621 | KF881617 | KF881616 | KF881615 |
| A/chicken/Egypt/D7100/2013 | H9N2 | Avian |  |  | KF881685 | KF881683 | KF881686 | KF881682 | KF881681 | KF881680 |
| A/chicken/Egypt/D7108E/2013 | H9N2 | Avian |  |  | KF881336 | KF881334 | KF881337 | KF881333 | KF881332 | KF881331 |
| A/Anser_fabalis/China/HuBS428/2014 | H9N2 | Avian |  |  | KM076707 | KM076705 | KM076708 | KM076703 | KM076702 | KM076701 |
| A/chicken/Jiangsu/SQ79/2011 | H9N2 | Avian |  |  | KJ426410 | KJ426432 | KJ426443 | KJ426454 | KJ426465 | KJ426476 |
| A/chicken/Egypt/NLQP139V-AR754/2013 | H9N2 | avian |  |  | EPI_ISL_170358 | EPI_ISL_170358 | EPI_ISL_170358 | EPI_ISL_170358 | EPI_ISL_170358 | EPI_ISL_170358 |
| A/chicken/Shandong/1/2008 | H9N2 | Avian |  |  | JQ904463 | JQ904462 | JQ904464 | JQ904461 | JQ904460 | JQ904459 |
| A/duck/Jiangsu/1/2008 | H9N2 | Avian |  |  | KF142484 | KF142482 | KF142485 | KF142480 | KF142479 | KF142478 |
| A/chicken/Israel/32/2009 | H9N2 | Avian |  |  | JQ254957 | JQ254938 | JQ254958 | JQ254959 | JQ254960 | JQ254961 |
| A/chicken/Shandong/KD/2009 | H9N2 | Avian |  |  | JF795105 | JF795103 | JF795106 | JF795101 | JF795100 | JF795099 |
| A/chicken/Guangdong/SIC1/2012 | H9N2 | Avian |  |  | KX598700 | KX598658 | KX598742 | KX598616 | KX598574 | KX598532 |
| A/chicken/Shanghai/C1/2012 | H9N2 | Avian |  |  | KC417049 | KC417055 | KC417058 | KC417061 | KC417064 | KC417067 |
| A/chicken/Shanghai/C2/2012 | H9N2 | Avian |  |  | KC417050 | KC417056 | KC417059 | KC417062 | KC417065 | KC417068 |
| A/chicken/Shanghai/C3/2012 | H9N2 | Avian |  |  | KC417051 | KC417057 | KC417060 | KC417063 | KC417066 | KC417069 |
| A/chicken/Guangdong/LG1/2013 | H9N2 | Avian |  |  | KC951125 | KC951123 | KC951126 | KC951121 | KC951120 | KC951119 |
| A/chicken/Sichuan/SIC8/2013 | H9N2 | Avian |  |  | KX598684 | KX598642 | KX598726 | KX598600 | KX598558 | KX598516 |
| A/chicken/Guangxi/SIC22/2014 | H9N2 | Avian |  |  | KX598694 | KX598652 | KX598736 | KX598610 | KX598568 | KX598526 |
| A/chicken/Guangdong/SIC37/2015 | H9N2 | Avian |  |  | KX598715 | KX598673 | KX598757 | KX598631 | KX598589 | KX598547 |
| A/chicken/Guangdong/SIC38/2015 | H9N2 | Avian |  |  | KX598718 | KX598676 | KX598760 | KX598634 | KX598592 | KX598550 |
| A/chicken/Shandong/SIC39/2015 | H9N2 | Avian |  |  | KX598717 | KX598675 | KX598759 | KX598633 | KX598591 | KX598549 |
| A/chicken/Shanghai/02/2015 | H9N2 | Avian |  |  | KU720442 | KU720448 | KU720451 | KU720454 | KU720457 | KU720460 |
| A/chicken/Taizhou/TZJF05/2015 | H9N2 | Avian |  |  | KU143326 | KU143412 | KU143455 | KU143498 | KU143541 | KU143584 |
| A/chicken/Wuhan/JXQL01/2015 | H9N2 | Avian |  |  | KU143327 | KU143413 | KU143457 | KU143499 | KU143544 | KU143585 |
| A/chicken/Zhejiang/SIC40/2015 | H9N2 | Avian |  |  | KX598720 | KX598678 | KX598762 | KX598636 | KX598594 | KX598552 |
| A/chicken/Jiangsu/B20/2011 | H9N2 | Avian |  |  | KJ426411 | KJ426433 | KJ426444 | KJ426455 | KJ426466 | KJ426477 |
| A/chicken/Jiangsu/DTNSZ/2011 | H9N2 | avian |  |  | EPI_ISL_145378 | EPI_ISL_145378 | EPI_ISL_145378 | EPI_ISL_145378 | EPI_ISL_145378 | EPI_ISL_145378 |
| A/chicken/India/01CL1619/2012 | H9N2 | Avian |  |  | KT285352 | KT285350 | KT285353 | KT285348 | KT285347 | KT285346 |
| A/chicken/India/01CL1826/2012 | H9N2 | Avian |  |  | KT285344 | KT285342 | KT285345 | KT285340 | KT285339 | KT285338 |
| A/chicken/Henan/01/2009 | H9N2 | Avian |  |  | KJ426495 | KJ426519 | KJ426279 | KJ426290 | KJ426301 | KJ426313 |
| A/chicken/Egypt/NLQP73VD-AR755/2013 | H9N2 | avian |  |  | EPI_ISL_170359 | EPI_ISL_170359 | EPI_ISL_170359 | EPI_ISL_170359 | EPI_ISL_170359 | EPI_ISL_170359 |
| A/chicken/Jiangsu/CZJT2/2013 | H9N2 | avian |  |  | EPI_ISL_145387 | EPI_ISL_145387 | EPI_ISL_145387 | EPI_ISL_145387 | EPI_ISL_145387 | EPI_ISL_145387 |
| A/chicken/Jiangsu/ZJ4/2013 | H9N2 | avian |  |  | EPI_ISL_145388 | EPI_ISL_145388 | EPI_ISL_145388 | EPI_ISL_145388 | EPI_ISL_145388 | EPI_ISL_145388 |
| A/chicken/Hubei/ZYSJF15/2016 | H9N2 | Avian |  |  | KY415660 | KY415748 | KY415924 | KY415792 | KY415836 | KY415880 |
| A/duck/Guangxi/NN/2006 | H9N2 | Avian |  |  | KF768217 | KF768222 | KF768216 | KF768218 | KF768220 | KF768221 |
| A/chicken/Shangdong/SDWD/2011 | H9N2 | avian |  |  | EPI_ISL_145367 | EPI_ISL_145367 | EPI_ISL_145367 | EPI_ISL_145367 | EPI_ISL_145367 | EPI_ISL_145367 |
| A/gadwall_duck/Netherlands/2/2006 | H9N2 | Avian |  |  | KX977763 | KX977909 | KX978755 | KX977949 | KX979696 | KX978047 |
| A/chicken/Ganzhou/GZ86/2016 | H9N2 | Avian |  |  | KY415683 | KY415771 | KY415947 | KY415815 | KY415859 | KY415903 |
| A/duck/Ganzhou/GZ188/2016 | H9N2 | Avian |  |  | KY415684 | KY415772 | KY415948 | KY415816 | KY415860 | KY415904 |
| A/chicken/Bangladesh/18857/2013 | H9N2 | Avian |  |  | KJ643811 | KJ643809 | KJ643812 | KJ643807 | KJ643806 | KJ643805 |
| A/chicken/Egypt/NLQP194V-AR756/2013 | H9N2 | avian |  |  | EPI_ISL_170360 | EPI_ISL_170360 | EPI_ISL_170360 | EPI_ISL_170360 | EPI_ISL_170360 | EPI_ISL_170360 |
| A/chicken/Zhejiang/221/2016 | H9N2 | Avian |  |  | KY056312 | KY056298 | KY056319 | KY056284 | KY056277 | KY056270 |
| A/chicken/Shandong/zc2/2009 | H9N2 | Avian |  |  | KC821183 | KC821146 | KC821257 | KC821109 | KC821072 | KC821035 |
| A/chicken/Shandong/sd02/2010 | H9N2 | Avian |  |  | KC821187 | KC821152 | KC821261 | KC821117 | KC821082 | KC821038 |
| A/chicken/Shandong/02/2011 | H9N2 | Avian |  |  | KC821208 | KC821162 | KC821275 | KC821129 | KC821092 | KC821050 |
| A/chicken/Shandong/B2/2011 | H9N2 | Avian |  |  | KJ426409 | KJ426431 | KJ426442 | KJ426453 | KJ426464 | KJ426475 |
| A/chicken/Sichuan/02/2011 | H9N2 | Avian |  |  | KC821202 | KC821163 | KC821282 | KC821130 | KC821096 | KC821062 |
| A/chicken/Hunan/1/2012 | H9N2 | Avian |  |  | KF714778 | KF714776 | KF714779 | KF714774 | KF714773 | KF714772 |
| A/chicken/Beijing/11/2013 | H9N2 | Avian |  |  | KF059291 | KF059311 | KF059321 | KF059331 | KF059341 | KF059351 |
| A/chicken/Jiangsu/TM55/2013 | H9N2 | Avian |  |  | KJ426336 | KJ426354 | KJ426363 | KJ426372 | KJ426381 | KJ426390 |
| A/chicken/Jiangsu/02/2008 | H9N2 | Avian |  |  | KJ426500 | KJ426524 | KJ426284 | KJ426294 | KJ426306 | KJ426318 |
| A/chicken/Shandong/wf0202/2012 | H9N2 | Avian |  |  | KM609608 | KM609688 | KM609728 | KM609768 | KM609808 | KM609848 |
| A/chicken/Anhui/02/2008 | H9N2 | Avian |  |  | KJ426497 | KJ426521 | KJ426281 | KJ426291 | KJ426303 | KJ426315 |
| A/chicken/Egypt/D4692A/2012 | H9N2 | Avian |  |  | KF881514 | KF881513 | KF881515 | KF881512 | KF881511 | KF881510 |
| A/chicken/Egypt/F7297B/2013 | H9N2 | Avian |  |  | KF881381 | KF881379 | KF881382 | KF881377 | KF881376 | KF881375 |
| A/chicken/Fujian/C1161/2013 | H9N2 | Avian |  |  | KM113272 | KM113270 | KM113269 | KM113268 | KM113267 | KM113266 |
| A/chicken/Beijing/3/1999 | H9N2 | Avian |  |  | KP055064 | KP055062 | KP055065 | KP055060 | KP055059 | KP055058 |
| A/chicken/Jiangsu/XZ57/2012 | H9N2 | Avian |  |  | KJ426407 | KJ426429 | KJ426440 | KJ426451 | KJ426462 | KJ426473 |
| A/chicken/Egypt/NLQP257V-AR757/2013 | H9N2 | avian |  |  | EPI_ISL_170361 | EPI_ISL_170361 | EPI_ISL_170361 | EPI_ISL_170361 | EPI_ISL_170361 | EPI_ISL_170361 |
| A/chicken/Jiangsu/DT0112/2012 | H9N2 | avian |  |  | EPI_ISL_145376 | EPI_ISL_145376 | EPI_ISL_145376 | EPI_ISL_145376 | EPI_ISL_145376 | EPI_ISL_145376 |
| A/chicken/Egypt/NLQP123VD-AR758/2013 | H9N2 | avian |  |  | EPI_ISL_170362 | EPI_ISL_170362 | EPI_ISL_170362 | EPI_ISL_170362 | EPI_ISL_170362 | EPI_ISL_170362 |
| A/chicken/Jiangxi/6719/2013 | H9N2 | Avian |  |  | KP285864 | KP285862 | KP285865 | KP285860 | KP285859 | KP285858 |
| A/chicken/Israel/184/2009 | H9N2 | Avian |  |  | GQ148823 | GQ148837 | GQ148851 | GQ140280 | GQ148865 | GQ140266 |
| A/pigeon/Nanchang/2-0461/2000 | H9N2 | Avian |  |  | CY005507 | CY005509 | CY006020 | CY005510 | CY005511 | CY005512 |
| A/quail/Nanchang/2-0460/2000 | H9N2 | Avian |  |  | CY005501 | CY005503 | CY006019 | CY005504 | CY005505 | CY005506 |
| A/wild_duck/Nanchang/2-0480/2000 | H9N2 | Avian |  |  | CY005513 | CY005515 | CY006022 |  | CY005517 | CY005518 |
| A/duck/Guangxi/RX/2009 | H9N2 | Avian |  |  | KF768232 | KF768235 | KF768233 | KF768237 | KF768234 | KF768230 |
| A/chicken/Yuhuan/YH15/2016 | H9N2 | Avian |  |  | KY415673 | KY415761 | KY415937 | KY415805 | KY415849 | KY415893 |
| A/chicken/Egypt/S7404/2013 | H9N2 | Avian |  |  | KF881462 | KF881460 | KF881463 | KF881458 | KF881457 | KF881456 |
| A/chicken/Jiangxi/8209/2013 | H9N2 | Avian |  |  | KP285072 | KP285070 | KP285073 | KP285068 | KP285067 | KP285066 |
| A/chicken/Jiangxi/8281/2013 | H9N2 | Avian |  |  | KP285872 | KP285870 | KP285873 | KP285868 | KP285867 | KP285866 |
| A/duck/Jiangxi/8314/2013 | H9N2 | Avian |  |  | KP285744 | KP285742 | KP285745 | KP285740 | KP285739 | KP285738 |
| A/duck/Jiangxi/8358/2013 | H9N2 | Avian |  |  | KP285752 | KP285750 | KP285753 | KP285748 | KP285747 | KP285746 |
| A/Chicken/Jilin/13200/2014 | H9N2 | avian |  |  | EPI_ISL_161678 | EPI_ISL_161678 | EPI_ISL_161678 | EPI_ISL_161678 | EPI_ISL_161678 | EPI_ISL_161678 |
| A/chicken/Shandong/01/2009 | H9N2 | Avian |  |  | JF795147 | JF795145 | JF795148 | JF795143 | JF795142 | JF795141 |
| A/chicken/Iran/EBGV-88/2010 | H9N2 | Avian |  |  | JX465630 | JX465628 | JQ364984 | JX465624 | JX465622 | JX465620 |
| A/chicken/Jiangsu/Q3/2010 | H9N2 | Avian |  |  | JN869533 | JN869531 | JN869534 | JN869530 | JN869529 | JN869528 |
| A/chicken/Shandong/02/2010 | H9N2 | Avian |  |  | JF795065 | JF795063 | JF795066 | JF795061 | JF795060 | JF795059 |
| A/chicken/Zhejiang/Q1D4/2010 | H9N2 | Avian |  |  | JN869526 | JN869524 | JN869527 | JN869523 | JN869522 | JN869521 |
| A/chicken/Fujian/SIC9/2013 | H9N2 | Avian |  |  | KX598698 | KX598656 | KX598740 | KX598614 | KX598572 | KX598530 |
| A/chicken/Guangdong/SIC10/2013 | H9N2 | Avian |  |  | KX598691 | KX598649 | KX598733 | KX598607 | KX598565 | KX598523 |
| A/chicken/Guangdong/SIC23/2014 | H9N2 | Avian |  |  | KX598719 | KX598677 | KX598761 | KX598635 | KX598593 | KX598551 |
| A/chicken/Guangdong/SIC41/2015 | H9N2 | Avian |  |  | KX598714 | KX598672 | KX598756 | KX598630 | KX598588 | KX598546 |
| A/chicken/Hubei/01/1999 | H9N2 | Avian |  |  | CY077087 | CY077085 | CY077088 | CY077083 | CY077081 | CY077082 |
| A/chicken/Jiangsu/WJ58/2012 | H9N2 | Avian |  |  | KJ426408 | KJ426430 | KJ426441 | KJ426452 | KJ426463 | KJ426474 |
| A/chicken/Dongguan/1674/2014 | H9N2 | Avian |  |  | KP416449 | KP416447 | KP416450 | KP416445 | KP416444 | KP416443 |
| A/Chicken/Jilin/13204/2014 | H9N2 | avian |  |  | EPI_ISL_161677 | EPI_ISL_161677 | EPI_ISL_161677 | EPI_ISL_161677 | EPI_ISL_161677 | EPI_ISL_161677 |
| A/chicken/Pakistan/UDL-02/2008 | H9N2 | Avian |  |  | CY038469 | CY038467 | CY038470 | CY038465 | CY038464 | CY038463 |
| A/chicken/Sihala/NARC-12103/2008 | H9N2 | Avian |  |  | JN540069 | JN540070 | JN540068 | JN540071 | JN540072 | JN540073 |
| A/chicken/Ganzhou/GZ140/2016 | H9N2 | Avian |  |  | KY415686 | KY415774 | KY415950 | KY415818 | KY415862 | KY415906 |
| A/chicken/Jilin/SD001/2014 | H9N2 | Avian |  |  | KM054789 | KM054791 | KM054792 | KM054793 | KM054794 | KM054795 |
| A/chicken/Egypt/D7436C/2013 | H9N2 | Avian |  |  | KF881556 | KF881554 | KF881557 | KF881552 | KF881551 | KF881550 |
| A/chicken/Hebei/DF/2008 | H9N2 | Avian |  |  | KC821178 | KC821141 | KC821252 | KC821104 | KC821066 | KC821033 |
| A/chicken/Shandong/03/2008 | H9N2 | Avian |  |  | KJ426498 | KJ426522 | KJ426282 | KJ426292 | KJ426304 | KJ426316 |
| A/chicken/Shandong/03/2011 | H9N2 | Avian |  |  | KC821203 | KC821164 | KC821283 | KC821131 | KC821093 | KC821060 |
| A/wild_waterfowl/Dongting/C4296/2012 | H9N2 | Avian |  |  | KF972008 | KF972006 | KF972009 | KF972004 | KF972003 | KF972002 |
| A/wild_waterfowl/Dongting/C4316/2012 | H9N2 | Avian |  |  |  | KF972014 |  | KF972012 | KF972011 | KF972010 |
| A/wild_waterfowl/Dongting/C4317/2012 | H9N2 | Avian |  |  | KF972024 | KF972022 | KF972025 | KF972020 | KF972019 | KF972018 |
| A/wild_waterfowl/Dongting/C4326/2012 | H9N2 | Avian |  |  | KF972032 | KF972030 | KF972033 | KF972028 | KF972027 | KF972026 |
| A/wild_waterfowl/Dongting/C4327/2012 | H9N2 | Avian |  |  | KF972040 | KF972038 | KF972041 | KF972036 | KF972035 | KF972034 |
| A/wild_waterfowl/Dongting/C4329/2012 | H9N2 | Avian |  |  | KF972048 | KF972046 | KF972049 | KF972044 | KF972043 | KF972042 |
| A/wild_waterfowl/Dongting/C4429/2012 | H9N2 | Avian |  |  | KF972056 | KF972054 | KF972057 | KF972052 | KF972051 | KF972050 |
| A/wild_waterfowl/Dongting/C4430/2012 | H9N2 | Avian |  |  | KF972064 | KF972062 | KF972065 | KF972060 | KF972059 | KF972058 |
| A/wild_waterfowl/Dongting/PC2539/2012 | H9N2 | Avian |  |  | KF972072 | KF972070 | KF972073 | KF972068 | KF972067 | KF972066 |
| A/wild_waterfowl/Dongting/PC2540/2012 | H9N2 | Avian |  |  | KF972080 | KF972078 | KF972081 | KF972076 | KF972075 | KF972074 |
| A/wild_waterfowl/Dongting/PC2553/2012 | H9N2 | Avian |  |  | KF972088 | KF972086 | KF972089 | KF972084 | KF972083 | KF972082 |
| A/wild_waterfowl/Dongting/PC2559/2012 | H9N2 | Avian |  |  | KF972096 | KF972094 | KF972097 | KF972092 | KF972091 | KF972090 |
| A/wild_waterfowl/Dongting/PC2560/2012 | H9N2 | Avian |  |  | KF972104 | KF972102 | KF972105 | KF972100 | KF972099 | KF972098 |
| A/wild_waterfowl/Dongting/PC2562/2012 | H9N2 | Avian |  |  | KF972112 | KF972110 | KF972113 | KF972108 | KF972107 | KF972106 |
| A/wild_waterfowl/Dongting/PC2574/2012 | H9N2 | Avian |  |  | KF972120 | KF972118 | KF972121 | KF972116 | KF972115 | KF972114 |
| A/chicken/Guangdong/TS/2004 | H9N2 | Avian |  |  | JQ639781 | JQ639779 | JQ639782 | JQ639777 | JQ639776 | JQ639775 |
| A/chicken/Shandong/sx01/2008 | H9N2 | Avian |  |  | KM977628 | KM977626 | KM977629 | KM977624 | KM977623 | KM977622 |
| A/chicken/Jiangsu/JT34/2011 | H9N2 | Avian |  |  | KJ426413 | KJ426435 | KJ426446 | KJ426457 | KJ426468 | KJ426479 |
| A/chicken/Jiangxi/9696/2013 | H9N2 | Avian |  |  | KP285880 | KP285878 | KP285881 | KP285876 | KP285875 | KP285874 |
| A/chicken/Pakistan/UDL-03/2008 | H9N2 | Avian |  |  | CY038477 | CY038475 | CY038478 | CY038473 | CY038472 | CY038471 |
| A/duck/Bangladesh/1009/2009 | H9N2 | avian |  |  | EPI_ISL_142873 | EPI_ISL_142873 | EPI_ISL_142873 | EPI_ISL_142873 | EPI_ISL_142873 | EPI_ISL_142873 |
| A/chicken/Egypt/NLQP758V-AR759/2013 | H9N2 | avian |  |  | EPI_ISL_170363 | EPI_ISL_170363 | EPI_ISL_170363 | EPI_ISL_170363 | EPI_ISL_170363 | EPI_ISL_170363 |
| A/chicken/Israel/375/2007 | H9N2 | Avian |  |  | GQ148826 | GQ148840 | GQ148854 | GQ140283 | GQ148868 | GQ140269 |
| A/chicken/Shandong/qd0307/2013 | H9N2 | Avian |  |  | KM609618 | KM609698 | KM609738 | KM609778 | KM609818 | KM609858 |
| A/chicken/Beijing/0309/2013 | H9N2 | Avian |  |  | KM609639 | KM609719 | KM609759 | KM609799 | KM609839 | KM609879 |
| A/duck/Guangxi/NN/2005 | H9N2 | Avian |  |  | KF768243 | KF768238 | KF768241 | KF768242 | KF768244 | KF768245 |
| A/chicken/Jiangxi/10269/2013 | H9N2 | Avian |  |  | KP285760 | KP285758 | KP285761 | KP285756 | KP285755 | KP285754 |
| A/chicken/Jiangxi/10278/2013 | H9N2 | Avian |  |  | KP416657 | KP416655 | KP416658 | KP416653 | KP416652 | KP416651 |
| A/chicken/Ganzhou/GZ126/2016 | H9N2 | Avian |  |  | KY415687 | KY415775 | KY415951 | KY415819 | KY415863 | KY415907 |
| A/chicken/Egypt/D4905B/2012 | H9N2 | Avian |  |  | KF881454 | KF881452 | KF881455 | KF881451 | KF881450 | KF881449 |
| A/chicken/Egypt/D4907A/2012 | H9N2 | Avian |  |  | KF881678 | KF881677 | KF881679 | KF881676 | KF881675 | KF881674 |
| A/chicken/Shandong/qd0312/2013 | H9N2 | Avian |  |  | KM609617 | KM609697 | KM609737 | KM609777 | KM609817 | KM609857 |
| A/chicken/Egypt/NLQP426V-AR760/2013 | H9N2 | avian |  |  | EPI_ISL_170364 | EPI_ISL_170364 | EPI_ISL_170364 | EPI_ISL_170364 | EPI_ISL_170364 | EPI_ISL_170364 |
| A/chicken/Egypt/NLQP488V-AR761/2013 | H9N2 | avian |  |  | EPI_ISL_170365 | EPI_ISL_170365 | EPI_ISL_170365 | EPI_ISL_170365 | EPI_ISL_170365 | EPI_ISL_170365 |
| A/turtledove/Guangxi/49B6/2013 | H9N2 | Avian |  |  | KJ725015 | KJ725013 | KJ725016 | KJ725011 | KJ725010 | KJ725009 |
| A/chicken/Jiangsu/TM38/2013 | H9N2 | Avian |  |  | KJ426335 | KJ426353 | KJ426362 | KJ426371 | KJ426380 | KJ426389 |
| A/chicken/Jiangsu/03/2007 | H9N2 | Avian |  |  | KJ426502 | KJ426526 | KJ426286 | KJ426296 | KJ426308 | KJ426320 |
| A/chicken/China/Guangxi1/2000 | H9N2 | Avian |  |  | DQ485211 | DQ485209 | DQ485212 | DQ485207 | DQ485206 | DQ485205 |
| A/chicken/Yunnan/C1212/2010 | H9N2 | Avian |  |  | KM113264 | KM113262 | KM113261 | KM113260 | KM113259 | KM113258 |
| A/chicken/Zhejiang/C1219/2010 | H9N2 | Avian |  |  | KM113256 | KM113254 | KM113253 | KM113252 | KM113251 | KM113250 |
| A/chicken/Chongqing/C1258/2011 | H9N2 | Avian |  |  | KM113240 | KM113238 | KM113237 | KM113236 | KM113235 | KM113234 |
| A/chicken/Guangdong/C1122/2011 | H9N2 | Avian |  |  | KM113288 | KM113286 | KM113285 | KM113284 | KM113283 | KM113282 |
| A/chicken/Zhejiang/C1083/2011 | H9N2 | Avian |  |  | KM113304 | KM113302 | KM113301 | KM113300 | KM113299 | KM113298 |
| A/duck/Hubei/C1146/2011 | H9N2 | Avian |  |  | KM113280 | KM113278 | KM113277 | KM113276 | KM113275 | KM113274 |
| A/black-billed_magpie/Guangxi/29/2005 | H9N2 | Avian |  |  | GU121385 | GU121383 | GU121386 | GU121381 | GU121380 | GU121379 |
| A/chicken/Hebei/7/2008 | H9N2 | Avian |  |  | GQ202051 | GQ202054 | GQ202050 | GQ202055 | GQ202052 | GQ202053 |
| A/chicken/Hebei/Y2/2009 | H9N2 | Avian |  |  | GQ202039 | GQ202042 | GQ202038 | GQ202043 | GQ202040 | GQ202041 |
| A/chicken/Zhejiang/329/2011 | H9N2 | Avian |  |  | JQ356890 | JQ356878 | JQ356893 | JQ356881 | JQ356887 | JQ356884 |
| A/wild_chicken/Shanghai/C1/2014 | H9N2 | Avian |  |  | KJ726700 | KJ726712 | KJ726718 | KJ726724 | KJ726730 | KJ726736 |
| A/chicken/Pakistan/UDL-04/2007 | H9N2 | Avian |  |  | CY038485 | CY038483 | CY038486 | CY038481 | CY038480 | CY038479 |
| A/chicken/Guangxi/C1435/2012 | H9N2 | Avian |  |  | KM113232 | KM113230 | KM113229 | KM113228 | KM113227 | KM113226 |
| A/chicken/Jiangsu/CZLJG/2012 | H9N2 | avian |  |  | EPI_ISL_145372 | EPI_ISL_145372 | EPI_ISL_145372 | EPI_ISL_145372 | EPI_ISL_145372 | EPI_ISL_145372 |
| A/chicken/Jiangsu/CZYWP/2012 | H9N2 | avian |  |  | EPI_ISL_145379 | EPI_ISL_145379 | EPI_ISL_145379 | EPI_ISL_145379 | EPI_ISL_145379 | EPI_ISL_145379 |
| A/chicken/Jiangsu/MYJMF/2012 | H9N2 | avian |  |  | EPI_ISL_145382 | EPI_ISL_145382 | EPI_ISL_145382 | EPI_ISL_145382 | EPI_ISL_145382 | EPI_ISL_145382 |
| A/chicken/Jiangsu/WJHDL/2012 | H9N2 | avian |  |  | EPI_ISL_145361 | EPI_ISL_145361 | EPI_ISL_145361 | EPI_ISL_145361 | EPI_ISL_145361 | EPI_ISL_145361 |
| A/chicken/Jiangsu/WJHRG/2012 | H9N2 | avian |  |  | EPI_ISL_145365 | EPI_ISL_145365 | EPI_ISL_145365 | EPI_ISL_145365 | EPI_ISL_145365 | EPI_ISL_145365 |
| A/chicken/Jiangsu/WJYBF/2012 | H9N2 | avian |  |  | EPI_ISL_145384 | EPI_ISL_145384 | EPI_ISL_145384 | EPI_ISL_145384 | EPI_ISL_145384 | EPI_ISL_145384 |
| A/chicken/Jiangsu/XZWHL/2012 | H9N2 | avian |  |  | EPI_ISL_145374 | EPI_ISL_145374 | EPI_ISL_145374 | EPI_ISL_145374 | EPI_ISL_145374 | EPI_ISL_145374 |
| A/chicken/Jiangsu/XZZSL/2012 | H9N2 | avian |  |  | EPI_ISL_145373 | EPI_ISL_145373 | EPI_ISL_145373 | EPI_ISL_145373 | EPI_ISL_145373 | EPI_ISL_145373 |
| A/chicken/Beijing/16/2013 | H9N2 | Avian |  |  | KM609634 | KM609714 | KM609754 | KM609794 | KM609834 | KM609874 |
| A/chicken/Jiangxi/11708/2013 | H9N2 | Avian |  |  | KP285344 | KP285342 | KP285345 | KP285340 | KP285339 | KP285338 |
| A/chicken/Fujian/C1239/2009 | H9N2 | Avian |  |  | KM113248 | KM113246 | KM113245 | KM113244 | KM113243 | KM113242 |
| A/chicken/Guangdong/C1095/2009 | H9N2 | Avian |  |  | KM113296 | KM113294 | KM113293 | KM113292 | KM113291 | KM113290 |
| A/chicken/Guizhou/C1011/2009 | H9N2 | Avian |  |  | KM113320 | KM113318 | KM113317 | KM113316 | KM113315 | KM113314 |
| A/duck/Zhejiang/C1036/2009 | H9N2 | Avian |  |  | KM113312 | KM113310 | KM113309 | KM113308 | KM113307 | KM113306 |
| A/chicken/Egypt/S5018C/2012 | H9N2 | Avian |  |  | KF881486 | KF881484 | KF881487 | KF881482 | KF881481 | KF881480 |
| A/chicken/Egypt/S5018D/2012 | H9N2 | Avian |  |  | KF881397 | KF881395 | KF881398 | KF881393 | KF881392 | KF881391 |
| A/chicken/Jiangsu/XZPZ/2011 | H9N2 | avian |  |  | EPI_ISL_145369 | EPI_ISL_145369 | EPI_ISL_145369 | EPI_ISL_145369 | EPI_ISL_145369 | EPI_ISL_145369 |
| A/chicken/Jiangsu/ZJDT1/2013 | H9N2 | avian |  |  | EPI_ISL_145385 | EPI_ISL_145385 | EPI_ISL_145385 | EPI_ISL_145385 | EPI_ISL_145385 | EPI_ISL_145385 |
| A/duck/Fujian/MH/2003 | H9N2 | Avian |  |  | JF916724 | JF916722 | JF916725 | JF916720 | JF916719 | JF916718 |
| A/chicken/Shandong/zc4/2012 | H9N2 | Avian |  |  | KF059285 | KF059305 | KF059315 | KF059325 | KF059335 | KF059345 |
| A/chicken/Shandong/ZL1/2013 | H9N2 | Avian |  |  | KJ426334 | KJ426352 | KJ426361 | KJ426370 | KJ426379 | KJ426388 |
| A/quail/Hangzhou/35/2013 | H9N2 | avian |  |  | EPI_ISL_141773 | EPI_ISL_141773 | EPI_ISL_141773 | EPI_ISL_141773 | EPI_ISL_141773 | EPI_ISL_141773 |
| A/duck/Egypt/C9787/2014 | H9N2 | Avian |  |  | KX000728 | KX000767 | KX000747 | KX000755 | KX000841 | KX000804 |
| A/chicken/Jiangsu/DD6/2013 | H9N2 | Avian |  |  | KJ426338 | KJ426356 | KJ426365 | KJ426374 | KJ426383 | KJ426392 |
| A/chicken/Jiangsu/HA2/2013 | H9N2 | avian |  |  | EPI_ISL_145391 | EPI_ISL_145391 | EPI_ISL_145391 | EPI_ISL_145391 | EPI_ISL_145391 | EPI_ISL_145391 |
| A/duck/Jiangsu/YC2/2013 | H9N2 | avian |  |  | EPI_ISL_145389 | EPI_ISL_145389 | EPI_ISL_145389 | EPI_ISL_145389 | EPI_ISL_145389 | EPI_ISL_145389 |
| A/chicken/Jiangxi/12562/2013 | H9N2 | Avian |  |  | KP285768 | KP285766 | KP285769 | KP285764 | KP285763 | KP285762 |
| A/environment/Zhenjiang/h9/2013 | H9N2 | avian |  |  | EPI_ISL_145081 | EPI_ISL_145081 | EPI_ISL_145081 | EPI_ISL_145081 | EPI_ISL_145081 | EPI_ISL_145081 |
| A/chicken/Zhejiang/C482/2013 | H9N2 | Avian |  |  | KU042421 | KU042315 | KU042474 | KU042209 | KU042156 | KU042103 |
| A/chicken/Zhejiang/C485/2013 | H9N2 | Avian |  |  | KU042422 | KU042316 | KU042475 | KU042210 | KU042157 | KU042104 |
| A/chicken/Zhejiang/C488/2013 | H9N2 | Avian |  |  | KU042423 | KU042317 | KU042476 | KU042211 | KU042158 | KU042105 |
| A/chicken/Zhejiang/C489/2013 | H9N2 | Avian |  |  | KU042424 | KU042318 | KU042477 | KU042212 | KU042159 | KU042106 |
| A/pigeon/Zhejiang/P3/2013 | H9N2 | Avian |  |  | KU042416 | KU042310 | KU042469 | KU042204 | KU042151 | KU042098 |
| A/pigeon/Zhejiang/P4/2013 | H9N2 | Avian |  |  | KU042417 | KU042311 | KU042470 | KU042205 | KU042152 | KU042099 |
| A/pigeon/Zhejiang/P5/2013 | H9N2 | Avian |  |  | KU042418 | KU042312 | KU042471 | KU042206 | KU042153 | KU042100 |
| A/quail/Zhejiang/A2/2013 | H9N2 | Avian |  |  | KU042406 | KU042300 | KU042459 | KU042194 | KU042141 | KU042088 |
| A/chicken/Egypt/D7663C/2013 | H9N2 | Avian |  |  | KF881373 | KF881371 | KF881374 | KF881369 | KF881368 | KF881367 |
| A/chicken/Zhejiang/C4910/2013 | H9N2 | Avian |  |  | KU042427 | KU042321 | KU042480 | KU042215 | KU042162 | KU042109 |
| A/chicken/Zhejiang/C493/2013 | H9N2 | Avian |  |  | KU042425 | KU042319 | KU042478 | KU042213 | KU042160 | KU042107 |
| A/chicken/Zhejiang/C496/2013 | H9N2 | Avian |  |  | KU042426 | KU042320 | KU042479 | KU042214 | KU042161 | KU042108 |
| A/chicken/Hangzhou/48-2/2013 | H9N2 | avian |  |  | EPI_ISL_142591 | EPI_ISL_142591 | EPI_ISL_142591 | EPI_ISL_142591 | EPI_ISL_142591 | EPI_ISL_142591 |
| A/chicken/Hangzhou/50-2/2013 | H9N2 | avian |  |  | EPI_ISL_142592 | EPI_ISL_142592 | EPI_ISL_142592 | EPI_ISL_142592 | EPI_ISL_142592 | EPI_ISL_142592 |
| A/chicken/Quang_Ninh/26/2013 | H9N2 | Avian |  |  | AB836742 | AB836740 | AB836743 | AB836738 | AB836737 | AB836736 |
| A/muscovy_duck/Quang_Ninh/11/2013 | H9N2 | Avian |  |  | LC028358 | LC028356 | LC028359 | LC028354 | LC028353 | LC028352 |
| A/chicken/Zhejiang/3C14/2014 | H9N2 | Avian |  |  | KU042623 | KU042587 | KU042641 | KU042551 | KU042533 | KU042515 |
| A/chicken/Zhejiang/3C28/2014 | H9N2 | Avian |  |  | KU042624 | KU042588 | KU042642 | KU042552 | KU042534 | KU042516 |
| A/chicken/Zhejiang/3C34/2014 | H9N2 | Avian |  |  | KU042625 | KU042589 | KU042643 | KU042553 | KU042535 | KU042517 |
| A/chicken/Zhejiang/3C37/2014 | H9N2 | Avian |  |  | KU042626 | KU042590 | KU042644 | KU042554 | KU042536 | KU042518 |
| A/chicken/Zhejiang/3C9/2014 | H9N2 | Avian |  |  | KU042622 | KU042586 | KU042640 | KU042550 | KU042532 | KU042514 |
| A/chicken/Jiangsu/XZMG/2012 | H9N2 | avian |  |  | EPI_ISL_145368 | EPI_ISL_145368 | EPI_ISL_145368 | EPI_ISL_145368 | EPI_ISL_145368 | EPI_ISL_145368 |
| A/chicken/Shaanxi/xa0414/2013 | H9N2 | Avian |  |  | KM609625 | KM609705 | KM609745 | KM609785 | KM609825 | KM609865 |
| A/turkey/Israel/689/2008 | H9N2 | Avian |  |  | GQ148827 | GQ148841 | GQ148855 | GQ140284 | GQ148869 | GQ140270 |
| A/chicken/Jiangsu/XZLZG/2012 | H9N2 | avian |  |  | EPI_ISL_145381 | EPI_ISL_145381 | EPI_ISL_145381 | EPI_ISL_145381 | EPI_ISL_145381 | EPI_ISL_145381 |
| A/chicken/Shandong/qd01417/2013 | H9N2 | Avian |  |  | KM609620 | KM609700 | KM609740 | KM609780 | KM609820 | KM609860 |
| A/turkey/Poland/08/2014 | H9N2 | Avian |  |  |  | KX470443 | KX470446 | KX470441 | KX470440 | KX470439 |
| A/chicken/Israel/694/2008 | H9N2 | Avian |  |  | GQ148828 | GQ148842 | GQ148856 | GQ140285 | GQ148870 | GQ140271 |
| A/turkey/Poland/09/2014 | H9N2 | Avian |  |  |  | KX470451 | KX470454 | KX470449 | KX470448 | KX470447 |
| A/chicken/Anhui/1/2008 | H9N2 | Avian |  |  | KP081536 | KP081534 | KP081537 | KP081532 | KP081531 | KP081530 |
| A/chicken/Hebei/C4/2008 | H9N2 | Avian |  |  | GQ202045 | GQ202048 | GQ202044 | GQ202049 | GQ202046 | GQ202047 |
| A/chicken/Korea/KNUSWR09/2009 | H9N2 | Avian |  |  | JN852796 | JN852798 | JN852799 | JN852800 | JN852801 | JN852802 |
| A/chicken/Jiangsu/SIC11/2013 | H9N2 | Avian |  |  | KX598721 | KX598679 | KX598763 | KX598637 | KX598595 | KX598553 |
| A/chicken/Shanghai/020/2013 | H9N2 | Avian |  |  | KF500978 | KF500980 | KF500981 | KF500982 | KF500983 | KF500984 |
| A/chicken/Zhejiang/C484/2013 | H9N2 | Avian |  |  | KF042118 | KF042147 | KF042128 | KF042138 | KF042133 | KF042113 |
| A/chicken/Zhejiang/C487/2013 | H9N2 | Avian |  |  | KF042119 | KF042148 | KF042129 | KF042139 | KF042134 | KF042114 |
| A/chicken/Zhejiang/C494/2013 | H9N2 | Avian |  |  | KF042120 | KF042149 | KF042130 | KF042140 | KF042135 | KF042115 |
| A/chicken/Zhejiang/C497/2013 | H9N2 | Avian |  |  | KF042121 | KF042150 | KF042131 | KF042141 | KF042136 | KF042116 |
| A/pigeon/Shanghai/JC1/2013 | H9N2 | Avian |  |  | KJ128363 | KJ128365 | KJ128366 | KJ128367 | KJ128368 | KJ128369 |
| A/chicken/Jiangsu/SIC42/2015 | H9N2 | Avian |  |  | KX598723 | KX598681 | KX598765 | KX598639 | KX598597 | KX598555 |
| A/chicken/Israel/702/2008 | H9N2 | Avian |  |  | GQ148829 | GQ148843 | GQ148857 | GQ140286 | GQ148871 | GQ140272 |
| A/quail/Egypt/D9842/2014 | H9N2 | Avian |  |  | KX000771 | KX000733 | KX000725 | KX000740 | KX000869 | KX000720 |
| A/turkey/Poland/14/2013 | H9N2 | Avian |  |  |  | KX470435 | KX470438 | KX470433 | KX470432 | KX470431 |
| A/duck/Fujian/C2246/2009 | H9N2 | Avian |  |  | KM113192 | KM113190 | KM113189 | KM113188 | KM113187 | KM113186 |
| A/chicken/Nanchang/4-301/2001 | H9N2 | Avian |  |  | CY005519 | CY005521 | CY006024 | CY005522 | CY005523 | CY005524 |
| A/chicken/Shandong/qd0427/2012 | H9N2 | Avian |  |  | KM609616 | KM609696 | KM609736 | KM609776 | KM609816 | KM609856 |
| A/chicken/Egypt/F10993B/2015 | H9N2 | Avian |  |  | KX000847 | KX000867 | KX000783 | KX000825 | KX000769 | KX000706 |
| A/chicken/Hebei/fx05/2010 | H9N2 | Avian |  |  | KC821189 | KC821149 | KC821263 | KC821119 | KC821085 | KC821048 |
| A/chicken/Shandong/05/2010 | H9N2 | Avian |  |  | KC821188 | KC821153 | KC821262 | KC821118 | KC821083 | KC821039 |
| A/chicken/Shandong/05/2011 | H9N2 | Avian |  |  | KC821204 | KC821168 | KC821284 | KC821132 | KC821094 | KC821061 |
| A/chicken/Jiangsu/YZLH3/2013 | H9N2 | Avian |  |  | KJ426337 | KJ426355 | KJ426364 | KJ426373 | KJ426382 | KJ426391 |
| A/chicken/Pakistan/UDL-01/2006 | H9N2 | Avian |  |  | CY038429 | CY038427 | CY038430 | CY038425 | CY038424 | CY038423 |
| A/sparrow/Shanghai/09/2013 | H9N2 | Avian |  |  | KP412435 | KP412433 | KP412436 | KP412431 | KP412430 | KP412429 |
| A/chicken/Shandong/1167/2015 | H9N2 | Avian |  |  | KT338303 | KT338301 | KT338304 | KT338299 | KT338298 | KT338297 |
| A/muscovy_duck/Vietnam/LBM417/2013 | H9N2 | Avian |  |  | AB841275 | AB841273 | AB841276 | AB841271 | AB841270 | AB841269 |
| A/chicken/Shandong/SDWF17/2012 | H9N2 | avian |  |  | EPI_ISL_145364 | EPI_ISL_145364 | EPI_ISL_145364 | EPI_ISL_145364 | EPI_ISL_145364 | EPI_ISL_145364 |
| A/chicken/Shangdong/SDWF14/2012 | H9N2 | avian |  |  | EPI_ISL_145366 | EPI_ISL_145366 | EPI_ISL_145366 | EPI_ISL_145366 | EPI_ISL_145366 | EPI_ISL_145366 |
| A/chicken/Shangdong/SDWF29/2012 | H9N2 | avian |  |  | EPI_ISL_145375 | EPI_ISL_145375 | EPI_ISL_145375 | EPI_ISL_145375 | EPI_ISL_145375 | EPI_ISL_145375 |
| A/Chinese_francolin/Guangxi/B7/2010 | H9N2 | Avian |  |  | KF768200 | KF768201 | KF768205 | KF768199 | KF768202 | KF768206 |
| A/chicken/Shandong/Li-2/2010 | H9N2 | Avian |  |  | KM411631 | KM411629 | KM411632 | KM411627 | KM411626 | KM411625 |
| A/chicken/Jiangsu/YZ0511/2011 | H9N2 | avian |  |  | EPI_ISL_145380 | EPI_ISL_145380 | EPI_ISL_145380 | EPI_ISL_145380 | EPI_ISL_145380 | EPI_ISL_145380 |
| A/chicken/Pakistan/10A/2015 | H9N2 | Avian |  |  | KU042913 | KU042911 | KU042914 | KU042909 | KU042908 | KU042907 |
| A/chicken/Jiangsu/CZJJF/2012 | H9N2 | avian |  |  | EPI_ISL_145383 | EPI_ISL_145383 | EPI_ISL_145383 | EPI_ISL_145383 | EPI_ISL_145383 | EPI_ISL_145383 |
| A/chicken/Jiangsu/CZZWQ/2012 | H9N2 | avian |  |  | EPI_ISL_145362 | EPI_ISL_145362 | EPI_ISL_145362 | EPI_ISL_145362 | EPI_ISL_145362 | EPI_ISL_145362 |
| A/chicken/Beijing/0512/2013 | H9N2 | Avian |  |  | KM609636 | KM609716 | KM609756 | KM609796 | KM609836 | KM609876 |
| A/chicken/Chongqing/C2093/2013 | H9N2 | Avian |  |  | KM113216 | KM113214 | KM113213 | KM113212 | KM113211 | KM113210 |
| A/chicken/Hubei/SC122/2013 | H9N2 | Avian |  |  | KM113080 | KM113078 | KM113077 | KM113076 | KM113075 | KM113074 |
| A/chicken/Jiangsu/SC502/2013 | H9N2 | Avian |  |  | KM113048 | KM113046 | KM113045 | KM113044 | KM113043 | KM113042 |
| A/chicken/Shanghai/SC197/2013 | H9N2 | Avian |  |  | KM113072 | KM113070 | KM113069 | KM113068 | KM113067 | KM113066 |
| A/chicken/Shanghai/SC387/2013 | H9N2 | Avian |  |  | KM113056 | KM113054 | KM113053 | KM113052 | KM113051 | KM113050 |
| A/chicken/Zhejiang/SC324/2013 | H9N2 | Avian |  |  | KM113064 | KM113062 | KM113061 | KM113060 | KM113059 | KM113058 |
| A/chicken/Shandong/WF0513/2012 | H9N2 | Avian |  |  | KJ426333 | KJ426351 | KJ426360 | KJ426369 | KJ426378 | KJ426387 |
| A/chicken/Shandong/wf0514/2013 | H9N2 | Avian |  |  | KM609607 | KM609687 | KM609727 | KM609767 | KM609807 | KM609847 |
| A/chicken/Shandong/qd0516/2012 | H9N2 | Avian |  |  | KM609615 | KM609695 | KM609735 | KM609775 | KM609815 | KM609855 |
| A/chicken/Liaoning/0517/2013 | H9N2 | Avian |  |  | KM609628 | KM609708 | KM609748 | KM609788 | KM609828 | KM609868 |
| A/chicken/Pakistan/UDL-01/2005 | H9N2 | Avian |  |  | CY038413 | CY038411 | CY038414 | CY038409 | CY038408 | CY038407 |
| A/chicken/Guangxi/C2163/2012 | H9N2 | Avian |  |  | KM113200 | KM113198 | KM113197 | KM113196 | KM113195 | KM113194 |
| A/chicken/Sichuan/C2151/2012 | H9N2 | Avian |  |  | KM113208 | KM113206 | KM113205 | KM113204 | KM113203 | KM113202 |
| A/duck/Zhejiang/C2046/2012 | H9N2 | Avian |  |  | KM113224 | KM113222 | KM113221 | KM113220 | KM113219 | KM113218 |
| A/cackling_goose/Alaska/UGAI15-3075/2015 | H9N2 | Avian |  |  | KU310458 | KU310456 | KU310459 | KU310454 | KU310453 | KU310452 |
| A/shorebird/Delaware_Bay/127/2003 | H9N2 | Avian |  |  | CY102729 | CY102731 | CY102732 | CY102733 | CY102734 | CY102735 |
| A/chicken/Jilin/0519/2012 | H9N2 | Avian |  |  | KM609630 | KM609710 | KM609750 | KM609790 | KM609830 | KM609870 |
| A/chicken/Pakistan/13A/2015 | H9N2 | Avian |  |  | KU042905 | KU042903 | KU042906 | KU042901 | KU042900 | KU042899 |
| A/black-billed_magpie/Guangxi/30/2005 | H9N2 | Avian |  |  | HM590765 | HM590763 | HM590766 | HM590761 | HM590760 | HM590759 |
| A/black-billed_magpie/Guangxi/31/2005 | H9N2 | Avian |  |  | HM590772 | HM590771 | HM590773 | HM590769 | HM590768 | HM590767 |
| A/chicken/Korea/KNUWSJ09/2009 | H9N2 | Avian |  |  | JN852804 | JN852806 | JN852807 | JN852808 | JN852809 | JN852810 |
| A/chicken/Shandong/H/2009 | H9N2 | Avian |  |  | JF795097 | JF795095 | JF795098 | JF795093 | JF795092 | JF795091 |
| A/duck/Korea/KNUDPJ09/2009 | H9N2 | Avian |  |  | JN852780 | JN852782 | JN852783 | JN852784 | JN852785 | JN852786 |
| A/chicken/Shandong/03/2010 | H9N2 | Avian |  |  | JF795073 | JF795071 | JF795074 | JF795069 | JF795068 | JF795067 |
| A/chicken/Shandong/BD/2010 | H9N2 | Avian |  |  | JF795089 | JF795087 | JF795090 | JF795085 | JF795084 | JF795083 |
| A/chicken/Shandong/SIC24/2014 | H9N2 | Avian |  |  | KX598709 | KX598667 | KX598751 | KX598625 | KX598583 | KX598541 |
| A/chicken/Shandong/SIC25/2014 | H9N2 | Avian |  |  | KX598710 | KX598668 | KX598752 | KX598626 | KX598584 | KX598542 |
| A/chicken/Shanghai/06/2015 | H9N2 | Avian |  |  | KU720443 | KU720449 | KU720452 | KU720455 | KU720458 | KU720461 |
| A/chicken/Shanghai/15/2015 | H9N2 | Avian |  |  | KU720444 | KU720450 | KU720453 | KU720456 | KU720459 | KU720462 |
| A/ruddy_turnstone/Delaware/AI03-114/2003 | H9N2 | Avian |  |  | CY144484 | CY144486 | CY144487 | CY144488 | CY144489 | CY144490 |
| A/chicken/Jiangsu/ZJDT/2011 | H9N2 | avian |  |  | EPI_ISL_145370 | EPI_ISL_145370 | EPI_ISL_145370 | EPI_ISL_145370 | EPI_ISL_145370 | EPI_ISL_145370 |
| A/shorebird/Delaware/249/2006 | H9N2 | Avian |  |  | CY043913 | CY043915 | CY043916 | CY043917 | CY043918 | CY043919 |
| A/greater_white-fronted_goose/Alaska/81081/2008 | H9N2 | Avian |  |  | KX377333 | KX377331 | KX377334 | KX377329 | KX377328 | KX377327 |
| A/sanderling/Delaware_Bay/449/2006 | H9N2 | Avian |  |  | CY103000 | CY077145 | CY077146 | CY103004 | CY103005 | CY077149 |
| A/ruddy_turnstone/New_Jersey/AI06-539/2006 | H9N2 | Avian |  |  | CY144700 | CY144702 | CY144703 | CY144704 | CY144705 | CY144706 |
| A/chicken/Guangxi/067C4/2010 | H9N2 | Avian |  |  | KF768225 | KF768227 | KF768228 | KF768229 | KF768223 | KF768224 |
| A/chicken/Bangladesh/19870/2013 | H9N2 | Avian |  |  | KJ643600 | KJ643598 | KJ643601 | KJ643596 | KJ643595 | KJ643594 |
| A/chicken/Egypt/S5440E/2012 | H9N2 | Avian |  |  | KF881478 | KF881476 | KF881479 | KF881474 | KF881473 | KF881472 |
| A/chicken/Egypt/S5442C/2012 | H9N2 | Avian |  |  | KF881577 | KF881575 | KF881578 | KF881573 | KF881572 | KF881571 |
| A/chicken/Egypt/S5442E/2012 | H9N2 | Avian |  |  | KF881735 | KF881733 | KF881736 | KJ561639 | KF881731 | KF881730 |
| A/chicken/Hubei/2014 | H9N2 | Avian |  |  | KT164854 | KT164852 | KT164855 | KT164850 | KT164849 | KT164848 |
| A/chicken/Israel/54/2008 | H9N2 | Avian |  |  | GQ148821 | GQ148835 | GQ148849 | GQ140278 | GQ148863 | GQ140264 |
| A/chicken/Shandong/06/2010 | H9N2 | Avian |  |  | KC821190 | KC821150 | KC821264 | KC821120 | KC821081 | KC821047 |
| A/chicken/Shandong/06/2011 | H9N2 | Avian |  |  | KC821200 | KC821165 | KC821279 | KC821133 | KC821098 | KC821052 |
| A/pigeon/Pakistan/25A/2015 | H9N2 | Avian |  |  | KU042921 | KU042919 | KU042922 | KU042917 | KU042916 | KU042915 |
| A/chicken/Jiangsu/YZ0657/2012 | H9N2 | Avian |  |  | KJ426340 | KJ426358 | KJ426367 | KJ426376 | KJ426385 | KJ426394 |
| A/chicken/Egypt/D5490B/2012 | H9N2 | Avian |  |  | KF881344 | KF881342 | KF881345 | KF881340 | KF881339 | KF881338 |
| A/muscovy_duck/Vietnam/LBM675/2014 | H9N2 | Avian |  |  | AB983225 | AB983223 | AB983226 | AB983221 | AB983220 | AB983219 |
| A/duck/Japan/AQ-HE5/2015 | H9N2 | Avian |  |  | LC208506 | LC208505 | LC208507 | LC208504 | LC208503 | LC208502 |
| A/chicken/Shandong/zc0606/2012 | H9N2 | Avian |  |  | KM609601 | KM609681 | KM609721 | KM609761 | KM609801 | KM609841 |
| A/chicken/Pakistan/UDL-01/2007 | H9N2 | Avian |  |  | CY038397 | CY038395 | CY038398 | CY038393 | CY038392 | CY038391 |
| A/chicken/Israel/182/2008 | H9N2 | Avian |  |  | GQ148822 | GQ148836 | GQ148850 | GQ140279 | GQ148864 | GQ140265 |
| A/chicken/Guangxi/066C10/2010 | H9N2 | Avian |  |  | KF768183 | KF768185 | KF768184 | KF768186 | KF768190 | KF768189 |
| A/turkey/Wisconsin/1/1966 | H9N2 | Avian |  |  | CY087825 | CY130057 | CY130058 | CY130059 | CY130060 | CY014670 |
| A/chicken/Jiangsu/YZ0611/2011 | H9N2 | avian |  |  | EPI_ISL_145377 | EPI_ISL_145377 | EPI_ISL_145377 | EPI_ISL_145377 | EPI_ISL_145377 | EPI_ISL_145377 |
| A/chicken/Japan/AQ-HE14/2015 | H9N2 | Avian |  |  | LC208474 | LC208473 | LC208475 | LC208472 | LC208471 | LC208470 |
| A/chicken/Israel/883/2008 | H9N2 | Avian |  |  | GQ148831 | GQ148845 | GQ148859 | GQ140288 | GQ148873 | GQ140274 |
| A/chicken/Shandong/0613/2012 | H9N2 | Avian |  |  | KJ426332 | KJ426350 | KJ426359 | KJ426368 | KJ426377 | KJ426386 |
| A/chicken/Jiangsu/XZ0616/2012 | H9N2 | Avian |  |  | KJ426339 | KJ426357 | KJ426366 | KJ426375 | KJ426384 | KJ426393 |
| A/mallard/Portugal/83660/2009 | H9N2 | Avian |  |  | CY184158 | CY184160 | CY184161 | CY184162 | CY184163 | CY184164 |
| A/duck/Japan/AQ-HE28/2015 | H9N2 | Avian |  |  | LC208514 | LC208513 | LC208515 | LC208512 | LC208511 | LC208510 |
| A/chicken/Pakistan/UDL-04/2006 | H9N2 | Avian |  |  | CY038405 | CY038403 | CY038406 | CY038401 | CY038400 | CY038399 |
| A/chicken/Iran/ZMT-101/1998 | H9N2 | Avian |  |  | JX465629 | JX465627 | JQ364985 | JX465623 | JX465621 | JX465619 |
| A/chicken/Jiangsu/JS4539/2014 | H9N2 | Avian |  |  | KX867844 | KX867842 | KX867845 | KX867840 | KX867839 | KX867838 |
| A/chicken/Shanghai/A/2010 | H9N2 | Avian |  |  | KJ726695 | KJ726707 | KJ726713 | KJ726719 | KJ726725 | KJ726731 |
| A/chicken/Guangdong/ZHJ/2011 | H9N2 | Avian |  |  | JN869541 | JN869539 | JN869542 | JN869537 | JN869536 | JN869535 |
| A/chicken/Shanghai/C/2011 | H9N2 | Avian |  |  | KJ726697 | KJ726709 | KJ726715 | KJ726721 | KJ726727 | KJ726733 |
| A/chicken/Zhejiang/607/2011 | H9N2 | Avian |  |  | JQ356891 | JQ356879 | JQ356894 | JQ356882 | JQ356888 | JQ356885 |
| A/chicken/Zhejiang/611/2011 | H9N2 | Avian |  |  | JQ356892 | JQ356880 | JQ356895 | JQ356883 | JQ356889 | JQ356886 |
| A/chicken/Shandong/SIC26/2014 | H9N2 | Avian |  |  | KX598706 | KX598664 | KX598748 | KX598622 | KX598580 | KX598538 |
| A/chicken/Myanmar/NK-2/2015 | H9N2 | Avian |  |  | KY115370 | KY115368 | KY115371 | KY115366 | KY115365 | KY115364 |
| A/chicken/Myanmar/NK-4/2015 | H9N2 | Avian |  |  | KY115378 | KY115376 | KY115379 | KY115374 | KY115373 | KY115372 |
| A/chicken/Myanmar/NK-5/2015 | H9N2 | Avian |  |  | KY115386 | KY115384 |  | KY115382 | KY115381 | KY115380 |
| A/mallard/Portugal/83695/2009 | H9N2 | Avian |  |  | CY184166 | CY184168 | CY184169 | CY184170 | CY184171 | CY184172 |
| A/chicken/Jiangsu/YZ4/2011 | H9N2 | Avian |  |  | KJ426412 | KJ426434 | KJ426445 | KJ426456 | KJ426467 | KJ426478 |
| A/chicken/Jiangsu/C3089/2011 | H9N2 | Avian |  |  | KM113184 | KM113182 | KM113181 | KM113180 | KM113179 | KM113178 |
| A/chicken/Israel/310/2008 | H9N2 | Avian |  |  | GQ148824 | GQ148838 | GQ148852 | GQ140281 | GQ148866 | GQ140267 |
| A/chicken/Israel/330/2008 | H9N2 | Avian |  |  | GQ148825 | GQ148839 | GQ148853 | GQ140282 | GQ148867 | GQ140268 |
| A/chicken/Shandong/07/2009 | H9N2 | Avian |  |  | KJ426496 | KJ426520 | KJ426280 | KJ000701 | KJ426302 | KJ426314 |
| A/chicken/Shandong/07/2011 | H9N2 | Avian |  |  | KC821199 | KC821166 | KC821280 | KC821134 | KC821099 | KC821053 |
| A/mallard/Interior_Alaska/10BM02980R0/2010 | H9N2 | Avian |  |  | CY130454 | CY130456 | CY130457 | CY130458 | CY130459 | CY130460 |
| A/chicken/Shanxi/0703/2012 | H9N2 | Avian |  |  | KM609600 | KM609680 | KM609720 | KM609760 | KM609800 | KM609840 |
| A/chicken/Liaoning/0704/2012 | H9N2 | Avian |  |  | KM609627 | KM609707 | KM609747 | KM609787 | KM609827 | KM609867 |
| A/chicken/Zhejiang/77082/2014 | H9N2 | Avian |  |  | KU042627 | KU042591 | KU042645 | KU042555 | KU042537 | KU042519 |
| A/pigeon/Zhejiang/77037/2014 | H9N2 | Avian |  |  | KU042620 | KU042584 | KU042638 | KU042548 | KU042530 | KU042512 |
| A/chicken/Japan/AQ-HE61/2015 | H9N2 | Avian |  |  | LC208482 | LC208481 | LC208483 | LC208480 | LC208479 | LC208478 |
| A/chicken/Shanghai/014/2014 | H9N2 | Avian |  |  | KP404001 | KP404005 | KP404007 | KP404009 | KP404011 | KP404013 |
| A/chicken/Shanghai/015/2014 | H9N2 | Avian |  |  | KP404002 | KP404006 | KP404008 | KP404010 | KP404012 | KP404014 |
| A/muscovy_duck/Vietnam/LBM694/2014 | H9N2 | Avian |  |  | LC000599 | LC000597 | LC000600 | LC000595 | LC000594 | LC000593 |
| A/chicken/Jiangsu/07/2009 | H9N2 | Avian |  |  | KJ426494 | KJ426518 | KJ426278 | KJ426289 | KJ426300 | KJ426312 |
| A/chicken/Shandong/yt0711/2013 | H9N2 | Avian |  |  | KM609602 | KM609682 | KM609722 | KM609762 | KM609802 | KM609842 |
| A/chicken/Shandong/wf0712/2013 | H9N2 | Avian |  |  | KM609606 | KM609686 | KM609726 | KM609766 | KM609806 | KM609846 |
| A/chicken/Zhejiang/C71914/2013 | H9N2 | Avian |  |  | KU042430 | KU042324 | KU042483 | KU042218 | KU042165 | KU042112 |
| A/chicken/Zhejiang/C71916/2013 | H9N2 | Avian |  |  | KU042431 | KU042325 | KU042484 | KU042219 | KU042166 | KU042113 |
| A/chicken/Zhejiang/C71921/2013 | H9N2 | Avian |  |  | KU042432 | KU042326 | KU042485 | KU042220 | KU042167 | KU042114 |
| A/chicken/Zhejiang/C71926/2013 | H9N2 | Avian |  |  | KU042433 | KU042327 | KU042486 | KU042221 | KU042168 | KU042115 |
| A/chicken/Zhejiang/C7195/2013 | H9N2 | Avian |  |  | KU042428 | KU042322 | KU042481 | KU042216 | KU042163 | KU042110 |
| A/chicken/Zhejiang/C7199/2013 | H9N2 | Avian |  |  | KU042429 | KU042323 | KU042482 | KU042217 | KU042164 | KU042111 |
| A/chicken/Guangdong/ZCY/2011 | H9N2 | Avian |  |  | JN869549 | JN869547 | JN869550 | JN869545 | JN869544 | JN869543 |
| A/chicken/Shanghai/D/2011 | H9N2 | Avian |  |  | KJ726698 | KJ726710 | KJ726716 | KJ726722 | KJ726728 | KJ726734 |
| A/chicken/Guangdong/SIC27/2014 | H9N2 | Avian |  |  | KX598707 | KX598665 | KX598749 | KX598623 | KX598581 | KX598539 |
| A/chicken/Guangdong/SIC28/2014 | H9N2 | Avian |  |  | KX598708 | KX598666 | KX598750 | KX598624 | KX598582 | KX598540 |
| A/chicken/Hebei/0721/2013 | H9N2 | Avian |  |  | KM609632 | KM609712 | KM609752 | KM609792 | KM609832 | KM609872 |
| A/chicken/Jiangxi/17403/2013 | H9N2 | Avian |  |  | KP285776 | KP285774 | KP285777 | KP285772 | KP285771 | KP285770 |
| A/chicken/Anhui/AH120/2015 | H9N2 | Avian |  |  | KX867836 | KX867834 | KX867837 | KX867832 | KX867831 | KX867830 |
| A/chicken/Zhejiang/727063/2014 | H9N2 | Avian |  |  | KU042628 | KU042592 | KU042646 | KU042556 | KU042538 | KU042520 |
| A/chicken/Zhejiang/727192/2014 | H9N2 | Avian |  |  | KU042629 | KU042593 | KU042647 | KU042557 | KU042539 | KU042521 |
| A/chicken/Zhejiang/727198/2014 | H9N2 | Avian |  |  | KU042630 | KU042594 | KU042648 | KU042558 | KU042540 | KU042522 |
| A/duck/Zhejiang/727036/2014 | H9N2 | Avian |  |  | KU042619 | KU042583 | KU042637 | KU042547 | KU042529 | KU042511 |
| A/pigeon/Zhejiang/727044/2014 | H9N2 | Avian |  |  | KU042621 | KU042585 | KU042639 | KU042549 | KU042531 | KU042513 |
| A/chicken/Jiangxi/17913/2013 | H9N2 | Avian |  |  | KP285784 | KP285782 | KP285785 | KP285780 | KP285779 | KP285778 |
| A/chicken/Jiangxi/18445/2013 | H9N2 | Avian |  |  | KP285792 | KP285790 | KP285793 | KP285788 | KP285787 | KP285786 |
| A/chicken/Shandong/818/2012 | H9N2 | Avian |  |  | KM285400 | KM285398 | KM285401 | KM285396 | KM285395 | KM285394 |
| A/mallard/Alberta/11/1991 | H9N2 | Avian |  |  | CY005148 | CY005149 | CY005150 |  | CY005152 | CY005153 |
| A/mallard/Alberta/17/1991 | H9N2 | Avian |  |  | CY005154 | CY005156 | CY005157 | CY005158 | CY005159 | CY005160 |
| A/chicken/Jiangxi/18901/2013 | H9N2 | Avian |  |  | KP285800 | KP285798 | KP285801 | KP285796 | KP285795 | KP285794 |
| A/chicken/Jiangxi/18913/2013 | H9N2 | Avian |  |  | KP285080 | KP285078 | KP285081 | KP285076 | KP285075 | KP285074 |
| A/chicken/Jiangxi/18922/2013 | H9N2 | Avian |  |  | KP285088 | KP285086 | KP285089 | KP285084 | KP285083 | KP285082 |
| A/chicken/Jiangxi/18946/2013 | H9N2 | Avian |  |  | KP285096 | KP285094 | KP285097 | KP285092 | KP285091 | KP285090 |
| A/chicken/Jiangxi/18952/2013 | H9N2 | Avian |  |  | KP285104 | KP285102 | KP285105 | KP285100 | KP285099 | KP285098 |
| A/chicken/Jiangxi/18957/2013 | H9N2 | Avian |  |  | KP285112 | KP285110 | KP285113 | KP285108 | KP285107 | KP285106 |
| A/chicken/Jiangxi/18980/2013 | H9N2 | Avian |  |  | KP285120 | KP285118 | KP285121 | KP285116 | KP285115 | KP285114 |
| A/northern_pintail/Interior_Alaska/10BM14807R2/2010 | H9N2 | Avian |  |  | CY125751 | CY125753 | CY125754 | CY125755 | CY125756 | CY125757 |
| A/chicken/Jiangxi/19407/2013 | H9N2 | Avian |  |  | KP285808 | KP285806 | KP285809 | KP285804 | KP285803 | KP285802 |
| A/chicken/Jiangxi/19436/2013 | H9N2 | Avian |  |  | KP285128 | KP285126 | KP285129 | KP285124 | KP285123 | KP285122 |
| A/chicken/Jiangxi/19448/2013 | H9N2 | Avian |  |  | KP285136 | KP285134 | KP285137 | KP285132 | KP285131 | KP285130 |
| A/quail/Egypt/D10093/2014 | H9N2 | Avian |  |  | KX000833 | KX000818 | KX000724 | KX000730 | KX000772 | KX000731 |
| A/quail/Egypt/D10105/2014 | H9N2 | Avian |  |  | KX000819 | KX000809 | KX000780 | KX000800 | KX000711 | KX000784 |
| A/quail/Egypt/D10106/2014 | H9N2 | Avian |  |  | KX000834 | KX000810 | KX000705 | KX000737 | KX000826 | KX000827 |
| A/chicken/Shanghai/B/2010 | H9N2 | Avian |  |  | KJ726696 | KJ726708 | KJ726714 | KJ726720 | KJ726726 | KJ726732 |
| A/quail/Lebanon/272/2010 | H9N2 | Avian |  |  | CY093091 | CY093089 | CY093092 | CY093087 | CY093086 | CY093085 |
| A/quail/Lebanon/273/2010 | H9N2 | Avian |  |  | CY093099 | CY093097 | CY093100 | CY093095 | CY093094 | CY093093 |
| A/chicken/Jiangsu/SIC12/2013 | H9N2 | Avian |  |  | KX598688 | KX598646 | KX598730 | KX598604 | KX598562 | KX598520 |
| A/chicken/Guangdong/SIC29/2014 | H9N2 | Avian |  |  | KX598711 | KX598669 | KX598753 | KX598627 | KX598585 | KX598543 |
| A/chicken/Hangzhou/410/2013 | H9N2 | avian |  |  | EPI_ISL_169428 | EPI_ISL_169428 | EPI_ISL_169428 | EPI_ISL_169428 | EPI_ISL_169428 | EPI_ISL_169428 |
| A/mallard/California/3370/2012 | H9N2 | Avian |  |  | CY157535 | CY157537 | CY157538 | CY157539 | CY157540 | CY157541 |
| A/chicken/Jiangxi/19898/2013 | H9N2 | Avian |  |  | KP285816 | KP285814 | KP285817 | KP285812 | KP285811 | KP285810 |
| A/chicken/Jiangxi/19934/2013 | H9N2 | Avian |  |  | KP285144 | KP285142 | KP285145 | KP285140 | KP285139 | KP285138 |
| A/chicken/Jiangxi/19981/2013 | H9N2 | Avian |  |  | KP285152 | KP285150 | KP285153 | KP285148 | KP285147 | KP285146 |
| A/duck/Jiangxi/20147/2013 | H9N2 | Avian |  |  | KP285504 | KP285502 | KP285505 | KP285500 | KP285499 | KP285498 |
| A/chicken/Shandong/lc0830/2012 | H9N2 | Avian |  |  | KM609623 | KM609703 | KM609743 | KM609783 | KM609823 | KM609863 |
| A/northern_shoveler/Interior_Alaska/8BM3470/2008 | H9N2 | Avian |  |  | CY079647 | CY079649 | CY079650 | CY079651 | CY079652 | CY079653 |
| A/chicken/Shandong/09/2011 | H9N2 | Avian |  |  | KC821210 | KC821174 | KC821272 | KC821135 | KC821095 | KC821057 |
| A/chicken/Jiangxi/20443/2013 | H9N2 | Avian |  |  | KP285160 | KP285158 | KP285161 | KP285156 | KP285155 | KP285154 |
| A/chicken/Jiangxi/20446/2013 | H9N2 | Avian |  |  | KP285168 | KP285166 | KP285169 | KP285164 | KP285163 | KP285162 |
| A/chicken/Jiangxi/20457/2013 | H9N2 | Avian |  |  | KP285176 | KP285174 | KP285177 | KP285172 | KP285171 | KP285170 |
| A/chicken/Jiangxi/20478/2013 | H9N2 | Avian |  |  | KP285184 | KP285182 | KP285185 | KP285180 | KP285179 | KP285178 |
| A/chicken/Jiangxi/20482/2013 | H9N2 | Avian |  |  | KP285192 | KP285190 | KP285193 | KP285188 | KP285187 | KP285186 |
| A/chicken/Jiangxi/20489/2013 | H9N2 | Avian |  |  | KP285200 | KP285198 | KP285201 | KP285196 | KP285195 | KP285194 |
| A/chicken/Jiangxi/20506/2013 | H9N2 | Avian |  |  | KP285208 | KP285206 | KP285209 | KP285204 | KP285203 | KP285202 |
| A/mallard_duck/Alberta/321/1988 | H9N2 | Avian |  |  | CY005141 | CY005143 | CY005144 | CY005145 | CY005146 | CY005147 |
| A/chicken/Shandong/lc0903/2013 | H9N2 | Avian |  |  | KM609622 | KM609702 | KM609742 | KM609782 | KM609822 | KM609862 |
| A/mallard/PT/27972-B139/2007 | H9N2 | Avian |  |  | JF745933 | JF745932 | JF745934 | JF745930 | JF745929 | JF745928 |
| A/northern_shoveler/Interior_Alaska/10BM16764R0/2010 | H9N2 | Avian |  |  | CY125759 | CY125761 | CY125762 | CY125763 | CY125764 | CY125765 |
| A/chicken/Jiangxi/20927/2013 | H9N2 | Avian |  |  | KP285824 | KP285822 | KP285825 | KP285820 | KP285819 | KP285818 |
| A/chicken/Jiangxi/20935/2013 | H9N2 | Avian |  |  | KP285216 | KP285214 | KP285217 | KP285212 | KP285211 | KP285210 |
| A/chicken/Jiangxi/20976/2013 | H9N2 | Avian |  |  | KP285224 | KP285222 | KP285225 | KP285220 | KP285219 | KP285218 |
| A/Anas_platyrhynchos/Belgium/24311pcs5/2012 | H9N2 | Avian |  |  | KU646987 | KU646985 | KU646988 | KU646983 | KU646982 | KU646981 |
| A/chicken/Tibet/S1/2009 | H9N2 | Avian |  |  | CY087174 | CY087172 | CY087175 | CY087170 | CY087169 | CY087168 |
| A/chicken/Tibet/S4/2009 | H9N2 | Avian |  |  | CY087182 | CY087180 | CY087183 | CY087178 | CY087177 | CY087176 |
| A/duck/Tibet/S2/2009 | H9N2 | Avian |  |  | CY087190 | CY087188 | CY087191 | CY087186 | CY087185 | CY087184 |
| A/Muscovy_duck/Fujian/CL/1997 | H9N2 | Avian |  |  | JF916716 | JF916714 | JF916717 | JF916712 | JF916711 | JF916710 |
| A/chicken/Jiangxi/21468/2013 | H9N2 | Avian |  |  | KP285832 | KP285830 | KP285833 | KP285828 | KP285827 | KP285826 |
| A/chicken/Jiangxi/21481/2013 | H9N2 | Avian |  |  | KP285232 | KP285230 | KP285233 | KP285228 | KP285227 | KP285226 |
| A/muscovy_duck/Vietnam/LBM719/2014 | H9N2 | Avian |  |  | LC028179 | LC028177 | LC028180 | LC028175 | LC028174 | LC028173 |
| A/mallard/Sweden/99668/2009 | H9N2 | Avian |  |  | CY184174 | CY184176 | CY184177 | CY184178 | CY184179 | CY184180 |
| A/chicken/Shandong/qd0917/2013 | H9N2 | Avian |  |  | KM609613 | KM609693 | KM609733 | KM609773 | KM609813 | KM609853 |
| A/chicken/Jiangsu/DT/2009 | H9N2 | avian |  |  | EPI_ISL_145371 | EPI_ISL_145371 | EPI_ISL_145371 | EPI_ISL_145371 | EPI_ISL_145371 | EPI_ISL_145371 |
| A/chicken/Zhejiang/C3188/2010 | H9N2 | Avian |  |  | KM113176 | KM113174 | KM113173 | KM113172 | KM113171 | KM113170 |
| A/duck/Guangdong/C3204/2010 | H9N2 | Avian |  |  | KM113168 | KM113166 | KM113165 | KM113164 | KM113163 | KM113162 |
| A/chicken/Shandong/qd0920/2012 | H9N2 | Avian |  |  | KM609612 | KM609692 | KM609732 | KM609772 | KM609812 | KM609852 |
| A/chicken/Zhejiang/SIC30/2014 | H9N2 | Avian |  |  | KX598705 | KX598663 | KX598747 | KX598621 | KX598579 | KX598537 |
| A/chicken/Bangladesh/23426/2014 | H9N2 | Avian |  |  | KT362007 | KT362005 | KT362008 | KT362003 | KT362002 | KT362001 |
| A/chicken/Jiangxi/21978/2013 | H9N2 | Avian |  |  | KP285840 | KP285838 | KP285841 | KP285836 | KP285835 | KP285834 |
| A/chicken/Jiangxi/21984/2013 | H9N2 | Avian |  |  | KP285240 | KP285238 | KP285241 | KP285236 | KP285235 | KP285234 |
| A/emperor_goose/Alaska/2011-0713/2011 | H9N2 | Avian |  |  | KP336390 | KP336388 | KP336391 | KP336386 | KP336385 | KP336384 |
| A/chicken/India/09CL1359/2013 | H9N2 | Avian |  |  | KT285336 | KT285334 | KT285337 | KT285332 | KT285331 | KT285330 |
| A/duck/Hokkaido/K04/2014 | H9N2 | Avian |  |  | LC042046 | LC042044 | LC042047 | LC042042 | LC042041 | LC042040 |
| A/chicken/Zhejiang/925018/2014 | H9N2 | Avian |  |  | KU042631 | KU042595 | KU042649 | KU042559 | KU042541 | KU042523 |
| A/chicken/Zhejiang/925060/2014 | H9N2 | Avian |  |  | KU042632 | KU042596 | KU042650 | KU042560 | KU042542 | KU042524 |
| A/chicken/Zhejiang/925117/2014 | H9N2 | Avian |  |  | KU042633 | KU042597 | KU042651 | KU042561 | KU042543 | KU042525 |
| A/chicken/Zhejiang/925122/2014 | H9N2 | Avian |  |  | KU042634 | KU042598 | KU042652 | KU042562 | KU042544 | KU042526 |
| A/chicken/Zhejiang/925134/2014 | H9N2 | Avian |  |  | KU042635 | KU042599 | KU042653 | KU042563 | KU042545 | KU042527 |
| A/chicken/Zhejiang/925159/2014 | H9N2 | Avian |  |  | KU042636 | KU042600 | KU042654 | KU042564 | KU042546 | KU042528 |
| A/American_oystercatcher/Chile/C1307/2015 | H9N2 | Avian |  |  | KX185929 | KX185922 | KX185909 | KX185907 | KX185931 | KX185900 |
| A/mallard/Sweden/99785/2009 | H9N2 | Avian |  |  | CY184182 | CY184184 | CY184185 | CY184186 | CY184187 | CY184188 |
| A/chicken/Bangladesh/23527/2014 | H9N2 | Avian |  |  | KT362041 | KT362039 | KT362042 | KT362037 | KT362036 | KT362035 |
| A/northern_pintail/Alaska/2011-0703/2011 | H9N2 | Avian |  |  | KP336382 | KP336380 | KP336383 | KP336378 | KP336377 | KP336376 |
| A/chicken/Shandong/JL/2008 | H9N2 | Avian |  |  | KC821179 | KC821142 | KC821253 | KC821105 | KC821067 | KC821030 |
| A/chicken/Shandong/10/2010 | H9N2 | Avian |  |  | KC821191 | KC821154 | KC821265 | KC821121 | KC821077 | KC821046 |
| A/duck/Hong_Kong/784/1979 | H9N2 | Avian |  |  | CY005633 | CY005634 | CY005635 | CY005636 | CY005637 | CY005638 |
| A/bean_goose/Korea/220/2011 | H9N2 | Avian |  |  | KJ013300 | KJ013298 | KJ013301 | KJ013296 | KJ013295 | KJ013294 |
| A/mallard/Ohio/13OS3856/2013 | H9N2 | Avian |  |  | KJ568316 | KJ568314 | KJ568317 | KJ568312 | KJ568311 | KJ568310 |
| A/chicken/Shandong/qd1013/2012 | H9N2 | Avian |  |  | KM609611 | KM609691 | KM609731 | KM609771 | KM609811 | KM609851 |
| A/quail/Zhejiang/2A1-2/2013 | H9N2 | Avian |  |  | KU042407 | KU042301 | KU042460 | KU042195 | KU042142 | KU042089 |
| A/quail/Zhejiang/2A1-4/2013 | H9N2 | Avian |  |  | KU042408 | KU042302 | KU042461 | KU042196 | KU042143 | KU042090 |
| A/quail/Zhejiang/2A1-5/2013 | H9N2 | Avian |  |  | KU042409 | KU042303 | KU042462 | KU042197 | KU042144 | KU042091 |
| A/quail/Zhejiang/2A1-6/2013 | H9N2 | Avian |  |  | KU042410 | KU042304 | KU042463 | KU042198 | KU042145 | KU042092 |
| A/chicken/Zhejiang/C1/2013 | H9N2 | Avian |  |  | KU042434 | KU042328 | KU042487 | KU042222 | KU042169 | KU042116 |
| A/chicken/Zhejiang/C16/2013 | H9N2 | Avian |  |  | KU042437 | KU042331 | KU042490 | KU042225 | KU042172 | KU042119 |
| A/chicken/Zhejiang/C19/2013 | H9N2 | Avian |  |  | KU042438 | KU042332 | KU042491 | KU042226 | KU042173 | KU042120 |
| A/chicken/Zhejiang/C2/2013 | H9N2 | Avian |  |  | KU042435 | KU042329 | KU042488 | KU042223 | KU042170 | KU042117 |
| A/chicken/Zhejiang/C2-5/2013 | H9N2 | Avian |  |  | KU042449 | KU042343 | KU042502 | KU042237 | KU042184 | KU042131 |
| A/chicken/Zhejiang/C3/2013 | H9N2 | Avian |  |  | KU042436 | KU042330 | KU042489 | KU042224 | KU042171 | KU042118 |
| A/chicken/Zhejiang/C31/2013 | H9N2 | Avian |  |  | KU042439 | KU042333 | KU042492 | KU042227 | KU042174 | KU042121 |
| A/chicken/Zhejiang/C38/2013 | H9N2 | Avian |  |  | KU042440 | KU042334 | KU042493 | KU042228 | KU042175 | KU042122 |
| A/chicken/Zhejiang/C45/2013 | H9N2 | Avian |  |  | KU042441 | KU042335 | KU042494 | KU042229 | KU042176 | KU042123 |
| A/chicken/Zhejiang/C46/2013 | H9N2 | Avian |  |  | KU042442 | KU042336 | KU042495 | KU042230 | KU042177 | KU042124 |
| A/chicken/Zhejiang/C47/2013 | H9N2 | Avian |  |  | KU042443 | KU042337 | KU042496 | KU042231 | KU042178 | KU042125 |
| A/chicken/Zhejiang/C50/2013 | H9N2 | Avian |  |  | KU042444 | KU042338 | KU042497 | KU042232 | KU042179 | KU042126 |
| A/chicken/Zhejiang/C52/2013 | H9N2 | Avian |  |  | KU042445 | KU042339 | KU042498 | KU042233 | KU042180 | KU042127 |
| A/chicken/Zhejiang/C54/2013 | H9N2 | Avian |  |  | KU042446 | KU042340 | KU042499 | KU042234 | KU042181 | KU042128 |
| A/chicken/Zhejiang/C55/2013 | H9N2 | Avian |  |  | KU042447 | KU042341 | KU042500 | KU042235 | KU042182 | KU042129 |
| A/chicken/Zhejiang/C58/2013 | H9N2 | Avian |  |  | KU042448 | KU042342 | KU042501 | KU042236 | KU042183 | KU042130 |
| A/pigeon/Zhejiang/2P4/2013 | H9N2 | Avian |  |  | KU042419 | KU042313 | KU042472 | KU042207 | KU042154 | KU042101 |
| A/pigeon/Zhejiang/2P5/2013 | H9N2 | Avian |  |  | KU042420 | KU042314 | KU042473 | KU042208 | KU042155 | KU042102 |
| A/quail/Zhejiang/2A2/2013 | H9N2 | Avian |  |  | KU042411 | KU042305 | KU042464 | KU042199 | KU042146 | KU042093 |
| A/quail/Zhejiang/2A3/2013 | H9N2 | Avian |  |  | KU042412 | KU042306 | KU042465 | KU042200 | KU042147 | KU042094 |
| A/quail/Zhejiang/2A4/2013 | H9N2 | Avian |  |  | KU042413 | KU042307 | KU042466 | KU042201 | KU042148 | KU042095 |
| A/quail/Zhejiang/2A5/2013 | H9N2 | Avian |  |  | KU042414 | KU042308 | KU042467 | KU042202 | KU042149 | KU042096 |
| A/quail/Zhejiang/2A6/2013 | H9N2 | Avian |  |  | KU042415 | KU042309 | KU042468 | KU042203 | KU042150 | KU042097 |
| A/chicken/Bangladesh/23618/2014 | H9N2 | Avian |  |  | KT361999 | KT361997 | KT362000 | KT361995 | KT361994 | KT361993 |
| A/chicken/Hunan/C3247/2009 | H9N2 | Avian |  |  | KM113144 | KM113142 | KM113141 | KM113140 | KM113139 | KM113138 |
| A/chicken/Jiangxi/23773/2013 | H9N2 | Avian |  |  | KP285248 | KP285246 | KP285249 | KP285244 | KP285243 | KP285242 |
| A/chicken/Jiangxi/23797/2013 | H9N2 | Avian |  |  | KP285256 | KP285254 | KP285257 | KP285252 | KP285251 | KP285250 |
| A/chicken/Ningbo/2727/2013 | H9N2 | Avian |  |  | KP415344 | KP415342 | KP415345 | KP415340 | KP415339 | KP415338 |
| A/chicken/Ningbo/2929/2013 | H9N2 | Avian |  |  | KP415352 | KP415350 | KP415353 | KP415348 | KP415347 | KP415346 |
| A/chicken/Yantai/2243/2013 | H9N2 | Avian |  |  | KP415182 | KP415180 | KP415183 | KP415178 | KP415177 | KP415176 |
| A/chicken/Yantai/2303/2013 | H9N2 | Avian |  |  | KP415190 | KP415188 | KP415191 | KP415186 | KP415185 | KP415184 |
| A/silkie_chicken/Shaoxing/2471/2013 | H9N2 | Avian |  |  | KP415316 | KP415314 | KP415317 | KP415312 | KP415311 | KP415310 |
| A/chicken/Israel/1067/2010 | H9N2 | Avian |  |  | JQ254967 | JQ254944 | JQ254968 | JQ254969 | JQ254970 | JQ254971 |
| A/baikal_teal/Xianghai/421/2011 | H9N2 | Avian |  |  | KC162239 | KC162241 | KC162240 | KC162236 | KC162237 | KC162238 |
| A/chicken/Guangdong/SIC31/2014 | H9N2 | Avian |  |  | KX598682 | KX598640 | KX598724 | KX598598 | KX598556 | KX598514 |
| A/chicken/Zhejiang/SIC32/2014 | H9N2 | Avian |  |  | KX598699 | KX598657 | KX598741 | KX598615 | KX598573 | KX598531 |
| A/chicken/Bangladesh/18301/2012 | H9N2 | Avian |  |  | KJ643758 | KJ643756 | KJ643759 | KJ643754 | KJ643753 | KJ643752 |
| A/chicken/Shandong/903/2013 | H9N2 | Avian |  |  | KX900509 | KX900507 | KX900510 | KX900505 | KX900504 | KX900503 |
| A/chicken/Yantai/2468/2013 | H9N2 | Avian |  |  | KP414705 | KP414703 | KP414706 | KP414701 | KP414700 | KP414699 |
| A/chicken/Yantai/2469/2013 | H9N2 | Avian |  |  | KP414713 | KP414711 | KP414714 | KP414709 | KP414708 | KP414707 |
| A/chicken/Yantai/2484/2013 | H9N2 | Avian |  |  | KP415198 | KP415196 | KP415199 | KP415194 | KP415193 | KP415192 |
| A/chicken/Qingdao/2044/2013 | H9N2 | Avian |  |  | KP415166 | KP415164 | KP415167 | KP415162 | KP415161 | KP415160 |
| A/chicken/Qingdao/2144/2013 | H9N2 | Avian |  |  | KP415174 | KP415172 | KP415175 | KP415170 | KP415169 | KP415168 |
| A/chicken/Wenzhou/3293/2013 | H9N2 | Avian |  |  | KP415369 | KP415367 | KP415370 | KP415365 | KP415364 | KP415363 |
| A/chicken/Wenzhou/3330/2013 | H9N2 | Avian |  |  | KP415377 | KP415375 | KP415378 | KP415373 | KP415372 | KP415371 |
| A/northern_shoveler/California/6535/2009 | H9N2 | Avian |  |  | CY157503 | CY157505 | CY157506 | CY157507 | CY157508 | CY157509 |
| A/chicken/Huzhou/3801/2013 | H9N2 | Avian |  |  | KP415385 | KP415383 | KP415386 | KP415381 | KP415380 | KP415379 |
| A/chicken/Huzhou/3861/2013 | H9N2 | Avian |  |  | KP415393 | KP415391 | KP415394 | KP415389 | KP415388 | KP415387 |
| A/chicken/Huzhou/3892/2013 | H9N2 | Avian |  |  | KP417130 | KP417128 | KP417131 | KP417126 | KP417125 | KP417124 |
| A/chicken/Rizhao/2658/2013 | H9N2 | Avian |  |  | KP415222 | KP415220 | KP415223 | KP415218 | KP415217 | KP415216 |
| A/chicken/Rizhao/2670/2013 | H9N2 | Avian |  |  | KP415230 | KP415228 | KP415231 | KP415226 | KP415225 | KP415224 |
| A/chicken/Rizhao/3052/2013 | H9N2 | Avian |  |  | KP414793 | KP414791 | KP414794 | KP414789 | KP414788 | KP414787 |
| A/chicken/Zhejiang/W41/2013 | H9N2 | Avian |  |  | KU042450 | KU042344 | KU042503 | KU042238 | KU042185 | KU042132 |
| A/silkie_chicken/Huzhou/4218/2013 | H9N2 | Avian |  |  | KP417138 | KP417136 | KP417139 | KP417134 | KP417133 | KP417132 |
| A/chicken/Jiaxing/4321/2013 | H9N2 | Avian |  |  | KP414721 | KP414719 | KP414722 | KP414717 | KP414716 | KP414715 |
| A/chicken/Jiaxing/4376/2013 | H9N2 | Avian |  |  | KP417146 | KP417144 | KP417147 | KP417142 | KP417141 | KP417140 |
| A/chicken/Jiaxing/4380/2013 | H9N2 | Avian |  |  | KP414729 | KP414727 | KP414730 | KP414725 | KP414724 | KP414723 |
| A/chicken/Juxian/3387/2013 | H9N2 | Avian |  |  | KP415247 | KP415245 | KP415248 | KP415243 | KP415242 | KP415241 |
| A/chicken/Juxian/3412/2013 | H9N2 | Avian |  |  | KP415255 | KP415253 | KP415256 | KP415251 | KP415250 | KP415249 |
| A/chicken/Jiaxing/4713/2013 | H9N2 | Avian |  |  | KP417162 | KP417160 | KP417163 | KP417158 | KP417157 | KP417156 |
| A/chicken/Jiaxing/4725/2013 | H9N2 | Avian |  |  | KP417170 | KP417168 | KP417171 | KP417166 | KP417165 | KP417164 |
| A/chicken/Jiaxing/4742/2013 | H9N2 | Avian |  |  | KP414801 | KP414799 | KP414802 | KP414797 | KP414796 | KP414795 |
| A/chicken/Suzhou/4822/2013 | H9N2 | Avian |  |  | KP414745 | KP414743 | KP414746 | KP414741 | KP414740 | KP414739 |
| A/chicken/Suzhou/4837/2013 | H9N2 | Avian |  |  | KP414753 | KP414751 | KP414754 | KP414749 | KP414748 | KP414747 |
| A/chicken/Suzhou/4954/2013 | H9N2 | Avian |  |  | KP414761 | KP414759 | KP414762 | KP414757 | KP414756 | KP414755 |
| A/chicken/Suzhou/4960/2013 | H9N2 | Avian |  |  | KP414777 | KP414775 | KP414778 | KP414773 | KP414772 | KP414771 |
| A/chicken/Suzhou/4965/2013 | H9N2 | Avian |  |  | KP414785 | KP414783 | KP414786 | KP414781 | KP414780 | KP414779 |
| A/chicken/Shaoxing/5088/2013 | H9N2 | Avian |  |  | KP417197 | KP417195 | KP417198 | KP417193 | KP417192 | KP417191 |
| A/northern_pintail/California/2789/2011 | H9N2 | Avian |  |  | CY157519 | CY157521 | CY157522 | CY157523 | CY157524 | CY157525 |
| A/chicken/Jinan/3925/2013 | H9N2 | Avian |  |  | KP415263 | KP415261 | KP415264 | KP415259 | KP415258 | KP415257 |
| A/chicken/Jinan/3952/2013 | H9N2 | Avian |  |  | KP415271 | KP415269 | KP415272 | KP415267 | KP415266 | KP415265 |
| A/chicken/Jinan/4225/2013 | H9N2 | Avian |  |  | KP415279 | KP415277 | KP415280 | KP415275 | KP415274 | KP415273 |
| A/chicken/Jinan/4261/2013 | H9N2 | Avian |  |  | KP415287 | KP415285 | KP415288 | KP415283 | KP415282 | KP415281 |
| A/chicken/Shaoxing/5453/2013 | H9N2 | Avian |  |  | KP417224 | KP417222 | KP417225 | KP417220 | KP417219 | KP417218 |
| A/chicken/Shaoxing/5493/2013 | H9N2 | Avian |  |  | KP417232 | KP417230 | KP417233 | KP417228 | KP417227 | KP417226 |
| A/chicken/Shaoxing/5498/2013 | H9N2 | Avian |  |  | KP414737 | KP414735 | KP414738 | KP414733 | KP414732 | KP414731 |
| A/silkie_chicken/Shaoxing/5371/2013 | H9N2 | Avian |  |  | KP417216 | KP417214 | KP417217 | KP417212 | KP417211 | KP417210 |
| A/ostrich/Eshkol/1436/2003 | H9N2 | Avian |  |  | EF492261 |  | DQ683040 | EF492363 | EF492392 | EF492421 |
| A/mallard/Sweden/4932/2004 | H9N2 | Avian |  |  | CY184118 | CY184120 | CY184121 | CY184122 | CY184123 | CY184124 |
| A/chicken/Jilin/1031/2012 | H9N2 | Avian |  |  | KM609629 | KM609709 | KM609749 | KM609789 | KM609829 | KM609869 |
| A/mallard/Sweden/67860/2007 | H9N2 | Avian |  |  | CY184150 | CY184152 | CY184153 | CY184154 | CY184155 | CY184156 |
| A/chicken/Beijing/HD/2010 | H9N2 | Avian |  |  | KC821194 | KC821157 | KC821268 | KC821124 | KC821080 | KC821044 |
| A/chicken/Hebei/ZR/2010 | H9N2 | Avian |  |  | KC821193 | KC821156 | KC821267 | KC821123 | KC821084 | KC821045 |
| A/chicken/Shandong/11/2010 | H9N2 | Avian |  |  | KC821192 | KC821155 | KC821266 | KC821122 | KC821079 | KC821041 |
| A/chicken/Shandong/11/2011 | H9N2 | Avian |  |  | KC821201 | KC821167 | KC821281 | KC821136 | KC821097 | KC821051 |
| A/wild_waterfowl/Dongting/2123/2011 | H9N2 | Avian |  |  | KF971960 | KF971958 | KF971961 | KF971956 | KF971955 | KF971954 |
| A/wild_waterfowl/Dongting/C2032/2011 | H9N2 | Avian |  |  | KF971952 | KF971950 | KF971953 | KF971948 | KF971947 | KF971946 |
| A/wild_waterfowl/Dongting/C2148/2011 | H9N2 | Avian |  |  | KF971968 | KF971966 | KF971969 | KF971964 | KF971963 | KF971962 |
| A/wild_waterfowl/Dongting/C2149/2011 | H9N2 | Avian |  |  | KF971976 | KF971974 | KF971977 | KF971972 | KF971971 | KF971970 |
| A/wild_waterfowl/Dongting/C2150/2011 | H9N2 | Avian |  |  | KF971984 | KF971982 | KF971985 | KF971980 | KF971979 | KF971978 |
| A/wild_waterfowl/Dongting/C2203/2011 | H9N2 | Avian |  |  | KF971992 | KF971990 | KF971993 | KF971988 | KF971987 | KF971986 |
| A/wild_waterfowl/Dongting/C3109_/2011 | H9N2 | Avian |  |  | KF972000 | KF971998 | KF972001 | KF971996 | KF971995 | KF971994 |
| A/chicken/Israel/869/2007 | H9N2 | Avian |  |  | GQ148830 | GQ148844 | GQ148858 | GQ140287 | GQ148872 | GQ140273 |
| A/duck/Shanghai/C163/2009 | H9N2 | Avian |  |  | KC768043 | KC768049 | KC768052 | KC768055 | KC768058 | KC768061 |
| A/duck/Shanghai/C164/2009 | H9N2 | Avian |  |  | KC768044 | KC768050 | KC768053 | KC768056 | KC768059 | KC768062 |
| A/chicken/Jiangsu/XZ6/2012 | H9N2 | avian |  |  | EPI_ISL_145386 | EPI_ISL_145386 | EPI_ISL_145386 | EPI_ISL_145386 | EPI_ISL_145386 | EPI_ISL_145386 |
| A/brambling/Beijing/16/2012 | H9N2 | Avian |  |  | KC464601 | KC464599 | KC464602 | KC464597 | KC464596 | KC464595 |
| A/chicken/Jiangxi/28563/2013 | H9N2 | Avian |  |  | KP285416 | KP285414 | KP285417 | KP285412 | KP285411 | KP285410 |
| A/chicken/Jiangxi/28601/2013 | H9N2 | Avian |  |  | KP285272 | KP285270 | KP285273 | KP285268 | KP285267 | KP285266 |
| A/duck/Hunan/S4111/2011 | H9N2 | Avian |  |  | CY146663 | CY146661 | CY146664 | CY146659 | CY146658 | CY146657 |
| A/chicken/Zhejiang/JX42/2012 | H9N2 | Avian |  |  | KJ426406 | KJ426428 | KJ426439 | KJ426450 | KJ426461 | KJ426472 |
| A/chicken/China/Guangxi14/2000 | H9N2 | Avian |  |  | DQ485219 | DQ485217 | DQ485220 | DQ485215 | DQ485214 | DQ485213 |
| A/chicken/Zhejiang/3C19/2013 | H9N2 | Avian |  |  | KU042453 | KU042347 | KU042506 | KU042241 | KU042188 | KU042135 |
| A/chicken/Zhejiang/3C2/2013 | H9N2 | Avian |  |  | KU042451 | KU042345 | KU042504 | KU042239 | KU042186 | KU042133 |
| A/chicken/Zhejiang/3C22/2013 | H9N2 | Avian |  |  | KU042454 | KU042348 | KU042507 | KU042242 | KU042189 | KU042136 |
| A/chicken/Zhejiang/3C31/2013 | H9N2 | Avian |  |  | KU042455 | KU042349 | KU042508 | KU042243 | KU042190 | KU042137 |
| A/chicken/Zhejiang/3C7/2013 | H9N2 | Avian |  |  | KU042452 | KU042346 | KU042505 | KU042240 | KU042187 | KU042134 |
| A/chicken/Guizhou/C4058/2011 | H9N2 | Avian |  |  | KM113136 | KM113134 | KM113133 | KM113132 | KM113131 | KM113130 |
| A/chicken/Yunnan/C4090/2011 | H9N2 | Avian |  |  | KM113112 | KM113110 | KM113109 | KM113108 | KM113107 | KM113106 |
| A/chicken/Bangladesh/18408/2012 | H9N2 | Avian |  |  | KJ643787 | KJ643785 | KJ643788 | KJ643783 | KJ643782 | KJ643781 |
| A/chicken/Shandong/qd1115/2012 | H9N2 | Avian |  |  | KM609610 | KM609690 | KM609730 | KM609770 | KM609810 | KM609850 |
| A/chicken/Beijing/1115/2013 | H9N2 | Avian |  |  | KM609635 | KM609715 | KM609755 | KM609795 | KM609835 | KM609875 |
| A/chicken/Liaoning/1116/2012 | H9N2 | Avian |  |  | KM609626 | KM609706 | KM609746 | KM609786 | KM609826 | KM609866 |
| A/bewicks_swan/Netherlands/4/2006 | H9N2 | Avian |  |  | KX978350 | KX978585 | KX977751 | KX977955 | KX979704 | KX979384 |
| A/chicken/Shaanxi/11/2012 | H9N2 | Avian |  |  | KC767262 | KC767260 | KC767263 | KC767258 | KC767257 | KC767264 |
| A/chicken/Jiangxi/29075/2013 | H9N2 | Avian |  |  | KP285280 | KP285278 | KP285281 | KP285276 | KP285275 | KP285274 |
| A/chicken/Jiangxi/29086/2013 | H9N2 | Avian |  |  | KP285288 | KP285286 | KP285289 | KP285284 | KP285283 | KP285282 |
| A/chicken/Jiangxi/29099/2013 | H9N2 | Avian |  |  | KP285296 | KP285294 | KP285297 | KP285292 | KP285291 | KP285290 |
| A/chicken/Jiangxi/29117/2013 | H9N2 | Avian |  |  | KP285304 | KP285302 | KP285305 | KP285300 | KP285299 | KP285298 |
| A/mallard/Sweden/7146/2004 | H9N2 | Avian |  |  | CY184126 | CY184128 | CY184129 | CY184130 | CY184131 | CY184132 |
| A/avian/Bangladesh/91254/2012 | H9N2 | avian |  |  | EPI_ISL_165820 | EPI_ISL_165820 | EPI_ISL_165820 | EPI_ISL_165820 | EPI_ISL_165820 | EPI_ISL_165820 |
| A/avian/Bangladesh/91256/2012 | H9N2 | avian |  |  | EPI_ISL_165818 | EPI_ISL_165818 | EPI_ISL_165818 | EPI_ISL_165818 | EPI_ISL_165818 | EPI_ISL_165818 |
| A/chicken/China/Guangxi17/2000 | H9N2 | Avian |  |  | DQ485227 | DQ485225 | DQ485228 | DQ485223 | DQ485222 | DQ485221 |
| A/chicken/Guangxi/LF/2007 | H9N2 | Avian |  |  | KF768253 | KF768249 | KF768247 | KF768250 | KF768246 | KF768248 |
| A/wild_bird/Korea/8G39/2005 | H9N2 | Avian |  |  | HQ143706 | HQ143738 | KC209499 | KC209498 | KC209495 | KC209496 |
| A/chicken/Hebei/A/2007 | H9N2 | Avian |  |  | GQ202059 | GQ202062 | GQ202058 | GQ202063 | GQ202060 | GQ202061 |
| A/African_Stonechat/Vietnam/8/2009 | H9N2 | Avian |  |  | AB753221 | AB753218 | AB753220 | AB753216 | AB753215 | AB753214 |
| A/chicken/Guangdong/G7/2009 | H9N2 | Avian |  |  | KF367734 | KF367732 | KF367735 | KF367731 | KF367730 | KF367729 |
| A/chicken/Guangxi/G8/2009 | H9N2 | Avian |  |  | KF367741 | KF367739 | KF367742 | KF367738 | KF367737 | KF367736 |
| A/Japanese_Quail/Vietnam/4/2009 | H9N2 | Avian |  |  | AB753195 | AB753193 | AB753196 | AB753197 | AB753191 | AB753190 |
| A/Japanese_Quail/Vietnam/7/2009 | H9N2 | Avian |  |  | AB753211 | AB753209 | AB753212 | AB753208 | AB753207 | AB753206 |
| A/Rock_Pigeon/Vietnam/6/2009 | H9N2 | Avian |  |  | AB753204 | AB753202 | AB753205 | AB753200 | AB753199 | AB753198 |
| A/chicken/Israel/1167/2010 | H9N2 | Avian |  |  | JQ254972 | JQ254947 | JQ254973 | JQ254974 | JQ254975 | JQ254976 |
| A/chicken/Israel/1184/2010 | H9N2 | Avian |  |  | JQ254977 | JQ254950 | JQ254978 | JQ254979 | JQ254980 | JQ254981 |
| A/chicken/Guangdong/SIC2/2012 | H9N2 | Avian |  |  | KX598697 | KX598655 | KX598739 | KX598613 | KX598571 | KX598529 |
| A/chicken/Shanghai/1107/2013 | H9N2 | Avian |  |  | KJ726699 | KJ726711 | KJ726717 | KJ726723 | KJ726729 | KJ726735 |
| A/chicken/Hainan/SIC33/2014 | H9N2 | Avian |  |  | KX598722 | KX598680 | KX598764 | KX598638 | KX598596 | KX598554 |
| A/chicken/Shandong/SIC34/2014 | H9N2 | Avian |  |  | KX598712 | KX598670 | KX598754 | KX598628 | KX598586 | KX598544 |
| A/chicken/Jiangxi/25970/2013 | H9N2 | Avian |  |  | KP285264 | KP285262 | KP285265 | KP285260 | KP285259 | KP285258 |
| A/chicken/Guangxi/LS/2013 | H9N2 | Avian |  |  | KM070513 | KM070511 | KM070514 | KM070509 | KM070508 | KM070507 |
| A/chicken/Korea/GH2/2007 | H9N2 | Avian |  |  | HQ871934 | HQ871937 | HQ871936 | HQ871938 | HQ871940 | HQ871939 |
| A/quail/Bangladesh/18521/2012 | H9N2 | Avian |  |  | KJ643646 | KJ643644 | KJ643647 | KJ643642 | KJ643641 | KJ643640 |
| A/chicken/Zhejiang/4C105/2013 | H9N2 | Avian |  |  | KU042457 | KU042351 | KU042510 | KU042245 | KU042192 | KU042139 |
| A/chicken/Zhejiang/4C91/2013 | H9N2 | Avian |  |  | KU042456 | KU042350 | KU042509 | KU042244 | KU042191 | KU042138 |
| A/chicken/Dubai/D2506.A/2015 | H9N2 | Avian |  |  | KX351201 | KX351199 | KX351202 | KX351197 | KX351196 | KX351195 |
| A/chicken/Israel/953/2007 | H9N2 | Avian |  |  | GQ148833 | GQ148847 | GQ148861 | GQ140290 | GQ148875 | GQ140276 |
| A/avian/Bangladesh/91286/2012 | H9N2 | avian |  |  | EPI_ISL_165821 | EPI_ISL_165821 | EPI_ISL_165821 | EPI_ISL_165821 | EPI_ISL_165821 | EPI_ISL_165821 |
| A/chicken/Henan/12/2008 | H9N2 | Avian |  |  | KJ426501 | KJ426525 | KJ426285 | KJ426295 | KJ426307 | KJ426319 |
| A/chicken/Shandong/LY/2008 | H9N2 | Avian |  |  | KC821181 | KC821143 | KC821255 | KC821107 | KC821069 | KC821032 |
| A/chicken/Shandong/zc12/2009 | H9N2 | Avian |  |  | KC821185 | KC821148 | KC821259 | KC821111 | KC821074 | KC821037 |
| A/chicken/Hebei/YT/2010 | H9N2 | Avian |  |  | KC821195 | KC821160 | KC821269 | KC821125 | KC821086 | KC821049 |
| A/chicken/Jiangsu/TS/2010 | H9N2 | Avian |  |  | KC821197 | KC821159 | KC821271 | KC821127 | KC821076 | KC821043 |
| A/chicken/Shandong/B1/2010 | H9N2 | Avian |  |  | KJ426414 | KJ426436 | KJ426447 | KJ426458 | KJ426469 | KJ426480 |
| A/chicken/Shandong/HL/2010 | H9N2 | Avian |  |  | KC821196 | KC821158 | KC821270 | KC821126 | KC821078 | KC821040 |
| A/chicken/Hebei/FL/2011 | H9N2 | Avian |  |  | KC821206 | KC821169 | KC821285 | KC821137 | KC821100 | KC821063 |
| A/chicken/Jiangxi/30772/2013 | H9N2 | Avian |  |  | KP285424 | KP285422 | KP285425 | KP285420 | KP285419 | KP285418 |
| A/chicken/Shanghai/5/2003 | H9N2 | Avian |  |  | KP866015 | KP865781 | KP866059 | KP865815 | KP865871 | KP865918 |
| A/chicken/Shanghai/2/2004 | H9N2 | Avian |  |  | KP866017 | KP865779 | KP866061 | KP865813 | KP865869 | KP865916 |
| A/chicken/Shanghai/4/2006 | H9N2 | Avian |  |  | KP866019 | KP865780 | KP866063 | KP865814 | KP865870 | KP865917 |
| A/chicken/Israel/951/2007 | H9N2 | Avian |  |  | GQ148832 | GQ148846 | GQ148860 | GQ140289 | GQ148874 | GQ140275 |
| A/chicken/Guangdong/1494/2009 | H9N2 | Avian |  |  | KP866030 | KP865755 | KP866072 | KP865823 | KP865858 | KP865905 |
| A/chicken/Guangdong/1683/2009 | H9N2 | Avian |  |  | KP866031 | KP865756 | KP866073 | KP865824 | KP865859 | KP865906 |
| A/chicken/Hunan/2351/2009 | H9N2 | Avian |  |  | KP866035 | KP865757 | KP866077 | KP865825 | KP865862 | KP865909 |
| A/chicken/Jiangsu/925/2009 | H9N2 | Avian |  |  | KP866027 | KP865758 | KP866070 | KP865826 | KP865864 | KP865911 |
| A/chicken/Shandong/2552/2009 | H9N2 | Avian |  |  | KP866037 | KP865760 | KP866079 | KP865828 | KP865866 | KP865913 |
| A/chicken/Shandong/2567/2009 | H9N2 | Avian |  |  | KP866038 | KP865761 | KP866080 | KP865829 | KP865867 | KP865914 |
| A/chicken/Shanghai/169/2009 | H9N2 | Avian |  |  | KP866022 | KP865763 | KP866066 | KP865831 | KP865875 | KP865922 |
| A/chicken/Shanghai/340/2009 | H9N2 | Avian |  |  | KP866023 | KP865784 | KP866067 | KP865818 | KP865876 | KP865923 |
| A/chicken/Shanghai/441/2009 | H9N2 | Avian |  |  | KP866024 | KP865785 | KP866068 | KP865819 | KP865877 | KP865924 |
| A/chicken/Shanghai/50/2009 | H9N2 | Avian |  |  | KP866020 | KP865762 | KP866064 | KP865830 | KP865873 | KP865920 |
| A/chicken/Shanghai/510/2009 | H9N2 | Avian |  |  | KP866025 | KP865786 | KP866069 | KP865820 | KP865878 | KP865925 |
| A/Duck/Fujian/1753/2009 | H9N2 | Avian |  |  | KP866032 | KP865764 | KP866074 | KP865832 | KP865879 | KP865926 |
| A/chicken/China/AH-10-01/2010 | H9N2 | Avian |  |  | JF906209 | JF906207 | JF906210 | JF906205 | JF906204 | JF906203 |
| A/chicken/Guangdong/C76/2012 | H9N2 | Avian |  |  | KP866053 | KP865770 | KP866095 | KP865792 | KP865838 | KP865885 |
| A/chicken/Jiangsu/C1243/2012 | H9N2 | Avian |  |  | KP866057 | KP865765 | KP866099 | KP865787 | KP865833 | KP865880 |
| A/chicken/Zhejiang/B2013/2012 | H9N2 | Avian |  |  | KP866054 | KP865771 | KP866096 | KP865793 | KP865839 | KP865886 |
| A/duck/Anhui/C313/2012 | H9N2 | Avian |  |  | KP866052 | KP865769 | KP866094 | KP865791 | KP865837 | KP865884 |
| A/duck/Anhui/C330/2012 | H9N2 | Avian |  |  | KP866055 | KP865768 | KP866097 | KP865790 | KP865836 | KP865883 |
| A/duck/Anhui/C652/2012 | H9N2 | Avian |  |  | KP866056 | KP865767 | KP866098 | KP865789 | KP865835 | KP865882 |
| A/chicken/Attock/NARC-14994/2009 | H9N2 | Avian |  |  | JN540077 | JN540078 | JN540076 | JN540079 | JN540080 | JN540081 |
| A/chicken/Beijing/B7/2010 | H9N2 | Avian |  |  | KJ426415 | KJ426437 | KJ426448 | KJ426459 | KJ426470 | KJ426481 |
| A/chicken/Hunan/12/2011 | H9N2 | Avian |  |  | KF714786 | KF714784 | KF714787 | KF714782 | KF714781 | KF714780 |
| A/northern_shoveler/Missouri/298/2009 | H9N2 | Avian |  |  | CY097631 | CY097633 | CY097634 | CY097635 | CY097636 | CY097637 |
| A/chicken/Shandong/wf1206/2012 | H9N2 | Avian |  |  | KM609604 | KM609684 | KM609724 | KM609764 | KM609804 | KM609844 |
| A/chicken/Hebei/B5/2010 | H9N2 | Avian |  |  | KJ426416 | KJ426438 | KJ426449 | KJ426460 | KJ426471 | KJ426482 |
| A/northern_pintail/California/3100/2011 | H9N2 | Avian |  |  | CY157527 | CY157529 | CY157530 | CY157531 | CY157532 | CY157533 |
| A/chicken/Jiangxi/31872/2013 | H9N2 | Avian |  |  | KP285440 | KP285438 | KP285441 | KP285436 | KP285435 | KP285434 |
| A/chicken/Shenzhen/844/2013 | H9N2 | Avian |  |  | KP414289 | KP414287 | KP414290 | KP414285 | KP414284 | KP414283 |
| A/chicken/Shenzhen/881/2013 | H9N2 | Avian |  |  | KP414297 | KP414295 | KP414298 | KP414293 | KP414292 | KP414291 |
| A/pigeon/Egypt/S10408B/2014 | H9N2 | Avian |  |  | KX000779 | KX000866 | KX000859 | KX000855 | KX000837 | KX000876 |
| A/pigeon/Egypt/S10409A/2014 | H9N2 | Avian |  |  | KX000828 | KX000806 | KX000785 | KX000723 | KX000729 | KX000764 |
| A/chicken/Shandong/wf12010/2012 | H9N2 | Avian |  |  | KM609605 | KM609685 | KM609725 | KM609765 | KM609805 | KM609845 |
| A/chicken/Shenzhen/317/2013 | H9N2 | Avian |  |  | KP414230 | KP414228 | KP414231 | KP414226 | KP414225 | KP414224 |
| A/chicken/Shenzhen/354/2013 | H9N2 | Avian |  |  | KP414238 | KP414236 | KP414239 | KP414234 | KP414233 | KP414232 |
| A/chicken/Shenzhen/515/2013 | H9N2 | Avian |  |  | KP414263 | KP414261 | KP414264 | KP414259 | KP414258 | KP414257 |
| A/chicken/Shenzhen/678/2013 | H9N2 | Avian |  |  | KP414272 | KP414270 | KP414273 | KP414268 | KP414267 | KP414266 |
| A/mallard/Arkansas/AI09-5649/2009 | H9N2 | Avian |  |  | CY146514 | CY146516 | CY146517 | CY146518 | CY146519 | CY146520 |
| A/chicken/Shenzhen/1047/2013 | H9N2 | Avian |  |  | KP414334 | KP414332 | KP414335 | KP414330 | KP414329 | KP414328 |
| A/chicken/Shenzhen/1098/2013 | H9N2 | Avian |  |  | KP414342 | KP414340 | KP414343 | KP414338 | KP414337 | KP414336 |
| A/chicken/Shenzhen/1377/2013 | H9N2 | Avian |  |  | KP414368 | KP414366 | KP414369 | KP414364 | KP414363 | KP414362 |
| A/chicken/Shenzhen/1426/2013 | H9N2 | Avian |  |  | KP414376 | KP414374 | KP414377 | KP414372 | KP414371 | KP414370 |
| A/chicken/Shenzhen/1488/2013 | H9N2 | Avian |  |  | KP414384 | KP414382 | KP414385 | KP414380 | KP414379 | KP414378 |
| A/chicken/Guangxi/DX/2008 | H9N2 | Avian |  |  | KF768194 | KF768197 | KF768193 | KF768198 | KF768191 | KF768196 |
| A/mallard/Missouri/10OS4670/2010 | H9N2 | Avian |  |  | CY133374 | CY133376 | CY133377 | CY133378 | CY133379 | CY133380 |
| A/chicken/Shenzhen/1519/2013 | H9N2 | Avian |  |  | KP414246 | KP414244 | KP414247 | KP414242 | KP414241 | KP414240 |
| A/chicken/Shenzhen/1591/2013 | H9N2 | Avian |  |  | KP414400 | KP414398 | KP414401 | KP414396 | KP414395 | KP414394 |
| A/chicken/Shenzhen/1657/2013 | H9N2 | Avian |  |  | KP414416 | KP414414 | KP414417 | KP414412 | KP414411 | KP414410 |
| A/chicken/Shenzhen/1737/2013 | H9N2 | Avian |  |  | KP414424 | KP414422 | KP414425 | KP414420 | KP414419 | KP414418 |
| A/chicken/Shenzhen/1770/2013 | H9N2 | Avian |  |  | KP414544 | KP414542 | KP414545 | KP414540 | KP414539 | KP414538 |
| A/chicken/Shenzhen/1799/2013 | H9N2 | Avian |  |  | KP414432 | KP414430 | KP414433 | KP414428 | KP414427 | KP414426 |
| A/chicken/Shenzhen/1882/2013 | H9N2 | Avian |  |  | KP414440 | KP414438 | KP414441 | KP414436 | KP414435 | KP414434 |
| A/chicken/Dongguan/2309/2013 | H9N2 | Avian |  |  | KP414480 | KP414478 | KP414481 | KP414476 | KP414475 | KP414474 |
| A/chicken/Shenzhen/2014/2013 | H9N2 | Avian |  |  | KP414456 | KP414454 | KP414457 | KP414452 | KP414451 | KP414450 |
| A/chicken/Shenzhen/2066/2013 | H9N2 | Avian |  |  | KP414464 | KP414462 | KP414465 | KP414460 | KP414459 | KP414458 |
| A/chicken/Shenzhen/2121/2013 | H9N2 | Avian |  |  | KP414830 | KP414828 | KP414831 | KP414826 | KP414825 | KP414824 |
| A/chicken/Shenzhen/2130/2013 | H9N2 | Avian |  |  | KP417557 | KP417555 | KP417558 | KP417553 | KP417552 | KP417551 |
| A/chicken/Shenzhen/2403/2013 | H9N2 | Avian |  |  | KP414488 | KP414486 | KP414489 | KP414484 | KP414483 | KP414482 |
| A/chicken/Shenzhen/2477/2013 | H9N2 | Avian |  |  | KP414496 | KP414494 | KP414497 | KP414492 | KP414491 | KP414490 |
| A/chicken/Shenzhen/2588/2013 | H9N2 | Avian |  |  | KP414504 | KP414502 | KP414505 | KP414500 | KP414499 | KP414498 |
| A/chicken/Shenzhen/2636/2013 | H9N2 | Avian |  |  | KP414512 | KP414510 | KP414513 | KP414508 | KP414507 | KP414506 |
| A/chicken/Egypt/Q10429E/2014 | H9N2 | Avian |  |  | KX000714 | KX000744 | KX000849 | KX000874 | KX000877 | KX000752 |
| A/white-fronted_goose/Korea/20-36/2007 | H9N2 | Avian |  |  | KC693639 | KC693646 | KC693644 | KC693643 | KC693640 | KC693641 |
| A/poultry/Bangladesh/91311/2012 | H9N2 | avian |  |  | EPI_ISL_165816 | EPI_ISL_165816 | EPI_ISL_165816 | EPI_ISL_165816 | EPI_ISL_165816 | EPI_ISL_165816 |
| A/poultry/Bangladesh/91349/2012 | H9N2 | avian |  |  | EPI_ISL_165817 | EPI_ISL_165817 | EPI_ISL_165817 | EPI_ISL_165817 | EPI_ISL_165817 | EPI_ISL_165817 |
| A/poultry/Bangladesh/91354/2012 | H9N2 | avian |  |  | EPI_ISL_165822 | EPI_ISL_165822 | EPI_ISL_165822 | EPI_ISL_165822 | EPI_ISL_165822 | EPI_ISL_165822 |
| A/chicken/Jiangxi/33542/2013 | H9N2 | Avian |  |  | KP285448 | KP285446 | KP285449 | KP285444 | KP285443 | KP285442 |
| A/chicken/Jiangxi/33552/2013 | H9N2 | Avian |  |  | KP416665 | KP416663 | KP416666 | KP416661 | KP416660 | KP416659 |
| A/chicken/Pakistan/UDL-03/2007 | H9N2 | Avian |  |  | CY038453 | CY038451 | CY038454 | CY038449 | CY038448 | CY038447 |
| A/chicken/Dongguan/2701/2013 | H9N2 | Avian |  |  | KP414520 | KP414518 | KP414521 | KP414516 | KP414515 | KP414514 |
| A/chicken/Dongguan/2754/2013 | H9N2 | Avian |  |  | KP414528 | KP414526 | KP414529 | KP414524 | KP414523 | KP414522 |
| A/chicken/Dongguan/2827/2013 | H9N2 | Avian |  |  | KP414536 | KP414534 | KP414537 | KP414532 | KP414531 | KP414530 |
| A/chicken/Dongguan/2900/2013 | H9N2 | Avian |  |  | KP414552 | KP414550 | KP414553 | KP414548 | KP414547 | KP414546 |
| A/chicken/Dongguan/3056/2013 | H9N2 | Avian |  |  | KP414568 | KP414566 | KP414569 | KP414564 | KP414563 | KP414562 |
| A/chicken/Dongguan/3186/2013 | H9N2 | Avian |  |  | KP414584 | KP414582 | KP414585 | KP414580 | KP414579 | KP414578 |
| A/chicken/Dongguan/3250/2013 | H9N2 | Avian |  |  | KP414697 | KP414695 | KP414698 | KP414693 | KP414692 | KP414691 |
| A/chicken/Dongguan/3315/2013 | H9N2 | Avian |  |  | KP414592 | KP414590 | KP414593 | KP414588 | KP414587 | KP414586 |
| A/Anser_fabalis/Anhui/L139/2014 | H9N2 | Avian |  |  | KT699059 | KT699057 | KT699060 | KT699055 | KT699054 | KT699053 |
| A/chicken/Dongguan/3409/2013 | H9N2 | Avian |  |  | KP414608 | KP414606 | KP414609 | KP414604 | KP414603 | KP414602 |
| A/chicken/Dongguan/3550/2013 | H9N2 | Avian |  |  | KP414616 | KP414614 | KP414617 | KP414612 | KP414611 | KP414610 |
| A/chicken/Dongguan/3562/2013 | H9N2 | Avian |  |  | KP414624 | KP414622 | KP414625 | KP414620 | KP414619 | KP414618 |
| A/chicken/Dongguan/3839/2013 | H9N2 | Avian |  |  | KP414640 | KP414638 | KP414641 | KP414636 | KP414635 | KP414634 |
| A/chicken/Dongguan/3964/2013 | H9N2 | Avian |  |  | KP414648 | KP414646 | KP414649 | KP414644 | KP414643 | KP414642 |
| A/chicken/Dongguan/4189/2013 | H9N2 | Avian |  |  | KP414672 | KP414670 | KP414673 | KP414668 | KP414667 | KP414666 |
| A/chicken/Shenzhen/3745/2013 | H9N2 | Avian |  |  | KP414632 | KP414630 | KP414633 | KP414628 | KP414627 | KP414626 |
| A/silkie_chicken/Dongguan/4184/2013 | H9N2 | Avian |  |  | KP414664 | KP414662 | KP414665 | KP414660 | KP414659 | KP414658 |
| A/duck/ShanDong/JN1/2015 | H9N2 | Avian |  |  | KY212943 | KY212944 | KY212942 | KY212945 | KY212949 | KY212948 |
| A/Chinese_Hwamei/Vietnam/38/2006 | H9N2 | Avian |  |  | AB753180 | AB753178 | AB753181 | AB753176 | AB753175 | AB753174 |
| A/duck/Fujian/FQ107/2007 | H9N2 | Avian |  |  | HQ425337 | HQ425336 | HQ425338 | HQ425333 | HQ425332 | HQ425331 |
| A/chicken/Shandong/02/2008 | H9N2 | Avian |  |  | JF795049 | JF795047 | JF795050 | JF795045 | JF795044 | JF795043 |
| A/chicken/Shandong/BD/2008 | H9N2 | Avian |  |  | JF795081 | JF795079 | JF795082 | JF795077 | JF795076 | JF795075 |
| A/chicken/Shandong/LY-1/2008 | H9N2 | Avian |  |  | JF795113 | JF795111 | JF795114 | JF795109 | JF795108 | JF795107 |
| A/chicken/Fujian/G9/2009 | H9N2 | Avian |  |  | JN869519 | JN869517 | JN869520 | JN869516 | JN869515 | JN869514 |
| A/chicken/Israel/1293/2010 | H9N2 | Avian |  |  | JQ254982 | JQ254953 | JQ254983 | JQ254984 | JQ254985 | JQ254986 |
| A/chicken/Hubei/SIC3/2012 | H9N2 | Avian |  |  | KX598685 | KX598643 | KX598727 | KX598601 | KX598559 | KX598517 |
| A/chicken/Fujian/SIC13/2013 | H9N2 | Avian |  |  | KX598693 | KX598651 | KX598735 | KX598609 | KX598567 | KX598525 |
| A/chicken/Guangdong/SIC14/2013 | H9N2 | Avian |  |  | KX598683 | KX598641 | KX598725 | KX598599 | KX598557 | KX598515 |
| A/chicken/Guangxi/SIC15/2013 | H9N2 | Avian |  |  | KX598695 | KX598653 | KX598737 | KX598611 | KX598569 | KX598527 |
| A/chicken/Shandong/SIC35/2014 | H9N2 | Avian |  |  | KX598713 | KX598671 | KX598755 | KX598629 | KX598587 | KX598545 |
| A/chicken/Sichuan/SIC36/2014 | H9N2 | Avian |  |  | KX598716 | KX598674 | KX598758 | KX598632 | KX598590 | KX598548 |
| A/chicken/Wenzhou/YHQL04/2014 | H9N2 | Avian |  |  | KU143324 | KU143410 | KU143453 | KU143496 | KU143539 | KU143582 |
| A/duck/Wenzhou/YHQL64/2014 | H9N2 | Avian |  |  | KU143325 | KU143411 | KU143454 | KU143497 | KU143540 | KU143583 |
| A/duck/Wuhan/WHYF05/2014 | H9N2 | Avian |  |  | KU143329 | KU143414 | KU143456 | KU143500 | KU143543 | KU143586 |
| A/duck/Wuhan/WHYF14/2014 | H9N2 | Avian |  |  | KU143328 | KU143415 | KU143458 | KU143501 | KU143542 | KU143587 |
| A/chicken/Pakistan/UDL-03/2005 | H9N2 | Avian |  |  | CY038445 | CY038443 | CY038446 | CY038441 | CY038440 | CY038439 |
| A/chicken/Vietnam/NCVD-1156/2011 | H9N2 | avian |  |  | EPI_ISL_142872 | EPI_ISL_142872 | EPI_ISL_142872 | EPI_ISL_142872 | EPI_ISL_142872 | EPI_ISL_142872 |
| A/chicken/Israel/1040/2007 | H9N2 | Avian |  |  | GQ148820 | GQ148834 | GQ148848 | GQ140277 | GQ148862 | GQ140263 |
| A/chicken/Jiangxi/34682/2013 | H9N2 | Avian |  |  | KP285464 | KP285462 | KP285465 | KP285460 | KP285459 | KP285458 |
| A/chicken/Shandong/qd1224/2012 | H9N2 | Avian |  |  | KM609609 | KM609689 | KM609729 | KM609769 | KM609809 | KM609849 |
| A/duck/Shanghai/C60/2007 | H9N2 | Avian |  |  | KC768042 | KC768048 | KC768051 | KC768054 | KC768057 | KC768060 |
| A/chicken/Hunan/C3229/2012 | H9N2 | Avian |  |  | KM113152 | KM113150 | KM113149 | KM113148 | KM113147 | KM113146 |
| A/chicken/Jiangsu/C3226/2012 | H9N2 | Avian |  |  | KM113160 | KM113158 | KM113157 | KM113156 | KM113155 | KM113154 |
| A/chicken/Jiangsu/C4258/2012 | H9N2 | Avian |  |  | KM113088 | KM113086 | KM113085 | KM113084 | KM113083 | KM113082 |
| A/chicken/Jiangsu/WJ57/2012 | H9N2 | Avian |  |  | KP893707 | KP893705 | KP893708 | KJ000709 | KP893704 | KP893703 |
| A/chicken/Hubei/C4196/2009 | H9N2 | Avian |  |  | KM113096 | KM113094 | KM113093 | KM113092 | KM113091 | KM113090 |
| A/chicken/Egypt/S4454E/2011 | H9N2 | Avian |  |  | KF881650 | KF881648 | KF881651 | KF881647 | KF881646 | KF881645 |
| A/chicken/Egypt/S4456B/2011 | H9N2 | Avian |  |  | JX273134 | JX273139 | JX273138 | JX273137 | JX273135 | JX273136 |
| A/duck/Jiangxi/7554/2007 | H9N2 | Avian |  |  | KU366363 | KU366361 | KU366364 | KU366359 | KU366358 | KU366357 |
| A/chicken/Guangxi/C4080/2010 | H9N2 | Avian |  |  | KM113120 | KM113118 | KM113117 | KM113116 | KM113115 | KM113114 |
| A/chicken/Hubei/C4071/2010 | H9N2 | Avian |  |  | KM113128 | KM113126 | KM113125 | KM113124 | KM113123 | KM113122 |
| A/chicken/Hunan/C4136/2010 | H9N2 | Avian |  |  | KM113104 | KM113102 | KM113101 | KM113100 | KM113099 | KM113098 |
| A/chicken/Pakistan/UDL-02/2006 | H9N2 | Avian |  |  | CY038437 | CY038435 | CY038438 | CY038433 | CY038432 | CY038431 |
| A/chicken/Jiangxi/36277/2013 | H9N2 | Avian |  |  | KP416692 | KP416690 | KP416693 | KP416688 | KP416687 | KP416686 |
| A/duck/Jiangxi/36485/2013 | H9N2 | Avian |  |  | KP286240 | KP286238 | KP286241 | KP286236 | KP286235 | KP286234 |
| A/duck/Jiangxi/36487/2013 | H9N2 | Avian |  |  | KP286248 | KP286246 | KP286249 | KP286244 | KP286243 | KP286242 |
| A/duck/Jiangxi/36489/2013 | H9N2 | Avian |  |  | KP286256 | KP286254 | KP286257 | KP286252 | KP286251 | KP286250 |
| A/duck/Jiangxi/36501/2013 | H9N2 | Avian |  |  | KP286264 | KP286262 | KP286265 | KP286260 | KP286259 | KP286258 |
| A/duck/Jiangxi/36505/2013 | H9N2 | Avian |  |  | KP285064 | KP285062 | KP285065 | KP285060 | KP285059 | KP285058 |
| A/chicken/Pakistan/UDL-02/2005 | H9N2 | Avian |  |  | CY038421 | CY038419 | CY038422 | CY038417 | CY038416 | CY038415 |
| A/environment/Bangladesh/18849/2013 | H9N2 | environment |  |  | KJ643631 | KJ643629 | KJ643632 | KJ643627 | KJ643626 | KJ643625 |
| A/environment/Bangladesh/18894/2013 | H9N2 | environment |  |  | KJ643843 | KJ643841 | KJ643844 | KJ643839 | KJ643838 | KJ643837 |
| A/environment/Jilin/13206/2014 | H9N2 | environment |  |  | EPI_ISL_161676 | EPI_ISL_161676 | EPI_ISL_161676 | EPI_ISL_161676 | EPI_ISL_161676 | EPI_ISL_161676 |
| A/environment/Hunan/18556/2014 | H9N2 | environment |  |  | KT356788 | KT356766 | KT356799 | KT356744 | KT356733 | KT356722 |
| A/environment/Jiangxi/00449/2013 | H9N2 | environment |  |  | EPI_ISL_152855 | EPI_ISL_152855 | EPI_ISL_152855 | EPI_ISL_152855 | EPI_ISL_152855 | EPI_ISL_152855 |
| A/environment/Hunan/18355/2014 | H9N2 | environment |  |  | KT356784 | KT356762 | KT356795 | KT356740 | KT356729 | KT356718 |
| A/environment/Hunan/18498/2014 | H9N2 | environment |  |  | KT356787 | KT356765 | KT356798 | KT356743 | KT356732 | KT356721 |
| A/environment/Hunan/18489/2014 | H9N2 | environment |  |  | KT356786 | KT356764 | KT356797 | KT356742 | KT356731 | KT356720 |
| A/environment/Hunan/21403/2014 | H9N2 | environment |  |  | KT356789 | KT356767 | KT356800 | KT356745 | KT356734 | KT356723 |
| A/environment/Hunan/18370/2014 | H9N2 | environment |  |  | KT356785 | KT356763 | KT356796 | KT356741 | KT356730 | KT356719 |
| A/environment/Hunan/1-18/2007 | H9N2 | environment |  |  | GU474553 | GU474551 | GU474554 | GU474549 | GU474548 | GU474547 |
| A/environment/Hunan/1-23/2007 | H9N2 | environment |  |  | GU474561 | GU474559 | GU474562 | GU474557 | GU474556 | GU474555 |
| A/environment/Hunan/1-70/2007 | H9N2 | environment |  |  | GU474569 | GU474567 | GU474570 | GU474565 | GU474564 | GU474563 |
| A/environment/Hunan/1-81/2007 | H9N2 | environment |  |  | GU474577 | GU474575 | GU474578 | GU474573 | GU474572 | GU474571 |
| A/environment/Hunan/25998/2014 | H9N2 | environment |  |  | KT356790 | KT356768 | KT356801 | KT356746 | KT356735 | KT356724 |
| A/environment/Zhejiang/09/2013 | H9N2 | environment |  |  | KF178666 | KF178665 | KF178667 | KF178664 | KF178668 | KF178669 |
| A/environment/Zhejiang/13/2013 | H9N2 | environment |  |  | KF178674 | KF178673 | KF178675 | KF178672 | KF178676 | KF178677 |
| A/environment/Zhejiang/14/2013 | H9N2 | environment |  |  | KF178682 | KF178681 | KF178683 | KF178680 | KF178684 | KF178685 |
| A/environment/Zhejiang/15/2013 | H9N2 | environment |  |  | KF178690 | KF178689 | KF178691 | KF178688 | KF178692 | KF178693 |
| A/environment/Zhejiang/16/2013 | H9N2 | environment |  |  | KF178698 | KF178697 | KF178699 | KF178696 | KF178700 | KF178701 |
| A/environment/Bangladesh/19336/2013 | H9N2 | environment |  |  | KJ643615 | KJ643613 | KJ643616 | KJ643611 | KJ643610 | KJ643609 |
| A/environment/Hunan/26018/2014 | H9N2 | environment |  |  | KP289318 | KP289309 | KP289321 | KP289301 | KP289297 | KP289293 |
| A/environment/Hunan/27408/2014 | H9N2 | environment |  |  | KT356791 | KT356769 | KT356802 | KT356747 | KT356736 | KT356725 |
| A/environment/Hunan/27420/2014 | H9N2 | environment |  |  | KT356792 | KT356770 | KT356803 | KT356748 | KT356737 | KT356726 |
| A/environment/Guangdong/C13280011/2013 | H9N2 | environment |  |  | EPI_ISL_151431 | EPI_ISL_151431 | EPI_ISL_151431 | EPI_ISL_151431 | EPI_ISL_151431 | EPI_ISL_151431 |
| A/environment/Hunan/2-84/2007 | H9N2 | environment |  |  | GU474593 | GU474591 | GU474594 | GU474589 | GU474588 | GU474587 |
| A/feces/Hunan/2-28/2007 | H9N2 | environment |  |  | GU474585 | GU474583 | GU474586 | GU474581 | GU474580 | GU474579 |
| A/environment/Hunan/28034/2014 | H9N2 | environment |  |  | KT356794 | KT356772 | KT356805 | KT356750 | KT356739 | KT356728 |
| A/environment/Hunan/28176/2014 | H9N2 | environment |  |  | KP289317 | KP289308 | KP289320 | KP289300 | KP289296 | KP289292 |
| A/environment/Hunan/28184/2014 | H9N2 | environment |  |  | KP289316 | KP289307 | KP289319 | KP289299 | KP289295 | KP289291 |
| A/environment/Hunan/28028/2014 | H9N2 | environment |  |  | KT356793 | KT356771 | KT356804 | KT356749 | KT356738 | KT356727 |
| A/environment/Guangdong/GD130184/2013 | H9N2 | environment |  |  | EPI_ISL_151432 | EPI_ISL_151432 | EPI_ISL_151432 | EPI_ISL_151432 | EPI_ISL_151432 | EPI_ISL_151432 |
| A/environment/Guangdong/C13058252/2013 | H9N2 | environment |  |  | EPI_ISL_151437 | EPI_ISL_151437 | EPI_ISL_151437 | EPI_ISL_151437 | EPI_ISL_151437 | EPI_ISL_151437 |
| A/environment/Guangdong/C13058260/2013 | H9N2 | environment |  |  | EPI_ISL_151435 | EPI_ISL_151435 | EPI_ISL_151435 | EPI_ISL_151435 | EPI_ISL_151435 | EPI_ISL_151435 |
| A/environment/Guangdong/2013XN11652/2013 | H9N2 | environment |  |  | EPI_ISL_151436 | EPI_ISL_151436 | EPI_ISL_151436 | EPI_ISL_151436 | EPI_ISL_151436 | EPI_ISL_151436 |
| A/environment/Bangladesh/100/2010 | H9N2 | environment |  |  | EPI_ISL_141291 | EPI_ISL_141291 | EPI_ISL_141291 | EPI_ISL_141291 | EPI_ISL_141291 | EPI_ISL_141291 |
| A/environment/Guangdong/GD130206/2013 | H9N2 | environment |  |  | EPI_ISL_151433 | EPI_ISL_151433 | EPI_ISL_151433 | EPI_ISL_151433 | EPI_ISL_151433 | EPI_ISL_151433 |
| A/environment/Guangdong/GD130210/2013 | H9N2 | environment |  |  | EPI_ISL_151434 | EPI_ISL_151434 | EPI_ISL_151434 | EPI_ISL_151434 | EPI_ISL_151434 | EPI_ISL_151434 |
| A/environment/Zhongshan/ZS201601/2016 | H9N2 | environment |  |  | KX783318 | KX783300 | KX783327 | KX783282 | KX783273 | KX783264 |
| A/environment/Zhongshan/ZS201602/2016 | H9N2 | environment |  |  | KX783319 | KX783301 | KX783328 | KX783283 | KX783274 | KX783265 |
| A/environment/Zhongshan/ZS201603/2016 | H9N2 | environment |  |  | KX783320 | KX783302 | KX783329 | KX783284 | KX783275 | KX783266 |
| A/environment/Bangladesh/124/2010 | H9N2 | environment |  |  | EPI_ISL_165813 | EPI_ISL_165813 | EPI_ISL_165813 | EPI_ISL_165813 | EPI_ISL_165813 | EPI_ISL_165813 |
| A/environment/Jiangxi/14737/2014 | H9N2 | environment |  |  | EPI_ISL_237447 | EPI_ISL_237447 | EPI_ISL_237447 | EPI_ISL_237447 | EPI_ISL_237447 | EPI_ISL_237447 |
| A/environment/Jiangxi/02899/2012 | H9N2 | environment |  |  | EPI_ISL_152858 | EPI_ISL_152858 | EPI_ISL_152858 | EPI_ISL_152858 | EPI_ISL_152858 | EPI_ISL_152858 |
| A/environment/Bangladesh/23571/2014 | H9N2 | environment |  |  | KT361965 | KT361963 | KT361966 | KT361961 | KT361960 | KT361959 |
| A/environment/Bangladesh/155/2010 | H9N2 | environment |  |  | EPI_ISL_165814 | EPI_ISL_165814 | EPI_ISL_165814 | EPI_ISL_165814 | EPI_ISL_165814 | EPI_ISL_165814 |
| A/feces/Hunan/3-91/2007 | H9N2 | environment |  |  | GU474601 | GU474599 | GU474602 | GU474597 | GU474596 | GU474595 |
| A/environment/Hunan/20502/2013 | H9N2 | environment |  |  | KP289315 | KP289310 | KP289322 | KP289302 | KP289298 | KP289294 |
| A/environment/Zhongshan/ZS201501/2015 | H9N2 | environment |  |  | KX783312 | KX783294 | KX783321 | KX783276 | KX783267 | KX783258 |
| A/environment/Zhongshan/ZS201502/2015 | H9N2 | environment |  |  | KX783313 | KX783295 | KX783322 | KX783277 | KX783268 | KX783259 |
| A/environment/Zhongshan/ZS201503/2015 | H9N2 | environment |  |  | KX783314 | KX783296 | KX783323 | KX783278 | KX783269 | KX783260 |
| A/environment/Zhongshan/ZS201504/2015 | H9N2 | environment |  |  | KX783315 | KX783297 | KX783324 | KX783279 | KX783270 | KX783261 |
| A/environment/Zhongshan/ZS201505/2015 | H9N2 | environment |  |  | KX783316 | KX783298 | KX783325 | KX783280 | KX783271 | KX783262 |
| A/environment/Zhongshan/ZS201506/2015 | H9N2 | environment |  |  | KX783317 | KX783299 | KX783326 | KX783281 | KX783272 | KX783263 |
| A/environment/Hunan/5-38/2007 | H9N2 | environment |  |  | GU474609 | GU474607 | GU474610 | GU474605 | GU474604 | GU474603 |
| A/equine/Guangxi/3/2011 | H9N2 | Equine |  |  | JQ228392 | JQ228393 | JQ228394 | JQ228397 | JQ228395 | JQ228396 |
| A/ferret/Maryland/P10-UMD/2008 | H9N2 | Ferret |  |  | CY036280 | CY036278 | CY036281 | CY036276 | CY036275 | CY036274 |
| A/ferret/Maryland/P10-UMD_CIP046_RGJS07/2008 | H9N2 | Ferret |  |  | KX859442 | KX859373 | KX859327 | KX859376 | KX859420 | KX859311 |
| A/ferret/Maryland/P10-UMD_CIP046_RGJS08/2008 | H9N2 | Ferret |  |  | KX859383 | KX859340 | KX859331 | KX859390 | KX859353 | KX859396 |
| A/ferret/Maryland/P10-UMD/2012 | H9N2 | Ferret |  |  | KX838873 | KX838868 | KX838870 | KX838874 | KX838871 | KX838875 |
| A/Hong_Kong/1073/99 | H9N2 | Human |  |  | AJ278647 | AJ289871 | AJ278649 | AJ404637 | AJ404634 | AJ404630 |
| A/Hong_Kong/1074/1999 | H9N2 | Human |  |  | AF255364 | AJ289872 | AJ404735 | AJ404636 | AJ404635 | AJ404631 |
| A/Hong_Kong/33982/2009 | H9N2 | Human |  |  | KF188319 | KF188317 | KF188320 | KF188315 | KF188314 | KF188313 |
| A/Zhongshan/201501/2015 | H9N2 | Human |  |  | KU217317 | KU217320 | KU217319 | KU217321 | KU217322 | KU217323 |
| A/Bangladesh/0994/2011 | H9N2 | human |  |  | EPI_ISL_140388 | EPI_ISL_140388 | EPI_ISL_140388 | EPI_ISL_140388 | EPI_ISL_140388 | EPI_ISL_140388 |
| A/Guangdong/MZ058/2016 | H9N2 | Human |  |  | KX808591 | KX808589 | KX808592 | KX808587 | KX808586 | KX808585 |
| A/Guangdong/W1/2004 | H9N2 | Human |  |  | KX867852 | KX867850 | KX867853 | KX867848 | KX867847 | KX867846 |
| A/Lengshuitan/11197/2013 | H9N2 | Human |  |  | KM455875 | KM455873 | KM455876 | KM455871 | KM455870 | KM455869 |
| A/Hunan/44557/2015 | H9N2 | Human |  |  | KX595341 | KX595339 | KX595342 | KX595337 | KX595336 | KX595335 |
| A/Hong_Kong/308/2014 | H9N2 | human |  |  | EPI_ISL_153064 | EPI_ISL_153064 | EPI_ISL_153064 | EPI_ISL_153064 | EPI_ISL_153064 | EPI_ISL_153064 |
| A/mink/Shandong/F6/2013 | H9N2 | Mink |  |  | KM576105 | KM576107 | KM576106 | KM576108 | KM576109 | KM576110 |
| A/mink/Shandong/F10/2013 | H9N2 | Mink |  |  | KM576113 | KM576115 | KM576114 | KM576116 | KM576117 | KM576118 |
| A/swine/Hong_Kong/9/98 | H9N2 | Swine |  |  | KX879592 | KX879590 | KX879593 | KX879588 | KX879587 | KX879586 |
| A/swine/Shandong/w4/2003 | H9N2 | Swine |  |  | EU516306 | EU516308 | EU516307 | EU516311 | EU516310 | EU516309 |
| A/swine/Guangdong/wxl/2004 | H9N2 | Swine |  |  | EU516314 | EU516316 | EU516315 | EU516319 | EU516318 | EU516317 |
| A/swine/Jiangxi/1/2004 | H9N2 | Swine |  |  | EU502895 | EU502897 | EU502896 | EU502900 | EU502899 | EU502898 |
| A/swine/Jiangxi/wx2/2004 | H9N2 | Swine |  |  | EU502903 | EU502905 | EU502904 | EU502908 | EU502907 | EU502906 |
| A/swine/Korea/S452/2004 | H9N2 | Swine |  |  | AY790306 | AY790308 | AY790309 | AY790310 | AY790312 | AY790311 |
| A/swine/Guangxi/58/2005 | H9N2 | Swine |  |  | EF612743 | EF612745 | EF612746 | EF612747 | EF612748 | EF612749 |
| A/swine/Guangxi/FS2/2005 | H9N2 | Swine |  |  | EU086304 | EU086309 | EU086310 | EU086313 | EU086314 | EU086316 |
| A/swine/Guangxi/S11/2005 | H9N2 | Swine |  |  | EU086320 | EU086325 | EU086327 | EU086329 | EU086330 | EU086332 |
| A/swine/Guangxi/S15/2005 | H9N2 | Swine |  |  | EU086321 | EU086324 | EU086326 | EU086328 | EU086331 | EU086333 |
| A/swine/Guangdong/L1/2010 | H9N2 | Swine |  |  | HQ893765 | HQ893763 | HQ893766 | HQ893761 | HQ893760 | HQ893759 |
| A/swine/Guangxi/10/2007 | H9N2 | Swine |  |  | CY075057 | CY075055 | CY075058 | CY075053 | CY075052 | CY075051 |
| A/swine/Guangxi/7/2007 | H9N2 | Swine |  |  | CY075033 | CY075031 | CY075034 | CY075029 | CY075028 | CY075027 |
| A/swine/Guangxi/8/2007 | H9N2 | Swine |  |  | CY075041 | CY075039 | CY075042 | CY075037 | CY075036 | CY075035 |
| A/swine/Guangxi/9/2007 | H9N2 | Swine |  |  | CY075049 | CY075047 | CY075050 | CY075045 | CY075044 | CY075043 |
| A/swine/Hong_Kong/NS943/2010 | H9N2 | Swine |  |  | KM029506 | KM029504 | KM029507 | KM029502 | KM029501 | KM029500 |
| A/swine/Henan/Y1/2009 | H9N2 | Swine |  |  | KC779049 | KC779053 | KC779055 | KC779057 | KC779059 | KC779061 |
| A/swine/Yantai/16/2012 | H9N2 | Swine |  |  | KX421153 | KX421151 | KX421154 | KX421149 | KX421148 | KX421147 |
| A/swine/Jiangsu/C1/2008 | H9N2 | Swine |  |  | KX867828 | KX867826 | KX867829 | KX867824 | KX867823 | KX867822 |
| A/swine/Shanghai/Y1/2009 | H9N2 | Swine |  |  | KC779050 | KC779054 | KC779056 | KC779058 | KC779060 | KC779062 |
| A/mallard/Wisconsin/356/1984 | H11N9 | Avian |  | CY178601 |  |  |  |  |  |  |
| A/duck/Guangdong/4323/2007 | H11N9 | Avian |  | KF259712 |  |  |  |  |  |  |
| A/red-necked_stint/Western_Australia/5745/1982 | H12N9 | Avian |  | CY029875 |  |  |  |  |  |  |
| A/muscovy_duck/Thailand/CU-LM1984/2009 | H4N9 | Avian |  | CY062566 |  |  |  |  |  |  |
| A/mallard_duck/New_York/180/1986 | H4N9 | Avian |  | CY014859 |  |  |  |  |  |  |
| A/duck/Hunan/1590/2007 | H6N9 | Avian |  | CY109684 |  |  |  |  |  |  |
| A/turkey/Italy/2379/2000 | H7N1 | Avian | GU053007 |  |  |  |  |  |  |  |
| A/turkey/Italy/1083/1999 | H7N1 | Avian | KF493003 |  |  |  |  |  |  |  |
| A/chicken/Jilin/SD020/2014 | H7N2 | Avian | KM054796 |  |  |  |  |  |  |  |
| A/chicken/Anhui/A1180/2014 | H7N2 | Avian | KP765994 |  |  |  |  |  |  |  |
| A/chicken/Wuxi/SC6395/2015/H7N2 | H7N2 | avian | EPI_ISL_223201 |  |  |  |  |  |  |  |
| A/chicken/Wuxi/SC4315/2015/H7N2 | H7N2 | avian | EPI_ISL_223199 |  |  |  |  |  |  |  |
| A/chicken/Zhejiang/233/2016 | H7N6 | Avian | KY056290 |  |  |  |  |  |  |  |
| A/chicken/Jiangxi/14501/2014 | H7N6 | Avian | KP417953 |  |  |  |  |  |  |  |
| A/chicken/Jiangxi/14535/2014 | H7N6 | Avian | KP418022 |  |  |  |  |  |  |  |
| A/goose/Guangdong/7472/2012 | H7N7 | Avian | KF258958 |  |  |  |  |  |  |  |
| A/chicken/Wenzhou/189/2013 | H7N7 | Avian | KF259011 |  |  |  |  |  |  |  |
| A/duck/Wenzhou/47/2013 | H7N7 | Avian | KF259040 |  |  |  |  |  |  |  |
| A/environment/Fujian/S3307/2012 | H7N7 | environment | KX061824 |  |  |  |  |  |  |  |
